# Supplementary material for: Respiratory syncytial virus genotypes NA1, ON1, and BA9 are prevalent in Thailand, 2012–2015
Source: PeerJ. 2017 Oct 27;5:e3970. doi: 10.7717/peerj.3970 (PMC5661434; doi:10.7717/peerj.3970)
Supplement: Supplemental Information 1 [file peerj-05-3970-s001.pdf]

GenBank flat file:

```
LOCUS       KY327937                600 bp    cRNA    linear   VRL 13-DEC-2016
DEFINITION  Human respiratory syncytial virus A isolate TH-CU446/2012
            attachment glycoprotein gene, partial cds.
ACCESSION   KY327937
VERSION     KY327937
KEYWORDS    .
SOURCE      Human respiratory syncytial virus A
  ORGANISM  Human respiratory syncytial virus A
            Viruses; ssRNA viruses; ssRNA negative-strand viruses;
            Mononegavirales; Pneumoviridae; Orthopneumovirus.
REFERENCE   1  (bases 1 to 600)
  AUTHORS   Thongpan,I., Mauleekoonphairoj,J., Vichiwattana,P., Korkong,S.,
            Vongpunsawad,S. and Poovorawan,Y.
  TITLE     Molecular Characterization of Respiratory Syncytial Virus in
            Thailand, 2012-2015
  JOURNAL   Unpublished
REFERENCE   2  (bases 1 to 600)
  AUTHORS   Thongpan,I., Mauleekoonphairoj,J., Vichiwattana,P., Korkong,S.,
            Vongpunsawad,S. and Poovorawan,Y.
  TITLE     Direct Submission
  JOURNAL   Submitted (13-DEC-2016) Department of Pediatrics, Center of
            Excellence in Clinical Virology, Faculty of Medicine, Chulalongkorn
            University, Bangkok 10330, Thailand
COMMENT     ##Assembly-Data-START##
            Assembly Method       :: DNASTAR-Lasergene v. 6
            Sequencing Technology :: Sanger dideoxy sequencing
            ##Assembly-Data-END##
FEATURES             Location/Qualifiers
     source          1..600
                     /organism="Human respiratory syncytial virus A"
                     /mol_type="viral cRNA"
                     /isolate="TH-CU446/2012"
                     /host="Homo sapiens"
                     /db_xref="taxon:208893"
                     /country="Thailand"
                     /collection_date="01-Sep-2012"
                     /note="group: A"
     CDS             <1..600
                     /note="G protein"
                     /codon_start=1
                     /product="attachment glycoprotein"
                     /protein_id="APY20278"
                     /translation="ISFSNLSGTTSQSTTILASITPSAESTPQSTTVKIKNTTTTQIL
                     PSKPTTKQRQNKPQNKPNNDFHFEVFNFVPCSICSNNPTCWAICKRIPNKKPGKKTTT
                     KPTKKPTLKTTKKDPKPQTTKSKEVLTTKPTEKPTINTTKTNIRTTLLTSNTTENPEH
                     TSQEEILHSTTSEGYPSPSQVHTTSEHLSQSPSSSNTTK"
ORIGIN
        1 atcagcttct ccaatctgtc cggaactaca tcacaatcca ccaccatact agcttcaata
       61 acaccaagtg ctgagtcaac cccacaatcc acaacagtca agatcaaaaa cacaacaaca
      121 acccaaatat tacccagcaa acccactaca aaacaacgcc aaaataaacc acaaaacaaa
      181 cccaacaatg attttcactt tgaagtgttc aattttgtac cctgcagcat atgcagcaac
```

```
     241 aatccaacct gctgggccat ctgcaagaga ataccaaaca aaaaacctgg aaagaaaacc
     301 accaccaagc ccacaaaaaa accaaccctc aagacaacca aaaaagatcc caaacctcaa
     361 accacaaaat caaaggaagt actcaccacc aagcctacag aaaagccaac catcaacacc
     421 accaaaacaa acatcagaac tacactgctc acttccaaca ccacagaaaa tccagaacac
     481 acaagtcaag aggaaatcct ccactcaacc acctccgaag gctatccaag cccatcacaa
     541 gtccatacaa catccgagca cctatcacaa tctccatctt catccaacac aacaaaatga
//
LOCUS       KY327938                 600 bp    cRNA    linear   VRL 13-DEC-2016
DEFINITION  Human respiratory syncytial virus A isolate TH-CU401/2012
            attachment glycoprotein gene, partial cds.
ACCESSION   KY327938
VERSION     KY327938
KEYWORDS    .
SOURCE      Human respiratory syncytial virus A
  ORGANISM  Human respiratory syncytial virus A
            Viruses; ssRNA viruses; ssRNA negative-strand viruses;
            Mononegavirales; Pneumoviridae; Orthopneumovirus.
REFERENCE   1  (bases 1 to 600)
  AUTHORS   Thongpan,I., Mauleekoonphairoj,J., Vichiwattana,P., Korkong,S.,
            Vongpunsawad,S. and Poovorawan,Y.
  TITLE     Molecular Characterization of Respiratory Syncytial Virus in
            Thailand, 2012-2015
  JOURNAL   Unpublished
REFERENCE   2  (bases 1 to 600)
  AUTHORS   Thongpan,I., Mauleekoonphairoj,J., Vichiwattana,P., Korkong,S.,
            Vongpunsawad,S. and Poovorawan,Y.
  TITLE     Direct Submission
  JOURNAL   Submitted (13-DEC-2016) Department of Pediatrics, Center of
            Excellence in Clinical Virology, Faculty of Medicine, Chulalongkorn
            University, Bangkok 10330, Thailand
COMMENT     ##Assembly-Data-START##
            Assembly Method       :: DNASTAR-Lasergene v. 6
            Sequencing Technology :: Sanger dideoxy sequencing
            ##Assembly-Data-END##
FEATURES             Location/Qualifiers
     source          1..600
                     /organism="Human respiratory syncytial virus A"
                     /mol_type="viral cRNA"
                     /isolate="TH-CU401/2012"
                     /host="Homo sapiens"
                     /db_xref="taxon:208893"
                     /country="Thailand"
                     /collection_date="01-Aug-2012"
                     /note="group: A"
     CDS             <1..600
                     /note="G protein"
                     /codon_start=1
                     /product="attachment glycoprotein"
                     /protein_id="APY20279"
                     /translation="ISFSNLSGTTSQSTTILASITPSAESTPQSTTVKIKNTTTTQIL
                     PSKPTTKQRQNKPQNKPNNDFHFEVFNFVPCSICSNNPTCWAICKRIPNKKPGKKTTT
                     KPTKKPTLKTTKKDPKPQTTKSKEVLTTKPTEKPTINTTKTNIRTTLLTSNTTENPEH
                     TSQEETLHSTTSEGYLSPSQVYTTSEYLSQSPSSSNTTK"
ORIGIN
```

```
        1 atcagcttct ccaatctgtc cggaactaca tcacaatcca ccaccatact agcttcaata
       61 acaccaagtg ctgagtcaac ccccacaatcc acaacagtca agatcaaaaa cacaacaaca
      121 acccaaatat tacccagcaa acccactaca aaacaacgcc aaaataaacc acaaaacaaa
      181 cccaacaatg attttcactt tgaagtgttc aattttgtac cctgcagcat atgcagcaac
      241 aatccaacct gctgggccat ctgcaagaga ataccaaaca aaaaacctgg aaagaaaacc
      301 accaccaagc ccacaaaaaa accaaccctc aagacaacca aaaaagatcc caaacctcaa
      361 accacaaaat caaaggaagt actcaccacc aagcctacag aaaagccaac catcaacacc
      421 accaaaacaa acatcagaac tacactgctc acttccaaca ccacagaaaa tccagaacac
      481 acaagtcaag aggaaaccct ccactcaacc acctccgaag gctatctaag cccatcacaa
      541 gtctatacaa catccgagta cctatcacaa tctccatctt catccaacac aacaaaatga
//
LOCUS       KY327939                 600 bp    cRNA    linear   VRL 13-DEC-2016
DEFINITION  Human respiratory syncytial virus A isolate TH-CU381/2012
            attachment glycoprotein gene, partial cds.
ACCESSION   KY327939
VERSION     KY327939
KEYWORDS    .
SOURCE      Human respiratory syncytial virus A
  ORGANISM  Human respiratory syncytial virus A
            Viruses; ssRNA viruses; ssRNA negative-strand viruses;
            Mononegavirales; Pneumoviridae; Orthopneumovirus.
REFERENCE   1  (bases 1 to 600)
  AUTHORS   Thongpan,I., Mauleekoonphairoj,J., Vichiwattana,P., Korkong,S.,
            Vongpunsawad,S. and Poovorawan,Y.
  TITLE     Molecular Characterization of Respiratory Syncytial Virus in
            Thailand, 2012-2015
  JOURNAL   Unpublished
REFERENCE   2  (bases 1 to 600)
  AUTHORS   Thongpan,I., Mauleekoonphairoj,J., Vichiwattana,P., Korkong,S.,
            Vongpunsawad,S. and Poovorawan,Y.
  TITLE     Direct Submission
  JOURNAL   Submitted (13-DEC-2016) Department of Pediatrics, Center of
            Excellence in Clinical Virology, Faculty of Medicine, Chulalongkorn
            University, Bangkok 10330, Thailand
COMMENT     ##Assembly-Data-START##
            Assembly Method       :: DNASTAR-Lasergene v. 6
            Sequencing Technology :: Sanger dideoxy sequencing
            ##Assembly-Data-END##
FEATURES             Location/Qualifiers
     source          1..600
                     /organism="Human respiratory syncytial virus A"
                     /mol_type="viral cRNA"
                     /isolate="TH-CU381/2012"
                     /host="Homo sapiens"
                     /db_xref="taxon:208893"
                     /country="Thailand"
                     /collection_date="01-Jun-2012"
                     /note="group: A"
     CDS             <1..600
                     /note="G protein"
                     /codon_start=1
                     /product="attachment glycoprotein"
                     /protein_id="APY20280"
                     /translation="ISFSNLSGTTSQSTTILASITPSAESTPQSTTVKIKNTTTTQIL
```

file:///D|/Final%20peerJ/GenBank%20KY327937-KY328148_.txt[3/10/2560 14:59:50]

PSKPTTKQRQNKPQNKPNNDFHFEVFNFVPCSICSNNPTCWAICKRIPNKKPGKKTTT
KPTKKPTLKTTKKDPKPQTTKPKEVLTTKPTEKPTINTTKTNIRTTLLTSNTTGNPEH
TSQEETLHSTTSEGYLSPSQVYTTSEYLSQSPSSSNTTK"

ORIGIN

```
        1 atcagcttct ccaatctgtc cggaactaca tcacaatcca ccaccatact agcttcaata
       61 acaccaagtg ctgagtcaac cccacaatcc acaacagtca agatcaaaaa cacaacaaca
      121 acccaaatat tacctagcaa acccaccaca aaacaacgcc aaaataaacc acaaaacaaa
      181 cccaacaatg attttcactt tgaagtgttc aattttgtac cctgcagcat atgcagcaac
      241 aatccaacct gctgggccat ctgcaagaga ataccaaaca aaaaacctgg aaagaaaacc
      301 accaccaagc ccacaaaaaa accaaccctc aagacaacca aaaaagatcc caaacctcaa
      361 accacaaaac caaaggaagt actcaccacc aagcctacag aaaagccaac catcaacacc
      421 accaaaacaa acatcagaac tacactgctc acctccaaca ccacaggaaa tccagaacac
      481 acaagtcaag aggaaaccct ccactcaacc acctccgaag gctatctaag cccatcacaa
      541 gtctatacaa catccgagta cctatcacaa tctccatctt catccaacac aacaaaatga
```

//

LOCUS KY327940 600 bp cRNA linear VRL 13-DEC-2016

DEFINITION Human respiratory syncytial virus A isolate TH-CU281/2012
attachment glycoprotein gene, partial cds.

ACCESSION KY327940

VERSION KY327940

KEYWORDS .

SOURCE Human respiratory syncytial virus A

ORGANISM Human respiratory syncytial virus A
Viruses; ssRNA viruses; ssRNA negative-strand viruses;
Mononegavirales; Pneumoviridae; Orthopneumovirus.

REFERENCE 1 (bases 1 to 600)

AUTHORS Thongpan,I., Mauleekoonphairoj,J., Vichiwattana,P., Korkong,S.,
Vongpunsawad,S. and Poovorawan,Y.

TITLE Molecular Characterization of Respiratory Syncytial Virus in
Thailand, 2012-2015

JOURNAL Unpublished

REFERENCE 2 (bases 1 to 600)

AUTHORS Thongpan,I., Mauleekoonphairoj,J., Vichiwattana,P., Korkong,S.,
Vongpunsawad,S. and Poovorawan,Y.

TITLE Direct Submission

JOURNAL Submitted (13-DEC-2016) Department of Pediatrics, Center of
Excellence in Clinical Virology, Faculty of Medicine, Chulalongkorn
University, Bangkok 10330, Thailand

COMMENT ##Assembly-Data-START##
Assembly Method :: DNASTAR-Lasergene v. 6
Sequencing Technology :: Sanger dideoxy sequencing
##Assembly-Data-END##

FEATURES Location/Qualifiers

source 1..600
/organism="Human respiratory syncytial virus A"
/mol_type="viral cRNA"
/isolate="TH-CU281/2012"
/host="Homo sapiens"
/db_xref="taxon:208893"
/country="Thailand"
/collection_date="01-Feb-2012"
/note="group: A"

CDS <1..600
/note="G protein"

```
                     /codon_start=1
                     /product="attachment glycoprotein"
                     /protein_id="APY20281"
                     /translation="ISFSNLSGTTSQSTTILASITPSAESTPQSTTVKIKNTTTTQIL
                     PSKPTTKQRQNKPQNKPNNDFHFEVFNFVPCSICSNNPTCWAICKRIPNKKPGKKTTT
                     KPTKKPTLKTTKKDPKPQTTKPKEVLTTKPTEKPTINTTKTNIRTTLLTSNTTGNPEH
                     TSQEETLHSTTSEGYLSPSQVYTTSEYLSQSPSSSNTTK"
ORIGIN
        1 atcagcttct ccaatctgtc cggaactaca tcacaatcca ccaccatact agcttcaata
       61 acaccaagtg ctgagtcaac cccacaatcc acaacagtca agatcaaaaa tacaacaaca
      121 acccaaatat tacctagcaa acccaccaca aaacaacgcc aaaataaacc acaaaacaaa
      181 cccaacaatg attttcactt tgaagtgttc aattttgtac cctgcagcat atgcagcaac
      241 aatccaacct gctgggccat ctgcaagaga ataccaaaca aaaaacctgg aaagaaaacc
      301 accaccaagc ctacaaaaaa accaaccctc aagacaacca aaaaagatcc caaacctcaa
      361 accacaaaac caaaggaagt actcaccacc aagcctacag aaaagccaac catcaacacc
      421 accaaaacaa acatcagaac cacactgctc acctccaaca ccacaggaaa tccagaacac
      481 acaagtcaag aggaaaccct ccactcaacc acctccgaag gctatctaag cccatcacaa
      541 gtctatacaa catccgagta cctatcacaa tctccatctt catccaacac aacaaaatga
//
LOCUS       KY327941                 600 bp    cRNA    linear   VRL 13-DEC-2016
DEFINITION  Human respiratory syncytial virus A isolate TH-CU/C3211/2012
            attachment glycoprotein gene, partial cds.
ACCESSION   KY327941
VERSION     KY327941
KEYWORDS    .
SOURCE      Human respiratory syncytial virus A
  ORGANISM  Human respiratory syncytial virus A
            Viruses; ssRNA viruses; ssRNA negative-strand viruses;
            Mononegavirales; Pneumoviridae; Orthopneumovirus.
REFERENCE   1  (bases 1 to 600)
  AUTHORS   Thongpan,I., Mauleekoonphairoj,J., Vichiwattana,P., Korkong,S.,
            Vongpunsawad,S. and Poovorawan,Y.
  TITLE     Molecular Characterization of Respiratory Syncytial Virus in
            Thailand, 2012-2015
  JOURNAL   Unpublished
REFERENCE   2  (bases 1 to 600)
  AUTHORS   Thongpan,I., Mauleekoonphairoj,J., Vichiwattana,P., Korkong,S.,
            Vongpunsawad,S. and Poovorawan,Y.
  TITLE     Direct Submission
  JOURNAL   Submitted (13-DEC-2016) Department of Pediatrics, Center of
            Excellence in Clinical Virology, Faculty of Medicine, Chulalongkorn
            University, Bangkok 10330, Thailand
COMMENT     ##Assembly-Data-START##
            Assembly Method       :: DNASTAR-Lasergene v. 6
            Sequencing Technology :: Sanger dideoxy sequencing
            ##Assembly-Data-END##
FEATURES             Location/Qualifiers
     source          1..600
                     /organism="Human respiratory syncytial virus A"
                     /mol_type="viral cRNA"
                     /isolate="TH-CU/C3211/2012"
                     /host="Homo sapiens"
                     /db_xref="taxon:208893"
                     /country="Thailand"
```

```
                     /collection_date="01-Sep-2012"
                     /note="group: A"
     CDS             <1..600
                     /note="G protein"
                     /codon_start=1
                     /product="attachment glycoprotein"
                     /protein_id="APY20282"
                     /translation="ISFSNLSGTTSQSTTILASTTPSAESTPQSTIVKIKNTTTTQIL
                     PSKPTTKQRQNKPQNKPNNDFHFEVFNFVPCSICSNNPTCWAICKRIPNKKPGKKTTT
                     KPTKKPTLKTTKKDPKLQTTKPKEVLTTKPTEKPTIDTTKTNIRTTLLTSNTTGNPEH
                     TSQEETLHSTTSEGYLSPSQVYTTSEYLSQSPSSSNTTK"
ORIGIN
        1 atcagcttct ccaatctgtc cggaactaca tcacaatcca ccaccatact agcttcaaca
       61 acaccaagtg ctgagtcaac cccacaatcc acaatagtca agatcaaaaa cacaacaaca
      121 acccaaatat tacctagcaa acccaccaca aaacaacgcc aaaataaacc acaaaacaaa
      181 cccaacaatg attttcactt tgaagtgttc aattttgtac cctgcagcat atgcagcaac
      241 aatccaacct gctgggctat ctgcaagaga ataccaaaca aaaaacctgg aaagaaaacc
      301 accaccaagc ccacaaaaaa accaaccctc aagacaacca aaaaagatcc caaacttcaa
      361 accacaaaac caaaggaagt actcaccacc aagcctacag aaaagccaac catcgacacc
      421 accaaaacaa acatcagaac tacactgctc acctccaaca ccacaggaaa tccagaacac
      481 acaagtcaag aggaaaccct ccactcaacc acctccgaag gctatctaag cccatcacaa
      541 gtctatacaa catccgagta cctatcacaa tctccatctt catccaacac aacaaaatga
//
LOCUS       KY327942                 600 bp    cRNA    linear   VRL 13-DEC-2016
DEFINITION  Human respiratory syncytial virus A isolate TH-CU408/2012
            attachment glycoprotein gene, partial cds.
ACCESSION   KY327942
VERSION     KY327942
KEYWORDS    .
SOURCE      Human respiratory syncytial virus A
  ORGANISM  Human respiratory syncytial virus A
            Viruses; ssRNA viruses; ssRNA negative-strand viruses;
            Mononegavirales; Pneumoviridae; Orthopneumovirus.
REFERENCE   1  (bases 1 to 600)
  AUTHORS   Thongpan,I., Mauleekoonphairoj,J., Vichiwattana,P., Korkong,S.,
            Vongpunsawad,S. and Poovorawan,Y.
  TITLE     Molecular Characterization of Respiratory Syncytial Virus in
            Thailand, 2012-2015
  JOURNAL   Unpublished
REFERENCE   2  (bases 1 to 600)
  AUTHORS   Thongpan,I., Mauleekoonphairoj,J., Vichiwattana,P., Korkong,S.,
            Vongpunsawad,S. and Poovorawan,Y.
  TITLE     Direct Submission
  JOURNAL   Submitted (13-DEC-2016) Department of Pediatrics, Center of
            Excellence in Clinical Virology, Faculty of Medicine, Chulalongkorn
            University, Bangkok 10330, Thailand
COMMENT     ##Assembly-Data-START##
            Assembly Method       :: DNASTAR-Lasergene v. 6
            Sequencing Technology :: Sanger dideoxy sequencing
            ##Assembly-Data-END##
FEATURES             Location/Qualifiers
     source          1..600
                     /organism="Human respiratory syncytial virus A"
                     /mol_type="viral cRNA"
```

/isolate="TH-CU408/2012"
/host="Homo sapiens"
/db_xref="taxon:208893"
/country="Thailand"
/collection_date="01-Aug-2012"
/note="group: A"
CDS <1..600
/note="G protein"
/codon_start=1
/product="attachment glycoprotein"
/protein_id="APY20283"
/translation="ISFSNLSGTTSQSTTILASTTPSAESIPQSTTVKIKNTTTTQIL
PSKPTTKQRQNKPQNKPNNDFHFEVFNFVPCSICSNNPTCWAICKRIPNKKPGKKTTT
KPTKKPTLKTTKKDPKPQTTKPKEVLTTKPTEKPTIDTTKTNIRTTLLTSNTTGNPEH
TSQEETLHSTTSEGYLSPSQVYTTSEYLSQYPSSSNTTK"
ORIGIN
1 atcagcttct ccaatctgtc cggaactaca tcacaatcca ccaccatact agcttcaaca
61 acaccaagtg ctgagtcaat cccacaatcc acaacagtca agatcaaaaa cacaacaaca
121 acccaaatat tacccagcaa acccaccaca aaacaacgcc aaaacaaacc acaaaacaaa
181 cccaacaatg attttcactt tgaagtgttc aattttgtac cctgcagcat atgcagcaac
241 aatccaacct gctgggccat ctgcaagaga ataccaaaca aaaaacctgg aaagaaaacc
301 accaccaagc ccacaaaaaa accaaccctc aagacaacca aaaaagatcc caaacctcaa
361 accacaaaac caaaggaagt actcaccacc aagcccacag aaaaaccaac catcgacacc
421 accaaaacaa acatcagaac tacactgctc acctccaaca ccacaggaaa tccagaacac
481 acaagtcaag aggaaaccct ccactcaacc acctccgaag gctatctaag cccatcacaa
541 gtctatacaa catccgagta cctatcacaa tatccatctt catccaacac aacaaaatga
//
LOCUS KY327943 600 bp cRNA linear VRL 13-DEC-2016
DEFINITION Human respiratory syncytial virus A isolate TH-CU/C3180/2012
attachment glycoprotein gene, partial cds.
ACCESSION KY327943
VERSION KY327943
KEYWORDS .
SOURCE Human respiratory syncytial virus A
ORGANISM Human respiratory syncytial virus A
Viruses; ssRNA viruses; ssRNA negative-strand viruses;
Mononegavirales; Pneumoviridae; Orthopneumovirus.
REFERENCE 1 (bases 1 to 600)
AUTHORS Thongpan,I., Mauleekoonphairoj,J., Vichiwattana,P., Korkong,S.,
Vongpunsawad,S. and Poovorawan,Y.
TITLE Molecular Characterization of Respiratory Syncytial Virus in
Thailand, 2012-2015
JOURNAL Unpublished
REFERENCE 2 (bases 1 to 600)
AUTHORS Thongpan,I., Mauleekoonphairoj,J., Vichiwattana,P., Korkong,S.,
Vongpunsawad,S. and Poovorawan,Y.
TITLE Direct Submission
JOURNAL Submitted (13-DEC-2016) Department of Pediatrics, Center of
Excellence in Clinical Virology, Faculty of Medicine, Chulalongkorn
University, Bangkok 10330, Thailand
COMMENT ##Assembly-Data-START##
Assembly Method :: DNASTAR-Lasergene v. 6
Sequencing Technology :: Sanger dideoxy sequencing
##Assembly-Data-END##

```
FEATURES             Location/Qualifiers
     source          1..600
                     /organism="Human respiratory syncytial virus A"
                     /mol_type="viral cRNA"
                     /isolate="TH-CU/C3180/2012"
                     /host="Homo sapiens"
                     /db_xref="taxon:208893"
                     /country="Thailand"
                     /collection_date="01-Sep-2012"
                     /note="group: A"
     CDS             <1..600
                     /note="G protein"
                     /codon_start=1
                     /product="attachment glycoprotein"
                     /protein_id="APY20284"
                     /translation="ISFSNLSGTTSQSTTILASTTPSAESTPQSTTVKIKNTTTTQIQ
                     PSKPTTKQRQNKPQNKPNNDFHFEVFNFVPCSICSNNPTCWAICKRIPNKKPGKKTTT
                     KPTKKPTIKTTKKDPKPQTTKPKEVLTTKSTEKPTIYTTKTNIRTTLLTSNTTGNPEH
                     TSQEETLHSTTSEGNLSPSQVYTTSEYLSQSPSSSNTSK"
ORIGIN
        1 atcagcttct ctaatctgtc cggaactaca tcacaatcca ccaccatact agcttcaaca
       61 acaccaagtg ctgagtcaac cccacaatcc acaacagtca agatcaaaaa cacaacaaca
      121 acccaaatac aacctagcaa acccaccaca aaacaacgcc aaaataaacc acaaaacaaa
      181 cccaacaatg attttcactt tgaagtgttc aattttgtac cctgcagcat atgcagcaac
      241 aatccaacct gctgggccat ctgcaagaga ataccaaaca aaaaacctgg aaagaaaacc
      301 accaccaagc ccacaaaaaa accaaccatc aagacaacca aaaaagatcc caaacctcaa
      361 accacaaaac caaaggaagt actcaccacc aagtccacag aaaagccaac catctacacc
      421 accaaaacaa acattagaac tacactgctc acctccaaca ccacaggaaa tccagaacac
      481 acaagtcaag aggaaaccct ccactcaacc acctccgaag gcaatctaag cccatcacaa
      541 gtctatacaa catccgagta cctatcacaa tctccatctt catccaacac atcaaaatga
//
LOCUS       KY327944                 600 bp    cRNA    linear   VRL 13-DEC-2016
DEFINITION  Human respiratory syncytial virus A isolate TH-CU358/2012
            attachment glycoprotein gene, partial cds.
ACCESSION   KY327944
VERSION     KY327944
KEYWORDS    .
SOURCE      Human respiratory syncytial virus A
  ORGANISM  Human respiratory syncytial virus A
            Viruses; ssRNA viruses; ssRNA negative-strand viruses;
            Mononegavirales; Pneumoviridae; Orthopneumovirus.
REFERENCE   1  (bases 1 to 600)
  AUTHORS   Thongpan,I., Mauleekoonphairoj,J., Vichiwattana,P., Korkong,S.,
            Vongpunsawad,S. and Poovorawan,Y.
  TITLE     Molecular Characterization of Respiratory Syncytial Virus in
            Thailand, 2012-2015
  JOURNAL   Unpublished
REFERENCE   2  (bases 1 to 600)
  AUTHORS   Thongpan,I., Mauleekoonphairoj,J., Vichiwattana,P., Korkong,S.,
            Vongpunsawad,S. and Poovorawan,Y.
  TITLE     Direct Submission
  JOURNAL   Submitted (13-DEC-2016) Department of Pediatrics, Center of
            Excellence in Clinical Virology, Faculty of Medicine, Chulalongkorn
            University, Bangkok 10330, Thailand
```

```
COMMENT     ##Assembly-Data-START##
            Assembly Method       :: DNASTAR-Lasergene v. 6
            Sequencing Technology :: Sanger dideoxy sequencing
            ##Assembly-Data-END##
FEATURES             Location/Qualifiers
     source          1..600
                     /organism="Human respiratory syncytial virus A"
                     /mol_type="viral cRNA"
                     /isolate="TH-CU358/2012"
                     /host="Homo sapiens"
                     /db_xref="taxon:208893"
                     /country="Thailand"
                     /collection_date="01-May-2012"
                     /note="group: A"
     CDS             <1..600
                     /note="G protein"
                     /codon_start=1
                     /product="attachment glycoprotein"
                     /protein_id="APY20285"
                     /translation="ISFSNLSGTTSQSTTILASTTPSAESTPQSTTVKIKNTTTTQIQ
                     PSKPTTKQRQNKPQNKPNNDFHFEVFNFVPCSICSNNPTCWAICNRIPNKKPGKKTTT
                     KPTKKPTIKITKKDPKPQTTKPKEVLTTKPTEKPTIYTTKTNIRTTLLTSNTTGNPEH
                     TSQEETLHSTTSEGNLSPSQVYTTSEYLSQSPSSSNTSK"
ORIGIN
        1 atcagcttct ccaatctgtc cggaactaca tcacaatcca ccaccatact agcttcaaca
       61 acaccaagtg ctgagtcaac cccacaatcc acaacagtca agatcaaaaa cacaactaca
      121 acccaaatac aacctagcaa acccaccaca aaacaacgcc aaaataaacc acaaaacaaa
      181 cccaacaatg attttcactt tgaagtgttc aattttgtac cctgcagcat atgcagcaac
      241 aatccaacct gctgggccat ctgcaataga ataccaaaca aaaaacctgg aaagaaaacc
      301 accaccaagc ccacaaaaaa accaaccatc aagataacca aaaaagatcc caaacctcaa
      361 accacaaaac caaaggaagt actcaccacc aagcccacag aaaagccaac catctacacc
      421 accaaaacaa acatcagaac tacactgctc acctccaaca ccacaggaaa tccagaacac
      481 acaagtcaag aggaaaccct ccactcaacc acctccgaag gcaatctaag cccatcacaa
      541 gtctatacaa catccgagta cctatcacaa tctccatctt catccaacac atcaaaatga
//
LOCUS       KY327945                 600 bp    cRNA    linear   VRL 13-DEC-2016
DEFINITION  Human respiratory syncytial virus A isolate TH-CU/CB128/2013
            attachment glycoprotein gene, partial cds.
ACCESSION   KY327945
VERSION     KY327945
KEYWORDS    .
SOURCE      Human respiratory syncytial virus A
  ORGANISM  Human respiratory syncytial virus A
            Viruses; ssRNA viruses; ssRNA negative-strand viruses;
            Mononegavirales; Pneumoviridae; Orthopneumovirus.
REFERENCE   1  (bases 1 to 600)
  AUTHORS   Thongpan,I., Mauleekoonphairoj,J., Vichiwattana,P., Korkong,S.,
            Vongpunsawad,S. and Poovorawan,Y.
  TITLE     Molecular Characterization of Respiratory Syncytial Virus in
            Thailand, 2012-2015
  JOURNAL   Unpublished
REFERENCE   2  (bases 1 to 600)
  AUTHORS   Thongpan,I., Mauleekoonphairoj,J., Vichiwattana,P., Korkong,S.,
            Vongpunsawad,S. and Poovorawan,Y.
```

```
  TITLE     Direct Submission
  JOURNAL   Submitted (13-DEC-2016) Department of Pediatrics, Center of
            Excellence in Clinical Virology, Faculty of Medicine, Chulalongkorn
            University, Bangkok 10330, Thailand
COMMENT     ##Assembly-Data-START##
            Assembly Method       :: DNASTAR-Lasergene v. 6
            Sequencing Technology :: Sanger dideoxy sequencing
            ##Assembly-Data-END##
FEATURES             Location/Qualifiers
     source          1..600
                     /organism="Human respiratory syncytial virus A"
                     /mol_type="viral cRNA"
                     /isolate="TH-CU/CB128/2013"
                     /host="Homo sapiens"
                     /db_xref="taxon:208893"
                     /country="Thailand"
                     /collection_date="01-Nov-2012"
                     /note="group: A"
     CDS             <1..600
                     /note="G protein"
                     /codon_start=1
                     /product="attachment glycoprotein"
                     /protein_id="APY20286"
                     /translation="ISFSNLSETTSQSTTILASTTPSAEPTPQSTTVKIKNTTTTQIQ
                     PSKPTTKQRQNKPQNKPNNDFHFEVFNFVPCSICSNNPTCWAICKRIPNKKPGKKTTT
                     KPTKKPTIKTTKKDPKPQTTKPKEVLTTKPTEKPTIYTTKTNIRTTLLTSNTTGNPEH
                     TSQEETLHSTTSEGNLSPSQVYTTSEYLSQSPSSSNTSK"
ORIGIN
        1 atcagcttct ccaatctgtc cgaaactaca tcacaatcca ccaccatact agcttcaaca
       61 acaccaagtg ctgagccaac cccacaatcc acaacagtca agatcaaaaa cacaacaaca
      121 acccaaatac aacctagcaa acccaccaca aaacaacgcc aaaacaaacc acaaaacaaa
      181 cccaacaatg attttcactt tgaagtgttc aattttgtac cctgtagcat atgcagcaac
      241 aatccaacct gctgggccat ctgcaagaga ataccaaaca aaaaacctgg aaagaaaacc
      301 accaccaagc ccacaaaaaa accaaccatc aagacaacca aaaaagatcc caaacctcaa
      361 accacaaaac caaaggaagt actcaccacc aagcccacag aaaagccaac catctacacc
      421 accaaaacaa acatcagaac tacactgctc acctccaaca ccacaggaaa tccagaacac
      481 acaagtcaag aggaaaccct ccactcaacc acctccgaag gcaatctaag cccatcacaa
      541 gtctatacaa catccgagta cctatcacaa tctccatctt catccaacac atcaaaatga
//
LOCUS       KY327946                 600 bp    cRNA    linear   VRL 13-DEC-2016
DEFINITION  Human respiratory syncytial virus A isolate TH-CU/C3292/2012
            attachment glycoprotein gene, partial cds.
ACCESSION   KY327946
VERSION     KY327946
KEYWORDS    .
SOURCE      Human respiratory syncytial virus A
  ORGANISM  Human respiratory syncytial virus A
            Viruses; ssRNA viruses; ssRNA negative-strand viruses;
            Mononegavirales; Pneumoviridae; Orthopneumovirus.
REFERENCE   1  (bases 1 to 600)
  AUTHORS   Thongpan,I., Mauleekoonphairoj,J., Vichiwattana,P., Korkong,S.,
            Vongpunsawad,S. and Poovorawan,Y.
  TITLE     Molecular Characterization of Respiratory Syncytial Virus in
            Thailand, 2012-2015
```

```
  JOURNAL   Unpublished
REFERENCE   2  (bases 1 to 600)
  AUTHORS   Thongpan,I., Mauleekoonphairoj,J., Vichiwattana,P., Korkong,S.,
            Vongpunsawad,S. and Poovorawan,Y.
  TITLE     Direct Submission
  JOURNAL   Submitted (13-DEC-2016) Department of Pediatrics, Center of
            Excellence in Clinical Virology, Faculty of Medicine, Chulalongkorn
            University, Bangkok 10330, Thailand
COMMENT     ##Assembly-Data-START##
            Assembly Method       :: DNASTAR-Lasergene v. 6
            Sequencing Technology :: Sanger dideoxy sequencing
            ##Assembly-Data-END##
FEATURES             Location/Qualifiers
     source          1..600
                     /organism="Human respiratory syncytial virus A"
                     /mol_type="viral cRNA"
                     /isolate="TH-CU/C3292/2012"
                     /host="Homo sapiens"
                     /db_xref="taxon:208893"
                     /country="Thailand"
                     /collection_date="01-Oct-2012"
                     /note="group: A"
     CDS             <1..600
                     /note="G protein"
                     /codon_start=1
                     /product="attachment glycoprotein"
                     /protein_id="APY20287"
                     /translation="ISFSNLSGTTSQSTTILASTTPSAESTPQSTTVKIKTTTTTQIQ
                     PSKPTTKQRQNKPQNKPNNDFHFEVFNFVPCSICSNNPTCWAICKRIPNKKPGKKTTT
                     KPTKKSTIKTTKKDPKPQTTKPKEVLTTKPTEKPTIDTTKTNIRTTLLTFNTTGNPEH
                     TSQEETLHSTTSEGNLSPSQVYTTSEYLSQSPSSSNTIK"
ORIGIN
        1 atcagcttct ccaatctgtc cggaactaca tcacaatcca ccaccatact agcttcaaca
       61 acaccaagtg ctgagtcaac cccacaatcc acaacagtca agatcaaaac cacaacaaca
      121 acccaaatac aacctagcaa acccaccaca aaacaacgcc aaaataaacc acaaaacaaa
      181 cccaacaatg attttcactt tgaagtgttc aattttgtac cctgcagcat atgcagcaac
      241 aatccaacct gctgggccat ctgcaagaga ataccaaaca aaaaacctgg aaagaaaacc
      301 accactaagc ccacaaaaaa atcaaccatc aagacaacca aaaaagatcc caaacctcaa
      361 accacaaaac caaaggaagt actcaccacc aagcccacag aaaagccaac catcgacacc
      421 accaaaacaa acatcagaac tacactgctc accttcaaca ccacaggaaa tccagaacac
      481 acaagtcaag aggaaaccct ccactcaacc acctccgaag gcaatctaag cccatcacaa
      541 gtctatacaa catccgagta cctatcacaa tctccatctt catccaacac aataaaatga
//
LOCUS       KY327947                 600 bp    cRNA    linear   VRL 13-DEC-2016
DEFINITION  Human respiratory syncytial virus A isolate TH-CU/C3133/2012
            attachment glycoprotein gene, partial cds.
ACCESSION   KY327947
VERSION     KY327947
KEYWORDS    .
SOURCE      Human respiratory syncytial virus A
  ORGANISM  Human respiratory syncytial virus A
            Viruses; ssRNA viruses; ssRNA negative-strand viruses;
            Mononegavirales; Pneumoviridae; Orthopneumovirus.
REFERENCE   1  (bases 1 to 600)
```

```
  AUTHORS   Thongpan,I., Mauleekoonphairoj,J., Vichiwattana,P., Korkong,S.,
            Vongpunsawad,S. and Poovorawan,Y.
  TITLE     Molecular Characterization of Respiratory Syncytial Virus in
            Thailand, 2012-2015
  JOURNAL   Unpublished
REFERENCE   2  (bases 1 to 600)
  AUTHORS   Thongpan,I., Mauleekoonphairoj,J., Vichiwattana,P., Korkong,S.,
            Vongpunsawad,S. and Poovorawan,Y.
  TITLE     Direct Submission
  JOURNAL   Submitted (13-DEC-2016) Department of Pediatrics, Center of
            Excellence in Clinical Virology, Faculty of Medicine, Chulalongkorn
            University, Bangkok 10330, Thailand
COMMENT     ##Assembly-Data-START##
            Assembly Method       :: DNASTAR-Lasergene v. 6
            Sequencing Technology :: Sanger dideoxy sequencing
            ##Assembly-Data-END##
FEATURES             Location/Qualifiers
     source          1..600
                     /organism="Human respiratory syncytial virus A"
                     /mol_type="viral cRNA"
                     /isolate="TH-CU/C3133/2012"
                     /host="Homo sapiens"
                     /db_xref="taxon:208893"
                     /country="Thailand"
                     /collection_date="01-Aug-2012"
                     /note="group: A"
     CDS             <1..600
                     /note="G protein"
                     /codon_start=1
                     /product="attachment glycoprotein"
                     /protein_id="APY20288"
                     /translation="ISFSNLSGTTSQSTTILASTTPSAESTPQSTTVKIKTTTTTQIQ
                     PSKPTTKQRQNKPQNKPNNDFHFEVFNFVPCSICSNNPTCWAICKRIPNKKPGKKTTT
                     KPTKKSTIKTTKKDPKPQTTKPKEVLTTKPTEKPTINTTKTNIRTTLLTFNTTGNPEH
                     TSQEETLHSTTSEGNLSPSQVYTTSEYLSQSPSSSNTIK"
ORIGIN
        1 atcagcttct ccaatctgtc cggaactaca tcacaatcca ccaccatact agcttcaaca
       61 acaccaagtg ctgagtcaac ccccacaatcc acaacagtca agatcaaaac cacaacaaca
      121 acccaaatac aacctagcaa acccaccaca aaacaacgcc aaaataaacc acaaaacaaa
      181 cccaacaatg attttcactt tgaagtgttc aattttgtac cctgcagcat atgcagcaac
      241 aatccaacct gctgggccat ctgcaagaga ataccaaaca aaaaacctgg aaagaaaacc
      301 accactaagc ccacaaaaaa atcaaccatc aagacaacca aaaaagatcc caaacctcaa
      361 accacaaaac caaaggaagt actcaccacc aagcccacag aaaagccaac catcaacacc
      421 accaaaacaa acatcagaac tacactgctc accttcaaca ccacaggaaa tccagaacac
      481 acaagtcaag aggaaaccct ccactcaacc acctccgaag gcaatctaag cccatcacaa
      541 gtctatacaa catccgagta cctatcacaa tctccatctt catccaacac aataaaatga
//
LOCUS       KY327948                 600 bp    cRNA    linear   VRL 13-DEC-2016
DEFINITION  Human respiratory syncytial virus A isolate TH-CU/C3134/2012
            attachment glycoprotein gene, partial cds.
ACCESSION   KY327948
VERSION     KY327948
KEYWORDS    .
SOURCE      Human respiratory syncytial virus A
```

```
  ORGANISM  Human respiratory syncytial virus A
            Viruses; ssRNA viruses; ssRNA negative-strand viruses;
            Mononegavirales; Pneumoviridae; Orthopneumovirus.
REFERENCE   1  (bases 1 to 600)
  AUTHORS   Thongpan,I., Mauleekoonphairoj,J., Vichiwattana,P., Korkong,S.,
            Vongpunsawad,S. and Poovorawan,Y.
  TITLE     Molecular Characterization of Respiratory Syncytial Virus in
            Thailand, 2012-2015
  JOURNAL   Unpublished
REFERENCE   2  (bases 1 to 600)
  AUTHORS   Thongpan,I., Mauleekoonphairoj,J., Vichiwattana,P., Korkong,S.,
            Vongpunsawad,S. and Poovorawan,Y.
  TITLE     Direct Submission
  JOURNAL   Submitted (13-DEC-2016) Department of Pediatrics, Center of
            Excellence in Clinical Virology, Faculty of Medicine, Chulalongkorn
            University, Bangkok 10330, Thailand
COMMENT     ##Assembly-Data-START##
            Assembly Method       :: DNASTAR-Lasergene v. 6
            Sequencing Technology :: Sanger dideoxy sequencing
            ##Assembly-Data-END##
FEATURES             Location/Qualifiers
     source          1..600
                     /organism="Human respiratory syncytial virus A"
                     /mol_type="viral cRNA"
                     /isolate="TH-CU/C3134/2012"
                     /host="Homo sapiens"
                     /db_xref="taxon:208893"
                     /country="Thailand"
                     /collection_date="01-Aug-2012"
                     /note="group: A"
     CDS             <1..600
                     /note="G protein"
                     /codon_start=1
                     /product="attachment glycoprotein"
                     /protein_id="APY20289"
                     /translation="ISFSNLSGTTSQSTTILASTTPSAESTPQSTTVKIKNTTTTQIQ
                     PSKPTTKQRQNKPQNKPNNDFHFEVFNFVPCSICSNNPTCWAICKRIPNKKPGKKTTT
                     KPTKKPTIKTTKKDPKPQTTKPKEVLTTKPTEKPTIDTTKTNIRTTLLTSYTTGNQEH
                     TSQEETLHSTTSEGNLSPSQVYTTSEYLSQSPSSSYTTK"
ORIGIN
        1 atcagcttct ccaatctgtc cggaactaca tcacaatcca ccaccatact agcttcaaca
       61 acaccaagtg ctgagtcaac cccacaatcc acaacagtca agatcaaaaa cacaacaaca
      121 acccaaatac aacctagcaa acccaccaca aaacaacgcc aaaataaacc acaaaacaaa
      181 cccaacaatg attttcactt tgaagtgttc aactttgtac cctgcagcat atgcagcaac
      241 aatccaacct gctgggctat ctgcaagaga ataccaaaca aaaaacctgg aaagaaaact
      301 accaccaagc ccacaaagaa accaaccatc aagacaacca aaaaagatcc caaacctcaa
      361 accacaaaac caaaggaagt actcaccacc aagcccacag aaaagccaac catcgacacc
      421 accaaaacaa acatcagaac tacactgctc acctcctaca ctacaggaaa tcaagaacac
      481 acaagtcaag aggaaaccct ccactcaacc acctccgaag gcaatctaag cccatcacaa
      541 gtctatacaa catccgagta cctatcacaa tctccatctt catcctacac aacaaaatga
//
LOCUS       KY327949                 600 bp    cRNA    linear   VRL 13-DEC-2016
DEFINITION  Human respiratory syncytial virus A isolate TH-CU/C3110/2012
            attachment glycoprotein gene, partial cds.
```

```
ACCESSION   KY327949
VERSION     KY327949
KEYWORDS    .
SOURCE      Human respiratory syncytial virus A
  ORGANISM  Human respiratory syncytial virus A
            Viruses; ssRNA viruses; ssRNA negative-strand viruses;
            Mononegavirales; Pneumoviridae; Orthopneumovirus.
REFERENCE   1  (bases 1 to 600)
  AUTHORS   Thongpan,I., Mauleekoonphairoj,J., Vichiwattana,P., Korkong,S.,
            Vongpunsawad,S. and Poovorawan,Y.
  TITLE     Molecular Characterization of Respiratory Syncytial Virus in
            Thailand, 2012-2015
  JOURNAL   Unpublished
REFERENCE   2  (bases 1 to 600)
  AUTHORS   Thongpan,I., Mauleekoonphairoj,J., Vichiwattana,P., Korkong,S.,
            Vongpunsawad,S. and Poovorawan,Y.
  TITLE     Direct Submission
  JOURNAL   Submitted (13-DEC-2016) Department of Pediatrics, Center of
            Excellence in Clinical Virology, Faculty of Medicine, Chulalongkorn
            University, Bangkok 10330, Thailand
COMMENT     ##Assembly-Data-START##
            Assembly Method       :: DNASTAR-Lasergene v. 6
            Sequencing Technology :: Sanger dideoxy sequencing
            ##Assembly-Data-END##
FEATURES             Location/Qualifiers
     source          1..600
                     /organism="Human respiratory syncytial virus A"
                     /mol_type="viral cRNA"
                     /isolate="TH-CU/C3110/2012"
                     /host="Homo sapiens"
                     /db_xref="taxon:208893"
                     /country="Thailand"
                     /collection_date="01-Aug-2012"
                     /note="group: A"
     CDS             <1..600
                     /note="G protein"
                     /codon_start=1
                     /product="attachment glycoprotein"
                     /protein_id="APY20290"
                     /translation="ISFSNLSGTTSQSTTILASTTPSAESTPQSTTVKIKNTTTTQIQ
                     PSKPTTKQRQNKPQNKPNNDFHFEVFNFVPCSICSNNPTCWAICKRIPNKKPGKKTTT
                     KPTKKPTIKTTKKDPKPQTTKPKEVLTTKPTEKPTIDTTKTNIRTTLLTSYTTGNQEH
                     TSQEETLHSTTSEGNLSPSQVYTTSEYLSQSPSSSYTTK"
ORIGIN
        1 atcagcttct ccaatctgtc cggaactaca tcacaatcca ccaccatact agcttcaaca
       61 acaccaagtg ctgagtcaac cccacaatcc acaacagtca agatcaaaaa cacaacaaca
      121 acccaaatac aacctagcaa acccaccaca aaacaacgcc aaaataaacc acaaaacaaa
      181 cccaacaatg attttcactt tgaagtgttc aactttgtac cctgcagcat atgcagcaac
      241 aatccaacct gctgggctat ctgcaagaga ataccaaaca aaaaacctgg aaagaaaact
      301 accaccaagc ccacaaagaa accaaccatc aagacaacca aaaaagatcc caaacctcaa
      361 accacaaaac caaaggaagt actcaccacc aagcccacag aaaagccaac catcgacacc
      421 accaaaacaa acatcagaac tacactgctc acctcctaca ctacaggaaa tcaagaacac
      481 acaagtcaag aggaaaccct ccactcaacc acctccgaag gcaatctaag cccatcacaa
      541 gtctatacaa catccgagta cctatcacaa tctccatctt catcctacac aacaaaatga
```

```
//
LOCUS       KY327950                 600 bp    cRNA    linear   VRL 13-DEC-2016
DEFINITION  Human respiratory syncytial virus A isolate TH-CU/C3143/2012
            attachment glycoprotein gene, partial cds.
ACCESSION   KY327950
VERSION     KY327950
KEYWORDS    .
SOURCE      Human respiratory syncytial virus A
  ORGANISM  Human respiratory syncytial virus A
            Viruses; ssRNA viruses; ssRNA negative-strand viruses;
            Mononegavirales; Pneumoviridae; Orthopneumovirus.
REFERENCE   1  (bases 1 to 600)
  AUTHORS   Thongpan,I., Mauleekoonphairoj,J., Vichiwattana,P., Korkong,S.,
            Vongpunsawad,S. and Poovorawan,Y.
  TITLE     Molecular Characterization of Respiratory Syncytial Virus in
            Thailand, 2012-2015
  JOURNAL   Unpublished
REFERENCE   2  (bases 1 to 600)
  AUTHORS   Thongpan,I., Mauleekoonphairoj,J., Vichiwattana,P., Korkong,S.,
            Vongpunsawad,S. and Poovorawan,Y.
  TITLE     Direct Submission
  JOURNAL   Submitted (13-DEC-2016) Department of Pediatrics, Center of
            Excellence in Clinical Virology, Faculty of Medicine, Chulalongkorn
            University, Bangkok 10330, Thailand
COMMENT     ##Assembly-Data-START##
            Assembly Method       :: DNASTAR-Lasergene v. 6
            Sequencing Technology :: Sanger dideoxy sequencing
            ##Assembly-Data-END##
FEATURES             Location/Qualifiers
     source          1..600
                     /organism="Human respiratory syncytial virus A"
                     /mol_type="viral cRNA"
                     /isolate="TH-CU/C3143/2012"
                     /host="Homo sapiens"
                     /db_xref="taxon:208893"
                     /country="Thailand"
                     /collection_date="01-Aug-2012"
                     /note="group: A"
     CDS             <1..600
                     /note="G protein"
                     /codon_start=1
                     /product="attachment glycoprotein"
                     /protein_id="APY20291"
                     /translation="ISLSNLSGTTSQSTTILASTTPSAESTPQSTTVKIKNTTTTQIQ
                     PSKPTTKQRQNKPQNKPNNDFHFEVFNFVPCSICSNNPTCWAICKRIPNKKPGKKTTT
                     KPTKKPTIKTTKKDPKPQTTKPKEVLTTKPTEKPTIDTTKTNIRTTLLTSYTTGNQEH
                     TSQEETLHSTTSEGNLSPSQVYTTSEYLSQSPSSSYTTK"
ORIGIN
        1 atcagcctct ccaatctgtc cggaactaca tcacaatcca ccaccatact agcttcaaca
       61 acaccaagtg ctgagtcaac cccacaatcc acaacagtca agatcaaaaa cacaacaaca
      121 acccaaatac aacctagcaa acccaccaca aaacaacgcc aaaataaacc acaaaacaaa
      181 cccaacaatg attttcactt tgaagtgttc aactttgtac cctgcagcat atgcagcaac
      241 aatccaacct gctgggctat ctgcaagaga ataccaaaca aaaaacctgg aaagaaaact
      301 accaccaagc ccacaaagaa accaaccatc aagacaacca aaaaagatcc caaacctcaa
```

```
     361 accacaaaac caaaggaagt actcaccacc aagcccacag aaaagccaac catcgacacc
     421 accaaaacaa acatcagaac tacactgctc acctcctaca ctacaggaaa tcaagaacac
     481 acaagtcaag aggaaaccct ccactcaacc acctccgaag gcaatctaag cccatcacaa
     541 gtctatacaa catccgagta cctatcacaa tctccatctt catcctacac aacaaaatga
//
LOCUS       KY327951                 600 bp    cRNA    linear   VRL 13-DEC-2016
DEFINITION  Human respiratory syncytial virus A isolate TH-CU/C3205/2012
            attachment glycoprotein gene, partial cds.
ACCESSION   KY327951
VERSION     KY327951
KEYWORDS    .
SOURCE      Human respiratory syncytial virus A
  ORGANISM  Human respiratory syncytial virus A
            Viruses; ssRNA viruses; ssRNA negative-strand viruses;
            Mononegavirales; Pneumoviridae; Orthopneumovirus.
REFERENCE   1  (bases 1 to 600)
  AUTHORS   Thongpan,I., Mauleekoonphairoj,J., Vichiwattana,P., Korkong,S.,
            Vongpunsawad,S. and Poovorawan,Y.
  TITLE     Molecular Characterization of Respiratory Syncytial Virus in
            Thailand, 2012-2015
  JOURNAL   Unpublished
REFERENCE   2  (bases 1 to 600)
  AUTHORS   Thongpan,I., Mauleekoonphairoj,J., Vichiwattana,P., Korkong,S.,
            Vongpunsawad,S. and Poovorawan,Y.
  TITLE     Direct Submission
  JOURNAL   Submitted (13-DEC-2016) Department of Pediatrics, Center of
            Excellence in Clinical Virology, Faculty of Medicine, Chulalongkorn
            University, Bangkok 10330, Thailand
COMMENT     ##Assembly-Data-START##
            Assembly Method       :: DNASTAR-Lasergene v. 6
            Sequencing Technology :: Sanger dideoxy sequencing
            ##Assembly-Data-END##
FEATURES             Location/Qualifiers
     source          1..600
                     /organism="Human respiratory syncytial virus A"
                     /mol_type="viral cRNA"
                     /isolate="TH-CU/C3205/2012"
                     /host="Homo sapiens"
                     /db_xref="taxon:208893"
                     /country="Thailand"
                     /collection_date="01-Sep-2012"
                     /note="group: A"
     CDS             <1..600
                     /note="G protein"
                     /codon_start=1
                     /product="attachment glycoprotein"
                     /protein_id="APY20292"
                     /translation="ISFSNLSGTTSQSTTILASTTPSAESTPQSTTVKIKNTTTTQIQ
                     PSKPTTKQHQNKPQNKPNNDFHFEVFNFVPCSICSNNPTCWAICKRIPNKKPGKKTTT
                     KPTKKPTIKTTKKDPKPQTTKPKEVLTTKPTEKPTIDTTKTNIRTTLLTSYTTGNQEH
                     TSQEETLHSTTSEGNLSPSQVYTTSEYLSQSPSSSYTTK"
ORIGIN
        1 atcagcttct ctaatctgtc cggaactaca tcacaatcca ccaccatact agcttcaaca
       61 acaccaagtg ctgagtcaac cccacaatcc acaacagtca agatcaaaaa cacaacaaca
```

```
     121 acccaaatac aacctagcaa acccaccaca aaacaacacc aaaataaacc acaaaacaaa
     181 cccaacaatg attttcactt tgaagtgttc aactttgtac cctgcagcat atgcagcaac
     241 aatccaacct gctgggctat ctgcaagaga ataccaaaca aaaaacctgg aaagaaaact
     301 accaccaagc ccacaaagaa accaaccatc aagacaacca aaaaagatcc caaacctcaa
     361 accacaaaac caaaggaagt actcaccacc aagcccacag aaaagccaac catcgacacc
     421 accaaaacaa acatcagaac tacactgctc acctcctaca ctacaggaaa tcaagaacac
     481 acaagtcaag aggaaaccct ccactcaacc acctccgaag gcaatctaag cccatcacaa
     541 gtctatacaa catccgagta cctatcacaa tctccatctt catcctacac aacaaaatga
//
LOCUS       KY327952                 600 bp    cRNA    linear   VRL 13-DEC-2016
DEFINITION  Human respiratory syncytial virus A isolate TH-CU/C3086/2012
            attachment glycoprotein gene, partial cds.
ACCESSION   KY327952
VERSION     KY327952
KEYWORDS    .
SOURCE      Human respiratory syncytial virus A
  ORGANISM  Human respiratory syncytial virus A
            Viruses; ssRNA viruses; ssRNA negative-strand viruses;
            Mononegavirales; Pneumoviridae; Orthopneumovirus.
REFERENCE   1  (bases 1 to 600)
  AUTHORS   Thongpan,I., Mauleekoonphairoj,J., Vichiwattana,P., Korkong,S.,
            Vongpunsawad,S. and Poovorawan,Y.
  TITLE     Molecular Characterization of Respiratory Syncytial Virus in
            Thailand, 2012-2015
  JOURNAL   Unpublished
REFERENCE   2  (bases 1 to 600)
  AUTHORS   Thongpan,I., Mauleekoonphairoj,J., Vichiwattana,P., Korkong,S.,
            Vongpunsawad,S. and Poovorawan,Y.
  TITLE     Direct Submission
  JOURNAL   Submitted (13-DEC-2016) Department of Pediatrics, Center of
            Excellence in Clinical Virology, Faculty of Medicine, Chulalongkorn
            University, Bangkok 10330, Thailand
COMMENT     ##Assembly-Data-START##
            Assembly Method       :: DNASTAR-Lasergene v. 6
            Sequencing Technology :: Sanger dideoxy sequencing
            ##Assembly-Data-END##
FEATURES             Location/Qualifiers
     source          1..600
                     /organism="Human respiratory syncytial virus A"
                     /mol_type="viral cRNA"
                     /isolate="TH-CU/C3086/2012"
                     /host="Homo sapiens"
                     /db_xref="taxon:208893"
                     /country="Thailand"
                     /collection_date="01-Aug-2012"
                     /note="group: A"
     CDS             <1..600
                     /note="G protein"
                     /codon_start=1
                     /product="attachment glycoprotein"
                     /protein_id="APY20293"
                     /translation="ISFSNLSGTTSQSTTILASTTPSAESTPQSTTVKIKNTTTTQIQ
                     PSKPTTKQRQNKPQNKPNNDFHFEVFNFVPCSICSNNPTCWAICKRIPNKKPGKKTTT
                     KPTKKPTIKTTKKDPKPQTTKPKEVLTTKPTEKPTIDTTKTNIRTTLLTSYTTGNQEH
```

```
                    TSQEETLHSTTSEGNLSPSQVYTTSEYLSQSPSSSYTTK"
ORIGIN
        1 atcagcttct ccaatctgtc cggaactaca tcacaatcca ccaccatact agcttcaaca
       61 acaccaagtg ctgagtcaac cccacaatcc acaacagtca agatcaaaaa cacaacaaca
      121 acccaaatac aacctagcaa acccaccaca aaacaacgcc aaaataaacc acaaaacaaa
      181 cccaacaatg attttcactt tgaagtgttc aactttgtac cctgcagcat atgcagcaac
      241 aatccaacct gctgggctat ctgcaagaga ataccaaaca aaaaacctgg aaagaaaact
      301 accaccaagc ccacaaagaa accaaccatc aagacaacca aaaaagatcc caaacctcaa
      361 accacaaaac caaaggaagt actcaccacc aagcccacag aaaagccaac catcgacacc
      421 accaaaacaa acatcagaac tacactgctc acctcctaca ctacaggaaa tcaagaacac
      481 acaagtcaag aggaaaccct ccactcaacc acctccgaag gcaatctaag cccatcacaa
      541 gtctatacaa catccgagta cctatcacaa tctccatctt catcctacac aacaaaatga
//
LOCUS       KY327953                 600 bp    cRNA    linear   VRL 13-DEC-2016
DEFINITION  Human respiratory syncytial virus A isolate TH-CU/C3066/2012
            attachment glycoprotein gene, partial cds.
ACCESSION   KY327953
VERSION     KY327953
KEYWORDS    .
SOURCE      Human respiratory syncytial virus A
  ORGANISM  Human respiratory syncytial virus A
            Viruses; ssRNA viruses; ssRNA negative-strand viruses;
            Mononegavirales; Pneumoviridae; Orthopneumovirus.
REFERENCE   1  (bases 1 to 600)
  AUTHORS   Thongpan,I., Mauleekoonphairoj,J., Vichiwattana,P., Korkong,S.,
            Vongpunsawad,S. and Poovorawan,Y.
  TITLE     Molecular Characterization of Respiratory Syncytial Virus in
            Thailand, 2012-2015
  JOURNAL   Unpublished
REFERENCE   2  (bases 1 to 600)
  AUTHORS   Thongpan,I., Mauleekoonphairoj,J., Vichiwattana,P., Korkong,S.,
            Vongpunsawad,S. and Poovorawan,Y.
  TITLE     Direct Submission
  JOURNAL   Submitted (13-DEC-2016) Department of Pediatrics, Center of
            Excellence in Clinical Virology, Faculty of Medicine, Chulalongkorn
            University, Bangkok 10330, Thailand
COMMENT     ##Assembly-Data-START##
            Assembly Method       :: DNASTAR-Lasergene v. 6
            Sequencing Technology :: Sanger dideoxy sequencing
            ##Assembly-Data-END##
FEATURES             Location/Qualifiers
     source          1..600
                     /organism="Human respiratory syncytial virus A"
                     /mol_type="viral cRNA"
                     /isolate="TH-CU/C3066/2012"
                     /host="Homo sapiens"
                     /db_xref="taxon:208893"
                     /country="Thailand"
                     /collection_date="01-Aug-2012"
                     /note="group: A"
     CDS             <1..600
                     /note="G protein"
                     /codon_start=1
                     /product="attachment glycoprotein"
```

/protein_id="APY20294"
/translation="ISFSNLSGTTSQSTTILASTTPSAESTPQSTTVKIKNTTTTQIQ
PSKPTTKQRQNKPQNKPNNDFHFEVFNFVPCSICSNNPTCWAICKRIPNKKPGKKTTT
KPTKKPTIKTTKKDPKPQTTKPKEVLTTKPTEKPTIDTTKTNIRTTLLTSYTTGNQEH
TSQEETLHSTTSEGNLSPSQVYTTSEYLSQSPSSSYTTK"
ORIGIN
1 atcagcttct ccaatctgtc cggaactaca tcacaatcca ccaccatact agcttcaaca
61 acaccaagtg ctgagtcaac ccccacaatcc acaacagtca agatcaaaaa cacaacaaca
121 acccaaatac aacctagcaa acccaccaca aaacaacgcc aaaataaacc acaaaacaaa
181 cccaacaatg attttcactt tgaagtgttc aactttgtac cctgcagcat atgcagcaac
241 aatccaacct gctgggctat ctgcaagaga ataccaaaca aaaaacctgg aaagaaaact
301 accaccaagc ccacaaagaa accaaccatc aagacaacca aaaaagatcc caaacctcaa
361 accacaaaac caaaggaagt actcaccacc aagcccacag aaaagccaac catcgacacc
421 accaaaacaa acatcagaac tacactgctc acctcctaca ctacaggaaa tcaagaacac
481 acaagtcaag aggaaaccct ccactcaacc acctccgaag gcaatctaag cccatcacaa
541 gtctatacaa catccgagta cctatcacaa tctccatctt catcctacac aacaaaatga
//
LOCUS KY327954 600 bp cRNA linear VRL 13-DEC-2016
DEFINITION Human respiratory syncytial virus A isolate TH-CU/C3037/2012
attachment glycoprotein gene, partial cds.
ACCESSION KY327954
VERSION KY327954
KEYWORDS .
SOURCE Human respiratory syncytial virus A
ORGANISM Human respiratory syncytial virus A
Viruses; ssRNA viruses; ssRNA negative-strand viruses;
Mononegavirales; Pneumoviridae; Orthopneumovirus.
REFERENCE 1 (bases 1 to 600)
AUTHORS Thongpan,I., Mauleekoonphairoj,J., Vichiwattana,P., Korkong,S.,
Vongpunsawad,S. and Poovorawan,Y.
TITLE Molecular Characterization of Respiratory Syncytial Virus in
Thailand, 2012-2015
JOURNAL Unpublished
REFERENCE 2 (bases 1 to 600)
AUTHORS Thongpan,I., Mauleekoonphairoj,J., Vichiwattana,P., Korkong,S.,
Vongpunsawad,S. and Poovorawan,Y.
TITLE Direct Submission
JOURNAL Submitted (13-DEC-2016) Department of Pediatrics, Center of
Excellence in Clinical Virology, Faculty of Medicine, Chulalongkorn
University, Bangkok 10330, Thailand
COMMENT ##Assembly-Data-START##
Assembly Method :: DNASTAR-Lasergene v. 6
Sequencing Technology :: Sanger dideoxy sequencing
##Assembly-Data-END##
FEATURES Location/Qualifiers
source 1..600
/organism="Human respiratory syncytial virus A"
/mol_type="viral cRNA"
/isolate="TH-CU/C3037/2012"
/host="Homo sapiens"
/db_xref="taxon:208893"
/country="Thailand"
/collection_date="01-Jul-2012"
/note="group: A"

```
     CDS             <1..600
                     /note="G protein"
                     /codon_start=1
                     /product="attachment glycoprotein"
                     /protein_id="APY20295"
                     /translation="ISFSNLSGTTSQSTTILASTTPSAESTPQSTTVKIKNTTTTQIQ
                     PSKPTTKQRQNKPQNKPNNDFHFEVFNFVPCSICSNNPTCWAICKRIPNKKPGKKTTT
                     KPTKKPTIKTTKKDPKPQTTKPKEVLTTKPTEKPTIDTTKTNIRTTLLTSYTTGNQEH
                     TSQEETLHSTTSEGNLSPSQVYTTSEYLSQSPSSSYTTK"
ORIGIN
        1 atcagcttct ccaatctgtc cggaactaca tcacaatcca ccaccatact agcttcaaca
       61 acaccaagtg ctgagtcaac ccccacaatcc acaacagtca agatcaaaaa cacaacaaca
      121 acccaaatac aacctagcaa acccaccaca aaacaacgcc aaaataaacc acaaaacaaa
      181 cccaacaatg attttcactt tgaagtgttc aactttgtac cctgcagcat atgcagcaac
      241 aatccaacct gctgggctat ctgcaagaga ataccaaaca aaaaacctgg aaagaaaact
      301 accaccaagc ccacaaagaa accaaccatc aagacaacca aaaaagatcc caaacctcaa
      361 accacaaaac caaaggaagt actcaccacc aagcccacag aaaagccaac catcgacacc
      421 accaaaacaa acatcagaac tacactgctc acctcctaca ctacaggaaa tcaagaacac
      481 acaagtcaag aggaaaccct ccactcaacc acctccgaag gcaatctaag cccatcacaa
      541 gtctatacaa catccgagta cctatcacaa tctccatctt catcctacac aacaaaatga
//
LOCUS       KY327955                 600 bp    cRNA    linear   VRL 13-DEC-2016
DEFINITION  Human respiratory syncytial virus A isolate TH-CU429/2012
            attachment glycoprotein gene, partial cds.
ACCESSION   KY327955
VERSION     KY327955
KEYWORDS    .
SOURCE      Human respiratory syncytial virus A
  ORGANISM  Human respiratory syncytial virus A
            Viruses; ssRNA viruses; ssRNA negative-strand viruses;
            Mononegavirales; Pneumoviridae; Orthopneumovirus.
REFERENCE   1  (bases 1 to 600)
  AUTHORS   Thongpan,I., Mauleekoonphairoj,J., Vichiwattana,P., Korkong,S.,
            Vongpunsawad,S. and Poovorawan,Y.
  TITLE     Molecular Characterization of Respiratory Syncytial Virus in
            Thailand, 2012-2015
  JOURNAL   Unpublished
REFERENCE   2  (bases 1 to 600)
  AUTHORS   Thongpan,I., Mauleekoonphairoj,J., Vichiwattana,P., Korkong,S.,
            Vongpunsawad,S. and Poovorawan,Y.
  TITLE     Direct Submission
  JOURNAL   Submitted (13-DEC-2016) Department of Pediatrics, Center of
            Excellence in Clinical Virology, Faculty of Medicine, Chulalongkorn
            University, Bangkok 10330, Thailand
COMMENT     ##Assembly-Data-START##
            Assembly Method       :: DNASTAR-Lasergene v. 6
            Sequencing Technology :: Sanger dideoxy sequencing
            ##Assembly-Data-END##
FEATURES             Location/Qualifiers
     source          1..600
                     /organism="Human respiratory syncytial virus A"
                     /mol_type="viral cRNA"
                     /isolate="TH-CU429/2012"
                     /host="Homo sapiens"
```

```
                     /db_xref="taxon:208893"
                     /country="Thailand"
                     /collection_date="01-Aug-2012"
                     /note="group: A"
     CDS             <1..600
                     /note="G protein"
                     /codon_start=1
                     /product="attachment glycoprotein"
                     /protein_id="APY20296"
                     /translation="ISFSNLSGTTSQSTTILASTTPGAESTPQSTTVKFKNTTTTQIL
                     PSKPTTKQRQNKPQNKPNNDFHFEVFNFVPCSICSNNPTCWAICKRIPNKKPGKKTTT
                     KPTKKPTIKTTKKDPKPQTTKPKEVLTTKPTEKPTIDTTKTNIRTTLLTSYTTGNPEH
                     TSQEETIHSTTSEGNLSPSQVYTTSEYLSQSPSSSYTTK"
ORIGIN
        1 atcagcttct ccaatctgtc cggaactaca tcacaatcca ccaccatact agcttcaaca
       61 acaccaggtg ctgagtcaac cccacaatcc acaacagtca agttcaaaaa cacaacaaca
      121 acccaaatac tacctagcaa acccaccaca aaacaacgcc aaaataaacc acaaaacaaa
      181 cccaacaatg attttcactt tgaagtgttc aactttgtac cctgcagcat atgcagcaac
      241 aatccaacct gctgggccat ctgcaagaga ataccaaaca aaaaacctgg aaagaaaacc
      301 accaccaagc ccacaaaaaa accaaccatc aagacaacca aaaaagatcc caaacctcaa
      361 accacaaaac caaaggaagt actcaccacc aagcccacag aaaagccaac catcgacacc
      421 accaaaacaa acatcagaac tacactgctc acctcctaca ccacaggaaa tccagaacac
      481 acaagtcaag aggaaaccat ccactcaacc acctccgaag gcaatctaag cccatcacaa
      541 gtctatacaa catccgagta cctatcacaa tctccatctt catcctacac aacaaaatga
//
LOCUS       KY327956                 600 bp    cRNA    linear   VRL 13-DEC-2016
DEFINITION  Human respiratory syncytial virus A isolate TH-CU428/2012
            attachment glycoprotein gene, partial cds.
ACCESSION   KY327956
VERSION     KY327956
KEYWORDS    .
SOURCE      Human respiratory syncytial virus A
  ORGANISM  Human respiratory syncytial virus A
            Viruses; ssRNA viruses; ssRNA negative-strand viruses;
            Mononegavirales; Pneumoviridae; Orthopneumovirus.
REFERENCE   1  (bases 1 to 600)
  AUTHORS   Thongpan,I., Mauleekoonphairoj,J., Vichiwattana,P., Korkong,S.,
            Vongpunsawad,S. and Poovorawan,Y.
  TITLE     Molecular Characterization of Respiratory Syncytial Virus in
            Thailand, 2012-2015
  JOURNAL   Unpublished
REFERENCE   2  (bases 1 to 600)
  AUTHORS   Thongpan,I., Mauleekoonphairoj,J., Vichiwattana,P., Korkong,S.,
            Vongpunsawad,S. and Poovorawan,Y.
  TITLE     Direct Submission
  JOURNAL   Submitted (13-DEC-2016) Department of Pediatrics, Center of
            Excellence in Clinical Virology, Faculty of Medicine, Chulalongkorn
            University, Bangkok 10330, Thailand
COMMENT     ##Assembly-Data-START##
            Assembly Method       :: DNASTAR-Lasergene v. 6
            Sequencing Technology :: Sanger dideoxy sequencing
            ##Assembly-Data-END##
FEATURES             Location/Qualifiers
     source          1..600
```

/organism="Human respiratory syncytial virus A"
/mol_type="viral cRNA"
/isolate="TH-CU428/2012"
/host="Homo sapiens"
/db_xref="taxon:208893"
/country="Thailand"
/collection_date="01-Aug-2012"
/note="group: A"
CDS <1..600
/note="G protein"
/codon_start=1
/product="attachment glycoprotein"
/protein_id="APY20297"
/translation="ISFSNLSGTTSQSTTILASTTPGAESTPQSTTVKFKNTTTTQIL
PSKPTTKQRQNKPQNKPNNDFHFEVFNFVPCSICSNNPTCWAICKRIPNKKPGKKTTT
KPTKKPTIKTTKKDPKPQTTKPKEVLTTKPTEKPTIDTTKTNIRTTLLTSYTTGNPEH
TSQEETIHSTTSEGNLSPSQVYTTSEYLSQSPSSSYTTK"

ORIGIN
1 atcagcttct ccaatctgtc cggaactaca tcacaatcca ccaccatact agcttcaaca
61 acaccaggtg ctgagtcaac cccacaatcc acaacagtca agttcaaaaa cacaacaaca
121 acccaaatac tacctagcaa acccaccaca aaacaacgcc aaaataaacc acaaaacaaa
181 cccaacaatg attttcactt tgaagtgttc aactttgtac cctgcagcat atgcagcaac
241 aatccaacct gctgggccat ctgcaagaga ataccaaaca aaaaacctgg aaagaaaacc
301 accaccaagc ccacaaaaaa accaaccatc aagacaacca aaaaagatcc caaacctcaa
361 accacaaaac caaaggaagt actcaccacc aagcccacag aaaagccaac catcgacacc
421 accaaaacaa acatcagaac tacactgctc acctcctaca ccacaggaaa tccagaacac
481 acaagtcaag aggaaaccat ccactcaacc acctccgaag gcaatctaag cccatcacaa
541 gtctatacaa catccgagta cctatcacaa tctccatctt catcctacac aacaaaatga
//

LOCUS KY327957 600 bp cRNA linear VRL 13-DEC-2016
DEFINITION Human respiratory syncytial virus A isolate TH-CU416/2012
attachment glycoprotein gene, partial cds.
ACCESSION KY327957
VERSION KY327957
KEYWORDS .
SOURCE Human respiratory syncytial virus A
ORGANISM Human respiratory syncytial virus A
Viruses; ssRNA viruses; ssRNA negative-strand viruses;
Mononegavirales; Pneumoviridae; Orthopneumovirus.
REFERENCE 1 (bases 1 to 600)
AUTHORS Thongpan,I., Mauleekoonphairoj,J., Vichiwattana,P., Korkong,S.,
Vongpunsawad,S. and Poovorawan,Y.
TITLE Molecular Characterization of Respiratory Syncytial Virus in
Thailand, 2012-2015
JOURNAL Unpublished
REFERENCE 2 (bases 1 to 600)
AUTHORS Thongpan,I., Mauleekoonphairoj,J., Vichiwattana,P., Korkong,S.,
Vongpunsawad,S. and Poovorawan,Y.
TITLE Direct Submission
JOURNAL Submitted (13-DEC-2016) Department of Pediatrics, Center of
Excellence in Clinical Virology, Faculty of Medicine, Chulalongkorn
University, Bangkok 10330, Thailand
COMMENT ##Assembly-Data-START##
Assembly Method :: DNASTAR-Lasergene v. 6

Sequencing Technology :: Sanger dideoxy sequencing
##Assembly-Data-END##
FEATURES   Location/Qualifiers
     source   1..600
                     /organism="Human respiratory syncytial virus A"
                     /mol_type="viral cRNA"
                     /isolate="TH-CU416/2012"
                     /host="Homo sapiens"
                     /db_xref="taxon:208893"
                     /country="Thailand"
                     /collection_date="01-Aug-2012"
                     /note="group: A"
     CDS   <1..600
                     /note="G protein"
                     /codon_start=1
                     /product="attachment glycoprotein"
                     /protein_id="APY20298"
                     /translation="ISFSNLSGTTSQSTTILASTTPGAESTPQSTTVKFKNTTTTQIL
                     PSKPTTKQRQNKPQNKPNNDFHFEVFNFVPCSICSNNPTCWAICKRIPNKKPGKKTTT
                     KPTKKPTIKTTKKDPKPQTTKPKEVLTTKPTEKPTIDTTKTNIRTTLLTSYTTGNPEH
                     TSQEETIHSTTSEGNLSPSQVYTTSEYLSQPSSSYTTK"
ORIGIN
        1 atcagcttct ccaatctgtc cggaactaca tcacaatcca ccaccatact agcttcaaca
       61 acaccaggtg ctgagtcaac cccacaatcc acaacagtca agttcaaaaa cacaacaaca
      121 acccaaatac tacctagcaa acccaccaca aaacaacgcc aaaataaacc acaaaacaaa
      181 cccaacaatg attttcactt tgaagtgttc aactttgtac cctgcagcat atgcagcaac
      241 aatccaacct gctgggccat ctgcaagaga ataccaaaca aaaaacctgg aaagaaaacc
      301 accaccaagc ccacaaaaaa accaaccatc aagacaacca aaaaagatcc caaacctcaa
      361 accacaaaac caaaggaagt actcaccacc aagcccacag aaaagccaac catcgacacc
      421 accaaaacaa acatcagaac tacactgctc acctcctaca ccacaggaaa tccagaacac
      481 acaagtcaag aggaaaccat ccactcaacc acctccgaag gcaatctaag cccatcacaa
      541 gtctatacaa catccgagta cctatcacaa tctccatctt catcctacac aacaaaatga
//
LOCUS   KY327958   600 bp   cRNA   linear   VRL 13-DEC-2016
DEFINITION Human respiratory syncytial virus A isolate TH-CU441/2012
            attachment glycoprotein gene, partial cds.
ACCESSION   KY327958
VERSION   KY327958
KEYWORDS   .
SOURCE   Human respiratory syncytial virus A
  ORGANISM Human respiratory syncytial virus A
            Viruses; ssRNA viruses; ssRNA negative-strand viruses;
            Mononegavirales; Pneumoviridae; Orthopneumovirus.
REFERENCE   1 (bases 1 to 600)
  AUTHORS   Thongpan,I., Mauleekoonphairoj,J., Vichiwattana,P., Korkong,S.,
            Vongpunsawad,S. and Poovorawan,Y.
  TITLE   Molecular Characterization of Respiratory Syncytial Virus in
            Thailand, 2012-2015
  JOURNAL   Unpublished
REFERENCE   2 (bases 1 to 600)
  AUTHORS   Thongpan,I., Mauleekoonphairoj,J., Vichiwattana,P., Korkong,S.,
            Vongpunsawad,S. and Poovorawan,Y.
  TITLE   Direct Submission
  JOURNAL   Submitted (13-DEC-2016) Department of Pediatrics, Center of

Excellence in Clinical Virology, Faculty of Medicine, Chulalongkorn
University, Bangkok 10330, Thailand
COMMENT ##Assembly-Data-START##
Assembly Method :: DNASTAR-Lasergene v. 6
Sequencing Technology :: Sanger dideoxy sequencing
##Assembly-Data-END##
FEATURES Location/Qualifiers
source 1..600
/organism="Human respiratory syncytial virus A"
/mol_type="viral cRNA"
/isolate="TH-CU441/2012"
/host="Homo sapiens"
/db_xref="taxon:208893"
/country="Thailand"
/collection_date="01-Sep-2012"
/note="group: A"
CDS <1..600
/note="G protein"
/codon_start=1
/product="attachment glycoprotein"
/protein_id="APY20299"
/translation="ISFSNLSGTTSQFTTILASTTPSAESTPQSTTVKIKNTTTTQIQ
PSKPTTKQRQNKPQNKPNNDFHFEAFNFVPCSTCSNNPTCWAICKRIPNKKPGKKTTT
KPTKKPTIKTTKKDPKPQTTKPKEVLTTKPTEKPTIDTTKTNIRTTLLTSYTTGNPEH
TSQEETLHSTTSEGNLSPSQVHTTSEYLSQSPSSSYTTK"
ORIGIN
1 atcagcttct ccaatctgtc cggaactaca tcacaattca ccaccatact agcttcaaca
61 acaccaagtg ctgagtcaac cccacaatcc acaacagtca agatcaaaaa cacaacaaca
121 acccaaatac aacctagcaa acccaccaca aaacaacgcc aaaataaacc acaaaacaaa
181 cccaacaatg attttcactt tgaagcgttc aactttgtac cctgcagcac atgcagcaac
241 aatccaacct gctgggccat ctgcaagaga ataccaaaca aaaaacctgg aaagaaaacc
301 accaccaagc ccacaaaaaa accaaccatc aagacaacca aaaaagatcc caaacctcaa
361 accacaaaac caaaggaagt actcaccacc aagcccacag aaaagccaac catcgacacc
421 accaaaacaa acatcagaac tacactgctc acctcctaca ccacaggaaa tccagaacac
481 acaagtcaag aggaaaccct ccactcaacc acctccgaag gcaatctaag cccatcacaa
541 gtccacacaa catccgagta cctatcacaa tctccatctt catcctacac aacaaaatga
//
LOCUS KY327959 600 bp cRNA linear VRL 13-DEC-2016
DEFINITION Human respiratory syncytial virus A isolate TH-CU/C3281/2012
attachment glycoprotein gene, partial cds.
ACCESSION KY327959
VERSION KY327959
KEYWORDS .
SOURCE Human respiratory syncytial virus A
ORGANISM Human respiratory syncytial virus A
Viruses; ssRNA viruses; ssRNA negative-strand viruses;
Mononegavirales; Pneumoviridae; Orthopneumovirus.
REFERENCE 1 (bases 1 to 600)
AUTHORS Thongpan,I., Mauleekoonphairoj,J., Vichiwattana,P., Korkong,S.,
Vongpunsawad,S. and Poovorawan,Y.
TITLE Molecular Characterization of Respiratory Syncytial Virus in
Thailand, 2012-2015
JOURNAL Unpublished
REFERENCE 2 (bases 1 to 600)

```
  AUTHORS   Thongpan,I., Mauleekoonphairoj,J., Vichiwattana,P., Korkong,S.,
            Vongpunsawad,S. and Poovorawan,Y.
  TITLE     Direct Submission
  JOURNAL   Submitted (13-DEC-2016) Department of Pediatrics, Center of
            Excellence in Clinical Virology, Faculty of Medicine, Chulalongkorn
            University, Bangkok 10330, Thailand
COMMENT     ##Assembly-Data-START##
            Assembly Method       :: DNASTAR-Lasergene v. 6
            Sequencing Technology :: Sanger dideoxy sequencing
            ##Assembly-Data-END##
FEATURES             Location/Qualifiers
     source          1..600
                     /organism="Human respiratory syncytial virus A"
                     /mol_type="viral cRNA"
                     /isolate="TH-CU/C3281/2012"
                     /host="Homo sapiens"
                     /db_xref="taxon:208893"
                     /country="Thailand"
                     /collection_date="01-Sep-2012"
                     /note="group: A"
     CDS             <1..600
                     /note="G protein"
                     /codon_start=1
                     /product="attachment glycoprotein"
                     /protein_id="APY20300"
                     /translation="ISFSNLSGTTSQSTTILASTTPSAESTPQSTTVKIKNTTTTQIQ
                     PSKPTTKQRQNKPQNKPNNDFHFEVFNFVPCSICSNNPTCWAICKRIPNKKPGKKTTT
                     KPTKKPTIKTTKKDPKPQTTKPKEVLTTKPTEKPTIDTTKTNIRTTLLTSYTTGNPEH
                     TSQEETLHSTTSEGNLSPSQVYTTSEYLSQSPSSSYTTK"
ORIGIN
        1 atcagcttct ccaatctgtc cggaactaca tcacaatcca ccaccatact agcttcaaca
       61 acaccaagtg ctgagtcaac cccacaatcc acaacagtca agatcaaaaa cacaacaaca
      121 acccaaatac aaccaagcaa acccaccaca aaacaacgcc aaaataaacc acaaaacaaa
      181 cccaacaatg attttcactt tgaagtgttc aactttgtac cctgcagcat atgcagcaac
      241 aatccaacct gctgggccat ctgcaagaga ataccaaaca aaaaacctgg aaagaaaacc
      301 accaccaagc ccacaaaaaa accaaccatc aagacaacca aaaaagatcc caaacctcaa
      361 accacaaaac caaaggaagt actcaccacc aagcccacag aaaagccaac catcgacacc
      421 accaaaacaa acatcagaac tacactgctc acctcctaca ccacaggaaa tccagaacac
      481 acaagtcaag aggaaaccct ccactcaacc acctccgaag gcaatctaag cccatcacaa
      541 gtctatacaa catccgagta cctatcacaa tctccatctt catcctacac aacaaaatga
//
LOCUS       KY327960                 600 bp    cRNA    linear   VRL 13-DEC-2016
DEFINITION  Human respiratory syncytial virus A isolate TH-CU412/2012
            attachment glycoprotein gene, partial cds.
ACCESSION   KY327960
VERSION     KY327960
KEYWORDS    .
SOURCE      Human respiratory syncytial virus A
  ORGANISM  Human respiratory syncytial virus A
            Viruses; ssRNA viruses; ssRNA negative-strand viruses;
            Mononegavirales; Pneumoviridae; Orthopneumovirus.
REFERENCE   1  (bases 1 to 600)
  AUTHORS   Thongpan,I., Mauleekoonphairoj,J., Vichiwattana,P., Korkong,S.,
            Vongpunsawad,S. and Poovorawan,Y.
```

```
  TITLE     Molecular Characterization of Respiratory Syncytial Virus in
            Thailand, 2012-2015
  JOURNAL   Unpublished
REFERENCE   2  (bases 1 to 600)
  AUTHORS   Thongpan,I., Mauleekoonphairoj,J., Vichiwattana,P., Korkong,S.,
            Vongpunsawad,S. and Poovorawan,Y.
  TITLE     Direct Submission
  JOURNAL   Submitted (13-DEC-2016) Department of Pediatrics, Center of
            Excellence in Clinical Virology, Faculty of Medicine, Chulalongkorn
            University, Bangkok 10330, Thailand
COMMENT     ##Assembly-Data-START##
            Assembly Method       :: DNASTAR-Lasergene v. 6
            Sequencing Technology :: Sanger dideoxy sequencing
            ##Assembly-Data-END##
FEATURES             Location/Qualifiers
     source          1..600
                     /organism="Human respiratory syncytial virus A"
                     /mol_type="viral cRNA"
                     /isolate="TH-CU412/2012"
                     /host="Homo sapiens"
                     /db_xref="taxon:208893"
                     /country="Thailand"
                     /collection_date="01-Aug-2012"
                     /note="group: A"
     CDS             <1..600
                     /note="G protein"
                     /codon_start=1
                     /product="attachment glycoprotein"
                     /protein_id="APY20301"
                     /translation="ISFSNLSGTTSQSTTILASTTPSAESTPQSTTVKFKNTTTTQIL
                     PSKPTTKQRQNKPQNKPNNDFHFEVFNFVPCSICSNNPTCWAICKRIPNKKPGKKTTT
                     KPTKKPTIKTTKKDPKPQTTKPKEVLTTKPTEKPTIDTTKTNIRTTLLTSYTTGNPEH
                     TSQEETLHSTTSEGNLSPSQVYTTSEYLSQSPSSSYTTK"
ORIGIN
        1 atcagcttct ccaatctgtc cggaactaca tcacaatcca ccaccatact agcttcaaca
       61 acaccaagtg ctgagtcaac cccacaatcc acaacagtca agttcaaaaa cacaacaaca
      121 acccaaatac tacctagcaa acccaccaca aaacaacgcc aaaataaacc acaaaacaaa
      181 cccaacaatg attttcactt tgaagtgttc aactttgtac cctgcagcat atgcagcaac
      241 aatccaacct gctgggccat ctgcaagaga ataccaaaca aaaaacctgg aaagaaaacc
      301 accaccaagc ccacaaaaaa accaaccatc aagacaacca aaaaagatcc caaacctcaa
      361 accacaaaac caaaggaagt actcaccacc aagcccacag aaaagccaac catcgacacc
      421 accaaaacaa acatcagaac tacactgctc acctcctaca ccacaggaaa tccagaacac
      481 acaagtcaag aggaaaccct ccactcaacc acctccgaag gcaatctaag cccatcacaa
      541 gtctatacaa catccgagta cctatcacaa tctccatctt catcctacac aacaaaatga
//
LOCUS       KY327961                 600 bp    cRNA    linear   VRL 13-DEC-2016
DEFINITION  Human respiratory syncytial virus A isolate TH-CU492/2012
            attachment glycoprotein gene, partial cds.
ACCESSION   KY327961
VERSION     KY327961
KEYWORDS    .
SOURCE      Human respiratory syncytial virus A
  ORGANISM  Human respiratory syncytial virus A
            Viruses; ssRNA viruses; ssRNA negative-strand viruses;
```

Mononegavirales; Pneumoviridae; Orthopneumovirus.
REFERENCE 1 (bases 1 to 600)
AUTHORS Thongpan,I., Mauleekoonphairoj,J., Vichiwattana,P., Korkong,S.,
Vongpunsawad,S. and Poovorawan,Y.
TITLE Molecular Characterization of Respiratory Syncytial Virus in
Thailand, 2012-2015
JOURNAL Unpublished
REFERENCE 2 (bases 1 to 600)
AUTHORS Thongpan,I., Mauleekoonphairoj,J., Vichiwattana,P., Korkong,S.,
Vongpunsawad,S. and Poovorawan,Y.
TITLE Direct Submission
JOURNAL Submitted (13-DEC-2016) Department of Pediatrics, Center of
Excellence in Clinical Virology, Faculty of Medicine, Chulalongkorn
University, Bangkok 10330, Thailand
COMMENT ##Assembly-Data-START##
Assembly Method :: DNASTAR-Lasergene v. 6
Sequencing Technology :: Sanger dideoxy sequencing
##Assembly-Data-END##
FEATURES Location/Qualifiers
source 1..600
/organism="Human respiratory syncytial virus A"
/mol_type="viral cRNA"
/isolate="TH-CU492/2012"
/host="Homo sapiens"
/db_xref="taxon:208893"
/country="Thailand"
/collection_date="01-Nov-2012"
/note="group: A"
CDS <1..600
/note="G protein"
/codon_start=1
/product="attachment glycoprotein"
/protein_id="APY20302"
/translation="ISFSNLSGTTSQSTTILASTTPSAESTPQSTTVKFKNTTTTQIL
PSKPTTKQRQNKPQNKPNNDFHFEVFNFVPCSICSNNPTCWAICKRIPNKKPGKKTTT
KPTKKPTIKTTKKDPKPQTTKPKEVLTTKPTEKPTIDTTKTNIRTTLLTSYTTGNPEH
TSQEETLHSTTSEGNLSPSQVYTTSEYLSQSPSSSYTTK"
ORIGIN
1 atcagcttct ccaatctgtc cggaactaca tcacaatcca ccaccatact agcttcaaca
61 acaccaagtg ctgagtcaac cccacaatcc acaacagtca agttcaaaaa cacaacaaca
121 acccaaatac tacctagcaa acccaccaca aaacaacgcc aaaataaacc acaaaacaaa
181 cccaacaatg attttcactt tgaagtgttc aactttgtac cctgcagcat atgcagcaac
241 aatccaacct gctgggccat ctgcaagaga ataccaaaca aaaaacctgg aaagaaaacc
301 accaccaagc ccacaaaaaa accaaccatc aagacaacca aaaaagatcc caaacctcaa
361 accacaaaac caaaggaagt actcaccacc aagcccacag aaaagccaac catcgacacc
421 accaaaacaa acatcagaac tacactgctc acctcctaca ccacaggaaa tccagaacac
481 acaagtcaag aggaaaccct ccactcaacc acctccgaag gcaatctaag cccatcacaa
541 gtctatacaa catccgagta cctatcacaa tctccatctt catcctacac aacaaaatga
//
LOCUS KY327962 600 bp cRNA linear VRL 13-DEC-2016
DEFINITION Human respiratory syncytial virus A isolate TH-CU477/2012
attachment glycoprotein gene, partial cds.
ACCESSION KY327962
VERSION KY327962

```
KEYWORDS    .
SOURCE      Human respiratory syncytial virus A
  ORGANISM  Human respiratory syncytial virus A
            Viruses; ssRNA viruses; ssRNA negative-strand viruses;
            Mononegavirales; Pneumoviridae; Orthopneumovirus.
REFERENCE   1  (bases 1 to 600)
  AUTHORS   Thongpan,I., Mauleekoonphairoj,J., Vichiwattana,P., Korkong,S.,
            Vongpunsawad,S. and Poovorawan,Y.
  TITLE     Molecular Characterization of Respiratory Syncytial Virus in
            Thailand, 2012-2015
  JOURNAL   Unpublished
REFERENCE   2  (bases 1 to 600)
  AUTHORS   Thongpan,I., Mauleekoonphairoj,J., Vichiwattana,P., Korkong,S.,
            Vongpunsawad,S. and Poovorawan,Y.
  TITLE     Direct Submission
  JOURNAL   Submitted (13-DEC-2016) Department of Pediatrics, Center of
            Excellence in Clinical Virology, Faculty of Medicine, Chulalongkorn
            University, Bangkok 10330, Thailand
COMMENT     ##Assembly-Data-START##
            Assembly Method       :: DNASTAR-Lasergene v. 6
            Sequencing Technology :: Sanger dideoxy sequencing
            ##Assembly-Data-END##
FEATURES             Location/Qualifiers
     source          1..600
                     /organism="Human respiratory syncytial virus A"
                     /mol_type="viral cRNA"
                     /isolate="TH-CU477/2012"
                     /host="Homo sapiens"
                     /db_xref="taxon:208893"
                     /country="Thailand"
                     /collection_date="01-Oct-2012"
                     /note="group: A"
     CDS             <1..600
                     /note="G protein"
                     /codon_start=1
                     /product="attachment glycoprotein"
                     /protein_id="APY20303"
                     /translation="ISFSNLSGTTSQSTTILASTTPSAESTPQSTTVKIKNTTTTQIQ
                     PSKPTTKQRQNKPQNKPNNDFHFEVFNFVPCSICSNNPTCWAICKRIPNKKPGKKTTT
                     KPTKKPTIKTTKKDPKPQTTKPKEVLTTKPTEKPTIDTTKTNIRTTLLTSYTTGNPEH
                     TSQEETLHSTTSEGNLSPSQVYTTSEYLSQSPSSSYTTK"
ORIGIN
        1 atcagcttct ccaatctgtc cggaactaca tcacaatcca ccaccatact agcttcaaca
       61 acaccaagtg ctgagtcaac cccacaatcc acaacagtca agatcaaaaa cacaacaaca
      121 acccaaatac aaccaagcaa acccaccaca aaacaacgcc aaaataaacc acaaaacaaa
      181 cccaacaatg attttcactt tgaagtgttc aactttgtac cctgcagcat atgcagcaac
      241 aatccaacct gctgggccat ctgcaagaga ataccaaaca aaaaacctgg aaagaaaacc
      301 accaccaagc ccacaaaaaa accaaccatc aagacaacca aaaaagatcc caaacctcaa
      361 accacaaaac caaaggaagt actcaccacc aagcccacag aaaagccaac catcgacacc
      421 accaaaacaa acatcagaac tacactgctc acctcctaca ccacaggaaa tccagaacac
      481 acaagtcaag aggaaaccct ccactcaacc acctccgaag gcaatctaag cccatcacaa
      541 gtctatacaa catccgagta cctatcacaa tccccatctt catcctacac aacaaaatga
//
LOCUS       KY327963                 600 bp    cRNA    linear   VRL 13-DEC-2016
```

DEFINITION Human respiratory syncytial virus A isolate TH-CU/C2599/2012
attachment glycoprotein gene, partial cds.
ACCESSION   KY327963
VERSION   KY327963
KEYWORDS   .
SOURCE   Human respiratory syncytial virus A
ORGANISM Human respiratory syncytial virus A
Viruses; ssRNA viruses; ssRNA negative-strand viruses;
Mononegavirales; Pneumoviridae; Orthopneumovirus.
REFERENCE   1 (bases 1 to 600)
AUTHORS   Thongpan,I., Mauleekoonphairoj,J., Vichiwattana,P., Korkong,S.,
Vongpunsawad,S. and Poovorawan,Y.
TITLE   Molecular Characterization of Respiratory Syncytial Virus in
Thailand, 2012-2015
JOURNAL   Unpublished
REFERENCE   2 (bases 1 to 600)
AUTHORS   Thongpan,I., Mauleekoonphairoj,J., Vichiwattana,P., Korkong,S.,
Vongpunsawad,S. and Poovorawan,Y.
TITLE   Direct Submission
JOURNAL   Submitted (13-DEC-2016) Department of Pediatrics, Center of
Excellence in Clinical Virology, Faculty of Medicine, Chulalongkorn
University, Bangkok 10330, Thailand
COMMENT   ##Assembly-Data-START##
Assembly Method   :: DNASTAR-Lasergene v. 6
Sequencing Technology :: Sanger dideoxy sequencing
##Assembly-Data-END##
FEATURES   Location/Qualifiers
source   1..600
/organism="Human respiratory syncytial virus A"
/mol_type="viral cRNA"
/isolate="TH-CU/C2599/2012"
/host="Homo sapiens"
/db_xref="taxon:208893"
/country="Thailand"
/collection_date="01-Feb-2012"
/note="group: A"
CDS   <1..600
/note="G protein"
/codon_start=1
/product="attachment glycoprotein"
/protein_id="APY20304"
/translation="ISFSNLSGTTSQSTTILASTTPSAESTPQSTTVKIKNTTTTQIQ
PSKPTTKQRQNKPQNKPNNDFHFEVFNFVPCSICSNNPTCWAICKRIPNKKPGKKTTT
KPTKKPTIKTTKKDPKPQTTKPKEVLTTKPTEKPTINTTKTNIRTTLLTSYTTGNPEH
TSQEETLHSTTSEGNISPSQVYTTSEYLSQSPSSSYTTK"
ORIGIN
1 atcagcttct ccaatctgtc cggaactaca tcacaatcca ccaccatact agcttcaaca
61 acaccaagtg ctgagtcaac ccccacaatcc acaacagtca agatcaaaaa cacaacaaca
121 acccaaatac aacctagcaa acccaccaca aaacaacgcc aaaataaacc acaaaacaaa
181 cccaacaatg attttcactt tgaagtgttc aactttgtac cctgcagcat atgcagcaac
241 aatccaacct gctgggccat ctgcaagaga ataccaaaca aaaaacctgg aaagaaaacc
301 accaccaagc ccacaaaaaa accaaccatc aagacaacca aaaaagatcc caaacctcaa
361 accacaaaac caaaggaagt actcaccacc aagcccacag aaaagccaac catcaacacc
421 accaaaacaa acatcagaac tacactgctc acctcctaca ccacaggaaa tccagaacac

file:///D|/Final%20peerJ/GenBank%20KY327937-KY328148_.txt[3/10/2560 14:59:50]

```
     481 acaagtcaag aggaaaccct ccactcaacc acctccgaag gcaatataag cccatcacaa
     541 gtctatacaa catccgagta cctatcacaa tctccatctt catcctacac aacaaaatga
//
LOCUS       KY327964                 600 bp    cRNA    linear   VRL 13-DEC-2016
DEFINITION  Human respiratory syncytial virus A isolate TH-CU/C3035/2012
            attachment glycoprotein gene, partial cds.
ACCESSION   KY327964
VERSION     KY327964
KEYWORDS    .
SOURCE      Human respiratory syncytial virus A
  ORGANISM  Human respiratory syncytial virus A
            Viruses; ssRNA viruses; ssRNA negative-strand viruses;
            Mononegavirales; Pneumoviridae; Orthopneumovirus.
REFERENCE   1  (bases 1 to 600)
  AUTHORS   Thongpan,I., Mauleekoonphairoj,J., Vichiwattana,P., Korkong,S.,
            Vongpunsawad,S. and Poovorawan,Y.
  TITLE     Molecular Characterization of Respiratory Syncytial Virus in
            Thailand, 2012-2015
  JOURNAL   Unpublished
REFERENCE   2  (bases 1 to 600)
  AUTHORS   Thongpan,I., Mauleekoonphairoj,J., Vichiwattana,P., Korkong,S.,
            Vongpunsawad,S. and Poovorawan,Y.
  TITLE     Direct Submission
  JOURNAL   Submitted (13-DEC-2016) Department of Pediatrics, Center of
            Excellence in Clinical Virology, Faculty of Medicine, Chulalongkorn
            University, Bangkok 10330, Thailand
COMMENT     ##Assembly-Data-START##
            Assembly Method       :: DNASTAR-Lasergene v. 6
            Sequencing Technology :: Sanger dideoxy sequencing
            ##Assembly-Data-END##
FEATURES             Location/Qualifiers
     source          1..600
                     /organism="Human respiratory syncytial virus A"
                     /mol_type="viral cRNA"
                     /isolate="TH-CU/C3035/2012"
                     /host="Homo sapiens"
                     /db_xref="taxon:208893"
                     /country="Thailand"
                     /collection_date="01-Jul-2012"
                     /note="group: A"
     CDS             <1..600
                     /note="G protein"
                     /codon_start=1
                     /product="attachment glycoprotein"
                     /protein_id="APY20305"
                     /translation="ISFSNLSGTTSQSTTILASTTPSAESTPQSTTVKIKNTTTTQIQ
                     PSKPTTKQRQNKPQNKPNNDFHFEVFNFVPCSICSNNPTCWAICKRIPNKKPGKKTTT
                     KPTKKPTIKTTKKDPKPQTTKPKEVLTTKPTEKSTIDTTKTNIRTTLLTSYTTGNPEH
                     TSQEETLHSTTSEGNISPSQVYTTSEYLSQSPSSSYTTQ"
ORIGIN
        1 atcagcttct ccaatctgtc cggaactaca tcacaatcca ccaccatact agcttcaaca
       61 acaccaagtg ctgagtcaac ccccacaatcc acaacagtca agatcaaaaa cacaacaaca
      121 acccaaatac aacctagcaa acccaccaca aaacaacgcc aaaataaacc acaaaacaaa
      181 cccaacaatg attttcactt tgaagtgttc aactttgtac cctgcagcat atgcagcaac
```

```
     241 aatccaacct gctgggccat ctgcaagaga ataccaaaca aaaaacctgg aaagaaaacc
     301 accaccaagc ccacaaaaaa accaaccatc aagacaacca aaaaagatcc caaacctcaa
     361 accacaaaac caaaggaagt actcaccacc aagcccacag aaaagtcaac catcgacacc
     421 accaaaacaa acatcagaac tacactgctc acctcctaca ccacaggaaa tccagaacac
     481 acaagtcaag aggaaaccct ccactcaacc acctccgaag gcaatataag cccatcacaa
     541 gtctatacaa catccgagta cctatcacaa tctccatctt catcctacac aacacaatga
//
LOCUS       KY327965                 600 bp    cRNA    linear   VRL 13-DEC-2016
DEFINITION  Human respiratory syncytial virus A isolate TH-CU/C2953/2012
            attachment glycoprotein gene, partial cds.
ACCESSION   KY327965
VERSION     KY327965
KEYWORDS    .
SOURCE      Human respiratory syncytial virus A
  ORGANISM  Human respiratory syncytial virus A
            Viruses; ssRNA viruses; ssRNA negative-strand viruses;
            Mononegavirales; Pneumoviridae; Orthopneumovirus.
REFERENCE   1  (bases 1 to 600)
  AUTHORS   Thongpan,I., Mauleekoonphairoj,J., Vichiwattana,P., Korkong,S.,
            Vongpunsawad,S. and Poovorawan,Y.
  TITLE     Molecular Characterization of Respiratory Syncytial Virus in
            Thailand, 2012-2015
  JOURNAL   Unpublished
REFERENCE   2  (bases 1 to 600)
  AUTHORS   Thongpan,I., Mauleekoonphairoj,J., Vichiwattana,P., Korkong,S.,
            Vongpunsawad,S. and Poovorawan,Y.
  TITLE     Direct Submission
  JOURNAL   Submitted (13-DEC-2016) Department of Pediatrics, Center of
            Excellence in Clinical Virology, Faculty of Medicine, Chulalongkorn
            University, Bangkok 10330, Thailand
COMMENT     ##Assembly-Data-START##
            Assembly Method       :: DNASTAR-Lasergene v. 6
            Sequencing Technology :: Sanger dideoxy sequencing
            ##Assembly-Data-END##
FEATURES             Location/Qualifiers
     source          1..600
                     /organism="Human respiratory syncytial virus A"
                     /mol_type="viral cRNA"
                     /isolate="TH-CU/C2953/2012"
                     /host="Homo sapiens"
                     /db_xref="taxon:208893"
                     /country="Thailand"
                     /collection_date="01-Jul-2012"
                     /note="group: A"
     CDS             <1..600
                     /note="G protein"
                     /codon_start=1
                     /product="attachment glycoprotein"
                     /protein_id="APY20306"
                     /translation="ISFSNLSGTTSQSTTILASTTPSAESTPQSTTVKIKNTTTTQIQ
                     PSKPTTKQRQNKPQNKPNNDFHFEVFNFVPCSICSNNPTCWAICKRIPNKKPGKKTTT
                     KPTKKPTIKTTKKDPKPQTTKPKEVLTTKPTEKPTIDTTKTNIRTTLLTSNTTGNPEH
                     TSQEETLHSTTSEGNLSPSQVYTTSEYLSQSPSSSNTSK"
ORIGIN
```

```
        1 atcagcttct ccaatctgtc cggaactaca tcacaatcca ccaccatact agcttcaaca
       61 acaccaagtg ctgagtcaac cccacaatcc acaacagtca agatcaaaaa cacaacaaca
      121 acccaaatac aacctagcaa acccaccaca aaacaacgcc aaaataaacc acaaaacaaa
      181 cccaacaatg attttcactt tgaagtgttc aattttgtac cctgcagcat atgcagcaac
      241 aatccaacct gctgggccat ctgcaagaga ataccaaaca aaaaacctgg aaagaaaacc
      301 accaccaagc ccacaaaaaa accaaccatc aagacaacca aaaaagatcc caaacctcaa
      361 accacaaaac caaaggaagt actcaccacc aagcccacag aaaagccaac catcgacacc
      421 accaaaacaa acatcagaac tacactgctc acctccaaca ccacaggaaa tccagaacac
      481 acaagtcaag aggaaaccct ccactcaacc acctccgaag gcaatctaag cccatcacaa
      541 gtctatacaa catccgagta cctatcacaa tctccatctt catccaacac atcaaaatga
//
LOCUS       KY327966                 600 bp    cRNA    linear   VRL 13-DEC-2016
DEFINITION  Human respiratory syncytial virus A isolate TH-CU/C3050/2012
            attachment glycoprotein gene, partial cds.
ACCESSION   KY327966
VERSION     KY327966
KEYWORDS    .
SOURCE      Human respiratory syncytial virus A
  ORGANISM  Human respiratory syncytial virus A
            Viruses; ssRNA viruses; ssRNA negative-strand viruses;
            Mononegavirales; Pneumoviridae; Orthopneumovirus.
REFERENCE   1  (bases 1 to 600)
  AUTHORS   Thongpan,I., Mauleekoonphairoj,J., Vichiwattana,P., Korkong,S.,
            Vongpunsawad,S. and Poovorawan,Y.
  TITLE     Molecular Characterization of Respiratory Syncytial Virus in
            Thailand, 2012-2015
  JOURNAL   Unpublished
REFERENCE   2  (bases 1 to 600)
  AUTHORS   Thongpan,I., Mauleekoonphairoj,J., Vichiwattana,P., Korkong,S.,
            Vongpunsawad,S. and Poovorawan,Y.
  TITLE     Direct Submission
  JOURNAL   Submitted (13-DEC-2016) Department of Pediatrics, Center of
            Excellence in Clinical Virology, Faculty of Medicine, Chulalongkorn
            University, Bangkok 10330, Thailand
COMMENT     ##Assembly-Data-START##
            Assembly Method       :: DNASTAR-Lasergene v. 6
            Sequencing Technology :: Sanger dideoxy sequencing
            ##Assembly-Data-END##
FEATURES             Location/Qualifiers
     source          1..600
                     /organism="Human respiratory syncytial virus A"
                     /mol_type="viral cRNA"
                     /isolate="TH-CU/C3050/2012"
                     /host="Homo sapiens"
                     /db_xref="taxon:208893"
                     /country="Thailand"
                     /collection_date="01-Jul-2012"
                     /note="group: A"
     CDS             <1..600
                     /note="G protein"
                     /codon_start=1
                     /product="attachment glycoprotein"
                     /protein_id="APY20307"
                     /translation="ISFSNLSGTTSQSTTILASTTPSAESTPQSTTVKIKNTTTTQIQ
```

PSKPTTKQRQNKPQNKPNNDFHFEVFNFVPCSICSNNPTCWAICKRIPNKKPGKKTTT
KPTKKPTIKTTKKDPKPQTTKPKEVLTTKPTEKPTIDTTKTNIRTTLLTSNTTGNPEH
TSQEETLHSTTSEGNLSPSQVYTTSEYLSQSPSSSNTSK"

```
ORIGIN
        1 atcagcttct ccaatctgtc cggaactaca tcacaatcca ccaccatact agcttcaaca
       61 acaccaagtg ctgagtcaac cccacaatcc acaacagtca agatcaaaaa cacaacaaca
      121 acccaaatac aacctagcaa acccaccaca aaacaacgcc aaaataaacc acaaaacaaa
      181 cccaacaatg attttcactt tgaagtgttc aattttgtac cctgcagcat atgcagcaac
      241 aatccaacct gctgggccat ctgcaagaga ataccaaaca aaaaacctgg aaagaaaacc
      301 accaccaagc ccacaaaaaa accaaccatc aagacaacca aaaaagatcc caaacctcaa
      361 accacaaaac caaaggaagt actcaccacc aagcccacag aaaagccaac catcgacacc
      421 accaaaacaa acatcagaac tacactgctc acctccaaca ccacaggaaa tccagaacac
      481 acaagtcaag aggaaaccct ccactcaacc acctccgaag gcaatctaag cccatcacaa
      541 gtctatacaa catccgagta cctatcacaa tctccatctt catccaacac atcaaaatga
//
LOCUS       KY327967                 600 bp    cRNA    linear   VRL 13-DEC-2016
DEFINITION  Human respiratory syncytial virus A isolate TH-CU/C3146/2012
            attachment glycoprotein gene, partial cds.
ACCESSION   KY327967
VERSION     KY327967
KEYWORDS    .
SOURCE      Human respiratory syncytial virus A
  ORGANISM  Human respiratory syncytial virus A
            Viruses; ssRNA viruses; ssRNA negative-strand viruses;
            Mononegavirales; Pneumoviridae; Orthopneumovirus.
REFERENCE   1  (bases 1 to 600)
  AUTHORS   Thongpan,I., Mauleekoonphairoj,J., Vichiwattana,P., Korkong,S.,
            Vongpunsawad,S. and Poovorawan,Y.
  TITLE     Molecular Characterization of Respiratory Syncytial Virus in
            Thailand, 2012-2015
  JOURNAL   Unpublished
REFERENCE   2  (bases 1 to 600)
  AUTHORS   Thongpan,I., Mauleekoonphairoj,J., Vichiwattana,P., Korkong,S.,
            Vongpunsawad,S. and Poovorawan,Y.
  TITLE     Direct Submission
  JOURNAL   Submitted (13-DEC-2016) Department of Pediatrics, Center of
            Excellence in Clinical Virology, Faculty of Medicine, Chulalongkorn
            University, Bangkok 10330, Thailand
COMMENT     ##Assembly-Data-START##
            Assembly Method       :: DNASTAR-Lasergene v. 6
            Sequencing Technology :: Sanger dideoxy sequencing
            ##Assembly-Data-END##
FEATURES             Location/Qualifiers
     source          1..600
                     /organism="Human respiratory syncytial virus A"
                     /mol_type="viral cRNA"
                     /isolate="TH-CU/C3146/2012"
                     /host="Homo sapiens"
                     /db_xref="taxon:208893"
                     /country="Thailand"
                     /collection_date="01-Sep-2012"
                     /note="group: A"
     CDS             <1..600
                     /note="G protein"
```

```
                     /codon_start=1
                     /product="attachment glycoprotein"
                     /protein_id="APY20308"
                     /translation="ISFSNLSGTTSQSTTILASTTPSAESTPQSTTVKIKNTTTTQIQ
                     PSKPTTKQRQNKPQNKPNNDFHFEVFNFVPCSICSNNPTCWAICKRIPNKKPGKKTTT
                     KPTKKPTIKTTKKDPKPQTTKPKEVLTTKPTEKPTIDTTKTNIRTTLLTSNTTGNPEH
                     TSQEETLHSTTSEGNLSPSQVYTTSEYLSQSPSSSNTSK"
ORIGIN
        1 atcagcttct ccaatctgtc cggaactaca tcacaatcca ccaccatact agcttcaaca
       61 acaccaagtg ctgagtcaac cccacaatcc acaacagtca agatcaaaaa cacaacaaca
      121 acccaaatac aacctagcaa acccaccaca aaacaacgcc aaaataaacc acaaaacaaa
      181 cccaacaatg attttcactt tgaagtgttc aattttgtac cctgcagcat atgcagcaac
      241 aatccaacct gctgggccat ctgcaagaga ataccaaaca aaaaacctgg aaagaaaacc
      301 accaccaagc ccacaaaaaa accaaccatc aagacaacca aaaaagatcc caaacctcaa
      361 accacaaaac caaaggaagt actcaccacc aagcccacag aaaagccaac catcgacacc
      421 accaaaacaa acatcagaac tacactgctc acctccaaca ccacaggaaa tccagaacac
      481 acaagtcaag aggaaaccct ccactcaacc acctccgaag gcaatctaag cccatcacaa
      541 gtctatacaa catccgagta cctatcacaa tctccatctt catccaacac atcaaaatga
//
LOCUS       KY327968                 600 bp    cRNA    linear   VRL 13-DEC-2016
DEFINITION  Human respiratory syncytial virus A isolate TH-CU/C3247/2012
            attachment glycoprotein gene, partial cds.
ACCESSION   KY327968
VERSION     KY327968
KEYWORDS    .
SOURCE      Human respiratory syncytial virus A
  ORGANISM  Human respiratory syncytial virus A
            Viruses; ssRNA viruses; ssRNA negative-strand viruses;
            Mononegavirales; Pneumoviridae; Orthopneumovirus.
REFERENCE   1  (bases 1 to 600)
  AUTHORS   Thongpan,I., Mauleekoonphairoj,J., Vichiwattana,P., Korkong,S.,
            Vongpunsawad,S. and Poovorawan,Y.
  TITLE     Molecular Characterization of Respiratory Syncytial Virus in
            Thailand, 2012-2015
  JOURNAL   Unpublished
REFERENCE   2  (bases 1 to 600)
  AUTHORS   Thongpan,I., Mauleekoonphairoj,J., Vichiwattana,P., Korkong,S.,
            Vongpunsawad,S. and Poovorawan,Y.
  TITLE     Direct Submission
  JOURNAL   Submitted (13-DEC-2016) Department of Pediatrics, Center of
            Excellence in Clinical Virology, Faculty of Medicine, Chulalongkorn
            University, Bangkok 10330, Thailand
COMMENT     ##Assembly-Data-START##
            Assembly Method       :: DNASTAR-Lasergene v. 6
            Sequencing Technology :: Sanger dideoxy sequencing
            ##Assembly-Data-END##
FEATURES             Location/Qualifiers
     source          1..600
                     /organism="Human respiratory syncytial virus A"
                     /mol_type="viral cRNA"
                     /isolate="TH-CU/C3247/2012"
                     /host="Homo sapiens"
                     /db_xref="taxon:208893"
                     /country="Thailand"
```

/collection_date="01-Sep-2012"
/note="group: A"
CDS <1..600
/note="G protein"
/codon_start=1
/product="attachment glycoprotein"
/protein_id="APY20309"
/translation="ISFSNLSGTTSQSTTILASTTPSAESTPQSTTVKIKNTTTTQIQ
PSKPTTKQRQNKPQNKPNNDFHFEVFNFVPCSICSNNPTCWAICKRIPNKKPGKKTTT
KPTKKPTIKTTKKDPKPQTTKPKEVLTTKPTEKPTIDTTKTNIRTTLLTSNTTGNPEH
TSQEETLHSTTSEGNLSPSQVYTTSEYLSQSPSSSNTSK"
ORIGIN
1 atcagcttct ccaatctgtc cggaactaca tcacaatcca ccaccatact agcttcaaca
61 acaccaagtg ctgagtcaac cccacaatcc acaacagtca agatcaaaaa cacaacaaca
121 acccaaatac aacctagcaa acccaccaca aaacaacgcc aaaataaacc acaaaacaaa
181 cccaacaatg attttcactt tgaagtgttc aattttgtac cctgcagcat atgcagcaac
241 aatccaacct gctgggccat ctgcaagaga ataccaaaca aaaaacctgg aaagaaaacc
301 accaccaagc ccacaaaaaa accaaccatc aagacaacca aaaaagatcc caaacctcaa
361 accacaaaac caaaggaagt actcaccacc aagcccacag aaaagccaac catcgacacc
421 accaaaacaa acatcagaac tacactgctc acctccaaca ccacaggaaa tccagaacac
481 acaagtcaag aggaaaccct ccactcaacc acctccgaag gcaatctaag cccatcacaa
541 gtctatacaa catccgagta cctatcacaa tctccatctt catccaacac atcaaaatga
//
LOCUS KY327969 600 bp cRNA linear VRL 13-DEC-2016
DEFINITION Human respiratory syncytial virus A isolate TH-CU/C3057/2012
attachment glycoprotein gene, partial cds.
ACCESSION KY327969
VERSION KY327969
KEYWORDS .
SOURCE Human respiratory syncytial virus A
ORGANISM Human respiratory syncytial virus A
Viruses; ssRNA viruses; ssRNA negative-strand viruses;
Mononegavirales; Pneumoviridae; Orthopneumovirus.
REFERENCE 1 (bases 1 to 600)
AUTHORS Thongpan,I., Mauleekoonphairoj,J., Vichiwattana,P., Korkong,S.,
Vongpunsawad,S. and Poovorawan,Y.
TITLE Molecular Characterization of Respiratory Syncytial Virus in
Thailand, 2012-2015
JOURNAL Unpublished
REFERENCE 2 (bases 1 to 600)
AUTHORS Thongpan,I., Mauleekoonphairoj,J., Vichiwattana,P., Korkong,S.,
Vongpunsawad,S. and Poovorawan,Y.
TITLE Direct Submission
JOURNAL Submitted (13-DEC-2016) Department of Pediatrics, Center of
Excellence in Clinical Virology, Faculty of Medicine, Chulalongkorn
University, Bangkok 10330, Thailand
COMMENT ##Assembly-Data-START##
Assembly Method :: DNASTAR-Lasergene v. 6
Sequencing Technology :: Sanger dideoxy sequencing
##Assembly-Data-END##
FEATURES Location/Qualifiers
source 1..600
/organism="Human respiratory syncytial virus A"
/mol_type="viral cRNA"

/isolate="TH-CU/C3057/2012"
/host="Homo sapiens"
/db_xref="taxon:208893"
/country="Thailand"
/collection_date="01-Aug-2012"
/note="group: A"
CDS <1..600
/note="G protein"
/codon_start=1
/product="attachment glycoprotein"
/protein_id="APY20310"
/translation="ISFSNLSGTTSQSTTILASTTPSAESTPQSTTVKIKNTTTTQIQ
PSKPTTKQRQNKPQNKPNNDFHFEVFNFVPCSICSNNPTCWAICKRIPNKKPGKKTTT
KPTKKPTIKTTKKDPKPQTTKPKEVLTTKPTEKPTIDTTKTNIRTTLLTSNTTGNPEH
TSQEETLHSTTSEGNLSPSQVYTTSEYLSQSPSSSNTSK"
ORIGIN
1 atcagcttct ccaatctgtc cggaactaca tcacaatcca ccaccatact agcttcaaca
61 acaccaagtg ctgagtcaac cccacaatcc acaacagtca agatcaaaaa cacaacaaca
121 acccaaatac aacctagcaa acccaccaca aaacaacgcc aaaataaacc acaaaacaaa
181 cccaacaatg attttcactt tgaagtgttc aattttgtac cctgcagcat atgcagcaac
241 aatccaacct gctgggccat ctgcaagaga ataccaaaca aaaaacctgg aaagaaaacc
301 accaccaagc ccacaaaaaa accaaccatc aagacaacca aaaaagatcc caaacctcaa
361 accacaaaac caaaggaagt actcaccacc aagcccacag aaaagccaac catcgacacc
421 accaaaacaa acatcagaac tacactgctc acctccaaca ccacaggaaa tccagaacac
481 acaagtcaag aggaaaccct ccactcaacc acctccgaag gcaatctaag cccatcacaa
541 gtctatacaa catccgagta cctatcacaa tctccatctt catccaacac atcaaaatga
//
LOCUS KY327970 600 bp cRNA linear VRL 13-DEC-2016
DEFINITION Human respiratory syncytial virus A isolate TH-CU/C3176/2012
attachment glycoprotein gene, partial cds.
ACCESSION KY327970
VERSION KY327970
KEYWORDS .
SOURCE Human respiratory syncytial virus A
ORGANISM Human respiratory syncytial virus A
Viruses; ssRNA viruses; ssRNA negative-strand viruses;
Mononegavirales; Pneumoviridae; Orthopneumovirus.
REFERENCE 1 (bases 1 to 600)
AUTHORS Thongpan,I., Mauleekoonphairoj,J., Vichiwattana,P., Korkong,S.,
Vongpunsawad,S. and Poovorawan,Y.
TITLE Molecular Characterization of Respiratory Syncytial Virus in
Thailand, 2012-2015
JOURNAL Unpublished
REFERENCE 2 (bases 1 to 600)
AUTHORS Thongpan,I., Mauleekoonphairoj,J., Vichiwattana,P., Korkong,S.,
Vongpunsawad,S. and Poovorawan,Y.
TITLE Direct Submission
JOURNAL Submitted (13-DEC-2016) Department of Pediatrics, Center of
Excellence in Clinical Virology, Faculty of Medicine, Chulalongkorn
University, Bangkok 10330, Thailand
COMMENT ##Assembly-Data-START##
Assembly Method :: DNASTAR-Lasergene v. 6
Sequencing Technology :: Sanger dideoxy sequencing
##Assembly-Data-END##

FEATURES   Location/Qualifiers
     source   1..600
                     /organism="Human respiratory syncytial virus A"
                     /mol_type="viral cRNA"
                     /isolate="TH-CU/C3176/2012"
                     /host="Homo sapiens"
                     /db_xref="taxon:208893"
                     /country="Thailand"
                     /collection_date="01-Sep-2012"
                     /note="group: A"
     CDS   <1..600
                     /note="G protein"
                     /codon_start=1
                     /product="attachment glycoprotein"
                     /protein_id="APY20311"
                     /translation="ISFSNLSGTTSQSTTILASTTPSAESTPQSTTVKIKNTTTTQIQ
                     PSKPTTKQRQNKPQNKPNNDFHFEVFNFVPCSICSNNPTCWAICKRIPNKKPGKKTTT
                     KPTKKPTIKTTKKDPKPQTTKPKEVLTTKPTEKPTIDTTKTNIRTTLLTSNTTGNPEH
                     TSQEETLHSTTSEGNLSPSQVYTTSEYLSQSPSSSNTSK"
ORIGIN
        1 atcagcttct ccaatctgtc cggaactaca tcacaatcca ccaccatact agcttcaaca
       61 acaccaagtg ctgagtcaac cccacaatcc acaacagtca agatcaaaaa cacaacaaca
      121 acccaaatac aacctagcaa acccaccaca aaacaacgcc aaaataaacc acaaaacaaa
      181 cccaacaatg attttcactt tgaagtgttc aattttgtac cctgcagcat atgcagcaac
      241 aatccaacct gctgggccat ctgcaagaga ataccaaaca aaaaacctgg aaagaaaacc
      301 accaccaagc ccacaaaaaa accaaccatc aagacaacca aaaaagatcc caaacctcaa
      361 accacaaaac caaaggaagt actcaccacc aagcccacag aaaagccaac catcgacacc
      421 accaaaacaa acatcagaac tacactgctc acctccaaca ccacaggaaa tccagaacac
      481 acaagtcaag aggaaaccct ccactcaacc acctccgaag gcaatctaag cccatcacaa
      541 gtctatacaa catccgagta cctatcacaa tctccatctt catccaacac atcaaaatga
//
LOCUS   KY327971   600 bp   cRNA   linear   VRL 13-DEC-2016
DEFINITION Human respiratory syncytial virus A isolate TH-CU436/2012
            attachment glycoprotein gene, partial cds.
ACCESSION   KY327971
VERSION   KY327971
KEYWORDS   .
SOURCE   Human respiratory syncytial virus A
  ORGANISM Human respiratory syncytial virus A
            Viruses; ssRNA viruses; ssRNA negative-strand viruses;
            Mononegavirales; Pneumoviridae; Orthopneumovirus.
REFERENCE   1 (bases 1 to 600)
  AUTHORS   Thongpan,I., Mauleekoonphairoj,J., Vichiwattana,P., Korkong,S.,
            Vongpunsawad,S. and Poovorawan,Y.
  TITLE   Molecular Characterization of Respiratory Syncytial Virus in
            Thailand, 2012-2015
  JOURNAL   Unpublished
REFERENCE   2 (bases 1 to 600)
  AUTHORS   Thongpan,I., Mauleekoonphairoj,J., Vichiwattana,P., Korkong,S.,
            Vongpunsawad,S. and Poovorawan,Y.
  TITLE   Direct Submission
  JOURNAL   Submitted (13-DEC-2016) Department of Pediatrics, Center of
            Excellence in Clinical Virology, Faculty of Medicine, Chulalongkorn
            University, Bangkok 10330, Thailand

```
COMMENT     ##Assembly-Data-START##
            Assembly Method       :: DNASTAR-Lasergene v. 6
            Sequencing Technology :: Sanger dideoxy sequencing
            ##Assembly-Data-END##
FEATURES             Location/Qualifiers
     source          1..600
                     /organism="Human respiratory syncytial virus A"
                     /mol_type="viral cRNA"
                     /isolate="TH-CU436/2012"
                     /host="Homo sapiens"
                     /db_xref="taxon:208893"
                     /country="Thailand"
                     /collection_date="01-Sep-2012"
                     /note="group: A"
     CDS             <1..600
                     /note="G protein"
                     /codon_start=1
                     /product="attachment glycoprotein"
                     /protein_id="APY20312"
                     /translation="ISFSNLSGTTSQSTTILASTTPSAESTPQSTTVKIKNTTTTQIQ
                     PSKPTTKQRQNKPQNKPNNDFHFEVFNFVPCSICSNNPTCWAICKRIPNKKPGKKTTT
                     KPTKKPTIKTTKKDPKPQTTKPKEVLTTKPTEKPTIDTTKTNIRTTLLTSNTTGNPEH
                     TSQEETLHSTTSEGNLSPSQVYTTSEYLSQSPSSSNTSK"
ORIGIN
        1 atcagcttct ccaatctgtc cggaactaca tcacaatcca ccaccatact agcttcaaca
       61 acaccaagtg ctgagtcaac cccacaatcc acaacagtca agatcaaaaa cacaacaaca
      121 acccaaatac aacctagcaa acccaccaca aaacaacgcc aaaataaacc acaaaacaaa
      181 cccaacaatg attttcactt tgaagtgttc aattttgtac cctgcagcat atgcagcaac
      241 aatccaacct gctgggccat ctgcaagaga ataccaaaca aaaaacctgg aaagaaaacc
      301 accaccaagc ccacaaaaaa accaaccatc aagacaacca aaaaagatcc caaacctcaa
      361 accacaaaac caaaggaagt actcaccacc aagcccacag aaaagccaac catcgacacc
      421 accaaaacaa acatcagaac tacactgctc acctccaaca ccacaggaaa tccagaacac
      481 acaagtcaag aggaaaccct ccactcaacc acctccgaag gcaatctaag cccatcacaa
      541 gtctatacaa catccgagta cctatcacaa tctccatctt catccaacac atcaaaatga
//
LOCUS       KY327972                 600 bp    cRNA    linear   VRL 13-DEC-2016
DEFINITION  Human respiratory syncytial virus A isolate TH-CU488/2012
            attachment glycoprotein gene, partial cds.
ACCESSION   KY327972
VERSION     KY327972
KEYWORDS    .
SOURCE      Human respiratory syncytial virus A
  ORGANISM  Human respiratory syncytial virus A
            Viruses; ssRNA viruses; ssRNA negative-strand viruses;
            Mononegavirales; Pneumoviridae; Orthopneumovirus.
REFERENCE   1  (bases 1 to 600)
  AUTHORS   Thongpan,I., Mauleekoonphairoj,J., Vichiwattana,P., Korkong,S.,
            Vongpunsawad,S. and Poovorawan,Y.
  TITLE     Molecular Characterization of Respiratory Syncytial Virus in
            Thailand, 2012-2015
  JOURNAL   Unpublished
REFERENCE   2  (bases 1 to 600)
  AUTHORS   Thongpan,I., Mauleekoonphairoj,J., Vichiwattana,P., Korkong,S.,
            Vongpunsawad,S. and Poovorawan,Y.
```

```
  TITLE     Direct Submission
  JOURNAL   Submitted (13-DEC-2016) Department of Pediatrics, Center of
            Excellence in Clinical Virology, Faculty of Medicine, Chulalongkorn
            University, Bangkok 10330, Thailand
COMMENT     ##Assembly-Data-START##
            Assembly Method       :: DNASTAR-Lasergene v. 6
            Sequencing Technology :: Sanger dideoxy sequencing
            ##Assembly-Data-END##
FEATURES             Location/Qualifiers
     source          1..600
                     /organism="Human respiratory syncytial virus A"
                     /mol_type="viral cRNA"
                     /isolate="TH-CU488/2012"
                     /host="Homo sapiens"
                     /db_xref="taxon:208893"
                     /country="Thailand"
                     /collection_date="01-Oct-2012"
                     /note="group: A"
     CDS             <1..600
                     /note="G protein"
                     /codon_start=1
                     /product="attachment glycoprotein"
                     /protein_id="APY20313"
                     /translation="ISFSNLSGTTSQSTTILASTTPSAESTPQSTTVKIKNTTTTQIQ
                     PSKPTTKQRQNKPQNKPNNDFHFEVFNFVPCSICSNNPTCWAICKRIPNKKPGKKTTT
                     KPTKKPTIKTTKKDPKPQTTKPKEVLTTKPTEKPTIDTTKTNIRTTLLTSNTTGNPEH
                     TSQEETLHSTTSEGNLSPSQVYTTSEYLSQSPSSSNTSK"
ORIGIN
        1 atcagcttct ccaatctgtc cggaactaca tcacaatcca ccaccatact agcttcaaca
       61 acaccaagtg ctgagtcaac cccacaatcc acaacagtca agatcaaaaa cacaacaaca
      121 acccaaatac aacctagcaa acccaccaca aaacaacgcc aaaataaacc acaaaacaaa
      181 cccaacaatg attttcactt tgaagtgttc aattttgtac cctgcagcat atgcagcaac
      241 aatccaacct gctgggccat ctgcaagaga ataccaaaca aaaaacctgg aaagaaaacc
      301 accaccaagc ccacaaaaaa accaaccatc aagacaacca aaaaagatcc caaacctcaa
      361 accacaaaac caaaggaagt actcaccacc aagcccacag aaaagccaac catcgacacc
      421 accaaaacaa acatcagaac tacactgctc acctccaaca ccacaggaaa tccagaacac
      481 acaagtcaag aggaaaccct ccactcaacc acctccgaag gcaatctaag cccatcacaa
      541 gtctatacaa catccgagta cctatcacaa tctccatctt catccaacac atcaaaatga
//
LOCUS       KY327973                 600 bp    cRNA    linear   VRL 13-DEC-2016
DEFINITION  Human respiratory syncytial virus A isolate TH-CU/C3132/2012
            attachment glycoprotein gene, partial cds.
ACCESSION   KY327973
VERSION     KY327973
KEYWORDS    .
SOURCE      Human respiratory syncytial virus A
  ORGANISM  Human respiratory syncytial virus A
            Viruses; ssRNA viruses; ssRNA negative-strand viruses;
            Mononegavirales; Pneumoviridae; Orthopneumovirus.
REFERENCE   1  (bases 1 to 600)
  AUTHORS   Thongpan,I., Mauleekoonphairoj,J., Vichiwattana,P., Korkong,S.,
            Vongpunsawad,S. and Poovorawan,Y.
  TITLE     Molecular Characterization of Respiratory Syncytial Virus in
            Thailand, 2012-2015
```

JOURNAL Unpublished
REFERENCE 2 (bases 1 to 600)
AUTHORS Thongpan,I., Mauleekoonphairoj,J., Vichiwattana,P., Korkong,S.,
Vongpunsawad,S. and Poovorawan,Y.
TITLE Direct Submission
JOURNAL Submitted (13-DEC-2016) Department of Pediatrics, Center of
Excellence in Clinical Virology, Faculty of Medicine, Chulalongkorn
University, Bangkok 10330, Thailand
COMMENT ##Assembly-Data-START##
Assembly Method :: DNASTAR-Lasergene v. 6
Sequencing Technology :: Sanger dideoxy sequencing
##Assembly-Data-END##
FEATURES Location/Qualifiers
source 1..600
/organism="Human respiratory syncytial virus A"
/mol_type="viral cRNA"
/isolate="TH-CU/C3132/2012"
/host="Homo sapiens"
/db_xref="taxon:208893"
/country="Thailand"
/collection_date="01-Aug-2012"
/note="group: A"
CDS <1..600
/note="G protein"
/codon_start=1
/product="attachment glycoprotein"
/protein_id="APY20314"
/translation="ISFSNLSGTTSQSTTILASTTPSAESTPQSTTVKIKNTTTTQIQ
PSKPTTKQRQNKPQNKPNNDFHFEVFNFVPCSICSNNPTCWAICKRIPNKKPGKKTTT
KPTKKPTIKTTKKDPKPQTTKPKEVLTTKPTEKPTIDTTKTNIRTTLLTSNTTGNPEH
TSQEETLHSTTSEGNLSPSQVYTTSEYLSQSPSSSNTSK"
ORIGIN
1 atcagcttct ccaatctgtc cggaactaca tcacaatcca ccaccatact agcttcaaca
61 acaccaagtg ctgagtcaac cccacaatcc acaacagtca agatcaaaaa cacaacaaca
121 acccaaatac aacctagcaa acccaccaca aaacaacgcc aaaataaacc acaaaacaaa
181 cccaacaatg attttcactt tgaagtgttc aattttgtac cctgcagcat atgcagcaac
241 aatccaacct gctgggccat ctgcaagaga ataccaaaca aaaaacctgg aaagaaaacc
301 accaccaagc ccacaaaaaa accaaccatc aagacaacca aaaaagatcc caaacctcaa
361 accacaaaac caaaggaagt actcaccacc aagcccacag aaaagccaac catcgacacc
421 accaaaacaa acatcagaac tacactgctc acctccaaca ccacaggaaa tccagaacac
481 acaagtcaag aggaaaccct ccactcaacc acctccgaag gcaatctaag cccatcacaa
541 gtctatacaa catccgagta cctatcacaa tctccatctt catccaacac atcaaaatga
//
LOCUS KY327974 600 bp cRNA linear VRL 13-DEC-2016
DEFINITION Human respiratory syncytial virus A isolate TH-CU/C3033/2012
attachment glycoprotein gene, partial cds.
ACCESSION KY327974
VERSION KY327974
KEYWORDS .
SOURCE Human respiratory syncytial virus A
ORGANISM Human respiratory syncytial virus A
Viruses; ssRNA viruses; ssRNA negative-strand viruses;
Mononegavirales; Pneumoviridae; Orthopneumovirus.
REFERENCE 1 (bases 1 to 600)

```
  AUTHORS   Thongpan,I., Mauleekoonphairoj,J., Vichiwattana,P., Korkong,S.,
            Vongpunsawad,S. and Poovorawan,Y.
  TITLE     Molecular Characterization of Respiratory Syncytial Virus in
            Thailand, 2012-2015
  JOURNAL   Unpublished
REFERENCE   2  (bases 1 to 600)
  AUTHORS   Thongpan,I., Mauleekoonphairoj,J., Vichiwattana,P., Korkong,S.,
            Vongpunsawad,S. and Poovorawan,Y.
  TITLE     Direct Submission
  JOURNAL   Submitted (13-DEC-2016) Department of Pediatrics, Center of
            Excellence in Clinical Virology, Faculty of Medicine, Chulalongkorn
            University, Bangkok 10330, Thailand
COMMENT     ##Assembly-Data-START##
            Assembly Method       :: DNASTAR-Lasergene v. 6
            Sequencing Technology :: Sanger dideoxy sequencing
            ##Assembly-Data-END##
FEATURES             Location/Qualifiers
     source          1..600
                     /organism="Human respiratory syncytial virus A"
                     /mol_type="viral cRNA"
                     /isolate="TH-CU/C3033/2012"
                     /host="Homo sapiens"
                     /db_xref="taxon:208893"
                     /country="Thailand"
                     /collection_date="01-Jul-2012"
                     /note="group: A"
     CDS             <1..600
                     /note="G protein"
                     /codon_start=1
                     /product="attachment glycoprotein"
                     /protein_id="APY20315"
                     /translation="ISFSNLSGTTSQSTTILASTTPSAESTPQSTTVKIKNTTTTQIQ
                     PSKPTTKQRQNKPQNKPNNDFHFEVFNFVPCSICSNNPTCWAICKRIPNKKPGKKTTT
                     KPTKKPTIKTTKKDPKPQTTKPKEVLTTKPTEKPTIDTTKTNIRTTLLTSNTTGNPEH
                     TSQEETLHSTTSEGNLSPSQVYTTSEYLSQSPSSSNTSK"
ORIGIN
        1 atcagcttct ccaatctgtc cggaactaca tcacaatcca ccaccatact agcttcaaca
       61 acaccaagtg ctgagtcaac ccccacaatcc acaacagtca agatcaaaaa cacaacaaca
      121 acccaaatac aacctagcaa acccaccaca aaacaacgcc aaaataaacc acaaaacaaa
      181 cccaacaatg attttcactt tgaagtgttc aattttgtac cctgcagcat atgcagcaac
      241 aatccaacct gctgggccat ctgcaagaga ataccaaaca aaaaacctgg aaagaaaacc
      301 accaccaagc ccacaaaaaa accaaccatc aagacaacca aaaaagatcc caaacctcaa
      361 accacaaaac caaaggaagt actcaccacc aagcccacag aaaagccaac catcgacacc
      421 accaaaacaa acatcagaac tacactgctc acctccaaca ccacaggaaa tccagaacac
      481 acaagtcaag aggaaaccct ccactcaacc acctccgaag gcaatctaag cccatcacaa
      541 gtctatacaa catccgagta cctatcacaa tctccatctt catccaacac atcaaaatga
//
LOCUS       KY327975                 600 bp    cRNA    linear   VRL 13-DEC-2016
DEFINITION  Human respiratory syncytial virus A isolate TH-CU/C3188/2012
            attachment glycoprotein gene, partial cds.
ACCESSION   KY327975
VERSION     KY327975
KEYWORDS    .
SOURCE      Human respiratory syncytial virus A
```

```
  ORGANISM  Human respiratory syncytial virus A
            Viruses; ssRNA viruses; ssRNA negative-strand viruses;
            Mononegavirales; Pneumoviridae; Orthopneumovirus.
REFERENCE   1  (bases 1 to 600)
  AUTHORS   Thongpan,I., Mauleekoonphairoj,J., Vichiwattana,P., Korkong,S.,
            Vongpunsawad,S. and Poovorawan,Y.
  TITLE     Molecular Characterization of Respiratory Syncytial Virus in
            Thailand, 2012-2015
  JOURNAL   Unpublished
REFERENCE   2  (bases 1 to 600)
  AUTHORS   Thongpan,I., Mauleekoonphairoj,J., Vichiwattana,P., Korkong,S.,
            Vongpunsawad,S. and Poovorawan,Y.
  TITLE     Direct Submission
  JOURNAL   Submitted (13-DEC-2016) Department of Pediatrics, Center of
            Excellence in Clinical Virology, Faculty of Medicine, Chulalongkorn
            University, Bangkok 10330, Thailand
COMMENT     ##Assembly-Data-START##
            Assembly Method       :: DNASTAR-Lasergene v. 6
            Sequencing Technology :: Sanger dideoxy sequencing
            ##Assembly-Data-END##
FEATURES             Location/Qualifiers
     source          1..600
                     /organism="Human respiratory syncytial virus A"
                     /mol_type="viral cRNA"
                     /isolate="TH-CU/C3188/2012"
                     /host="Homo sapiens"
                     /db_xref="taxon:208893"
                     /country="Thailand"
                     /collection_date="01-Sep-2012"
                     /note="group: A"
     CDS             <1..600
                     /note="G protein"
                     /codon_start=1
                     /product="attachment glycoprotein"
                     /protein_id="APY20316"
                     /translation="VSFSNLSGTTSQSTTILASTTPSAESTPQSTTVKIKNTTTTQIQ
                     PSKPTTKQRQNKPQNKPNNDFHFEVFNFVPCSICSNNPTCWAICKRIPNKKPGKKTTT
                     KPTKKPTIKTTKKDPKPQTTKPKEVLTTKPTEKPTIDTTKTNIRTTLLTSNTTGNPEH
                     TSQEETLHSTTSEGNLSPSQVYTTSEYLSQSPSSSNTSK"
ORIGIN
        1 gtcagcttct ccaatctgtc cggaactaca tcacaatcca ccaccatact agcttcaaca
       61 acaccaagtg ctgagtcaac ccccacaatcc acaacagtca agatcaaaaa cacaacaaca
      121 acccaaatac aacctagcaa acccaccaca aaacaacgcc aaaataaacc acaaaacaaa
      181 cccaacaatg attttcactt tgaagtgttc aattttgtac cctgcagcat atgcagcaac
      241 aatccaacct gctgggccat ctgcaagaga ataccaaaca aaaaacctgg aaagaaaacc
      301 accaccaagc ccacaaaaaa accaaccatc aagacaacca aaaaagatcc caaacctcaa
      361 accacaaaac caaaggaagt actcaccacc aagcccacag aaaagccaac catcgacacc
      421 accaaaacaa acatcagaac tacactgctc acctccaaca ccacaggaaa tccagaacac
      481 acaagtcaag aggaaaccct ccactcaacc acctccgaag gcaatctaag cccatcacaa
      541 gtctatacaa catccgagta cctatcacaa tctccatctt catccaacac atcaaaatga
//
LOCUS       KY327976                 600 bp    cRNA    linear   VRL 13-DEC-2016
DEFINITION  Human respiratory syncytial virus A isolate TH-CU411/2012
            attachment glycoprotein gene, partial cds.
```

```
ACCESSION   KY327976
VERSION     KY327976
KEYWORDS    .
SOURCE      Human respiratory syncytial virus A
  ORGANISM  Human respiratory syncytial virus A
            Viruses; ssRNA viruses; ssRNA negative-strand viruses;
            Mononegavirales; Pneumoviridae; Orthopneumovirus.
REFERENCE   1  (bases 1 to 600)
  AUTHORS   Thongpan,I., Mauleekoonphairoj,J., Vichiwattana,P., Korkong,S.,
            Vongpunsawad,S. and Poovorawan,Y.
  TITLE     Molecular Characterization of Respiratory Syncytial Virus in
            Thailand, 2012-2015
  JOURNAL   Unpublished
REFERENCE   2  (bases 1 to 600)
  AUTHORS   Thongpan,I., Mauleekoonphairoj,J., Vichiwattana,P., Korkong,S.,
            Vongpunsawad,S. and Poovorawan,Y.
  TITLE     Direct Submission
  JOURNAL   Submitted (13-DEC-2016) Department of Pediatrics, Center of
            Excellence in Clinical Virology, Faculty of Medicine, Chulalongkorn
            University, Bangkok 10330, Thailand
COMMENT     ##Assembly-Data-START##
            Assembly Method       :: DNASTAR-Lasergene v. 6
            Sequencing Technology :: Sanger dideoxy sequencing
            ##Assembly-Data-END##
FEATURES             Location/Qualifiers
     source          1..600
                     /organism="Human respiratory syncytial virus A"
                     /mol_type="viral cRNA"
                     /isolate="TH-CU411/2012"
                     /host="Homo sapiens"
                     /db_xref="taxon:208893"
                     /country="Thailand"
                     /collection_date="01-Aug-2012"
                     /note="group: A"
     CDS             <1..600
                     /note="G protein"
                     /codon_start=1
                     /product="attachment glycoprotein"
                     /protein_id="APY20317"
                     /translation="ISFSNLSGTTSQSTTILASTTPSAESTPQSTTVKIKNTTTTQIQ
                     PSKPTTKQRQNKPQNKPNNDFHFEVFNFVPCSICSNNPTCWAICKRIPNKKPGKKTTT
                     KPTKKPTIKTTKKDPKPQTTKPKEVLTTKPTEKPTIDTTKTNIRTTLLTSNTTGNPEH
                     TSQEETLHSTTSEGNLSPSQVYTTSEYLSQSPSSSNTSK"
ORIGIN
        1 atcagcttct ccaatctgtc cggaactaca tcacaatcca ccaccatact agcttcaaca
       61 acaccaagtg ctgagtcaac ccccacaatcc acaacagtca agatcaaaaa cacaacaaca
      121 acccaaatac aacctagcaa acccaccaca aaacaacgcc aaaataaacc acaaaacaaa
      181 cccaacaatg attttcactt tgaagtgttc aattttgtac cctgcagcat atgcagcaac
      241 aatccaacct gctgggccat ctgcaagaga ataccaaaca aaaaacctgg aaagaaaacc
      301 accaccaagc ccacaaaaaa accaaccatc aagacaacca aaaaagatcc caaacctcaa
      361 accacaaaac caaaggaagt actcaccacc aagcccacag aaaagccaac catcgacacc
      421 accaaaacaa acatcagaac tacactgctc acctccaaca ccacaggaaa tccagaacac
      481 acaagtcaag aggaaaccct ccactcaacc acctccgaag gcaatctaag cccatcacaa
      541 gtctatacaa catccgagta cctatcacaa tctccatctt catccaacac atcaaaatga
```

```
//
LOCUS       KY327977                600 bp    cRNA    linear   VRL 13-DEC-2016
DEFINITION  Human respiratory syncytial virus A isolate TH-CU455/2012
            attachment glycoprotein gene, partial cds.
ACCESSION   KY327977
VERSION     KY327977
KEYWORDS    .
SOURCE      Human respiratory syncytial virus A
  ORGANISM  Human respiratory syncytial virus A
            Viruses; ssRNA viruses; ssRNA negative-strand viruses;
            Mononegavirales; Pneumoviridae; Orthopneumovirus.
REFERENCE   1  (bases 1 to 600)
  AUTHORS   Thongpan,I., Mauleekoonphairoj,J., Vichiwattana,P., Korkong,S.,
            Vongpunsawad,S. and Poovorawan,Y.
  TITLE     Molecular Characterization of Respiratory Syncytial Virus in
            Thailand, 2012-2015
  JOURNAL   Unpublished
REFERENCE   2  (bases 1 to 600)
  AUTHORS   Thongpan,I., Mauleekoonphairoj,J., Vichiwattana,P., Korkong,S.,
            Vongpunsawad,S. and Poovorawan,Y.
  TITLE     Direct Submission
  JOURNAL   Submitted (13-DEC-2016) Department of Pediatrics, Center of
            Excellence in Clinical Virology, Faculty of Medicine, Chulalongkorn
            University, Bangkok 10330, Thailand
COMMENT     ##Assembly-Data-START##
            Assembly Method       :: DNASTAR-Lasergene v. 6
            Sequencing Technology :: Sanger dideoxy sequencing
            ##Assembly-Data-END##
FEATURES             Location/Qualifiers
     source          1..600
                     /organism="Human respiratory syncytial virus A"
                     /mol_type="viral cRNA"
                     /isolate="TH-CU455/2012"
                     /host="Homo sapiens"
                     /db_xref="taxon:208893"
                     /country="Thailand"
                     /collection_date="01-Sep-2012"
                     /note="group: A"
     CDS             <1..600
                     /note="G protein"
                     /codon_start=1
                     /product="attachment glycoprotein"
                     /protein_id="APY20318"
                     /translation="ISFSNLSGTTSQSTTILASTTPSAESTPQSTTVKIKNTTTTQIQ
                     PSKPTTKQRQNKPQNKPNNDFHFEVFNFVPCSICSNNPTCWAICKRIPNKKPGKKTTT
                     KPTKKPTIKTTKKDPKPQTTKPKEVLTTKPTEKPTIDTTKTNIRTTLLTSNTTGNPEH
                     TSQEETLHSTTSEGNLSPSQVYTTSEYLSQSPSSSNTSK"
ORIGIN
        1 atcagcttct ccaatctgtc cggaactaca tcacaatcca ccaccatact agcttcaaca
       61 acaccaagtg ctgagtcaac cccacaatcc acaacagtca agatcaaaaa cacaacaaca
      121 acccaaatac aacctagcaa acccaccaca aaacaacgcc aaaataaacc acaaaacaaa
      181 cccaacaatg attttcactt tgaagtgttc aattttgtac cctgcagcat atgcagcaac
      241 aatccaacct gctgggccat ctgcaagaga ataccaaaca aaaaacctgg aaagaaaacc
      301 accaccaagc ccacaaaaaa accaaccatc aagacaacca aaaaagatcc caaacctcaa
```

```
     361 accacaaaac caaaggaagt actcaccacc aagcccacag aaaagccaac catcgacacc
     421 accaaaacaa acatcagaac tacactgctc acctccaaca ccacaggaaa tccagaacac
     481 acaagtcaag aggaaaccct ccactcaacc acctccgaag gcaatctaag cccatcacaa
     541 gtctatacaa catccgagta cctatcacaa tctccatctt catccaacac atcaaaatga
//
LOCUS       KY327978                 600 bp    cRNA    linear   VRL 13-DEC-2016
DEFINITION  Human respiratory syncytial virus A isolate TH-CU458/2012
            attachment glycoprotein gene, partial cds.
ACCESSION   KY327978
VERSION     KY327978
KEYWORDS    .
SOURCE      Human respiratory syncytial virus A
  ORGANISM  Human respiratory syncytial virus A
            Viruses; ssRNA viruses; ssRNA negative-strand viruses;
            Mononegavirales; Pneumoviridae; Orthopneumovirus.
REFERENCE   1  (bases 1 to 600)
  AUTHORS   Thongpan,I., Mauleekoonphairoj,J., Vichiwattana,P., Korkong,S.,
            Vongpunsawad,S. and Poovorawan,Y.
  TITLE     Molecular Characterization of Respiratory Syncytial Virus in
            Thailand, 2012-2015
  JOURNAL   Unpublished
REFERENCE   2  (bases 1 to 600)
  AUTHORS   Thongpan,I., Mauleekoonphairoj,J., Vichiwattana,P., Korkong,S.,
            Vongpunsawad,S. and Poovorawan,Y.
  TITLE     Direct Submission
  JOURNAL   Submitted (13-DEC-2016) Department of Pediatrics, Center of
            Excellence in Clinical Virology, Faculty of Medicine, Chulalongkorn
            University, Bangkok 10330, Thailand
COMMENT     ##Assembly-Data-START##
            Assembly Method       :: DNASTAR-Lasergene v. 6
            Sequencing Technology :: Sanger dideoxy sequencing
            ##Assembly-Data-END##
FEATURES             Location/Qualifiers
     source          1..600
                     /organism="Human respiratory syncytial virus A"
                     /mol_type="viral cRNA"
                     /isolate="TH-CU458/2012"
                     /host="Homo sapiens"
                     /db_xref="taxon:208893"
                     /country="Thailand"
                     /collection_date="01-Sep-2012"
                     /note="group: A"
     CDS             <1..600
                     /note="G protein"
                     /codon_start=1
                     /product="attachment glycoprotein"
                     /protein_id="APY20319"
                     /translation="ISFSNLSGTTSQSTTILASTTPSAESTPQSTTVKIKNTTTTQIQ
                     PSKPTTKQRQNKPQNKPNNDFHFEVFNFVPCSICSNNPTCWAICKRIPNKKPGKKTTT
                     KPTKKPTIKTTKKDPKPQTTKPKEVLTTKPTEKPTIDTTKTNIRTTLLTSNTTGNPEH
                     TSQEETLHSTTSEGNLSPSQVYTTSEYLSQSPSSSNTSK"
ORIGIN
        1 atcagcttct ccaatctgtc cggaactaca tcacaatcca ccaccatact agcttcaaca
       61 acaccaagtg ctgagtcaac cccacaatcc acaacagtca agatcaaaaa cacaacaaca
```

121 acccaaatac aacctagcaa acccaccaca aaacaacgcc aaaataaacc acaaaacaaa
181 cccaacaatg attttcactt tgaagtgttc aattttgtac cctgcagcat atgcagcaac
241 aatccaacct gctgggccat ctgcaagaga ataccaaaca aaaaacctgg aaagaaaacc
301 accaccaagc ccacaaaaaa accaaccatc aagacaacca aaaaagatcc caaacctcaa
361 accacaaaac caaaggaagt actcaccacc aagcccacag aaaagccaac catcgacacc
421 accaaaacaa acatcagaac tacactgctc acctccaaca ccacaggaaa tccagaacac
481 acaagtcaag aggaaaccct ccactcaacc acctccgaag gcaatctaag cccatcacaa
541 gtctatacaa catccgagta cctatcacaa tctccatctt catccaacac atcaaaatga
//
LOCUS   KY327979   600 bp   cRNA   linear   VRL 13-DEC-2016
DEFINITION Human respiratory syncytial virus A isolate TH-CU/C2943/2012
            attachment glycoprotein gene, partial cds.
ACCESSION   KY327979
VERSION   KY327979
KEYWORDS   .
SOURCE   Human respiratory syncytial virus A
  ORGANISM Human respiratory syncytial virus A
            Viruses; ssRNA viruses; ssRNA negative-strand viruses;
            Mononegavirales; Pneumoviridae; Orthopneumovirus.
REFERENCE   1 (bases 1 to 600)
  AUTHORS   Thongpan,I., Mauleekoonphairoj,J., Vichiwattana,P., Korkong,S.,
            Vongpunsawad,S. and Poovorawan,Y.
  TITLE   Molecular Characterization of Respiratory Syncytial Virus in
            Thailand, 2012-2015
  JOURNAL   Unpublished
REFERENCE   2 (bases 1 to 600)
  AUTHORS   Thongpan,I., Mauleekoonphairoj,J., Vichiwattana,P., Korkong,S.,
            Vongpunsawad,S. and Poovorawan,Y.
  TITLE   Direct Submission
  JOURNAL   Submitted (13-DEC-2016) Department of Pediatrics, Center of
            Excellence in Clinical Virology, Faculty of Medicine, Chulalongkorn
            University, Bangkok 10330, Thailand
COMMENT   ##Assembly-Data-START##
            Assembly Method   :: DNASTAR-Lasergene v. 6
            Sequencing Technology :: Sanger dideoxy sequencing
            ##Assembly-Data-END##
FEATURES   Location/Qualifiers
     source   1..600
                     /organism="Human respiratory syncytial virus A"
                     /mol_type="viral cRNA"
                     /isolate="TH-CU/C2943/2012"
                     /host="Homo sapiens"
                     /db_xref="taxon:208893"
                     /country="Thailand"
                     /collection_date="01-Jun-2012"
                     /note="group: A"
     CDS   <1..600
                     /note="G protein"
                     /codon_start=1
                     /product="attachment glycoprotein"
                     /protein_id="APY20320"
                     /translation="ISFSNLSGTTSQSTTILASTTPSAESTPQSTTVKIKNTTTTQIQ
                     PSKPTTKQRQNKPQNKPNNDFHFEVFNFVPCSICSNNPTCWAICKRIPNKKPGKKTTT
                     KPTKKPTIKTTKKDPKPQTTKPKEVLTTKPTEKPTIDTTKTNIRTTLLTSNTTGNPEH

```
                    TSQEETLHSTTSEGNLSPSQVYTTSEYLSQSPSSSNTSK"
ORIGIN
        1 atcagcttct ccaatctgtc cggaactaca tcacaatcca ccaccatact agcttcaaca
       61 acaccaagtg ctgagtcaac cccacaatcc acaacagtca agatcaaaaa cacaacaaca
      121 acccaaatac aacctagcaa acccaccaca aaacaacgcc aaaataaacc acaaaacaaa
      181 cccaacaatg attttcactt tgaagtgttc aattttgtac cctgcagcat atgcagcaac
      241 aatccaacct gctgggccat ctgcaagaga ataccaaaca aaaaacctgg aaagaaaacc
      301 accaccaagc ccacaaaaaa accaaccatc aaaacaacca aaaaagatcc caaacctcaa
      361 accacaaaac caaaggaagt actcaccacc aagcccacag aaaagccaac catcgacacc
      421 accaaaacaa acatcagaac tacactgctc acctccaaca ccacaggaaa tccagaacac
      481 acaagtcaag aggaaaccct ccactcaacc acctccgaag gcaatctaag cccatcacaa
      541 gtctatacaa catccgagta cctatcacaa tctccatctt catccaacac atcaaaatga
//
LOCUS       KY327980                 600 bp    cRNA    linear   VRL 13-DEC-2016
DEFINITION  Human respiratory syncytial virus A isolate TH-CU438/2012
            attachment glycoprotein gene, partial cds.
ACCESSION   KY327980
VERSION     KY327980
KEYWORDS    .
SOURCE      Human respiratory syncytial virus A
  ORGANISM  Human respiratory syncytial virus A
            Viruses; ssRNA viruses; ssRNA negative-strand viruses;
            Mononegavirales; Pneumoviridae; Orthopneumovirus.
REFERENCE   1  (bases 1 to 600)
  AUTHORS   Thongpan,I., Mauleekoonphairoj,J., Vichiwattana,P., Korkong,S.,
            Vongpunsawad,S. and Poovorawan,Y.
  TITLE     Molecular Characterization of Respiratory Syncytial Virus in
            Thailand, 2012-2015
  JOURNAL   Unpublished
REFERENCE   2  (bases 1 to 600)
  AUTHORS   Thongpan,I., Mauleekoonphairoj,J., Vichiwattana,P., Korkong,S.,
            Vongpunsawad,S. and Poovorawan,Y.
  TITLE     Direct Submission
  JOURNAL   Submitted (13-DEC-2016) Department of Pediatrics, Center of
            Excellence in Clinical Virology, Faculty of Medicine, Chulalongkorn
            University, Bangkok 10330, Thailand
COMMENT     ##Assembly-Data-START##
            Assembly Method       :: DNASTAR-Lasergene v. 6
            Sequencing Technology :: Sanger dideoxy sequencing
            ##Assembly-Data-END##
FEATURES             Location/Qualifiers
     source          1..600
                     /organism="Human respiratory syncytial virus A"
                     /mol_type="viral cRNA"
                     /isolate="TH-CU438/2012"
                     /host="Homo sapiens"
                     /db_xref="taxon:208893"
                     /country="Thailand"
                     /collection_date="01-Sep-2012"
                     /note="group: A"
     CDS             <1..600
                     /note="G protein"
                     /codon_start=1
                     /product="attachment glycoprotein"
```

```
                     /protein_id="APY20321"
                     /translation="ISFTNLSGTTSKSTTILASTTPSAESTPQSTTVKIRNTTTTQIQ
                     PSKPTTKQRQNKPQNKPNNDFHFEVFNFVPCSICSNNPTCWAICKRIPNKKPGKKTTT
                     KPTKKPTIKTTKKDPKPQTTKPKEVLTTKLTEKPTIGTTKTNIRTTLLTSNTTGNPEH
                     TSQEETLHSTTSEGNLSPSQVYTTSEYLSQSPSSSNTTK"
ORIGIN
        1 atcagcttca ccaatctgtc cggaactaca tcaaaatcca ccaccatact agcttcaaca
       61 acaccaagtg ctgagtcaac ccccacaatcc acaacagtca agatcagaaa cacaacaaca
      121 acccaaatac aacctagcaa acccaccaca aaacaacgcc aaaataaacc acaaaacaaa
      181 cccaacaacg attttcactt tgaagtgttc aattttgtac cctgcagcat atgcagcaac
      241 aatccaacct gctgggccat ctgcaagaga ataccaaaca aaaaacctgg aaagaaaacc
      301 accaccaagc ccacaaaaaa accaaccatc aagacaacca aaaaagatcc caaacctcaa
      361 accacaaaac caaaggaagt actcaccacc aagctcacag aaaagccaac catcggcacc
      421 accaaaacaa acatcagaac tacactgctc acctccaaca ccacaggaaa tccagaacac
      481 acaagtcaag aggaaaccct ccactcaacc acctccgaag gcaatctaag cccatcacaa
      541 gtctatacaa catccgagta cctatcacaa tctccatctt catccaacac aacaaaatga
//
LOCUS       KY327981                 600 bp    cRNA    linear   VRL 13-DEC-2016
DEFINITION  Human respiratory syncytial virus A isolate TH-CU463/2012
            attachment glycoprotein gene, partial cds.
ACCESSION   KY327981
VERSION     KY327981
KEYWORDS    .
SOURCE      Human respiratory syncytial virus A
  ORGANISM  Human respiratory syncytial virus A
            Viruses; ssRNA viruses; ssRNA negative-strand viruses;
            Mononegavirales; Pneumoviridae; Orthopneumovirus.
REFERENCE   1  (bases 1 to 600)
  AUTHORS   Thongpan,I., Mauleekoonphairoj,J., Vichiwattana,P., Korkong,S.,
            Vongpunsawad,S. and Poovorawan,Y.
  TITLE     Molecular Characterization of Respiratory Syncytial Virus in
            Thailand, 2012-2015
  JOURNAL   Unpublished
REFERENCE   2  (bases 1 to 600)
  AUTHORS   Thongpan,I., Mauleekoonphairoj,J., Vichiwattana,P., Korkong,S.,
            Vongpunsawad,S. and Poovorawan,Y.
  TITLE     Direct Submission
  JOURNAL   Submitted (13-DEC-2016) Department of Pediatrics, Center of
            Excellence in Clinical Virology, Faculty of Medicine, Chulalongkorn
            University, Bangkok 10330, Thailand
COMMENT     ##Assembly-Data-START##
            Assembly Method       :: DNASTAR-Lasergene v. 6
            Sequencing Technology :: Sanger dideoxy sequencing
            ##Assembly-Data-END##
FEATURES             Location/Qualifiers
     source          1..600
                     /organism="Human respiratory syncytial virus A"
                     /mol_type="viral cRNA"
                     /isolate="TH-CU463/2012"
                     /host="Homo sapiens"
                     /db_xref="taxon:208893"
                     /country="Thailand"
                     /collection_date="01-Sep-2012"
                     /note="group: A"
```

```
     CDS             <1..600
                     /note="G protein"
                     /codon_start=1
                     /product="attachment glycoprotein"
                     /protein_id="APY20322"
                     /translation="ISFTNLSGTTSKSTTILASTTPSAESTPQSTTVKIRNTTTTQIQ
                     PSKPTTKQRQNKPQNKPNNDFHFEVFNFVPCSICSNNPTCWAICKRIPNKKPGKKTTT
                     KPTKKPTIKTTKKDPKPQTTKPKEVLTTKLTEKPTIGTTKTNIRTTLLTSNTTGNPEH
                     TSQEETLHSTTSEGNLSPSQVYTTSEYLSQSPSSSNTTK"
ORIGIN
        1 atcagcttca ccaatctgtc cggaactaca tcaaaatcca ccaccatact agcttcaaca
       61 acaccaagtg ctgagtcaac ccccacaatcc acaacagtca agatcagaaa cacaacaaca
      121 acccaaatac aacctagcaa acccaccaca aaacaacgcc aaaataaacc acaaaacaaa
      181 cccaacaacg attttcactt tgaagtgttc aattttgtac cctgcagcat atgcagcaac
      241 aatccaacct gctgggccat ctgcaagaga ataccaaaca aaaaacctgg aaagaaaacc
      301 accaccaagc ccacaaaaaa accaaccatc aagacaacca aaaaagatcc caaacctcaa
      361 accacaaaac caaaggaagt actcaccacc aagctcacag aaaagccaac catcggcacc
      421 accaaaacaa acatcagaac tacactgctc acctccaaca ccacaggaaa tccagaacac
      481 acaagtcaag aggaaaccct ccactcaacc acctccgaag gcaatctaag cccatcacaa
      541 gtctatacaa catccgagta cctatcacaa tctccatctt catccaacac aacaaaatga
//
LOCUS       KY327982                 600 bp    cRNA    linear   VRL 13-DEC-2016
DEFINITION  Human respiratory syncytial virus A isolate TH-CU/C3108/2012
            attachment glycoprotein gene, partial cds.
ACCESSION   KY327982
VERSION     KY327982
KEYWORDS    .
SOURCE      Human respiratory syncytial virus A
  ORGANISM  Human respiratory syncytial virus A
            Viruses; ssRNA viruses; ssRNA negative-strand viruses;
            Mononegavirales; Pneumoviridae; Orthopneumovirus.
REFERENCE   1  (bases 1 to 600)
  AUTHORS   Thongpan,I., Mauleekoonphairoj,J., Vichiwattana,P., Korkong,S.,
            Vongpunsawad,S. and Poovorawan,Y.
  TITLE     Molecular Characterization of Respiratory Syncytial Virus in
            Thailand, 2012-2015
  JOURNAL   Unpublished
REFERENCE   2  (bases 1 to 600)
  AUTHORS   Thongpan,I., Mauleekoonphairoj,J., Vichiwattana,P., Korkong,S.,
            Vongpunsawad,S. and Poovorawan,Y.
  TITLE     Direct Submission
  JOURNAL   Submitted (13-DEC-2016) Department of Pediatrics, Center of
            Excellence in Clinical Virology, Faculty of Medicine, Chulalongkorn
            University, Bangkok 10330, Thailand
COMMENT     ##Assembly-Data-START##
            Assembly Method       :: DNASTAR-Lasergene v. 6
            Sequencing Technology :: Sanger dideoxy sequencing
            ##Assembly-Data-END##
FEATURES             Location/Qualifiers
     source          1..600
                     /organism="Human respiratory syncytial virus A"
                     /mol_type="viral cRNA"
                     /isolate="TH-CU/C3108/2012"
                     /host="Homo sapiens"
```

```
                 /db_xref="taxon:208893"
                 /country="Thailand"
                 /collection_date="16-Aug-2012"
                 /note="group: A"
     CDS             <1..600
                 /note="G protein"
                 /codon_start=1
                 /product="attachment glycoprotein"
                 /protein_id="APY20323"
                 /translation="ISFSNLSETTSQSTTILASTTPSAEPTPQSTTVKIKNTTTTQIQ
                 PSKPTTKQRQNKPQNKPNNDFHFEVFNFVPCSICSNNPTCWAICKRIPNKKPGKKTTT
                 KPTKKPTIKTTKKDPKPQTTKPKEVLTTKPTEKPTIYTTKTNIRTTLLTSNTTGNPEH
                 TSQEETLHSTTSEGNLSPSQVYTTSEYLSQSPSSSNTSK"
ORIGIN
        1 atcagcttct ccaatctgtc cgaaactaca tcacaatcca ccaccatact agcttcaaca
       61 acaccaagtg ctgagccaac cccacaatcc acaacagtca agatcaaaaa cacaacaaca
      121 acccaaatac aacctagcaa acccaccaca aaacaacgcc aaaacaaacc acaaaacaaa
      181 cccaacaatg attttcactt tgaagtgttc aattttgtac cctgtagcat atgcagcaac
      241 aatccaacct gctgggccat ctgcaagaga ataccaaaca aaaaacctgg aaagaaaacc
      301 accaccaagc ccacaaaaaa accaaccatc aagacaacca aaaaagatcc caaacctcaa
      361 accacaaaac caaaggaagt actcaccacc aagcccacag aaaagccaac catctacacc
      421 accaaaacaa acatcagaac tacactgctc acctccaaca ccacaggaaa tccagaacac
      481 acaagtcaag aggaaaccct ccactcaacc acctccgaag gcaatctaag cccatcacaa
      541 gtctatacaa catccgagta cctatcacaa tctccatctt catccaacac atcaaaatga
//
LOCUS       KY327983                 306 bp    cRNA    linear   VRL 13-DEC-2016
DEFINITION  Human respiratory syncytial virus A isolate TH-CU346/2012
            attachment glycoprotein gene, partial cds.
ACCESSION   KY327983
VERSION     KY327983
KEYWORDS    .
SOURCE      Human respiratory syncytial virus A
  ORGANISM  Human respiratory syncytial virus A
            Viruses; ssRNA viruses; ssRNA negative-strand viruses;
            Mononegavirales; Pneumoviridae; Orthopneumovirus.
REFERENCE   1  (bases 1 to 306)
  AUTHORS   Thongpan,I., Mauleekoonphairoj,J., Vichiwattana,P., Korkong,S.,
            Vongpunsawad,S. and Poovorawan,Y.
  TITLE     Molecular Characterization of Respiratory Syncytial Virus in
            Thailand, 2012-2015
  JOURNAL   Unpublished
REFERENCE   2  (bases 1 to 306)
  AUTHORS   Thongpan,I., Mauleekoonphairoj,J., Vichiwattana,P., Korkong,S.,
            Vongpunsawad,S. and Poovorawan,Y.
  TITLE     Direct Submission
  JOURNAL   Submitted (13-DEC-2016) Department of Pediatrics, Center of
            Excellence in Clinical Virology, Faculty of Medicine, Chulalongkorn
            University, Bangkok 10330, Thailand
COMMENT     ##Assembly-Data-START##
            Assembly Method       :: DNASTAR-Lasergene v. 6
            Sequencing Technology :: Sanger dideoxy sequencing
            ##Assembly-Data-END##
FEATURES             Location/Qualifiers
     source          1..306
```

/organism="Human respiratory syncytial virus A"
/mol_type="viral cRNA"
/isolate="TH-CU346/2012"
/host="Homo sapiens"
/db_xref="taxon:208893"
/country="Thailand"
/collection_date="01-Apr-2012"
/note="group: A"
CDS <1..306
/note="G protein"
/codon_start=1
/product="attachment glycoprotein"
/protein_id="APY20324"
/translation="KTTTKPTKKPTLKTTKKDPKPQTTKPKEVLTTKPTEKPTIDTTK
TNIRTTLLTSNTTGNPEHTSQEETLHSTTSEGNLSPSQVYTTSEYLSQSPSSSNTSK"
ORIGIN
1 aaaaccacca ccaagcccac aaaaaagcca accctcaaga caaccaaaaa agatcccaaa
61 cctcaaacca caaaaccaaa ggaagtactc accaccaagc ccacagaaaa gccaaccatc
121 gacaccacca aaacaaacat cagaactaca ctgctcacct ccaacaccac aggaaatcca
181 gaacacacaa gtcaagagga aaccctccac tcaaccacct ccgaaggcaa tctaagccca
241 tcacaagtct atacaacatc cgagtaccta tcacaatctc catcttcatc caacacatca
301 aaatga
//
LOCUS KY327984 393 bp cRNA linear VRL 13-DEC-2016
DEFINITION Human respiratory syncytial virus A isolate TH-CU363/2012
attachment glycoprotein gene, partial cds.
ACCESSION KY327984
VERSION KY327984
KEYWORDS .
SOURCE Human respiratory syncytial virus A
ORGANISM Human respiratory syncytial virus A
Viruses; ssRNA viruses; ssRNA negative-strand viruses;
Mononegavirales; Pneumoviridae; Orthopneumovirus.
REFERENCE 1 (bases 1 to 393)
AUTHORS Thongpan,I., Mauleekoonphairoj,J., Vichiwattana,P., Korkong,S.,
Vongpunsawad,S. and Poovorawan,Y.
TITLE Molecular Characterization of Respiratory Syncytial Virus in
Thailand, 2012-2015
JOURNAL Unpublished
REFERENCE 2 (bases 1 to 393)
AUTHORS Thongpan,I., Mauleekoonphairoj,J., Vichiwattana,P., Korkong,S.,
Vongpunsawad,S. and Poovorawan,Y.
TITLE Direct Submission
JOURNAL Submitted (13-DEC-2016) Department of Pediatrics, Center of
Excellence in Clinical Virology, Faculty of Medicine, Chulalongkorn
University, Bangkok 10330, Thailand
COMMENT ##Assembly-Data-START##
Assembly Method :: DNASTAR-Lasergene v. 6
Sequencing Technology :: Sanger dideoxy sequencing
##Assembly-Data-END##
FEATURES Location/Qualifiers
source 1..393
/organism="Human respiratory syncytial virus A"
/mol_type="viral cRNA"

```
                     /isolate="TH-CU363/2012"
                     /host="Homo sapiens"
                     /db_xref="taxon:208893"
                     /country="Thailand"
                     /collection_date="01-Jun-2012"
                     /note="group: A"
     CDS             <1..393
                     /note="G protein"
                     /codon_start=1
                     /product="attachment glycoprotein"
                     /protein_id="APY20325"
                     /translation="FNFVPCSICSNNPTCWAICKRIPNKKPGKKTTTKPTKKPTIKTT
                     KKDPKPQTTKPKEVLTTKPTEKPTIDTTKTNIRTTLLTSYTTGNPEHTSQEETLHSTT
                     SEGNLSPSQVYTTSEYLSQSPSSSYTTK"
ORIGIN
        1 ttcaactttg taccctgcag catatgcagc aacaatccaa cctgctgggc tatctgcaag
       61 agaataccaa acaaaaaacc tggaaagaaa accaccacca agcccacaaa aaaaccaacc
      121 atcaagacaa ccaaaaaaga tcccaaacct caaaccacaa aaccaaagga ggtactcacc
      181 accaagccca cagaaaagcc aaccatcgac accaccaaaa caaacatcag aactacactg
      241 ctcacctcct acactacagg aaatccagaa cacacaagcc aagaggaaac cctccactca
      301 accacctccg aaggcaatct aagcccatca caagtctata caacatccga gtacctatca
      361 caatctccat cttcatccta cacaacaaaa tga
//
LOCUS       KY327985                 660 bp    cRNA    linear   VRL 13-DEC-2016
DEFINITION  Human respiratory syncytial virus A isolate TH-CU432/2012
            attachment glycoprotein gene, partial cds.
ACCESSION   KY327985
VERSION     KY327985
KEYWORDS    .
SOURCE      Human respiratory syncytial virus A
  ORGANISM  Human respiratory syncytial virus A
            Viruses; ssRNA viruses; ssRNA negative-strand viruses;
            Mononegavirales; Pneumoviridae; Orthopneumovirus.
REFERENCE   1  (bases 1 to 660)
  AUTHORS   Thongpan,I., Mauleekoonphairoj,J., Vichiwattana,P., Korkong,S.,
            Vongpunsawad,S. and Poovorawan,Y.
  TITLE     Molecular Characterization of Respiratory Syncytial Virus in
            Thailand, 2012-2015
  JOURNAL   Unpublished
REFERENCE   2  (bases 1 to 660)
  AUTHORS   Thongpan,I., Mauleekoonphairoj,J., Vichiwattana,P., Korkong,S.,
            Vongpunsawad,S. and Poovorawan,Y.
  TITLE     Direct Submission
  JOURNAL   Submitted (13-DEC-2016) Department of Pediatrics, Center of
            Excellence in Clinical Virology, Faculty of Medicine, Chulalongkorn
            University, Bangkok 10330, Thailand
COMMENT     ##Assembly-Data-START##
            Assembly Method       :: DNASTAR-Lasergene v. 6
            Sequencing Technology :: Sanger dideoxy sequencing
            ##Assembly-Data-END##
FEATURES             Location/Qualifiers
     source          1..660
                     /organism="Human respiratory syncytial virus A"
                     /mol_type="viral cRNA"
```

/isolate="TH-CU432/2012"
/host="Homo sapiens"
/db_xref="taxon:208893"
/country="Thailand"
/collection_date="01-Aug-2012"
/note="group: A"
CDS <1..660
/note="G protein"
/codon_start=1
/product="attachment glycoprotein"
/protein_id="APY20326"
/translation="ATSQIKNTTPTYLTQNPQLGISFSNLSGTTSQSTIILASTTPSA
ESTPQSTTVKIKNTTTTQIQPSKPTTKQRQNKPQNKPNNDFHFEVFNFVPCSICSNNP
TCWAICKRIPNKKPGKKTTTKPTKKPTIKTTKKDPKPQTTKPKEVLTTKPTEQPTINT
TKTNIRTTLLTSNTTGDPEHTSQEETLHSTTFEGNPSPSQVHTTPEYPSQSPSSSNTT
K"
ORIGIN
1 gcaacaagcc agatcaagaa cacaacccca acatacctca cccagaatcc ccagcttgga
61 atcagcttct ccaatctgtc cggaactaca tcacaatcca ccatcatact agcttcaaca
121 acaccaagtg ctgagtcaac ccccacaatcc acaacagtca agatcaaaaa cacaacaaca
181 acccaaatac aacctagcaa acccaccaca aaacaacgcc aaaataaacc acaaaacaaa
241 cccaacaatg attttcactt cgaagtgttc aattttgtac cctgcagcat atgcagcaac
301 aatccaacct gctgggccat ctgcaagaga ataccaaaca aaaaacctgg aaagaaaacc
361 accaccaagc ccacaaaaaa accaaccatc aagacaacca aaaaagatcc caaacctcaa
421 accacaaaac caaaggaagt actcaccacc aagcccacag aacagccaac catcaacacc
481 accaaaacaa acatcagaac cacactgctc acctccaaca ccacaggaga tccagaacac
541 acaagtcaag aggaaactct ccactcaacc accttcgaag gcaatccaag cccatcacaa
601 gtccacacaa cacccgagta cccatcacaa tctccatcct catctaacac aacaaaatga
//
LOCUS KY327986 651 bp cRNA linear VRL 12-DEC-2016
DEFINITION Human respiratory syncytial virus A isolate TH-CU/B10625/2014
attachment glycoprotein gene, partial cds.
ACCESSION KY327986
VERSION KY327986
KEYWORDS .
SOURCE Human respiratory syncytial virus A
ORGANISM Human respiratory syncytial virus A
Viruses; ssRNA viruses; ssRNA negative-strand viruses;
Mononegavirales; Pneumoviridae; Orthopneumovirus.
REFERENCE 1 (bases 1 to 651)
AUTHORS Thongpan,I., Mauleekoonphairoj,J., Vichiwattana,P., Korkong,S.,
Vongpunsawad,S. and Poovorawan,Y.
TITLE Molecular Characterization of Respiratory Syncytial Virus in
Thailand, 2012-2015
JOURNAL Unpublished
REFERENCE 2 (bases 1 to 651)
AUTHORS Thongpan,I., Mauleekoonphairoj,J., Vichiwattana,P., Korkong,S.,
Vongpunsawad,S. and Poovorawan,Y.
TITLE Direct Submission
JOURNAL Submitted (13-DEC-2016) Department of Pediatrics, Center of
Excellence in Clinical Virology, Faculty of Medicine, Chulalongkorn
University, Bangkok 10330, Thailand
COMMENT ##Assembly-Data-START##
Assembly Method :: DNASTAR-Lasergene v. 6

```
            Sequencing Technology :: Sanger dideoxy sequencing
            ##Assembly-Data-END##
FEATURES             Location/Qualifiers
     source          1..651
                     /organism="Human respiratory syncytial virus A"
                     /mol_type="viral cRNA"
                     /isolate="TH-CU/B10625/2014"
                     /host="Homo sapiens"
                     /db_xref="taxon:208893"
                     /country="Thailand"
                     /collection_date="09-Aug-2014"
                     /note="group: A"
     CDS             <1..651
                     /note="G protein"
                     /codon_start=1
                     /product="attachment glycoprotein"
                     /protein_id="APY20327"
                     /translation="GTTSQSTTILASTTPSAESTPQSTTVKIKNTTTTQILPSKTTTK
                     QHQNKPQNKPNNDFHFEVFNFVPCSICSNNPTCWAICKRIPNKKPGKKTTTKPTKKPT
                     LKTTKKDPKPQTTKPKEVLTTKPTGKPTINTTKTNIRTTLLTSNTKGNPEHTSQEETL
                     HSTTSEGYLSPSQVYTTSGQEETLHSTTSEGYLSPSQVYTTSEYLSQSLSSSNATK"
ORIGIN
        1 ggaactacat cacaatccac caccatacta gcttcaacaa caccaagtgc tgagtcaacc
       61 ccacaatcca caacagtcaa gatcaaaaac acaacaacaa cccaaatatt acctagcaaa
      121 accaccacaa aacaacacca aaataaacca caaaacaaac ccaacaatga ttttcacttt
      181 gaagtgttca attttgtacc ctgcagcata tgcagcaaca atccaacctg ctgggccatc
      241 tgcaagagaa taccaaacaa aaaacctgga aagaaaacca ccaccaagcc cacaaaaaaa
      301 ccaaccctca aaacaaccaa aaaagatccc aaacctcaaa ccacaaaacc aaaggaagta
      361 ctcactacca agcctacagg aaagccaacc atcaacacca ctaaaacaaa catcagaact
      421 acactgctca cctccaacac caaaggaaat ccagaacaca caagtcaaga ggaaaccctc
      481 cactcaacca cctccgaagg ctatctaagc ccatcacaag tctatacaac atccggtcaa
      541 gaggaaaccc tccactcaac cacctccgaa ggttatctaa gcccatcaca agtctataca
      601 acatccgagt acctatcaca atctctatct tcatccaacg caacaaaatg a
//
LOCUS       KY327987                 651 bp    cRNA    linear   VRL 12-DEC-2016
DEFINITION  Human respiratory syncytial virus A isolate TH-CU/C3083/2012
            attachment glycoprotein gene, partial cds.
ACCESSION   KY327987
VERSION     KY327987
KEYWORDS    .
SOURCE      Human respiratory syncytial virus A
  ORGANISM  Human respiratory syncytial virus A
            Viruses; ssRNA viruses; ssRNA negative-strand viruses;
            Mononegavirales; Pneumoviridae; Orthopneumovirus.
REFERENCE   1  (bases 1 to 651)
  AUTHORS   Thongpan,I., Mauleekoonphairoj,J., Vichiwattana,P., Korkong,S.,
            Vongpunsawad,S. and Poovorawan,Y.
  TITLE     Molecular Characterization of Respiratory Syncytial Virus in
            Thailand, 2012-2015
  JOURNAL   Unpublished
REFERENCE   2  (bases 1 to 651)
  AUTHORS   Thongpan,I., Mauleekoonphairoj,J., Vichiwattana,P., Korkong,S.,
            Vongpunsawad,S. and Poovorawan,Y.
  TITLE     Direct Submission
```

```
  JOURNAL   Submitted (13-DEC-2016) Department of Pediatrics, Center of
            Excellence in Clinical Virology, Faculty of Medicine, Chulalongkorn
            University, Bangkok 10330, Thailand
COMMENT     ##Assembly-Data-START##
            Assembly Method       :: DNASTAR-Lasergene v. 6
            Sequencing Technology :: Sanger dideoxy sequencing
            ##Assembly-Data-END##
FEATURES             Location/Qualifiers
     source          1..651
                     /organism="Human respiratory syncytial virus A"
                     /mol_type="viral cRNA"
                     /isolate="TH-CU/C3083/2012"
                     /host="Homo sapiens"
                     /db_xref="taxon:208893"
                     /country="Thailand"
                     /collection_date="16-Aug-2012"
                     /note="group: A"
     CDS             <1..651
                     /note="G protein"
                     /codon_start=1
                     /product="attachment glycoprotein"
                     /protein_id="APY20328"
                     /translation="GTTSQSTTILASTTPSAESTPQSTTVKIKNTTTTQILPSKPTTK
                     QRQNKPQNKPNNDFHFEVFNFVPCSICSNNPTCWAICKRIPNKKPGKKTTTKPTKKPT
                     LKTTKKDPKPQTTKPKEVLTTKPTGKPTINTTKTNIRTTLLTSNTKGNPEHTSQEETL
                     HSTTSEGYLSPSQVYTTSGQEETLHSTTSEGYLSPSQVYTTSEYLSQSLSSSNTTK"
ORIGIN
        1 ggaactacat cacaatccac caccatacta gcttcaacaa caccaagtgc tgagtcaacc
       61 ccacaatcca caacagtcaa gatcaaaaac acaacaacaa cccaaatatt acctagcaaa
      121 cccaccacaa aacaacgcca aaataaacca caaaacaaac ccaacaatga ttttcacttt
      181 gaagtgttca attttgtacc ctgcagcata tgcagcaaca atccaacctg ctgggccatc
      241 tgcaagagaa taccaaacaa aaaacctgga aagaaaacca ccaccaagcc cacaaaaaaa
      301 ccaaccctca agacaaccaa aaaagatccc aaacctcaaa ccacaaaacc aaaggaagta
      361 ctcactacca agcctacagg aaagccaacc atcaacacca ctaaaacaaa catcagaact
      421 acactgctca cctccaacac caaaggaaat ccagaacaca caagtcaaga ggaaaccctc
      481 cactcaacca cctccgaagg ctatctaagc ccatcacaag tctatacaac atctggtcaa
      541 gaggaaaccc tccactcaac cacctccgaa ggctatctaa gcccatcaca agtctataca
      601 acatccgagt acctatcaca atctctatct tcatccaaca caacaaaatg a
//
LOCUS       KY327988                 651 bp    cRNA    linear   VRL 12-DEC-2016
DEFINITION  Human respiratory syncytial virus A isolate TH-CU/C3142/2012
            attachment glycoprotein gene, partial cds.
ACCESSION   KY327988
VERSION     KY327988
KEYWORDS    .
SOURCE      Human respiratory syncytial virus A
  ORGANISM  Human respiratory syncytial virus A
            Viruses; ssRNA viruses; ssRNA negative-strand viruses;
            Mononegavirales; Pneumoviridae; Orthopneumovirus.
REFERENCE   1  (bases 1 to 651)
  AUTHORS   Thongpan,I., Mauleekoonphairoj,J., Vichiwattana,P., Korkong,S.,
            Vongpunsawad,S. and Poovorawan,Y.
  TITLE     Molecular Characterization of Respiratory Syncytial Virus in
            Thailand, 2012-2015
```

```
  JOURNAL   Unpublished
REFERENCE   2  (bases 1 to 651)
  AUTHORS   Thongpan,I., Mauleekoonphairoj,J., Vichiwattana,P., Korkong,S.,
            Vongpunsawad,S. and Poovorawan,Y.
  TITLE     Direct Submission
  JOURNAL   Submitted (13-DEC-2016) Department of Pediatrics, Center of
            Excellence in Clinical Virology, Faculty of Medicine, Chulalongkorn
            University, Bangkok 10330, Thailand
COMMENT     ##Assembly-Data-START##
            Assembly Method       :: DNASTAR-Lasergene v. 6
            Sequencing Technology :: Sanger dideoxy sequencing
            ##Assembly-Data-END##
FEATURES             Location/Qualifiers
     source          1..651
                     /organism="Human respiratory syncytial virus A"
                     /mol_type="viral cRNA"
                     /isolate="TH-CU/C3142/2012"
                     /host="Homo sapiens"
                     /db_xref="taxon:208893"
                     /country="Thailand"
                     /collection_date="22-Aug-2012"
                     /note="group: A"
     CDS             <1..651
                     /note="G protein"
                     /codon_start=1
                     /product="attachment glycoprotein"
                     /protein_id="APY20329"
                     /translation="GTTSQSTTILASTTPSAESTPQSTTVKIKNTTTTQILPSKPTTK
                     QRQNKPQNKPNNDFHFEVFNFVPCSICSNNPTCWAICKRIPNKKPGKKTTTKPTKKPT
                     LKTTKKDPKPQTTKPKEVLTTKPTGKPTINTTKTNIRTTLLTSNTKGNPEHTSQEETL
                     HSTTSEGYLSPSQVYTTSGQEETLHSTTSEGYLSPSQVYTTSEYLSQSLSSSNTTK"
ORIGIN
        1 ggaactacat cacaatccac caccatacta gcttcaacaa caccaagtgc tgagtcaacc
       61 ccacaatcca caacagtcaa gatcaaaaac acaacaacaa cccaaatatt acctagcaaa
      121 cccaccacaa aacaacgcca aaataaacca caaaacaaac ccaacaatga ttttcacttt
      181 gaagtgttca attttgtacc ctgcagcata tgcagcaaca atccaacctg ctgggccatc
      241 tgcaagagaa taccaaacaa aaaacctgga aagaaaacca ccaccaagcc cacaaaaaaa
      301 ccaaccctca agacaaccaa aaaagatccc aaacctcaaa ccacaaaacc aaaggaagta
      361 ctcactacca agcctacagg aaagccaacc atcaacacca ctaaaacaaa catcagaact
      421 acactgctca cctccaacac caaaggaaat ccagaacaca caagtcaaga ggaaaccctc
      481 cactcaacca cctccgaagg ctatctaagc ccatcacaag tctatacaac atccggtcaa
      541 gaggaaaccc tccactcaac cacctccgaa ggctatctaa gcccatcaca agtctataca
      601 acatccgagt acctatcaca atctctatct tcatccaaca caacaaaatg a
//
LOCUS       KY327989                 651 bp    cRNA    linear   VRL 12-DEC-2016
DEFINITION  Human respiratory syncytial virus A isolate TH-CU/C3304/2012
            attachment glycoprotein gene, partial cds.
ACCESSION   KY327989
VERSION     KY327989
KEYWORDS    .
SOURCE      Human respiratory syncytial virus A
  ORGANISM  Human respiratory syncytial virus A
            Viruses; ssRNA viruses; ssRNA negative-strand viruses;
            Mononegavirales; Pneumoviridae; Orthopneumovirus.
```

```
REFERENCE   1  (bases 1 to 651)
  AUTHORS   Thongpan,I., Mauleekoonphairoj,J., Vichiwattana,P., Korkong,S.,
            Vongpunsawad,S. and Poovorawan,Y.
  TITLE     Molecular Characterization of Respiratory Syncytial Virus in
            Thailand, 2012-2015
  JOURNAL   Unpublished
REFERENCE   2  (bases 1 to 651)
  AUTHORS   Thongpan,I., Mauleekoonphairoj,J., Vichiwattana,P., Korkong,S.,
            Vongpunsawad,S. and Poovorawan,Y.
  TITLE     Direct Submission
  JOURNAL   Submitted (13-DEC-2016) Department of Pediatrics, Center of
            Excellence in Clinical Virology, Faculty of Medicine, Chulalongkorn
            University, Bangkok 10330, Thailand
COMMENT     ##Assembly-Data-START##
            Assembly Method       :: DNASTAR-Lasergene v. 6
            Sequencing Technology :: Sanger dideoxy sequencing
            ##Assembly-Data-END##
FEATURES             Location/Qualifiers
     source          1..651
                     /organism="Human respiratory syncytial virus A"
                     /mol_type="viral cRNA"
                     /isolate="TH-CU/C3304/2012"
                     /host="Homo sapiens"
                     /db_xref="taxon:208893"
                     /country="Thailand"
                     /collection_date="25-Oct-2012"
                     /note="group: A"
     CDS             <1..651
                     /note="G protein"
                     /codon_start=1
                     /product="attachment glycoprotein"
                     /protein_id="APY20330"
                     /translation="GTTSQSTTILASTTPSAESTPQSTTVKIKNTTTTQILPSKPTTK
                     QRQNKPQNKPNNDFHFEVFNFVPCSICSNNPTCWAICKRIPNKKPGKKTTTKPTKKPT
                     LKTTKKDPKPQTTKPKEVLTTKPTGKPTINTTKTNIRTTLLTSNTKGNPEHTSQEETL
                     HSTTSEGYLSPSQVYTTSGQEETLHSTTSEGYLSPSQVYTTSEYLSQSLSSSNTTK"
ORIGIN
        1 ggaactacat cacaatccac caccatacta gcttcaacaa caccaagtgc tgagtcaacc
       61 ccacaatcca caacagtcaa gatcaaaaac acaacaacaa cccaaatatt acctagcaaa
      121 cccaccacaa aacaacgcca aaataaacca caaaacaaac ccaacaatga ttttcacttt
      181 gaagtgttca attttgtacc ctgcagcata tgcagcaaca atccaacctg ctgggccatc
      241 tgcaagagaa taccaaacaa aaaacctgga aagaaaacca ccaccaagcc cacaaaaaaa
      301 ccaaccctca agacaaccaa aaaagatccc aaacctcaaa ccacaaaacc aaaggaagta
      361 ctcactacca agcctacagg aaagccaacc atcaacacca ctaaaacaaa catcagaact
      421 acactgctca cctccaacac caaaggaaat ccagaacaca caagtcaaga ggaaaccctc
      481 cactcaacca cctccgaagg ctatctaagc ccatcacaag tctatacaac atccggtcaa
      541 gaggaaaccc tccactcaac cacctccgaa ggctatctaa gcccatcaca agtctataca
      601 acatccgagt acctatcaca atctctatct tcatccaaca caacaaaatg a
//
LOCUS       KY327990                 651 bp    cRNA    linear   VRL 12-DEC-2016
DEFINITION  Human respiratory syncytial virus A isolate TH-CU/C2860/2012
            attachment glycoprotein gene, partial cds.
ACCESSION   KY327990
VERSION     KY327990
```

```
KEYWORDS    .
SOURCE      Human respiratory syncytial virus A
  ORGANISM  Human respiratory syncytial virus A
            Viruses; ssRNA viruses; ssRNA negative-strand viruses;
            Mononegavirales; Pneumoviridae; Orthopneumovirus.
REFERENCE   1  (bases 1 to 651)
  AUTHORS   Thongpan,I., Mauleekoonphairoj,J., Vichiwattana,P., Korkong,S.,
            Vongpunsawad,S. and Poovorawan,Y.
  TITLE     Molecular Characterization of Respiratory Syncytial Virus in
            Thailand, 2012-2015
  JOURNAL   Unpublished
REFERENCE   2  (bases 1 to 651)
  AUTHORS   Thongpan,I., Mauleekoonphairoj,J., Vichiwattana,P., Korkong,S.,
            Vongpunsawad,S. and Poovorawan,Y.
  TITLE     Direct Submission
  JOURNAL   Submitted (13-DEC-2016) Department of Pediatrics, Center of
            Excellence in Clinical Virology, Faculty of Medicine, Chulalongkorn
            University, Bangkok 10330, Thailand
COMMENT     ##Assembly-Data-START##
            Assembly Method       :: DNASTAR-Lasergene v. 6
            Sequencing Technology :: Sanger dideoxy sequencing
            ##Assembly-Data-END##
FEATURES             Location/Qualifiers
     source          1..651
                     /organism="Human respiratory syncytial virus A"
                     /mol_type="viral cRNA"
                     /isolate="TH-CU/C2860/2012"
                     /host="Homo sapiens"
                     /db_xref="taxon:208893"
                     /country="Thailand"
                     /collection_date="25-May-2012"
                     /note="group: A"
     CDS             <1..651
                     /note="G protein"
                     /codon_start=1
                     /product="attachment glycoprotein"
                     /protein_id="APY20331"
                     /translation="GTTSQSTTILASTTPSAESTPQSTTVKIKNTTTTQILPSKPTTK
                     QRQNKPQNKPNNDFHFEVFNFVPCSICSNNPTCWAICKRIPNKKPGKKTTTKPTKKPT
                     LKTTKKDPKPQTTKPKEVLTTKPTGKPTINTTKTNIRTTLLTSNTKGNPEHTSQEETL
                     HSTTSEGYLSPSQVYTTSGQEETLHSTTSEGYLSPSQVYTTSEYLSQSLSSSNTTK"
ORIGIN
        1 ggaactacat cacaatccac caccatacta gcttcaacaa caccaagtgc tgagtcaacc
       61 ccacaatcca caacagtcaa gatcaaaaac acaacaacaa cccaaatatt acctagcaaa
      121 cccaccacaa aacaacgcca aaataaacca caaaacaaac ccaacaatga ttttcacttt
      181 gaagtgttca attttgtacc ctgcagcata tgcagcaaca atccaacctg ctgggccatc
      241 tgcaagagaa taccaaacaa aaaacctgga aagaaaacca ccaccaagcc cacaaaaaaa
      301 ccaaccctca agacaaccaa aaaagatccc aaacctcaaa ccacaaaacc aaaggaagta
      361 ctcactacca agcctacagg aaagccaacc atcaacacca ctaaaacaaa catcagaact
      421 acactgctca cctccaacac caaaggaaat ccagaacaca caagtcaaga ggaaaccctc
      481 cactcaacca cctccgaagg ctatctaagc ccatcacaag tctatacaac atccggtcaa
      541 gaggaaaccc tccactcaac cacctccgaa ggctatctaa gcccatcaca agtctataca
      601 acatccgagt acctatcaca atctctatct tcatccaaca caacaaaatg a
//
```

```
LOCUS       KY327991                 651 bp    cRNA    linear   VRL 12-DEC-2016
DEFINITION  Human respiratory syncytial virus A isolate TH-CU/C2993/2012
            attachment glycoprotein gene, partial cds.
ACCESSION   KY327991
VERSION     KY327991
KEYWORDS    .
SOURCE      Human respiratory syncytial virus A
  ORGANISM  Human respiratory syncytial virus A
            Viruses; ssRNA viruses; ssRNA negative-strand viruses;
            Mononegavirales; Pneumoviridae; Orthopneumovirus.
REFERENCE   1  (bases 1 to 651)
  AUTHORS   Thongpan,I., Mauleekoonphairoj,J., Vichiwattana,P., Korkong,S.,
            Vongpunsawad,S. and Poovorawan,Y.
  TITLE     Molecular Characterization of Respiratory Syncytial Virus in
            Thailand, 2012-2015
  JOURNAL   Unpublished
REFERENCE   2  (bases 1 to 651)
  AUTHORS   Thongpan,I., Mauleekoonphairoj,J., Vichiwattana,P., Korkong,S.,
            Vongpunsawad,S. and Poovorawan,Y.
  TITLE     Direct Submission
  JOURNAL   Submitted (13-DEC-2016) Department of Pediatrics, Center of
            Excellence in Clinical Virology, Faculty of Medicine, Chulalongkorn
            University, Bangkok 10330, Thailand
COMMENT     ##Assembly-Data-START##
            Assembly Method       :: DNASTAR-Lasergene v. 6
            Sequencing Technology :: Sanger dideoxy sequencing
            ##Assembly-Data-END##
FEATURES             Location/Qualifiers
     source          1..651
                     /organism="Human respiratory syncytial virus A"
                     /mol_type="viral cRNA"
                     /isolate="TH-CU/C2993/2012"
                     /host="Homo sapiens"
                     /db_xref="taxon:208893"
                     /country="Thailand"
                     /collection_date="10-Jul-2012"
                     /note="group: A"
     CDS             <1..651
                     /note="G protein"
                     /codon_start=1
                     /product="attachment glycoprotein"
                     /protein_id="APY20332"
                     /translation="GTTSQSTTILASTTPSAESTPQSTTVKIKNTTTTQILPSKPTTK
                     QRQNKPQNKPNNDFHFEVFNFVPCSICSNNPTCWAICKRIPNKKPGKKTTTKPTKKPT
                     LKTTKKDPKPQTTKPKEVLTTKPTGKPTINTTKTNIRTTLLTSNTKGNPEHTSQEETL
                     HSTTSEGYLSPSQVYTTSGQEETLHSTTSEGYLSPSQVYTTSEYLSQSLSSSNTTK"
ORIGIN
        1 ggaactacat cacaatccac caccatacta gcttcaacaa caccaagtgc tgagtcaacc
       61 ccacaatcca caacagtcaa gatcaaaaac acaacaacaa cccaaatatt acctagcaaa
      121 cccaccacaa aacaacgcca aaataaacca caaaacaaac ccaacaatga ttttcacttt
      181 gaagtgttca attttgtacc ctgcagcata tgcagcaaca atccaacctg ctgggccatc
      241 tgcaagagaa taccaaacaa aaaacctgga aagaaaacca ccaccaagcc cacaaaaaaa
      301 ccaaccctca agacaaccaa aaaagatccc aaacctcaaa ccacaaaacc aaaggaagta
      361 ctcactacca agcctacagg aaagccaacc atcaacacca ctaaaacaaa catcagaact
```

421 acactgctca cctccaacac caaaggaaat ccagaacaca caagtcaaga ggaaaccctc
481 cactcaacca cctccgaagg ctatctaagc ccatcacaag tctatacaac atccggtcaa
541 gaggaaaccc tccactcaac cacctccgaa ggctatctaa gcccatcaca agtctataca
601 acatccgagt acctatcaca atctctatct tcatccaaca caacaaaatg a
//
LOCUS KY327992 651 bp cRNA linear VRL 12-DEC-2016
DEFINITION Human respiratory syncytial virus A isolate TH-CU/C3130/2012
attachment glycoprotein gene, partial cds.
ACCESSION KY327992
VERSION KY327992
KEYWORDS .
SOURCE Human respiratory syncytial virus A
ORGANISM Human respiratory syncytial virus A
Viruses; ssRNA viruses; ssRNA negative-strand viruses;
Mononegavirales; Pneumoviridae; Orthopneumovirus.
REFERENCE 1 (bases 1 to 651)
AUTHORS Thongpan,I., Mauleekoonphairoj,J., Vichiwattana,P., Korkong,S.,
Vongpunsawad,S. and Poovorawan,Y.
TITLE Molecular Characterization of Respiratory Syncytial Virus in
Thailand, 2012-2015
JOURNAL Unpublished
REFERENCE 2 (bases 1 to 651)
AUTHORS Thongpan,I., Mauleekoonphairoj,J., Vichiwattana,P., Korkong,S.,
Vongpunsawad,S. and Poovorawan,Y.
TITLE Direct Submission
JOURNAL Submitted (13-DEC-2016) Department of Pediatrics, Center of
Excellence in Clinical Virology, Faculty of Medicine, Chulalongkorn
University, Bangkok 10330, Thailand
COMMENT ##Assembly-Data-START##
Assembly Method :: DNASTAR-Lasergene v. 6
Sequencing Technology :: Sanger dideoxy sequencing
##Assembly-Data-END##
FEATURES Location/Qualifiers
source 1..651
/organism="Human respiratory syncytial virus A"
/mol_type="viral cRNA"
/isolate="TH-CU/C3130/2012"
/host="Homo sapiens"
/db_xref="taxon:208893"
/country="Thailand"
/collection_date="22-Aug-2012"
/note="group: A"
CDS <1..651
/note="G protein"
/codon_start=1
/product="attachment glycoprotein"
/protein_id="APY20333"
/translation="GTTSQSTTILASTTPSAESTPQSTTVKIKNTTTTQILPSKPTTK
QRQNKPQNKPNNDFHFEVFNFVPCSICSNNPTCWAICKRIPNKKPGKKTTTKPTKKPT
LKTTKKDPKPQTTKPKEVLTTKPTGKPTINTTKTNIRTTLLTSNTKGNPEHTSQEETL
HSTTSEGYLSPSQVYTTSGQEETLHSTTSEGYLSPSQVYTTSEYLSQSLSSSNTTK"
ORIGIN
1 ggaactacat cacaatccac caccatacta gcttcaacaa caccaagtgc tgagtcaacc
61 ccacaatcca caacagtcaa gatcaaaaac acaacaacaa cccaaatatt acctagcaaa

```
     121 cccaccacaa aacaacgcca aaataaacca caaaacaaac ccaacaatga ttttcacttt
     181 gaagtgttca attttgtacc ctgcagcata tgcagcaaca atccaacctg ctgggccatc
     241 tgcaagagaa taccaaacaa aaaacctgga aagaaaacca ccaccaagcc cacaaaaaaa
     301 ccaaccctca agacaaccaa aaaagatccc aaacctcaaa ccacaaaacc aaaggaagta
     361 ctcactacca agcctacagg aaagccaacc atcaacacca ctaaaacaaa catcagaact
     421 acactgctca cctccaacac caaaggaaat ccagaacaca caagtcaaga ggaaaccctc
     481 cactcaacca cctccgaagg ctatctaagc ccatcacaag tctatacaac atctggtcaa
     541 gaggaaaccc tccactcaac cacctccgaa ggctatctaa gcccatcaca agtctataca
     601 acatccgagt acctatcaca atctctatct tcatccaaca caacaaaatg a
//
LOCUS       KY327993                 651 bp    cRNA    linear   VRL 12-DEC-2016
DEFINITION  Human respiratory syncytial virus A isolate TH-CU/B10623/2014
            attachment glycoprotein gene, partial cds.
ACCESSION   KY327993
VERSION     KY327993
KEYWORDS    .
SOURCE      Human respiratory syncytial virus A
  ORGANISM  Human respiratory syncytial virus A
            Viruses; ssRNA viruses; ssRNA negative-strand viruses;
            Mononegavirales; Pneumoviridae; Orthopneumovirus.
REFERENCE   1  (bases 1 to 651)
  AUTHORS   Thongpan,I., Mauleekoonphairoj,J., Vichiwattana,P., Korkong,S.,
            Vongpunsawad,S. and Poovorawan,Y.
  TITLE     Molecular Characterization of Respiratory Syncytial Virus in
            Thailand, 2012-2015
  JOURNAL   Unpublished
REFERENCE   2  (bases 1 to 651)
  AUTHORS   Thongpan,I., Mauleekoonphairoj,J., Vichiwattana,P., Korkong,S.,
            Vongpunsawad,S. and Poovorawan,Y.
  TITLE     Direct Submission
  JOURNAL   Submitted (13-DEC-2016) Department of Pediatrics, Center of
            Excellence in Clinical Virology, Faculty of Medicine, Chulalongkorn
            University, Bangkok 10330, Thailand
COMMENT     ##Assembly-Data-START##
            Assembly Method       :: DNASTAR-Lasergene v. 6
            Sequencing Technology :: Sanger dideoxy sequencing
            ##Assembly-Data-END##
FEATURES             Location/Qualifiers
     source          1..651
                     /organism="Human respiratory syncytial virus A"
                     /mol_type="viral cRNA"
                     /isolate="TH-CU/B10623/2014"
                     /host="Homo sapiens"
                     /db_xref="taxon:208893"
                     /country="Thailand"
                     /collection_date="09-Aug-2014"
                     /note="group: A"
     CDS             <1..651
                     /note="G protein"
                     /codon_start=1
                     /product="attachment glycoprotein"
                     /protein_id="APY20334"
                     /translation="GTTSQSTTILASTTPSAESTPQSTTVKIKNTTTTQILPSKTTTK
                     QHQNKPQNKPNNDFHFEVFNFVPCSICSNNPTCWAICKRIPNKKPGKKTTTKPTKKPT
```

```
                     LKTTKKDPKPQTTKPKEVLTTKPTGKPTINTTKTNIRTTLLTSNTKGNPEHTSQEETL
                     HSTTSEGYLSPSQVYTTSGQEETLHSTTSEGYLSPSQVYTTSEYLSQSLSSSNATK"
ORIGIN
        1 ggaactacat cacaatccac caccatacta gcttcaacaa caccaagtgc tgagtcaacc
       61 ccacaatcca caacagtcaa gatcaaaaac acaacaacaa cccaaatatt acctagcaaa
      121 accaccacaa aacaacacca aaataaacca caaaacaaac ccaacaatga ttttcacttt
      181 gaagtgttca attttgtacc ctgcagcata tgcagcaaca atccaacctg ctgggccatc
      241 tgcaagagaa taccaaacaa aaaacctgga aagaaaacca ccaccaagcc cacaaaaaaa
      301 ccaaccctca agacaaccaa aaaagatccc aaacctcaaa ccacaaaacc aaaggaagta
      361 ctcactacca agcctacagg aaagccaacc atcaacacca ctaaaacaaa catcagaact
      421 acattgctca cctccaacac caaaggaaat ccagaacaca caagtcaaga ggaaaccctc
      481 cactcaacca cctccgaagg ctatctaagc ccatcacaag tctatacaac atccggtcaa
      541 gaggaaaccc tccactcaac cacctccgaa ggttatctaa gcccatcaca agtctataca
      601 acatccgagt acctatcaca atctctatct tcatccaacg caacaaaatg a
//
LOCUS       KY327994                 651 bp    cRNA    linear   VRL 12-DEC-2016
DEFINITION  Human respiratory syncytial virus A isolate TH-CU/CB20/2013
            attachment glycoprotein gene, partial cds.
ACCESSION   KY327994
VERSION     KY327994
KEYWORDS    .
SOURCE      Human respiratory syncytial virus A
  ORGANISM  Human respiratory syncytial virus A
            Viruses; ssRNA viruses; ssRNA negative-strand viruses;
            Mononegavirales; Pneumoviridae; Orthopneumovirus.
REFERENCE   1  (bases 1 to 651)
  AUTHORS   Thongpan,I., Mauleekoonphairoj,J., Vichiwattana,P., Korkong,S.,
            Vongpunsawad,S. and Poovorawan,Y.
  TITLE     Molecular Characterization of Respiratory Syncytial Virus in
            Thailand, 2012-2015
  JOURNAL   Unpublished
REFERENCE   2  (bases 1 to 651)
  AUTHORS   Thongpan,I., Mauleekoonphairoj,J., Vichiwattana,P., Korkong,S.,
            Vongpunsawad,S. and Poovorawan,Y.
  TITLE     Direct Submission
  JOURNAL   Submitted (13-DEC-2016) Department of Pediatrics, Center of
            Excellence in Clinical Virology, Faculty of Medicine, Chulalongkorn
            University, Bangkok 10330, Thailand
COMMENT     ##Assembly-Data-START##
            Assembly Method       :: DNASTAR-Lasergene v. 6
            Sequencing Technology :: Sanger dideoxy sequencing
            ##Assembly-Data-END##
FEATURES             Location/Qualifiers
     source          1..651
                     /organism="Human respiratory syncytial virus A"
                     /mol_type="viral cRNA"
                     /isolate="TH-CU/CB20/2013"
                     /host="Homo sapiens"
                     /db_xref="taxon:208893"
                     /country="Thailand"
                     /collection_date="01-Jul-2013"
                     /note="group: A"
     CDS             <1..651
                     /note="G protein"
```

```
                     /codon_start=1
                     /product="attachment glycoprotein"
                     /protein_id="APY20335"
                     /translation="GTTSQSTTILASTTPSAESTPQSTTVKIINTTTTQILPSKPTTK
                     QRQNKPQNKPNNDFHFEVFNFVPCSICSNNPTCWAICKRIPNKKPGKKTTTKPTKKPT
                     LKTTKKDPKPQTTKPKEVLTTKPTGKPTINTTKTNIRTTLLTSNTKGNPEHTSQEETL
                     HSTTSEGYLSPSQVYTTSGQEETLHSTTSEGYPSPSQVYTTSEYLSQSLSSSNTTK"
ORIGIN
        1 ggaactacat cacaatccac caccatacta gcttcaacaa caccaagtgc tgagtcaacc
       61 ccacaatcca caacagtcaa gatcataaac acaacaacaa cccaaatatt acctagcaaa
      121 cccaccacaa aacaacgcca aaataaacca caaaacaaac ccaacaatga ttttcacttt
      181 gaagtgttca attttgtacc ctgcagcata tgcagcaaca atccaacctg ctgggccatc
      241 tgcaagagaa taccaaacaa aaaacctgga aagaaaacca ccaccaagcc cacaaaaaaa
      301 ccaaccctca agacaaccaa aaaagatccc aaacctcaaa ccacaaaacc aaaggaagta
      361 ctcactacca agcctacagg aaagccaacc atcaacacca ctaaaacaaa catcagaact
      421 acactgctca cctccaacac caaaggaaat ccagaacaca caagtcaaga ggaaaccctc
      481 cactcaacca cctccgaagg ctatctaagc ccatcacaag tctatacaac atccggtcaa
      541 gaggaaaccc tccactcaac cacctccgaa ggctatccaa gcccatcaca agtctacaca
      601 acatccgagt acctatcaca atctctatcc tcatccaaca caacaaaatg a
//
LOCUS       KY327995                 651 bp    cRNA    linear   VRL 12-DEC-2016
DEFINITION  Human respiratory syncytial virus A isolate TH-CU/C3207/2012
            attachment glycoprotein gene, partial cds.
ACCESSION   KY327995
VERSION     KY327995
KEYWORDS    .
SOURCE      Human respiratory syncytial virus A
  ORGANISM  Human respiratory syncytial virus A
            Viruses; ssRNA viruses; ssRNA negative-strand viruses;
            Mononegavirales; Pneumoviridae; Orthopneumovirus.
REFERENCE   1  (bases 1 to 651)
  AUTHORS   Thongpan,I., Mauleekoonphairoj,J., Vichiwattana,P., Korkong,S.,
            Vongpunsawad,S. and Poovorawan,Y.
  TITLE     Molecular Characterization of Respiratory Syncytial Virus in
            Thailand, 2012-2015
  JOURNAL   Unpublished
REFERENCE   2  (bases 1 to 651)
  AUTHORS   Thongpan,I., Mauleekoonphairoj,J., Vichiwattana,P., Korkong,S.,
            Vongpunsawad,S. and Poovorawan,Y.
  TITLE     Direct Submission
  JOURNAL   Submitted (13-DEC-2016) Department of Pediatrics, Center of
            Excellence in Clinical Virology, Faculty of Medicine, Chulalongkorn
            University, Bangkok 10330, Thailand
COMMENT     ##Assembly-Data-START##
            Assembly Method       :: DNASTAR-Lasergene v. 6
            Sequencing Technology :: Sanger dideoxy sequencing
            ##Assembly-Data-END##
FEATURES             Location/Qualifiers
     source          1..651
                     /organism="Human respiratory syncytial virus A"
                     /mol_type="viral cRNA"
                     /isolate="TH-CU/C3207/2012"
                     /host="Homo sapiens"
                     /db_xref="taxon:208893"
```

/country="Thailand"
/collection_date="17-Sep-2012"
/note="group: A"
CDS <1..651
/note="G protein"
/codon_start=1
/product="attachment glycoprotein"
/protein_id="APY20336"
/translation="GTTSQSTTILASTTPSAESTPQSTTVKIKNTTTTQILPSKPTTK
QRQNKPQNKPNNDFHFEVFNFVPCSICSNNPTCWAICKRIPNKKPGKKTTTKPTKKPT
LKTTKKDPKPQTTKPKEVLTTKPTGKPTINTTKTNIRTTLLTSNTKGNPEHTSQEETL
HSTTSEGYLSPSQVYTTSGQEETLHSTTSEGYLSPSQVYTTSEYLSQSLSSSNTTK"
ORIGIN
1 ggaactacat cacaatccac caccatacta gcttcaacaa caccaagtgc tgagtcaacc
61 ccacaatcca caacagtcaa gatcaaaaac acaacaacaa cccaaatatt acctagcaaa
121 cccaccacaa aacaacgcca aaataaacca caaaacaaac ccaacaatga ttttcacttt
181 gaagtgttca attttgtacc ctgcagcata tgcagcaaca atccaacctg ctgggccatc
241 tgcaagagaa taccaaacaa aaaacctgga aagaaaacca ccaccaagcc cacaaaaaaa
301 ccaaccctca agacaaccaa aaaagatccc aaacctcaaa ccacaaaacc aaaggaagta
361 ctcactacca agcctacagg aaagccaacc atcaacacca ctaaaacaaa catcagaact
421 acactgctca cctccaacac caaaggaaat ccagaacaca caagtcaaga ggaaaccctc
481 cactcaacca cctccgaagg ctatctaagc ccatcacaag tctatacaac atccggtcaa
541 gaggaaaccc tccactcaac cacctccgaa ggctatctaa gcccatcaca agtctataca
601 acatccgagt acctatcaca atctctatct tcatccaaca caacaaaatg a
//
LOCUS KY327996 651 bp cRNA linear VRL 12-DEC-2016
DEFINITION Human respiratory syncytial virus A isolate TH-CU/C3242/2012
attachment glycoprotein gene, partial cds.
ACCESSION KY327996
VERSION KY327996
KEYWORDS .
SOURCE Human respiratory syncytial virus A
ORGANISM Human respiratory syncytial virus A
Viruses; ssRNA viruses; ssRNA negative-strand viruses;
Mononegavirales; Pneumoviridae; Orthopneumovirus.
REFERENCE 1 (bases 1 to 651)
AUTHORS Thongpan,I., Mauleekoonphairoj,J., Vichiwattana,P., Korkong,S.,
Vongpunsawad,S. and Poovorawan,Y.
TITLE Molecular Characterization of Respiratory Syncytial Virus in
Thailand, 2012-2015
JOURNAL Unpublished
REFERENCE 2 (bases 1 to 651)
AUTHORS Thongpan,I., Mauleekoonphairoj,J., Vichiwattana,P., Korkong,S.,
Vongpunsawad,S. and Poovorawan,Y.
TITLE Direct Submission
JOURNAL Submitted (13-DEC-2016) Department of Pediatrics, Center of
Excellence in Clinical Virology, Faculty of Medicine, Chulalongkorn
University, Bangkok 10330, Thailand
COMMENT ##Assembly-Data-START##
Assembly Method :: DNASTAR-Lasergene v. 6
Sequencing Technology :: Sanger dideoxy sequencing
##Assembly-Data-END##
FEATURES Location/Qualifiers
source 1..651

```
                     /organism="Human respiratory syncytial virus A"
                     /mol_type="viral cRNA"
                     /isolate="TH-CU/C3242/2012"
                     /host="Homo sapiens"
                     /db_xref="taxon:208893"
                     /country="Thailand"
                     /collection_date="25-Sep-2012"
                     /note="group: A"
     CDS             <1..651
                     /note="G protein"
                     /codon_start=1
                     /product="attachment glycoprotein"
                     /protein_id="APY20337"
                     /translation="GTTSQSTTILASTTPSAESTPQSTTVKIKNTTTTQILPSKPTTK
                     QRQNKPQNKPNNDFHFEVFNFVPCSICSNNPTCWAICKRIPNKKPGKKTTTKPTKKPT
                     LKTTKKDPKPQTTKPKEVLTTKPTGKPTINTTKTNIRTTLLTSNTKGNPEHTSQEETL
                     HSTTSEGYLSPSQVYTTSGQEETLHSTTSEGYLSPSQVYTTSEYLSQSLSSSNTTK"
ORIGIN
        1 ggaactacat cacaatccac caccatacta gcttcaacaa caccaagtgc tgagtcaacc
       61 ccacaatcca caacagtcaa gatcaaaaac acaacaacaa cccaaatatt acctagcaaa
      121 cccaccacaa aacaacgcca aaataaacca caaaacaaac ccaacaatga ttttcacttt
      181 gaagtgttca attttgtacc ctgcagcata tgcagcaaca atccaacctg ctgggccatc
      241 tgcaagagaa taccaaacaa aaaacctgga aagaaaacca ccaccaagcc cacaaaaaaa
      301 ccaaccctca agacaaccaa aaaagatccc aaacctcaaa ccacaaaacc aaaggaagta
      361 ctcactacca agcctacagg aaagccaacc atcaacacca ctaaaacaaa catcagaact
      421 acactgctca cctccaacac caaaggaaat ccagaacaca caagtcaaga ggaaaccctc
      481 cactcaacca cctccgaagg ctatctaagc ccatcacaag tctatacaac atccggtcaa
      541 gaggaaaccc tccactcaac cacctccgaa ggctatctaa gcccatcaca agtctataca
      601 acatccgagt acctatcaca atctctatct tcatccaaca caacaaaatg a
//
LOCUS       KY327997                 651 bp    cRNA    linear   VRL 12-DEC-2016
DEFINITION  Human respiratory syncytial virus A isolate TH-CU/C3140/2012
            attachment glycoprotein gene, partial cds.
ACCESSION   KY327997
VERSION     KY327997
KEYWORDS    .
SOURCE      Human respiratory syncytial virus A
  ORGANISM  Human respiratory syncytial virus A
            Viruses; ssRNA viruses; ssRNA negative-strand viruses;
            Mononegavirales; Pneumoviridae; Orthopneumovirus.
REFERENCE   1  (bases 1 to 651)
  AUTHORS   Thongpan,I., Mauleekoonphairoj,J., Vichiwattana,P., Korkong,S.,
            Vongpunsawad,S. and Poovorawan,Y.
  TITLE     Molecular Characterization of Respiratory Syncytial Virus in
            Thailand, 2012-2015
  JOURNAL   Unpublished
REFERENCE   2  (bases 1 to 651)
  AUTHORS   Thongpan,I., Mauleekoonphairoj,J., Vichiwattana,P., Korkong,S.,
            Vongpunsawad,S. and Poovorawan,Y.
  TITLE     Direct Submission
  JOURNAL   Submitted (13-DEC-2016) Department of Pediatrics, Center of
            Excellence in Clinical Virology, Faculty of Medicine, Chulalongkorn
            University, Bangkok 10330, Thailand
COMMENT     ##Assembly-Data-START##
```

```
          Assembly Method       :: DNASTAR-Lasergene v. 6
          Sequencing Technology :: Sanger dideoxy sequencing
          ##Assembly-Data-END##
FEATURES             Location/Qualifiers
     source          1..651
                     /organism="Human respiratory syncytial virus A"
                     /mol_type="viral cRNA"
                     /isolate="TH-CU/C3140/2012"
                     /host="Homo sapiens"
                     /db_xref="taxon:208893"
                     /country="Thailand"
                     /collection_date="22-Aug-2012"
                     /note="group: A"
     CDS             <1..651
                     /note="G protein"
                     /codon_start=1
                     /product="attachment glycoprotein"
                     /protein_id="APY20338"
                     /translation="GTTSQSTTILASTTPSAESTPQSTTVKIKNTTTTQILPSKPTTK
                     QRQNKPQNKPNNDFHFEVFNFVPCSICSNNPTCWAICKRIPNKKPGKKTTTKPTKKPT
                     LKTTKKDPKPQTTKPKEVLTTKPTGKPTINTTKTNIRTTLLTSNTKGNPEHTSQEETL
                     HSTTSEGYLSPSQVYTTSGQEETLHSTTSEGYLSPSQVYTTSEYLSQSLSSSNTTK"
ORIGIN      
        1 ggaactacat cacaatccac caccatacta gcttcaacaa caccaagtgc tgagtcaacc
       61 ccacaatcca caacagtcaa gatcaaaaac acaacaacaa cccaaatatt acctagcaaa
      121 cccaccacaa aacaacgcca aaataaacca caaaacaaac ccaacaatga ttttcacttt
      181 gaagtgttca attttgtacc ctgcagcata tgcagcaaca atccaacctg ctgggccatc
      241 tgcaagagaa taccaaacaa aaaacctgga aagaaaacca ccaccaagcc cacaaaaaaa
      301 ccaaccctca agacaaccaa aaaagatccc aaacctcaaa ccacaaaacc aaaggaagta
      361 ctcactacca agcctacagg aaagccaacc atcaacacca ctaaaacaaa catcagaact
      421 acactgctca cctccaacac caaaggaaat ccagaacaca caagtcaaga ggaaaccctc
      481 cactcaacca cctccgaagg ctatctaagc ccatcacaag tctatacaac atccggtcaa
      541 gaggaaaccc tccactcaac cacctccgaa ggctatctaa gcccatcaca agtctataca
      601 acatccgagt acctatcaca atctctatct tcatccaaca caacaaaatg a
//
LOCUS       KY327998                 651 bp    cRNA    linear   VRL 12-DEC-2016
DEFINITION  Human respiratory syncytial virus A isolate TH-CU/C3208/2012
            attachment glycoprotein gene, partial cds.
ACCESSION   KY327998
VERSION     KY327998
KEYWORDS    .
SOURCE      Human respiratory syncytial virus A
  ORGANISM  Human respiratory syncytial virus A
            Viruses; ssRNA viruses; ssRNA negative-strand viruses;
            Mononegavirales; Pneumoviridae; Orthopneumovirus.
REFERENCE   1  (bases 1 to 651)
  AUTHORS   Thongpan,I., Mauleekoonphairoj,J., Vichiwattana,P., Korkong,S.,
            Vongpunsawad,S. and Poovorawan,Y.
  TITLE     Molecular Characterization of Respiratory Syncytial Virus in
            Thailand, 2012-2015
  JOURNAL   Unpublished
REFERENCE   2  (bases 1 to 651)
  AUTHORS   Thongpan,I., Mauleekoonphairoj,J., Vichiwattana,P., Korkong,S.,
            Vongpunsawad,S. and Poovorawan,Y.
```

TITLE Direct Submission
JOURNAL Submitted (13-DEC-2016) Department of Pediatrics, Center of
Excellence in Clinical Virology, Faculty of Medicine, Chulalongkorn
University, Bangkok 10330, Thailand
COMMENT ##Assembly-Data-START##
Assembly Method :: DNASTAR-Lasergene v. 6
Sequencing Technology :: Sanger dideoxy sequencing
##Assembly-Data-END##
FEATURES Location/Qualifiers
source 1..651
/organism="Human respiratory syncytial virus A"
/mol_type="viral cRNA"
/isolate="TH-CU/C3208/2012"
/host="Homo sapiens"
/db_xref="taxon:208893"
/country="Thailand"
/collection_date="17-Sep-2012"
/note="group: A"
CDS <1..651
/note="G protein"
/codon_start=1
/product="attachment glycoprotein"
/protein_id="APY20339"
/translation="GTTSQSTTILASTTPSAESTPQSTTVKIKNTTTTQILPSKPTTK
QRQNKPQNKPNNDFHFEVFNFVPCSICSNNPTCWAICKRIPNKKPGKKTTTKPTKKPT
LKTTKKDPKPQTTKPKEVLTTKPTGKPTINTTKTNIRTTLLTSNTKGNPEHTSQEETL
HSTTSEGYLSPSQVYTTSGQEETLHSTTSEGYLSPSQVYTTSEYLSQSLSSSNTTK"
ORIGIN
1 ggaactacat cacaatccac caccatacta gcttcaacaa caccaagtgc tgagtcaacc
61 ccacaatcca caacagtcaa gatcaaaaac acaacaacaa cccaaatatt acctagcaaa
121 cccaccacaa aacaacgcca aaataaacca caaaacaaac ccaacaatga ttttcacttt
181 gaagtgttca attttgtacc ctgcagcata tgcagcaaca atccaacctg ctgggccatc
241 tgcaagagaa taccaaacaa aaaacctgga aagaaaacca ccaccaagcc cacaaaaaaa
301 ccaaccctca agacaaccaa aaaagatccc aaacctcaaa ccacaaaacc aaaggaagta
361 ctcactacca agcctacagg aaagccaacc atcaacacca ctaaaacaaa catcagaact
421 acactgctca cctccaacac caaaggaaat ccagaacaca caagtcaaga ggaaaccctc
481 cactcaacca cctccgaagg ctatctaagc ccatcacaag tctatacaac atctggtcaa
541 gaggaaaccc tccactcaac cacctccgaa ggctatctaa gcccatcaca agtctataca
601 acatccgagt acctatcaca atctctatct tcatccaaca caacaaaatg a
//
LOCUS KY327999 651 bp cRNA linear VRL 12-DEC-2016
DEFINITION Human respiratory syncytial virus A isolate TH-CU/B10810/2014
attachment glycoprotein gene, partial cds.
ACCESSION KY327999
VERSION KY327999
KEYWORDS .
SOURCE Human respiratory syncytial virus A
ORGANISM Human respiratory syncytial virus A
Viruses; ssRNA viruses; ssRNA negative-strand viruses;
Mononegavirales; Pneumoviridae; Orthopneumovirus.
REFERENCE 1 (bases 1 to 651)
AUTHORS Thongpan,I., Mauleekoonphairoj,J., Vichiwattana,P., Korkong,S.,
Vongpunsawad,S. and Poovorawan,Y.
TITLE Molecular Characterization of Respiratory Syncytial Virus in

Thailand, 2012-2015
JOURNAL Unpublished
REFERENCE 2 (bases 1 to 651)
AUTHORS Thongpan,I., Mauleekoonphairoj,J., Vichiwattana,P., Korkong,S.,
Vongpunsawad,S. and Poovorawan,Y.
TITLE Direct Submission
JOURNAL Submitted (13-DEC-2016) Department of Pediatrics, Center of
Excellence in Clinical Virology, Faculty of Medicine, Chulalongkorn
University, Bangkok 10330, Thailand
COMMENT ##Assembly-Data-START##
Assembly Method :: DNASTAR-Lasergene v. 6
Sequencing Technology :: Sanger dideoxy sequencing
##Assembly-Data-END##
FEATURES Location/Qualifiers
source 1..651
/organism="Human respiratory syncytial virus A"
/mol_type="viral cRNA"
/isolate="TH-CU/B10810/2014"
/host="Homo sapiens"
/db_xref="taxon:208893"
/country="Thailand"
/collection_date="28-Aug-2014"
/note="group: A"
CDS <1..651
/note="G protein"
/codon_start=1
/product="attachment glycoprotein"
/protein_id="APY20340"
/translation="GTTSQSTTILASTTPSAESTPQSTTVKIKNTTTTQILPSKPTTK
QRQNKPQNKPNNDFHFEVFNFVPCSICSNNPTCWAICKRIPNKKPGKKTTTKPTKKPT
LKTTKKDPKPQTTKPKEVLTTKPTGKPTINTTKTNIRTTLLTSNTKGNPEHTSQEETL
HSTTSEGYLSPSQVYTTSGQEETLHSTTSEGYLSPSQVYTTSEYLSQSLSSSNTTK"
ORIGIN
1 ggaactacat cacaatccac caccatacta gcttcaacaa caccaagtgc tgagtcaacc
61 ccacaatcca caacagtcaa gatcaaaaac acaacaacaa cccaaatatt acctagcaaa
121 cccaccacaa aacaacgcca aaataaacca caaaacaaac ccaacaatga ttttcacttt
181 gaagtgttca attttgtacc ctgcagcata tgcagcaaca atccaacctg ctgggccatc
241 tgcaagagaa taccaaacaa aaaacctgga aagaaaacca ccaccaagcc cacaaaaaaa
301 ccaaccctca agacaaccaa aaaagatccc aaacctcaaa ccacaaaacc aaaggaagta
361 ctcactacca agcccacagg aaagccaacc atcaacacca ctaaaacaaa catcagaact
421 acactgctca cctccaacac caaaggaaat ccagaacaca caagtcaaga ggaaaccctc
481 cactcaacca cctccgaagg ctatctaagc ccatcacaag tctatacaac atccggtcaa
541 gaggaaaccc tccactcaac cacctccgaa ggctatctaa gcccatcaca agtctataca
601 acatccgagt acctatcaca atctctatct tcatccaaca caacaaaatg a
//
LOCUS KY328000 651 bp cRNA linear VRL 12-DEC-2016
DEFINITION Human respiratory syncytial virus A isolate TH-CU/C3434/2012
attachment glycoprotein gene, partial cds.
ACCESSION KY328000
VERSION KY328000
KEYWORDS .
SOURCE Human respiratory syncytial virus A
ORGANISM Human respiratory syncytial virus A
Viruses; ssRNA viruses; ssRNA negative-strand viruses;

Mononegavirales; Pneumoviridae; Orthopneumovirus.
REFERENCE 1 (bases 1 to 651)
AUTHORS Thongpan,I., Mauleekoonphairoj,J., Vichiwattana,P., Korkong,S.,
Vongpunsawad,S. and Poovorawan,Y.
TITLE Molecular Characterization of Respiratory Syncytial Virus in
Thailand, 2012-2015
JOURNAL Unpublished
REFERENCE 2 (bases 1 to 651)
AUTHORS Thongpan,I., Mauleekoonphairoj,J., Vichiwattana,P., Korkong,S.,
Vongpunsawad,S. and Poovorawan,Y.
TITLE Direct Submission
JOURNAL Submitted (13-DEC-2016) Department of Pediatrics, Center of
Excellence in Clinical Virology, Faculty of Medicine, Chulalongkorn
University, Bangkok 10330, Thailand
COMMENT ##Assembly-Data-START##
Assembly Method :: DNASTAR-Lasergene v. 6
Sequencing Technology :: Sanger dideoxy sequencing
##Assembly-Data-END##
FEATURES Location/Qualifiers
source 1..651
/organism="Human respiratory syncytial virus A"
/mol_type="viral cRNA"
/isolate="TH-CU/C3434/2012"
/host="Homo sapiens"
/db_xref="taxon:208893"
/country="Thailand"
/collection_date="18-Dec-2012"
/note="group: A"
CDS <1..651
/note="G protein"
/codon_start=1
/product="attachment glycoprotein"
/protein_id="APY20341"
/translation="GTTSQSTTILASTTPSAESTPQSTTVKIKNITTTQILPSKPTTK
QRQNKPQNKPNNDFHFEVFNFVPCSICSNNPTCWAICKRIPNKKPGKKTTTKPTKKPT
LKTTKKDPKPQTTKPKEVLTTKPTGMPTINTTKTNIRTTLLTSNTKGNPEHTSQEETL
HSTTSEGYLSPSQVYTTSGQEETLHSTTSKGYLSPSQVYTTSEYLSQSLSSSNTTK"
ORIGIN
1 ggaactacat cacaatccac caccatacta gcttcaacaa caccaagtgc tgagtcaacc
61 ccacaatcca caacagtcaa gatcaaaaac ataacaacaa cccaaatatt acctagcaaa
121 cccaccacaa aacaacgcca aaataaacca caaaacaaac ccaacaatga ttttcacttt
181 gaagtgttca attttgtacc ctgcagcata tgcagcaaca atccaacctg ctgggccatc
241 tgcaagagaa taccaaacaa aaaacctgga aagaaaacca ccaccaagcc cacaaaaaaa
301 ccaaccctca agacaaccaa aaaagatccc aaacccccaaa ccacaaaacc aaaggaagta
361 ctcactacca agcctacagg aatgccaacc atcaacacca ctaaaacaaa catcagaact
421 acactgctca cctccaacac caaaggaaat ccagaacaca caagtcaaga ggaaaccctc
481 cactcaacca cctccgaagg ctatctaagc ccatcacaag tctatacaac atccggtcaa
541 gaggaaaccc tccactcaac cacctccaaa ggctatctaa gcccatcaca agtctataca
601 acatccgagt acctatcaca atctctatct tcatccaaca caacaaaatg a
//
LOCUS KY328001 651 bp cRNA linear VRL 12-DEC-2016
DEFINITION Human respiratory syncytial virus A isolate TH-CU/B10631/2014
attachment glycoprotein gene, partial cds.
ACCESSION KY328001

```
VERSION     KY328001
KEYWORDS    .
SOURCE      Human respiratory syncytial virus A
  ORGANISM  Human respiratory syncytial virus A
            Viruses; ssRNA viruses; ssRNA negative-strand viruses;
            Mononegavirales; Pneumoviridae; Orthopneumovirus.
REFERENCE   1  (bases 1 to 651)
  AUTHORS   Thongpan,I., Mauleekoonphairoj,J., Vichiwattana,P., Korkong,S.,
            Vongpunsawad,S. and Poovorawan,Y.
  TITLE     Molecular Characterization of Respiratory Syncytial Virus in
            Thailand, 2012-2015
  JOURNAL   Unpublished
REFERENCE   2  (bases 1 to 651)
  AUTHORS   Thongpan,I., Mauleekoonphairoj,J., Vichiwattana,P., Korkong,S.,
            Vongpunsawad,S. and Poovorawan,Y.
  TITLE     Direct Submission
  JOURNAL   Submitted (13-DEC-2016) Department of Pediatrics, Center of
            Excellence in Clinical Virology, Faculty of Medicine, Chulalongkorn
            University, Bangkok 10330, Thailand
COMMENT     ##Assembly-Data-START##
            Assembly Method       :: DNASTAR-Lasergene v. 6
            Sequencing Technology :: Sanger dideoxy sequencing
            ##Assembly-Data-END##
FEATURES             Location/Qualifiers
     source          1..651
                     /organism="Human respiratory syncytial virus A"
                     /mol_type="viral cRNA"
                     /isolate="TH-CU/B10631/2014"
                     /host="Homo sapiens"
                     /db_xref="taxon:208893"
                     /country="Thailand"
                     /collection_date="10-Aug-2014"
                     /note="group: A"
     CDS             <1..651
                     /note="G protein"
                     /codon_start=1
                     /product="attachment glycoprotein"
                     /protein_id="APY20342"
                     /translation="GTTSQSTTILASTTPSAESTPQSTTVKIKNTTTTQILPSKPTTK
                     QRQNKPQNKPNNDFHFEVFNFVPCSICSNNPTCWAICKRIPNKKPGKKTTTKPTKKPT
                     LKTTKKDPKPQTTKPKEVLTTKPTGKPTINTTKTNIRTTLLTSNTKGNPEHTSQEETL
                     HSTTSEGYLSPSQVYTTSGQEETLHSTTSKGYPSPSQVYTISEYLSQSLSSSNTTK"
ORIGIN
        1 ggaactacat cacaatccac caccatacta gcttcaacaa caccaagtgc tgagtcaacc
       61 ccacaatcca caacagtcaa gatcaaaaac acaacaacaa cccaaatatt acctagcaaa
      121 cccaccacaa aacaacgcca aaataaacca caaaacaaac ccaacaatga ttttcacttt
      181 gaagtgttca attttgtacc ctgcagcata tgcagcaaca atccaacctg ctgggccatc
      241 tgcaagagaa taccaaacaa aaaacctgga aagaaaacca ccaccaagcc cacaaaaaaa
      301 ccaaccctca agacaaccaa aaaagatccc aaaccccaaa ccacaaaacc aaaggaagta
      361 ctcactacca agcctacagg aaagccaacc atcaacacca ctaaaacaaa catcagaact
      421 acactgctca cctccaacac caaaggaaat ccagaacaca caagtcaaga ggaaaccctc
      481 cactcaacca cctccgaagg ctatctaagc ccatcacaag tctatacaac atccggtcaa
      541 gaggaaaccc tccactcaac cacctccaaa ggctatccaa gcccatcaca agtctacaca
      601 atatccgagt acctatcaca atctctatct tcatccaaca caacaaaatg a
```

```
//
LOCUS       KY328002                 651 bp    cRNA    linear   VRL 12-DEC-2016
DEFINITION  Human respiratory syncytial virus A isolate TH-CU/B10633/2014
            attachment glycoprotein gene, partial cds.
ACCESSION   KY328002
VERSION     KY328002
KEYWORDS    .
SOURCE      Human respiratory syncytial virus A
  ORGANISM  Human respiratory syncytial virus A
            Viruses; ssRNA viruses; ssRNA negative-strand viruses;
            Mononegavirales; Pneumoviridae; Orthopneumovirus.
REFERENCE   1  (bases 1 to 651)
  AUTHORS   Thongpan,I., Mauleekoonphairoj,J., Vichiwattana,P., Korkong,S.,
            Vongpunsawad,S. and Poovorawan,Y.
  TITLE     Molecular Characterization of Respiratory Syncytial Virus in
            Thailand, 2012-2015
  JOURNAL   Unpublished
REFERENCE   2  (bases 1 to 651)
  AUTHORS   Thongpan,I., Mauleekoonphairoj,J., Vichiwattana,P., Korkong,S.,
            Vongpunsawad,S. and Poovorawan,Y.
  TITLE     Direct Submission
  JOURNAL   Submitted (13-DEC-2016) Department of Pediatrics, Center of
            Excellence in Clinical Virology, Faculty of Medicine, Chulalongkorn
            University, Bangkok 10330, Thailand
COMMENT     ##Assembly-Data-START##
            Assembly Method       :: DNASTAR-Lasergene v. 6
            Sequencing Technology :: Sanger dideoxy sequencing
            ##Assembly-Data-END##
FEATURES             Location/Qualifiers
     source          1..651
                     /organism="Human respiratory syncytial virus A"
                     /mol_type="viral cRNA"
                     /isolate="TH-CU/B10633/2014"
                     /host="Homo sapiens"
                     /db_xref="taxon:208893"
                     /country="Thailand"
                     /collection_date="10-Aug-2014"
                     /note="group: A"
     CDS             <1..651
                     /note="G protein"
                     /codon_start=1
                     /product="attachment glycoprotein"
                     /protein_id="APY20343"
                     /translation="GTTSQSTTILASTTPSAESTPQSTTVKIKNTTTTQILPSKPTTK
                     QRQNKPQNKPNNDFHFEVFNFVPCSICSNNPTCWAICKRIPNKKPGKKTTTKPTKKPT
                     LKTTKKDPKPQTTKPKEVLTTKPTGKPTINTTKTNIRTTLLTSNTKGNPEHTSQEETL
                     HSTTSEGYLSPSQVYTTSGQEETLHSTTSKGYPSPSQVYTTSEYLSQSLSSSNTTK"
ORIGIN
        1 ggaactacat cacaatccac caccatacta gcttcaacaa caccaagtgc tgagtcaacc
       61 ccacaatcca caacagtcaa gatcaaaaac acaacaacaa cccaaatatt acctagcaaa
      121 cccaccacaa aacaacgcca aaataaacca caaaacaaac ccaacaatga ttttcacttt
      181 gaagtgttca attttgtacc ctgcagcata tgcagcaaca atccaacctg ctgggccatc
      241 tgcaagagaa taccaaacaa aaaacctgga aagaaaacca ccaccaagcc cacaaaaaaa
      301 ccaaccctca agacaaccaa aaaagatccc aaaccccaaa ccacaaaacc aaaggaagta
```

```
     361 ctcactacca agcctacagg aaagccaacc atcaacacca ctaaaacaaa catcagaact
     421 acactgctca cctccaacac caaaggaaat ccagaacaca caagtcaaga ggaaaccctc
     481 cactcaacca cctccgaagg ctatctaagc ccatcacaag tctatacaac atccggtcaa
     541 gaggaaaccc tccactcaac cacctccaaa ggctatccaa gcccatcaca agtctacaca
     601 acatccgagt acctatcaca atctctatct tcatccaaca caacaaaatg a
//
LOCUS       KY328003                 651 bp    cRNA    linear   VRL 12-DEC-2016
DEFINITION  Human respiratory syncytial virus A isolate ON1/TH-CU448/2012
            attachment glycoprotein gene, partial cds.
ACCESSION   KY328003
VERSION     KY328003
KEYWORDS    .
SOURCE      Human respiratory syncytial virus A
  ORGANISM  Human respiratory syncytial virus A
            Viruses; ssRNA viruses; ssRNA negative-strand viruses;
            Mononegavirales; Pneumoviridae; Orthopneumovirus.
REFERENCE   1  (bases 1 to 651)
  AUTHORS   Thongpan,I., Mauleekoonphairoj,J., Vichiwattana,P., Korkong,S.,
            Vongpunsawad,S. and Poovorawan,Y.
  TITLE     Molecular Characterization of Respiratory Syncytial Virus in
            Thailand, 2012-2015
  JOURNAL   Unpublished
REFERENCE   2  (bases 1 to 651)
  AUTHORS   Thongpan,I., Mauleekoonphairoj,J., Vichiwattana,P., Korkong,S.,
            Vongpunsawad,S. and Poovorawan,Y.
  TITLE     Direct Submission
  JOURNAL   Submitted (13-DEC-2016) Department of Pediatrics, Center of
            Excellence in Clinical Virology, Faculty of Medicine, Chulalongkorn
            University, Bangkok 10330, Thailand
COMMENT     ##Assembly-Data-START##
            Assembly Method       :: DNASTAR-Lasergene v. 6
            Sequencing Technology :: Sanger dideoxy sequencing
            ##Assembly-Data-END##
FEATURES             Location/Qualifiers
     source          1..651
                     /organism="Human respiratory syncytial virus A"
                     /mol_type="viral cRNA"
                     /isolate="ON1/TH-CU448/2012"
                     /host="Homo sapiens"
                     /db_xref="taxon:208893"
                     /country="Thailand"
                     /collection_date="10-Sep-2012"
                     /note="group: A"
     CDS             <1..651
                     /note="G protein"
                     /codon_start=1
                     /product="attachment glycoprotein"
                     /protein_id="APY20344"
                     /translation="GTTSQSTTILASTTPSAESTPQSTTVKIKNTTTTQILPSKPTTK
                     QRQNKPQNKPNNDFHFEVFNFVPCSICSNNPTCWAICKRIPNKKPGKKTTTKPTKKPT
                     LKTTKKDPKPQTTKPKEVLTTKPTGKPTINTTKTNIRTTLLTSNTKGNPEHTSQEETL
                     HSTTSEGYLSPSQVYTTSGQEETLHSTTSKGYLSPSQVYTTSEYLSQSLSSSNTTK"
ORIGIN
        1 ggaactacat cacaatccac caccatacta gcttcaacaa caccaagtgc tgagtcaacc
```

```
      61 ccacaatcca caacagtcaa gatcaaaaac acaacaacaa cccaaatatt acctagcaaa
     121 cccaccacaa aacaacgcca aaataaacca caaaacaaac ccaacaatga ttttcacttt
     181 gaagtgttca attttgtacc ctgcagcata tgcagcaaca atccaacctg ctgggccatc
     241 tgcaagagaa taccaaacaa aaaacctgga aagaaaacca ccaccaagcc cacaaaaaaa
     301 ccaaccctca agacaaccaa aaaagatccc aaaccccaaa ccacaaaacc aaaggaagta
     361 ctcactacca agcctacagg aaagccaacc atcaacacca ctaaaacaaa catcagaact
     421 acactgctca cctccaacac caaaggaaat ccagaacaca caagtcaaga ggaaaccctc
     481 cactcaacca cctccgaagg ctatctaagc ccatcacaag tctatacaac atccggtcaa
     541 gaggaaaccc tccactcaac cacctccaaa ggctatctaa gcccatcaca agtctataca
     601 acatccgagt acctatcaca atctctatct tcatccaaca caacaaaatg a
//
LOCUS       KY328004                 651 bp    cRNA    linear   VRL 12-DEC-2016
DEFINITION  Human respiratory syncytial virus A isolate TH-CU392/2012
            attachment glycoprotein gene, partial cds.
ACCESSION   KY328004
VERSION     KY328004
KEYWORDS    .
SOURCE      Human respiratory syncytial virus A
  ORGANISM  Human respiratory syncytial virus A
            Viruses; ssRNA viruses; ssRNA negative-strand viruses;
            Mononegavirales; Pneumoviridae; Orthopneumovirus.
REFERENCE   1  (bases 1 to 651)
  AUTHORS   Thongpan,I., Mauleekoonphairoj,J., Vichiwattana,P., Korkong,S.,
            Vongpunsawad,S. and Poovorawan,Y.
  TITLE     Molecular Characterization of Respiratory Syncytial Virus in
            Thailand, 2012-2015
  JOURNAL   Unpublished
REFERENCE   2  (bases 1 to 651)
  AUTHORS   Thongpan,I., Mauleekoonphairoj,J., Vichiwattana,P., Korkong,S.,
            Vongpunsawad,S. and Poovorawan,Y.
  TITLE     Direct Submission
  JOURNAL   Submitted (13-DEC-2016) Department of Pediatrics, Center of
            Excellence in Clinical Virology, Faculty of Medicine, Chulalongkorn
            University, Bangkok 10330, Thailand
COMMENT     ##Assembly-Data-START##
            Assembly Method       :: DNASTAR-Lasergene v. 6
            Sequencing Technology :: Sanger dideoxy sequencing
            ##Assembly-Data-END##
FEATURES             Location/Qualifiers
     source          1..651
                     /organism="Human respiratory syncytial virus A"
                     /mol_type="viral cRNA"
                     /isolate="TH-CU392/2012"
                     /host="Homo sapiens"
                     /db_xref="taxon:208893"
                     /country="Thailand"
                     /collection_date="05-Jul-2012"
                     /note="group: A"
     CDS             <1..651
                     /note="G protein"
                     /codon_start=1
                     /product="attachment glycoprotein"
                     /protein_id="APY20345"
                     /translation="GTTSQSTTILASTTPSAESTPQSTTVKIKNTTTTQILPSKPTTK
```

QRQNKPQNKPNNDFHFEVFNFVPCSICSNNPTCWAICKRIPNKKPGKKTTTKPTKKPT
LKTTKKDPKPQTTKPKEVLTTKPTGKPTINTTKTNIRTTLLTSNTKGNPEHTSQEETL
HSTTSEGYLSPSQVYTTSGQEETLHSTTSKGYLSPSQVYTTSEYLSQSLSSSNTTK"

```
ORIGIN
        1 ggaactacat cacaatccac caccatacta gcttcaacaa caccaagtgc tgagtcaacc
       61 ccacaatcca caacagtcaa gatcaaaaac acaacaacaa cccaaatatt acctagcaaa
      121 cccaccacaa aacaacgcca aaataaacca caaaacaaac ccaacaatga ttttcacttt
      181 gaagtgttca attttgtacc ctgcagcata tgcagcaaca atccaacctg ctgggccatc
      241 tgcaagagaa taccaaacaa aaaacctgga aagaaaacca ccaccaagcc cacaaaaaaa
      301 ccaaccctca agacaaccaa aaaagatccc aaaccccaaa ccacaaaacc aaaggaagta
      361 ctcactacca agcctacagg aaagccaacc atcaacacca ctaaaacaaa catcagaact
      421 acactgctca cctccaacac caaaggaaat ccagaacaca caagtcaaga ggaaaccctc
      481 cactcaacca cctccgaagg ctatctaagc ccatcacaag tctatacaac atccggtcaa
      541 gaggaaaccc tccactcaac cacctccaaa ggctatctaa gcccatcaca agtctataca
      601 acatccgagt acctatcaca atctctatct tcatccaaca caacaaaatg a
//
LOCUS       KY328005                 651 bp    cRNA    linear   VRL 12-DEC-2016
DEFINITION  Human respiratory syncytial virus A isolate TH-CU495/2012
            attachment glycoprotein gene, partial cds.
ACCESSION   KY328005
VERSION     KY328005
KEYWORDS    .
SOURCE      Human respiratory syncytial virus A
  ORGANISM  Human respiratory syncytial virus A
            Viruses; ssRNA viruses; ssRNA negative-strand viruses;
            Mononegavirales; Pneumoviridae; Orthopneumovirus.
REFERENCE   1  (bases 1 to 651)
  AUTHORS   Thongpan,I., Mauleekoonphairoj,J., Vichiwattana,P., Korkong,S.,
            Vongpunsawad,S. and Poovorawan,Y.
  TITLE     Molecular Characterization of Respiratory Syncytial Virus in
            Thailand, 2012-2015
  JOURNAL   Unpublished
REFERENCE   2  (bases 1 to 651)
  AUTHORS   Thongpan,I., Mauleekoonphairoj,J., Vichiwattana,P., Korkong,S.,
            Vongpunsawad,S. and Poovorawan,Y.
  TITLE     Direct Submission
  JOURNAL   Submitted (13-DEC-2016) Department of Pediatrics, Center of
            Excellence in Clinical Virology, Faculty of Medicine, Chulalongkorn
            University, Bangkok 10330, Thailand
COMMENT     ##Assembly-Data-START##
            Assembly Method       :: DNASTAR-Lasergene v. 6
            Sequencing Technology :: Sanger dideoxy sequencing
            ##Assembly-Data-END##
FEATURES             Location/Qualifiers
     source          1..651
                     /organism="Human respiratory syncytial virus A"
                     /mol_type="viral cRNA"
                     /isolate="TH-CU495/2012"
                     /host="Homo sapiens"
                     /db_xref="taxon:208893"
                     /country="Thailand"
                     /collection_date="15-Nov-2012"
                     /note="group: A"
     CDS             <1..651
```

```
                     /note="G protein"
                     /codon_start=1
                     /product="attachment glycoprotein"
                     /protein_id="APY20346"
                     /translation="GTTSQSTTILASTTPSAESTPQSTTVKIKNTTTTQILPSKPTTK
                     QRQNKPQNKPNNDFHFEVFNFVPCSICSNNPTCWAICKRIPNKKPGKKTTTKPTKKPT
                     LKTTKKDPKPQTTKPKEVLTTKPTGKPTINTTKTNIRTTLLTSNTKGNPEHTSQEETL
                     HSTTSEGYLSPSQVYTTSGQEETLHSTTSKGYLSPSQVYTTSEYLSQSLSSSNTTK"
ORIGIN
        1 ggaactacat cacaatccac caccatacta gcttcaacaa caccaagtgc tgagtcaacc
       61 ccacaatcca caacagtcaa gatcaaaaac acaacaacaa cccaaatatt acctagcaaa
      121 cccaccacaa aacaacgcca aaataaacca caaaacaaac ccaacaatga ttttcacttt
      181 gaagtgttca attttgtacc ctgcagcata tgcagcaaca atccaacctg ctgggccatc
      241 tgcaagagaa taccaaacaa aaaacctgga aagaaaacca ccaccaagcc cacaaaaaaa
      301 ccaaccctca agacaaccaa aaaagatccc aaaccccaaa ccacaaaacc aaaggaagta
      361 ctcactacca agcctacagg aaagccaacc atcaacacca ctaaaacaaa catcagaact
      421 acactgctca cctccaacac caaaggaaat ccagaacaca caagtcaaga ggaaaccctc
      481 cactcaacca cctccgaagg ctatctaagc ccatcacaag tctatacaac atccggtcaa
      541 gaggaaaccc tccactcaac cacctccaaa ggctatctaa gcccatcaca agtctataca
      601 acatccgagt acctatcaca atctctatct tcatccaaca caacaaaatg a
//
LOCUS       KY328006                 651 bp    cRNA    linear   VRL 12-DEC-2016
DEFINITION  Human respiratory syncytial virus A isolate TH-CU437/2012
            attachment glycoprotein gene, partial cds.
ACCESSION   KY328006
VERSION     KY328006
KEYWORDS    .
SOURCE      Human respiratory syncytial virus A
  ORGANISM  Human respiratory syncytial virus A
            Viruses; ssRNA viruses; ssRNA negative-strand viruses;
            Mononegavirales; Pneumoviridae; Orthopneumovirus.
REFERENCE   1  (bases 1 to 651)
  AUTHORS   Thongpan,I., Mauleekoonphairoj,J., Vichiwattana,P., Korkong,S.,
            Vongpunsawad,S. and Poovorawan,Y.
  TITLE     Molecular Characterization of Respiratory Syncytial Virus in
            Thailand, 2012-2015
  JOURNAL   Unpublished
REFERENCE   2  (bases 1 to 651)
  AUTHORS   Thongpan,I., Mauleekoonphairoj,J., Vichiwattana,P., Korkong,S.,
            Vongpunsawad,S. and Poovorawan,Y.
  TITLE     Direct Submission
  JOURNAL   Submitted (13-DEC-2016) Department of Pediatrics, Center of
            Excellence in Clinical Virology, Faculty of Medicine, Chulalongkorn
            University, Bangkok 10330, Thailand
COMMENT     ##Assembly-Data-START##
            Assembly Method       :: DNASTAR-Lasergene v. 6
            Sequencing Technology :: Sanger dideoxy sequencing
            ##Assembly-Data-END##
FEATURES             Location/Qualifiers
     source          1..651
                     /organism="Human respiratory syncytial virus A"
                     /mol_type="viral cRNA"
                     /isolate="TH-CU437/2012"
                     /host="Homo sapiens"
```

```
                     /db_xref="taxon:208893"
                     /country="Thailand"
                     /collection_date="03-Sep-2012"
                     /note="group: A"
     CDS             <1..651
                     /note="G protein"
                     /codon_start=1
                     /product="attachment glycoprotein"
                     /protein_id="APY20347"
                     /translation="GTTSQSTTILASTTPSAESTPQSTTVKIKNTTTTQILPSKPTTK
                     QRQNKPQNKPNNDFHFEVFNFVPCSICSNNPTCWAICKRIPNKKPGKKTTTKPTKKPT
                     LKTTKKDPKPQTTKPKEVLTTKPTGKPTINTTKTNIRTTLLTSNTKGNPEHTSQEETL
                     HSTTSEGYLSPSQVYTTSGQEETLHSTTSKGYLSPSQVYTTSEYLSQSLSSSNTTK"
ORIGIN
        1 ggaactacat cacaatccac caccatacta gcttcaacaa caccaagtgc tgagtcaacc
       61 ccacaatcca caacagtcaa gatcaaaaac acaacaacaa cccaaatatt acctagcaaa
      121 cccaccacaa aacaacgcca aaataaacca caaaacaaac ccaacaatga ttttcacttt
      181 gaagtgttca attttgtacc ctgcagcata tgcagcaaca atccaacctg ctgggccatc
      241 tgcaagagaa taccaaacaa aaaacctgga aagaaaacca ccaccaagcc cacaaaaaaa
      301 ccaaccctca agacaaccaa aaaagatccc aaacccccaaa ccacaaaacc aaaggaagta
      361 ctcactacca agcctacagg aaagccaacc atcaacacca ctaaaacaaa catcagaact
      421 acactgctca cctccaacac caaaggaaat ccagaacaca caagtcaaga ggaaaccctc
      481 cactcaacca cctccgaagg ctatctaagc ccatcacaag tctatacaac atccggtcaa
      541 gaggaaaccc tccactcaac cacctccaaa ggctatctaa gcccatcaca agtctataca
      601 acatccgagt acctatcaca atctctatct tcatccaaca caacaaaatg a
//
LOCUS       KY328007                 651 bp    cRNA    linear   VRL 12-DEC-2016
DEFINITION  Human respiratory syncytial virus A isolate TH-CU457/2012
            attachment glycoprotein gene, partial cds.
ACCESSION   KY328007
VERSION     KY328007
KEYWORDS    .
SOURCE      Human respiratory syncytial virus A
  ORGANISM  Human respiratory syncytial virus A
            Viruses; ssRNA viruses; ssRNA negative-strand viruses;
            Mononegavirales; Pneumoviridae; Orthopneumovirus.
REFERENCE   1  (bases 1 to 651)
  AUTHORS   Thongpan,I., Mauleekoonphairoj,J., Vichiwattana,P., Korkong,S.,
            Vongpunsawad,S. and Poovorawan,Y.
  TITLE     Molecular Characterization of Respiratory Syncytial Virus in
            Thailand, 2012-2015
  JOURNAL   Unpublished
REFERENCE   2  (bases 1 to 651)
  AUTHORS   Thongpan,I., Mauleekoonphairoj,J., Vichiwattana,P., Korkong,S.,
            Vongpunsawad,S. and Poovorawan,Y.
  TITLE     Direct Submission
  JOURNAL   Submitted (13-DEC-2016) Department of Pediatrics, Center of
            Excellence in Clinical Virology, Faculty of Medicine, Chulalongkorn
            University, Bangkok 10330, Thailand
COMMENT     ##Assembly-Data-START##
            Assembly Method       :: DNASTAR-Lasergene v. 6
            Sequencing Technology :: Sanger dideoxy sequencing
            ##Assembly-Data-END##
FEATURES             Location/Qualifiers
```

```
     source          1..651
                     /organism="Human respiratory syncytial virus A"
                     /mol_type="viral cRNA"
                     /isolate="TH-CU457/2012"
                     /host="Homo sapiens"
                     /db_xref="taxon:208893"
                     /country="Thailand"
                     /collection_date="17-Sep-2012"
                     /note="group: A"
     CDS             <1..651
                     /note="G protein"
                     /codon_start=1
                     /product="attachment glycoprotein"
                     /protein_id="APY20348"
                     /translation="GTTSQSTTILASTTPSAESTPQSTTVKIKNTTTTQILPSKPTTK
                     QRQNKPQNKPNNDFHFEVFNFVPCSICSNNPTCWAICKRIPNKKPGKKTTTKPTKKPT
                     LKTTKKDPKPQTTKPKEVLTTKPTGKPTINTTKTNIRTTLLTSNTKGNPEHTSQEETL
                     HSTTSEGYLSPSQVYTTSGQEETLHSTTSKGYLSPSQVYTTSEYLSQSLSSSNTTK"
ORIGIN
        1 ggaactacat cacaatccac caccatacta gcttcaacaa caccaagtgc tgagtcaacc
       61 ccacaatcca caacagtcaa gatcaaaaac acaacaacaa cccaaatatt acctagcaaa
      121 cccaccacaa aacaacgcca aaataaacca caaaacaaac ccaacaatga ttttcacttt
      181 gaagtgttca attttgtacc ctgcagcata tgcagcaaca atccaacctg ctgggccatc
      241 tgcaagagaa taccaaacaa aaaacctgga aagaaaacca ccaccaagcc cacaaaaaaa
      301 ccaaccctca agacaaccaa aaaagatccc aaaccccaaa ccacaaaacc aaaggaagta
      361 ctcactacca agcctacagg aaagccaacc atcaacacca ctaaaacaaa catcagaact
      421 acactgctca cctccaacac caaaggaaat ccagaacaca caagtcaaga ggaaaccctc
      481 cactcaacca cctccgaagg ctatctaagc ccatcacaag tctatacaac atccggtcaa
      541 gaggaaaccc tccactcaac cacctccaaa ggctatctaa gcccatcaca agtctataca
      601 acatccgagt acctatcaca atctctatct tcatccaaca caacaaaatg a
//
LOCUS       KY328008                 651 bp    cRNA    linear   VRL 12-DEC-2016
DEFINITION  Human respiratory syncytial virus A isolate TH-CU456/2012
            attachment glycoprotein gene, partial cds.
ACCESSION   KY328008
VERSION     KY328008
KEYWORDS    .
SOURCE      Human respiratory syncytial virus A
  ORGANISM  Human respiratory syncytial virus A
            Viruses; ssRNA viruses; ssRNA negative-strand viruses;
            Mononegavirales; Pneumoviridae; Orthopneumovirus.
REFERENCE   1  (bases 1 to 651)
  AUTHORS   Thongpan,I., Mauleekoonphairoj,J., Vichiwattana,P., Korkong,S.,
            Vongpunsawad,S. and Poovorawan,Y.
  TITLE     Molecular Characterization of Respiratory Syncytial Virus in
            Thailand, 2012-2015
  JOURNAL   Unpublished
REFERENCE   2  (bases 1 to 651)
  AUTHORS   Thongpan,I., Mauleekoonphairoj,J., Vichiwattana,P., Korkong,S.,
            Vongpunsawad,S. and Poovorawan,Y.
  TITLE     Direct Submission
  JOURNAL   Submitted (13-DEC-2016) Department of Pediatrics, Center of
            Excellence in Clinical Virology, Faculty of Medicine, Chulalongkorn
            University, Bangkok 10330, Thailand
```

```
COMMENT     ##Assembly-Data-START##
            Assembly Method       :: DNASTAR-Lasergene v. 6
            Sequencing Technology :: Sanger dideoxy sequencing
            ##Assembly-Data-END##
FEATURES             Location/Qualifiers
     source          1..651
                     /organism="Human respiratory syncytial virus A"
                     /mol_type="viral cRNA"
                     /isolate="TH-CU456/2012"
                     /host="Homo sapiens"
                     /db_xref="taxon:208893"
                     /country="Thailand"
                     /collection_date="17-Sep-2012"
                     /note="group: A"
     CDS             <1..651
                     /note="G protein"
                     /codon_start=1
                     /product="attachment glycoprotein"
                     /protein_id="APY20349"
                     /translation="GTTSQSTTILASTTPSAESTPQSTTVKIKNTTTTQILPSKPTTK
                     QRQNKPQNKPNNDFHFEVFNFVPCSICSNNPTCWAICKRIPNKKPGKKTTTKPTKKPT
                     LKTTKKDPKPQTTKPKEVLTTKPTGKPTINTTKTNIRTTLLTSNTKGNPEHTSQEETL
                     HSTTSEGYLSPSQVYTTSGQEETLHSTTSKGYLSPSQVYTTSEYLSQSLSSSNTTK"
ORIGIN
        1 ggaactacat cacaatccac caccatacta gcttcaacaa caccaagtgc tgagtcaacc
       61 ccacaatcca caacagtcaa gatcaaaaac acaacaacaa cccaaatatt acctagcaaa
      121 cccaccacaa aacaacgcca aaataaacca caaaacaaac ccaacaatga ttttcacttt
      181 gaagtgttca attttgtacc ctgcagcata tgcagcaaca atccaacctg ctgggccatc
      241 tgcaagagaa taccaaacaa aaaacctgga aagaaaacca ccaccaagcc cacaaaaaaa
      301 ccaaccctca agacaaccaa aaaagatccc aaacccccaaa ccacaaaacc aaaggaagta
      361 ctcactacca agcctacagg aaagccaacc atcaacacca ctaaaacaaa catcagaact
      421 acactgctca cctccaacac caaaggaaat ccagaacaca caagtcaaga ggaaaccctc
      481 cactcaacca cctccgaagg ctatctaagc ccatcacaag tctatacaac atccggtcaa
      541 gaggaaaccc tccactcaac cacctccaaa ggctatctaa gcccatcaca ggtctataca
      601 acatccgagt acctatcaca atctctatct tcatccaaca caacaaaatg a
//
LOCUS       KY328009                 651 bp    cRNA    linear   VRL 12-DEC-2016
DEFINITION  Human respiratory syncytial virus A isolate TH-CU461/2012
            attachment glycoprotein gene, partial cds.
ACCESSION   KY328009
VERSION     KY328009
KEYWORDS    .
SOURCE      Human respiratory syncytial virus A
  ORGANISM  Human respiratory syncytial virus A
            Viruses; ssRNA viruses; ssRNA negative-strand viruses;
            Mononegavirales; Pneumoviridae; Orthopneumovirus.
REFERENCE   1  (bases 1 to 651)
  AUTHORS   Thongpan,I., Mauleekoonphairoj,J., Vichiwattana,P., Korkong,S.,
            Vongpunsawad,S. and Poovorawan,Y.
  TITLE     Molecular Characterization of Respiratory Syncytial Virus in
            Thailand, 2012-2015
  JOURNAL   Unpublished
REFERENCE   2  (bases 1 to 651)
  AUTHORS   Thongpan,I., Mauleekoonphairoj,J., Vichiwattana,P., Korkong,S.,
```

Vongpunsawad,S. and Poovorawan,Y.
TITLE Direct Submission
JOURNAL Submitted (13-DEC-2016) Department of Pediatrics, Center of
Excellence in Clinical Virology, Faculty of Medicine, Chulalongkorn
University, Bangkok 10330, Thailand
COMMENT ##Assembly-Data-START##
Assembly Method :: DNASTAR-Lasergene v. 6
Sequencing Technology :: Sanger dideoxy sequencing
##Assembly-Data-END##
FEATURES Location/Qualifiers
source 1..651
/organism="Human respiratory syncytial virus A"
/mol_type="viral cRNA"
/isolate="TH-CU461/2012"
/host="Homo sapiens"
/db_xref="taxon:208893"
/country="Thailand"
/collection_date="17-Sep-2012"
/note="group: A"
CDS <1..651
/note="G protein"
/codon_start=1
/product="attachment glycoprotein"
/protein_id="APY20350"
/translation="GTTSQSTTILASTTPSAESTPQSTTVKIKNTTTTQILPSKPTTK
QRQNKPQNKPNNDFHFEVFNFVPCSICSNNPTCWAICKRIPNKKPGKKTTTKPTKKPT
LKTTKKDPKPQTTKPKEVLTTKPTGKPTINTTKTNIRTTLLTSNTKGNPEHTSQEETL
HSTASEGYLSPSQVYTTSGQEETLHSTTSKGYLSPSQVYTTSEYLSQSLSSSNTTK"
ORIGIN
1 ggaactacat cacaatccac caccatacta gcttcaacaa caccaagtgc tgagtcaacc
61 ccacaatcca caacagtcaa gatcaaaaac acaacaacaa cccaaatatt acctagcaaa
121 cccaccacaa aacaacgcca aaataaacca caaaacaaac ccaacaatga ttttcacttt
181 gaagtgttca attttgtacc ctgcagcata tgcagcaaca atccaacctg ctgggccatc
241 tgcaagagaa taccaaacaa aaaacctgga aagaaaacca ccaccaagcc cacaaaaaaa
301 ccaaccctca agacaaccaa aaaagatccc aaaccccaaa ccacaaaacc aaaggaagta
361 ctcactacca agcctacagg aaagccaacc atcaacacca ctaaaacaaa catcagaact
421 acactgctca cctccaacac caaaggaaat ccagaacaca caagtcaaga ggaaaccctc
481 cactcaaccg cctccgaagg ctatctaagc ccatcacaag tctatacaac atccggtcaa
541 gaggaaaccc tccactcaac cacctccaaa ggctatctaa gcccatcaca agtctataca
601 acatccgagt acctatcaca atctctatct tcatccaaca caacaaaatg a
//
LOCUS KY328010 651 bp cRNA linear VRL 12-DEC-2016
DEFINITION Human respiratory syncytial virus A isolate TH-CU/CB117/2013
attachment glycoprotein gene, partial cds.
ACCESSION KY328010
VERSION KY328010
KEYWORDS .
SOURCE Human respiratory syncytial virus A
ORGANISM Human respiratory syncytial virus A
Viruses; ssRNA viruses; ssRNA negative-strand viruses;
Mononegavirales; Pneumoviridae; Orthopneumovirus.
REFERENCE 1 (bases 1 to 651)
AUTHORS Thongpan,I., Mauleekoonphairoj,J., Vichiwattana,P., Korkong,S.,
Vongpunsawad,S. and Poovorawan,Y.

```
  TITLE     Molecular Characterization of Respiratory Syncytial Virus in
            Thailand, 2012-2015
  JOURNAL   Unpublished
REFERENCE   2  (bases 1 to 651)
  AUTHORS   Thongpan,I., Mauleekoonphairoj,J., Vichiwattana,P., Korkong,S.,
            Vongpunsawad,S. and Poovorawan,Y.
  TITLE     Direct Submission
  JOURNAL   Submitted (13-DEC-2016) Department of Pediatrics, Center of
            Excellence in Clinical Virology, Faculty of Medicine, Chulalongkorn
            University, Bangkok 10330, Thailand
COMMENT     ##Assembly-Data-START##
            Assembly Method       :: DNASTAR-Lasergene v. 6
            Sequencing Technology :: Sanger dideoxy sequencing
            ##Assembly-Data-END##
FEATURES             Location/Qualifiers
     source          1..651
                     /organism="Human respiratory syncytial virus A"
                     /mol_type="viral cRNA"
                     /isolate="TH-CU/CB117/2013"
                     /host="Homo sapiens"
                     /db_xref="taxon:208893"
                     /country="Thailand"
                     /collection_date="02-Nov-2013"
                     /note="group: A"
     CDS             <1..651
                     /note="G protein"
                     /codon_start=1
                     /product="attachment glycoprotein"
                     /protein_id="APY20351"
                     /translation="GTTSQSTTILASTTPSAESTPQSTTVKIKNTTTTQILPSKPTTK
                     QRQNKPQNKPNNDFHFEVFNFVPCSICSNNPTCWAICKRIPNKKPGKKTTTKPTKKPT
                     LKTTKKDPKPQTTKPKEVLTTKPTGKPTINTTKTNIRTTLLTSNTKGNPEHTSQEGTL
                     HSTTSEGYLSPSQVYTISGQEETLHSTTSKGYLSPSQVYTTSEYLSQSLSSSNTTK"
ORIGIN
        1 ggaactacat cacaatccac caccatacta gcttcaacaa caccaagtgc tgagtcaacc
       61 ccacaatcca caacagtcaa gatcaaaaac acaacaacaa cccaaatatt acctagcaaa
      121 cccaccacaa aacaacgcca aaataaacca caaaacaaac ccaacaatga ttttcacttt
      181 gaagtgttca attttgtacc ctgcagcata tgcagcaaca atccaacctg ctgggccatc
      241 tgcaagagaa taccaaacaa aaaacctgga aagaaaacca ccaccaagcc cacaaaaaaa
      301 ccaaccctca agacaaccaa aaaagatccc aaaccccaaa ccacaaaacc aaaggaagta
      361 ctcactacca agcctacagg aaagccaacc atcaacacca ctaaaacaaa catcagaact
      421 acactgctca cctccaacac caaaggaaat ccagaacaca caagtcaaga gggaaccctc
      481 cactcaacca cctccgaagg ctatctaagc ccatcacaag tctatacaat atccggtcaa
      541 gaggaaaccc tccactcaac cacctccaaa ggctatctaa gcccatcaca agtctataca
      601 acatccgagt acctatcaca atctctatct tcatccaaca caacaaaatg a
//
LOCUS       KY328011                 651 bp    cRNA    linear   VRL 12-DEC-2016
DEFINITION  Human respiratory syncytial virus A isolate TH-CU/B12451/2015
            attachment glycoprotein gene, partial cds.
ACCESSION   KY328011
VERSION     KY328011
KEYWORDS    .
SOURCE      Human respiratory syncytial virus A
  ORGANISM  Human respiratory syncytial virus A
```

```
            Viruses; ssRNA viruses; ssRNA negative-strand viruses;
            Mononegavirales; Pneumoviridae; Orthopneumovirus.
REFERENCE   1  (bases 1 to 651)
  AUTHORS   Thongpan,I., Mauleekoonphairoj,J., Vichiwattana,P., Korkong,S.,
            Vongpunsawad,S. and Poovorawan,Y.
  TITLE     Molecular Characterization of Respiratory Syncytial Virus in
            Thailand, 2012-2015
  JOURNAL   Unpublished
REFERENCE   2  (bases 1 to 651)
  AUTHORS   Thongpan,I., Mauleekoonphairoj,J., Vichiwattana,P., Korkong,S.,
            Vongpunsawad,S. and Poovorawan,Y.
  TITLE     Direct Submission
  JOURNAL   Submitted (13-DEC-2016) Department of Pediatrics, Center of
            Excellence in Clinical Virology, Faculty of Medicine, Chulalongkorn
            University, Bangkok 10330, Thailand
COMMENT     ##Assembly-Data-START##
            Assembly Method       :: DNASTAR-Lasergene v. 6
            Sequencing Technology :: Sanger dideoxy sequencing
            ##Assembly-Data-END##
FEATURES             Location/Qualifiers
     source          1..651
                     /organism="Human respiratory syncytial virus A"
                     /mol_type="viral cRNA"
                     /isolate="TH-CU/B12451/2015"
                     /host="Homo sapiens"
                     /db_xref="taxon:208893"
                     /country="Thailand"
                     /collection_date="01-Aug-2015"
                     /note="group: A"
     CDS             <1..651
                     /note="G protein"
                     /codon_start=1
                     /product="attachment glycoprotein"
                     /protein_id="APY20352"
                     /translation="GTTSQSTTILASTTPSAESTPQSTTVKIKNTTTTQILPSKPTTK
                     QRQNKPQNKPNNDFHFEVFNFVPCSICSNNPTCWAICKRIPNKKPGKKTTTKPTKKPT
                     LKTTKKDPKPQTTKPKEVLTTKPTGKPTINTTKTNIRTTLLTSNTKGNPEHTSQEETL
                     HSTTSEGYLSPSQVYTTSGQEETLHSTTSEGHPRPSQVYTTSEYLSQSLSSSNTTK"
ORIGIN
        1 ggaactacat cacaatccac caccatacta gcttcaacaa caccaagtgc tgagtcaacc
       61 ccacaatcca caacagtcaa gatcaaaaac acaacaacaa cccaaatatt acctagcaaa
      121 cccaccacaa aacaacgcca aaataaacca caaaacaaac ccaacaatga ttttcacttt
      181 gaagtgttca attttgtacc ctgcagcata tgcagcaaca atccaacctg ctgggccatc
      241 tgcaagagaa taccaaacaa aaaacctgga aagaaaacca ccaccaagcc cacaaaaaaa
      301 ccaaccctca agacaaccaa aaaagatccc aaacctcaga ccacaaaacc aaaggaagta
      361 ctcactacca agcctacagg aaagccaacc atcaacacca ctaaaacaaa catcagaact
      421 acactgctca cctccaacac caaaggaaat ccagaacaca caagtcaaga ggaaaccctc
      481 cactcaacca cctccgaagg ctatctaagc ccatcccaag tctatacaac atccggtcaa
      541 gaggaaaccc tccactcaac cacctccgaa ggccatccaa gaccatcaca agtctataca
      601 acatccgagt acctatcaca atctctatct tcatccaaca caacaaaatg a
//
LOCUS       KY328012                 651 bp    cRNA    linear   VRL 12-DEC-2016
DEFINITION  Human respiratory syncytial virus A isolate TH-CU/C5161/2014
            attachment glycoprotein gene, partial cds.
```

```
ACCESSION   KY328012
VERSION     KY328012
KEYWORDS    .
SOURCE      Human respiratory syncytial virus A
  ORGANISM  Human respiratory syncytial virus A
            Viruses; ssRNA viruses; ssRNA negative-strand viruses;
            Mononegavirales; Pneumoviridae; Orthopneumovirus.
REFERENCE   1  (bases 1 to 651)
  AUTHORS   Thongpan,I., Mauleekoonphairoj,J., Vichiwattana,P., Korkong,S.,
            Vongpunsawad,S. and Poovorawan,Y.
  TITLE     Molecular Characterization of Respiratory Syncytial Virus in
            Thailand, 2012-2015
  JOURNAL   Unpublished
REFERENCE   2  (bases 1 to 651)
  AUTHORS   Thongpan,I., Mauleekoonphairoj,J., Vichiwattana,P., Korkong,S.,
            Vongpunsawad,S. and Poovorawan,Y.
  TITLE     Direct Submission
  JOURNAL   Submitted (13-DEC-2016) Department of Pediatrics, Center of
            Excellence in Clinical Virology, Faculty of Medicine, Chulalongkorn
            University, Bangkok 10330, Thailand
COMMENT     ##Assembly-Data-START##
            Assembly Method       :: DNASTAR-Lasergene v. 6
            Sequencing Technology :: Sanger dideoxy sequencing
            ##Assembly-Data-END##
FEATURES             Location/Qualifiers
     source          1..651
                     /organism="Human respiratory syncytial virus A"
                     /mol_type="viral cRNA"
                     /isolate="TH-CU/C5161/2014"
                     /host="Homo sapiens"
                     /db_xref="taxon:208893"
                     /country="Thailand"
                     /collection_date="08-Oct-2014"
                     /note="group: A"
     CDS             <1..651
                     /note="G protein"
                     /codon_start=1
                     /product="attachment glycoprotein"
                     /protein_id="APY20353"
                     /translation="GTTSQSTTILASTTPSAESTPQSTTVKIINTTTTQILPSKPTTK
                     QRQNKPQNKPNNDFHFEVFNFVPCSICSNNPTCWAICKRIPNKKPGKKTTTKPTKKPT
                     LKTTKKDPKPQTTKPKEVLTTKPTGKPTINTTKTNIRTTLIISNTKGNPEHTSQKETL
                     HSTTSEGYLSPSQVYTTSGQEETLHSTTSEGYLSPSQVYTTSEYLSQSLSSSNTTK"
ORIGIN
        1 ggaactacat cacaatccac caccatacta gcttcaacaa caccaagtgc tgagtcaacc
       61 ccacaatcca caacagtcaa gatcataaac acaacaacaa cccaaatatt acctagcaaa
      121 cccaccacaa aacaacgcca aaataaacca caaaacaaac ccaacaatga ttttcacttt
      181 gaagtgttca attttgtacc ctgcagcata tgcagcaaca atccaacctg ctgggccatc
      241 tgcaagagaa taccgaacaa aaaacctgga aagaaaacca ccaccaagcc cacaaaaaaa
      301 ccaaccctca agacaaccaa aaaagatccc aaacctcaaa ccacaaaacc aaaggaagta
      361 ctcactacca agcctacagg aaagccaacc atcaacacca ctaaaacaaa catcagaact
      421 acactgatca tctctaacac caaaggaaat ccagaacaca caagtcaaaa ggaaaccctc
      481 cactcaacca cctccgaagg ctatctaagc ccatcacaag tctatacaac atccggtcaa
      541 gaggaaaccc tccactcaac cacctccgaa ggctatctaa gcccatcaca agtctataca
```

601 acatccgagt acctatcaca atctctatct tcatccaaca caacaaaatg a
//
LOCUS   KY328013   651 bp   cRNA   linear   VRL 12-DEC-2016
DEFINITION Human respiratory syncytial virus A isolate TH-CU/B11007/2014
            attachment glycoprotein gene, partial cds.
ACCESSION   KY328013
VERSION   KY328013
KEYWORDS   .
SOURCE   Human respiratory syncytial virus A
  ORGANISM Human respiratory syncytial virus A
            Viruses; ssRNA viruses; ssRNA negative-strand viruses;
            Mononegavirales; Pneumoviridae; Orthopneumovirus.
REFERENCE   1 (bases 1 to 651)
  AUTHORS   Thongpan,I., Mauleekoonphairoj,J., Vichiwattana,P., Korkong,S.,
            Vongpunsawad,S. and Poovorawan,Y.
  TITLE   Molecular Characterization of Respiratory Syncytial Virus in
            Thailand, 2012-2015
  JOURNAL   Unpublished
REFERENCE   2 (bases 1 to 651)
  AUTHORS   Thongpan,I., Mauleekoonphairoj,J., Vichiwattana,P., Korkong,S.,
            Vongpunsawad,S. and Poovorawan,Y.
  TITLE   Direct Submission
  JOURNAL   Submitted (13-DEC-2016) Department of Pediatrics, Center of
            Excellence in Clinical Virology, Faculty of Medicine, Chulalongkorn
            University, Bangkok 10330, Thailand
COMMENT   ##Assembly-Data-START##
            Assembly Method   :: DNASTAR-Lasergene v. 6
            Sequencing Technology :: Sanger dideoxy sequencing
            ##Assembly-Data-END##
FEATURES   Location/Qualifiers
     source   1..651
                     /organism="Human respiratory syncytial virus A"
                     /mol_type="viral cRNA"
                     /isolate="TH-CU/B11007/2014"
                     /host="Homo sapiens"
                     /db_xref="taxon:208893"
                     /country="Thailand"
                     /collection_date="22-Sep-2014"
                     /note="group: A"
     CDS   <1..651
                     /note="G protein"
                     /codon_start=1
                     /product="attachment glycoprotein"
                     /protein_id="APY20354"
                     /translation="GTTSQSTTILASTTPSAESTPQSTTVKIINTTTTQISPSKPTTK
                     QRQNKPQNKPNNDFHFEVFNFVPCSICSNNPTCWAICKRIPNKKPGKKTTTKPTKKPT
                     LKTTKKDPKPQTTKPKEVLTTKPTGKPTINTTKTNIRTTLIISNTKGNPEHTSQKETL
                     HSTTSEGYLSPSQVYTTSGQEETLHSTTSEGYLSPSQVYTTSEYLSQSLSSSNTTK"
ORIGIN
        1 ggaactacat cacaatccac caccatacta gcttcaacaa caccaagtgc cgagtcaacc
       61 ccacaatcca caacagtcaa gatcataaac acaacaacaa cccaaatatc acccagcaaa
      121 cccaccacaa aacaacgcca aaacaaacca caaaacaaac ccaacaatga ttttcacttt
      181 gaagtgttca attttgtacc ctgcagcata tgcagcaaca atccaacctg ctgggccatc
      241 tgcaagagaa taccaaacaa aaaacctgga aagaaaacca ccaccaagcc cacaaaaaaa

```
     301 ccaaccctca agacaaccaa aaaagatccc aaacctcaaa ccacaaaacc aaaggaagta
     361 ctcactacca agcctacagg aaagccaacc atcaacacca ctaaaacaaa catcagaact
     421 acactgatca tctctaacac caaaggaaat ccagaacaca caagtcaaaa ggaaaccctc
     481 cactcaacca cctccgaagg ctatctaagc ccatcacaag tctatacaac atccggtcaa
     541 gaggaaaccc tccactcaac cacctccgaa ggctatctaa gcccatcaca agtctataca
     601 acatccgagt acctatcaca atctctatct tcatccaaca caacaaaatg a
//
LOCUS       KY328014               651 bp    cRNA    linear   VRL 12-DEC-2016
DEFINITION  Human respiratory syncytial virus A isolate TH-CU/B10627/2014
            attachment glycoprotein gene, partial cds.
ACCESSION   KY328014
VERSION     KY328014
KEYWORDS    .
SOURCE      Human respiratory syncytial virus A
  ORGANISM  Human respiratory syncytial virus A
            Viruses; ssRNA viruses; ssRNA negative-strand viruses;
            Mononegavirales; Pneumoviridae; Orthopneumovirus.
REFERENCE   1  (bases 1 to 651)
  AUTHORS   Thongpan,I., Mauleekoonphairoj,J., Vichiwattana,P., Korkong,S.,
            Vongpunsawad,S. and Poovorawan,Y.
  TITLE     Molecular Characterization of Respiratory Syncytial Virus in
            Thailand, 2012-2015
  JOURNAL   Unpublished
REFERENCE   2  (bases 1 to 651)
  AUTHORS   Thongpan,I., Mauleekoonphairoj,J., Vichiwattana,P., Korkong,S.,
            Vongpunsawad,S. and Poovorawan,Y.
  TITLE     Direct Submission
  JOURNAL   Submitted (13-DEC-2016) Department of Pediatrics, Center of
            Excellence in Clinical Virology, Faculty of Medicine, Chulalongkorn
            University, Bangkok 10330, Thailand
COMMENT     ##Assembly-Data-START##
            Assembly Method       :: DNASTAR-Lasergene v. 6
            Sequencing Technology :: Sanger dideoxy sequencing
            ##Assembly-Data-END##
FEATURES             Location/Qualifiers
     source          1..651
                     /organism="Human respiratory syncytial virus A"
                     /mol_type="viral cRNA"
                     /isolate="TH-CU/B10627/2014"
                     /host="Homo sapiens"
                     /db_xref="taxon:208893"
                     /country="Thailand"
                     /collection_date="09-Aug-2014"
                     /note="group: A"
     CDS             <1..651
                     /note="G protein"
                     /codon_start=1
                     /product="attachment glycoprotein"
                     /protein_id="APY20355"
                     /translation="GTTSQSTTILASTTPSAESTPQSTTVKIINTTTTQILPSKPTTK
                     QRQNKPQNKPNNDFHFEVFNFVPCSICSNNPTCWAICKRIPNKKPGKKTTTKPTKKPT
                     LKTTKKDPKPQTTKPKEVLTTKPTGKPTINTTKTNIRTTLIISNTKGNPEHTSQKETL
                     HSTTSEGYLSPSQVYTTSGQEETLHSTTSEGYLSPSQVYTTSEYLSQSLSSSNTTK"
ORIGIN
```

```
      1 ggaactacat cacaatccac caccatacta gcttcaacaa caccaagtgc tgagtcaacc
     61 ccacaatcca caacagtcaa gatcataaac acaacaacaa cccaaatatt acctagcaaa
    121 cccaccacaa aacaacgcca aaataaacca caaaacaaac ccaacaatga ttttcacttt
    181 gaagtgttca attttgtacc ctgcagcata tgcagcaaca atccaacctg ctgggccatc
    241 tgcaagagaa taccaaacaa aaaacctgga aagaaaacca ccaccaagcc cacaaaaaaa
    301 ccaaccctca agacaaccaa aaaagatccc aaacctcaaa ccacaaaacc aaaggaagta
    361 ctcactacca agcctacagg aaagccaacc atcaacacca ctaaaacaaa catcagaact
    421 acactgatca tctctaacac caaaggaaat ccagaacaca caagtcaaaa ggaaaccctc
    481 cactcaacca cctccgaagg ctatctaagc ccatcacaag tctatacaac atccggtcaa
    541 gaggaaaccc tccactcaac cacctccgaa ggctatctaa gcccatcaca agtctataca
    601 acatccgagt acctatcaca atctctatct tcatccaaca caacaaaatg a
//
LOCUS       KY328015                 651 bp    cRNA    linear   VRL 12-DEC-2016
DEFINITION  Human respiratory syncytial virus A isolate TH-CU/B10626/2014
            attachment glycoprotein gene, partial cds.
ACCESSION   KY328015
VERSION     KY328015
KEYWORDS    .
SOURCE      Human respiratory syncytial virus A
  ORGANISM  Human respiratory syncytial virus A
            Viruses; ssRNA viruses; ssRNA negative-strand viruses;
            Mononegavirales; Pneumoviridae; Orthopneumovirus.
REFERENCE   1  (bases 1 to 651)
  AUTHORS   Thongpan,I., Mauleekoonphairoj,J., Vichiwattana,P., Korkong,S.,
            Vongpunsawad,S. and Poovorawan,Y.
  TITLE     Molecular Characterization of Respiratory Syncytial Virus in
            Thailand, 2012-2015
  JOURNAL   Unpublished
REFERENCE   2  (bases 1 to 651)
  AUTHORS   Thongpan,I., Mauleekoonphairoj,J., Vichiwattana,P., Korkong,S.,
            Vongpunsawad,S. and Poovorawan,Y.
  TITLE     Direct Submission
  JOURNAL   Submitted (13-DEC-2016) Department of Pediatrics, Center of
            Excellence in Clinical Virology, Faculty of Medicine, Chulalongkorn
            University, Bangkok 10330, Thailand
COMMENT     ##Assembly-Data-START##
            Assembly Method       :: DNASTAR-Lasergene v. 6
            Sequencing Technology :: Sanger dideoxy sequencing
            ##Assembly-Data-END##
FEATURES             Location/Qualifiers
     source          1..651
                     /organism="Human respiratory syncytial virus A"
                     /mol_type="viral cRNA"
                     /isolate="TH-CU/B10626/2014"
                     /host="Homo sapiens"
                     /db_xref="taxon:208893"
                     /country="Thailand"
                     /collection_date="09-Aug-2014"
                     /note="group: A"
     CDS             <1..651
                     /note="G protein"
                     /codon_start=1
                     /product="attachment glycoprotein"
                     /protein_id="APY20356"
```

/translation="GTTSQSTTILASTTPSAESTPQSTTVKIINTTTTQILPSKPTTK
QRQNKPQNKPNNDFHFEVFNFVPCSICSNNPTCWAICKRIPNKKPGKKTTTKPTKKPT
LKTTKKDPKPQTTKPKEVLTTKPTGKPTINTTKTNIRTTLIISNTKGNPEHTSQKETL
HSTTSEGYLSPSQVYTTSGQEETLHSTTSEGYLSPSQVYTTSEYLSQSLSSSNTTK"
ORIGIN
1 ggaactacat cacaatccac caccatacta gcttcaacaa caccaagtgc tgagtcaacc
61 ccacaatcca caacagtcaa gatcataaac acaacaacaa cccaaatatt acctagcaaa
121 cccaccacaa aacaacgcca aaataaacca caaaacaaac ccaacaatga ttttcacttt
181 gaagtgttca attttgtacc ctgcagcata tgcagcaaca atccaacctg ctgggccatc
241 tgcaagagaa taccaaacaa aaaacctgga aagaaaacca ccaccaagcc cacaaaaaaa
301 ccaaccctca agacaaccaa aaaagatccc aaacctcaaa ccacaaaacc aaaggaagta
361 ctcactacca agcctacagg aaagccaacc atcaacacca ctaaaacaaa catcagaact
421 acactgatca tctctaacac caaaggaaat ccagaacaca caagtcaaaa ggaaaccctc
481 cactcaacca cctccgaagg ctatctaagc ccatcacaag tctatacaac atccggtcaa
541 gaggaaaccc tccactcaac cacctccgaa ggctatctaa gcccatcaca agtctataca
601 acatccgagt acctatcaca atctctatct tcatccaaca caacaaaatg a
//
LOCUS KY328016 651 bp cRNA linear VRL 12-DEC-2016
DEFINITION Human respiratory syncytial virus A isolate TH-CU/C6282/2015
attachment glycoprotein gene, partial cds.
ACCESSION KY328016
VERSION KY328016
KEYWORDS .
SOURCE Human respiratory syncytial virus A
ORGANISM Human respiratory syncytial virus A
Viruses; ssRNA viruses; ssRNA negative-strand viruses;
Mononegavirales; Pneumoviridae; Orthopneumovirus.
REFERENCE 1 (bases 1 to 651)
AUTHORS Thongpan,I., Mauleekoonphairoj,J., Vichiwattana,P., Korkong,S.,
Vongpunsawad,S. and Poovorawan,Y.
TITLE Molecular Characterization of Respiratory Syncytial Virus in
Thailand, 2012-2015
JOURNAL Unpublished
REFERENCE 2 (bases 1 to 651)
AUTHORS Thongpan,I., Mauleekoonphairoj,J., Vichiwattana,P., Korkong,S.,
Vongpunsawad,S. and Poovorawan,Y.
TITLE Direct Submission
JOURNAL Submitted (13-DEC-2016) Department of Pediatrics, Center of
Excellence in Clinical Virology, Faculty of Medicine, Chulalongkorn
University, Bangkok 10330, Thailand
COMMENT ##Assembly-Data-START##
Assembly Method :: DNASTAR-Lasergene v. 6
Sequencing Technology :: Sanger dideoxy sequencing
##Assembly-Data-END##
FEATURES Location/Qualifiers
source 1..651
/organism="Human respiratory syncytial virus A"
/mol_type="viral cRNA"
/isolate="TH-CU/C6282/2015"
/host="Homo sapiens"
/db_xref="taxon:208893"
/country="Thailand"
/collection_date="25-Nov-2015"
/note="group: A"

```
     CDS             <1..651
                     /note="G protein"
                     /codon_start=1
                     /product="attachment glycoprotein"
                     /protein_id="APY20357"
                     /translation="GTTSQSSTIRASTTPSAESTPQSTTVKIINTTTTQILPSKPTTK
                     QRQNKPQNKPNNDFHFEVFNFVPCSICSNNPTCWAICKRIPNKKPGKKTTTKPTKKPT
                     LKTTKKDPKPQTTKPKEVLTTKPTGKPTINTTKTNIRTTLLISNTKGNPEHTSQKETL
                     HSTTSEGYLSPSQVYTTSGQEETLHSTASEGYLSPSQVYTTSEYLSQSLSSSNTTK"
ORIGIN
        1 ggaactacat cacaatcctc caccatacga gcttcaacaa caccaagtgc tgagtcaacc
       61 ccacaatcca caacagtcaa gatcataaac acaacaacaa cccaaatatt acctagcaaa
      121 cccaccacaa aacaacgcca aaataaacca caaaacaaac ccaacaatga ttttcacttt
      181 gaagtgttca attttgtacc ctgcagcata tgcagtaaca atccaacctg ctgggccatc
      241 tgcaagagaa taccaaacaa aaaacctgga aagaaaacca ccaccaagcc cacaaaaaaa
      301 ccaaccctca aaacaaccaa aaaagatccc aaacctcaaa ccacaaaacc aaaggaagta
      361 ctcactacca agcctacagg aaagccaacc atcaacacca ctaaaacaaa catcagaact
      421 acactgctca tctctaacac caaaggaaat ccagaacaca caagtcaaaa ggaaaccctc
      481 cactcaacca cctccgaagg ctatctaagc ccatcacaag tctatacaac atccggtcaa
      541 gaggaaaccc tccactcaac cgcctccgaa ggctatctaa gcccatcaca agtctataca
      601 acatccgagt acctatcaca atctctatct tcatccaaca caacaaaatg a
//
LOCUS       KY328017                 651 bp    cRNA    linear   VRL 12-DEC-2016
DEFINITION  Human respiratory syncytial virus A isolate TH-CU/B12805/2015
            attachment glycoprotein gene, partial cds.
ACCESSION   KY328017
VERSION     KY328017
KEYWORDS    .
SOURCE      Human respiratory syncytial virus A
  ORGANISM  Human respiratory syncytial virus A
            Viruses; ssRNA viruses; ssRNA negative-strand viruses;
            Mononegavirales; Pneumoviridae; Orthopneumovirus.
REFERENCE   1  (bases 1 to 651)
  AUTHORS   Thongpan,I., Mauleekoonphairoj,J., Vichiwattana,P., Korkong,S.,
            Vongpunsawad,S. and Poovorawan,Y.
  TITLE     Molecular Characterization of Respiratory Syncytial Virus in
            Thailand, 2012-2015
  JOURNAL   Unpublished
REFERENCE   2  (bases 1 to 651)
  AUTHORS   Thongpan,I., Mauleekoonphairoj,J., Vichiwattana,P., Korkong,S.,
            Vongpunsawad,S. and Poovorawan,Y.
  TITLE     Direct Submission
  JOURNAL   Submitted (13-DEC-2016) Department of Pediatrics, Center of
            Excellence in Clinical Virology, Faculty of Medicine, Chulalongkorn
            University, Bangkok 10330, Thailand
COMMENT     ##Assembly-Data-START##
            Assembly Method       :: DNASTAR-Lasergene v. 6
            Sequencing Technology :: Sanger dideoxy sequencing
            ##Assembly-Data-END##
FEATURES             Location/Qualifiers
     source          1..651
                     /organism="Human respiratory syncytial virus A"
                     /mol_type="viral cRNA"
                     /isolate="TH-CU/B12805/2015"
```

/host="Homo sapiens"
/db_xref="taxon:208893"
/country="Thailand"
/collection_date="02-Sep-2015"
/note="group: A"
CDS <1..651
/note="G protein"
/codon_start=1
/product="attachment glycoprotein"
/protein_id="APY20358"
/translation="GTTSQSSTIRASTTPSAESTPQSTTVKIINTTTTQILPSKPTTK
QRQNKPQNKPNNDFHFEVFNFVPCSICSNNPTCWAICKRIPNKKPGKKTTTKPTKKPT
LKTTKKDPKPQTTKPKEVLTTKPTGKPTINTTKTNIRTTLLISNTKGNPEHTSQKETL
HSTTSEGYPSPSQVYTTSGQEETLHSTTSEGYLSPSQVYTTSEYLSQSLSSSNTTK"
ORIGIN
1 ggaactacat cacaatcctc caccatacga gcttcaacaa caccaagtgc tgagtcaacc
61 ccacaatcca caacagtcaa gatcataaac acaacaacaa cccaaatatt acctagcaaa
121 cccaccacaa aacaacgcca aaataaacca caaaacaaac ccaacaatga ttttcacttt
181 gaagtgttca attttgtacc ctgcagcata tgcagtaaca atccaacctg ttgggccatc
241 tgcaagagaa taccaaacaa aaaacctgga aagaaaacca ccaccaaacc cacaaaaaaa
301 ccaaccctca agacaaccaa aaaagatccc aaacctcaaa ccacaaaacc aaaggaagta
361 ctcactacca agcctacagg aaagccaacc atcaacacca ctaaaacaaa catcagaact
421 acactgctca tctctaacac caaaggaaat ccagaacaca caagtcaaaa ggaaaccctc
481 cactcaacca cctccgaagg ctatccaagc ccatcacaag tctacacaac atccggtcaa
541 gaggaaaccc tccactcaac cacctccgaa ggctatctaa gcccatcaca agtctataca
601 acatccgagt acctatcaca atctctatct tcatccaaca caacaaaatg a
//
LOCUS KY328018 651 bp cRNA linear VRL 12-DEC-2016
DEFINITION Human respiratory syncytial virus A isolate TH-CU/B12839/2015
attachment glycoprotein gene, partial cds.
ACCESSION KY328018
VERSION KY328018
KEYWORDS .
SOURCE Human respiratory syncytial virus A
ORGANISM Human respiratory syncytial virus A
Viruses; ssRNA viruses; ssRNA negative-strand viruses;
Mononegavirales; Pneumoviridae; Orthopneumovirus.
REFERENCE 1 (bases 1 to 651)
AUTHORS Thongpan,I., Mauleekoonphairoj,J., Vichiwattana,P., Korkong,S.,
Vongpunsawad,S. and Poovorawan,Y.
TITLE Molecular Characterization of Respiratory Syncytial Virus in
Thailand, 2012-2015
JOURNAL Unpublished
REFERENCE 2 (bases 1 to 651)
AUTHORS Thongpan,I., Mauleekoonphairoj,J., Vichiwattana,P., Korkong,S.,
Vongpunsawad,S. and Poovorawan,Y.
TITLE Direct Submission
JOURNAL Submitted (13-DEC-2016) Department of Pediatrics, Center of
Excellence in Clinical Virology, Faculty of Medicine, Chulalongkorn
University, Bangkok 10330, Thailand
COMMENT ##Assembly-Data-START##
Assembly Method :: DNASTAR-Lasergene v. 6
Sequencing Technology :: Sanger dideoxy sequencing
##Assembly-Data-END##

```
FEATURES             Location/Qualifiers
     source          1..651
                     /organism="Human respiratory syncytial virus A"
                     /mol_type="viral cRNA"
                     /isolate="TH-CU/B12839/2015"
                     /host="Homo sapiens"
                     /db_xref="taxon:208893"
                     /country="Thailand"
                     /collection_date="07-Sep-2015"
                     /note="group: A"
     CDS             <1..651
                     /note="G protein"
                     /codon_start=1
                     /product="attachment glycoprotein"
                     /protein_id="APY20359"
                     /translation="GTTSQSTTILASTTPSAESTPQSTTVKIINTTTTQILPSKPTTK
                     QRQNKPQNKPNNDFHFEVFNFVPCSICSNNPTCWAICKRIPNKKPGKKTTTKPTKKPT
                     LKTTKKDPKPQTTKPKGVLTTKPTGKPTINTTKTNSRTTLLTSNTKGNPEHTSQKETL
                     HSTTSEGYLSPSQVYTTSGQEETLHSTTSEGYPSPSQVYTTSEYLSQSLSSSNTTK"
ORIGIN
        1 ggaactacat cacaatccac caccatacta gcttcaacaa caccaagtgc tgagtcaacc
       61 ccacaatcca caacagtcaa gatcataaac acaacaacaa cccaaatatt acctagcaaa
      121 cccaccacaa aacaacgcca aaataaacca caaaacaaac ccaacaatga ttttcacttt
      181 gaagtgttca attttgtacc ctgcagcata tgcagcaaca atccaacctg ctgggccatc
      241 tgcaagagaa taccaaacaa aaaacctgga aagaaaacca ccaccaagcc cacaaaaaaa
      301 ccaaccctca agacaaccaa aaaagatccc aaacctcaaa ccacaaaacc aaagggagta
      361 ctcactacca agcctacagg aaagccaacc atcaacacca ctaaaacaaa cagcagaact
      421 acactgctca cctccaacac caaaggaaat ccagaacaca caagtcaaaa ggaaaccctc
      481 cactcaacca cctccgaagg ctatctaagc ccatcacaag tctatacaac atccggtcaa
      541 gaggaaaccc tccactcaac cacctccgaa ggctatccaa gcccatcaca agtctataca
      601 acatccgagt acctatcaca atctctatct tcatccaaca caacaaaatg a
//
LOCUS       KY328019                 651 bp    cRNA    linear   VRL 12-DEC-2016
DEFINITION  Human respiratory syncytial virus A isolate TH-CU/B13046/2015
            attachment glycoprotein gene, partial cds.
ACCESSION   KY328019
VERSION     KY328019
KEYWORDS    .
SOURCE      Human respiratory syncytial virus A
  ORGANISM  Human respiratory syncytial virus A
            Viruses; ssRNA viruses; ssRNA negative-strand viruses;
            Mononegavirales; Pneumoviridae; Orthopneumovirus.
REFERENCE   1  (bases 1 to 651)
  AUTHORS   Thongpan,I., Mauleekoonphairoj,J., Vichiwattana,P., Korkong,S.,
            Vongpunsawad,S. and Poovorawan,Y.
  TITLE     Molecular Characterization of Respiratory Syncytial Virus in
            Thailand, 2012-2015
  JOURNAL   Unpublished
REFERENCE   2  (bases 1 to 651)
  AUTHORS   Thongpan,I., Mauleekoonphairoj,J., Vichiwattana,P., Korkong,S.,
            Vongpunsawad,S. and Poovorawan,Y.
  TITLE     Direct Submission
  JOURNAL   Submitted (13-DEC-2016) Department of Pediatrics, Center of
            Excellence in Clinical Virology, Faculty of Medicine, Chulalongkorn
```

```
            University, Bangkok 10330, Thailand
COMMENT     ##Assembly-Data-START##
            Assembly Method       :: DNASTAR-Lasergene v. 6
            Sequencing Technology :: Sanger dideoxy sequencing
            ##Assembly-Data-END##
FEATURES             Location/Qualifiers
     source          1..651
                     /organism="Human respiratory syncytial virus A"
                     /mol_type="viral cRNA"
                     /isolate="TH-CU/B13046/2015"
                     /host="Homo sapiens"
                     /db_xref="taxon:208893"
                     /country="Thailand"
                     /collection_date="21-Sep-2015"
                     /note="group: A"
     CDS             <1..651
                     /note="G protein"
                     /codon_start=1
                     /product="attachment glycoprotein"
                     /protein_id="APY20360"
                     /translation="GTTSQSTTILASTTPSAESTPQSTTVKIINTTTTQILPSKPTTK
                     QRQNKPQNKPNNDFHFEVFNFVPCSICSNNPTCWAICKRIPNKKPGKKTTTKPTKKPT
                     LKTTKKDPKPQTTKPKGVLTTKPTGKPTINTTKTNSRTTLLTSNTKGNPEHTSQKETL
                     HSTTSEGYLSPSQVYTTSGQEETLHSTTSEGYPSPSQVYTTSEYLSQSLSSSNTTK"
ORIGIN
        1 ggaactacat cacaatccac caccatacta gcttcaacaa caccaagtgc tgagtcaacc
       61 ccacaatcca caacagtcaa gatcataaac acaacaacaa cccaaatatt acctagcaaa
      121 cccaccacaa aacaacgcca aaataaacca caaaacaaac ccaacaatga ttttcacttt
      181 gaagtgttca attttgtacc ctgcagcata tgcagcaaca atccaacctg ctgggccatc
      241 tgcaagagaa taccaaacaa aaaacctgga aagaaaacca ccaccaagcc cacaaaaaaa
      301 ccaaccctca agacaaccaa aaaagatccc aaacctcaaa ccacaaaacc aaagggagta
      361 ctcactacca agcctacagg aaagccaacc atcaacacca ctaaaacaaa cagcagaact
      421 acactgctca cctccaacac caaaggaaat ccagaacaca caagtcaaaa ggaaaccctc
      481 cactcaacca cctccgaagg ctatctaagc ccatcacaag tctatacaac atccggtcaa
      541 gaggaaaccc tccactcaac cacctccgaa ggctatccaa gcccatcaca agtctataca
      601 acatccgagt acctatcaca atctctatct tcatccaaca caacaaaatg a
//
LOCUS       KY328020                 651 bp    cRNA    linear   VRL 12-DEC-2016
DEFINITION  Human respiratory syncytial virus A isolate ON1/TH-CU/B12560/2015
            attachment glycoprotein gene, partial cds.
ACCESSION   KY328020
VERSION     KY328020
KEYWORDS    .
SOURCE      Human respiratory syncytial virus A
  ORGANISM  Human respiratory syncytial virus A
            Viruses; ssRNA viruses; ssRNA negative-strand viruses;
            Mononegavirales; Pneumoviridae; Orthopneumovirus.
REFERENCE   1  (bases 1 to 651)
  AUTHORS   Thongpan,I., Mauleekoonphairoj,J., Vichiwattana,P., Korkong,S.,
            Vongpunsawad,S. and Poovorawan,Y.
  TITLE     Molecular Characterization of Respiratory Syncytial Virus in
            Thailand, 2012-2015
  JOURNAL   Unpublished
REFERENCE   2  (bases 1 to 651)
```

AUTHORS Thongpan,I., Mauleekoonphairoj,J., Vichiwattana,P., Korkong,S.,
Vongpunsawad,S. and Poovorawan,Y.
TITLE Direct Submission
JOURNAL Submitted (13-DEC-2016) Department of Pediatrics, Center of
Excellence in Clinical Virology, Faculty of Medicine, Chulalongkorn
University, Bangkok 10330, Thailand
COMMENT ##Assembly-Data-START##
Assembly Method :: DNASTAR-Lasergene v. 6
Sequencing Technology :: Sanger dideoxy sequencing
##Assembly-Data-END##
FEATURES Location/Qualifiers
source 1..651
/organism="Human respiratory syncytial virus A"
/mol_type="viral cRNA"
/isolate="ON1/TH-CU/B12560/2015"
/host="Homo sapiens"
/db_xref="taxon:208893"
/country="Thailand"
/collection_date="16-Aug-2015"
/note="group: A"
CDS <1..651
/note="G protein"
/codon_start=1
/product="attachment glycoprotein"
/protein_id="APY20361"
/translation="GTTSQSTTILASTTPSAESTPQSTTVKIINTTTTQILPSKPTTK
QRQNKPQNKPNNDFHFEVFNFVPCSICSNNPTCWAICKRIPNKKPGKKTTTKPTKKPT
LKTTKKDPKPQTTKPKEVLTTKPTGKPTINTTKTNSRTTLLTSNTKGNPEHTSQKETL
HSTTSEGYLSPSQVYTTSGQEETLHSTTSEGYPSPSQVYTTSEYLSQSLSSSNTTK"
ORIGIN
1 ggaactacat cacaatccac caccatacta gcttcaacaa caccaagtgc tgagtcaacc
61 ccacaatcca caacagtcaa gatcataaac acaacaacaa cccaaatatt acctagcaaa
121 cccaccacaa aacaacgcca aaataaacca caaaacaaac ccaacaatga ttttcacttt
181 gaagtgttca attttgtacc ctgcagcata tgtagcaaca atccaacctg ctgggccatc
241 tgcaagagaa taccaaacaa aaaacctgga aagaaaacca ccaccaagcc cacaaaaaaa
301 ccaaccctca agacaaccaa aaaagatccc aaacctcaaa ccacaaaacc aaaggaagta
361 ctcactacca agcctacagg aaagccaacc atcaacacca ctaaaacaaa cagcagaact
421 acactgctca cctccaacac caaaggaaat ccagaacaca caagtcaaaa ggaaaccctc
481 cactcaacca cctccgaagg ctatctaagc ccatcacaag tctatacaac atccggtcaa
541 gaggaaaccc tccactcaac cacctccgaa ggctatccaa gcccatcaca agtctataca
601 acatccgagt acctatcaca atctctatct tcatccaaca caacaaaatg a
//
LOCUS KY328021 651 bp cRNA linear VRL 12-DEC-2016
DEFINITION Human respiratory syncytial virus A isolate TH-CU/C5068/2014
attachment glycoprotein gene, partial cds.
ACCESSION KY328021
VERSION KY328021
KEYWORDS .
SOURCE Human respiratory syncytial virus A
ORGANISM Human respiratory syncytial virus A
Viruses; ssRNA viruses; ssRNA negative-strand viruses;
Mononegavirales; Pneumoviridae; Orthopneumovirus.
REFERENCE 1 (bases 1 to 651)
AUTHORS Thongpan,I., Mauleekoonphairoj,J., Vichiwattana,P., Korkong,S.,

Vongpunsawad,S. and Poovorawan,Y.
TITLE Molecular Characterization of Respiratory Syncytial Virus in
Thailand, 2012-2015
JOURNAL Unpublished
REFERENCE 2 (bases 1 to 651)
AUTHORS Thongpan,I., Mauleekoonphairoj,J., Vichiwattana,P., Korkong,S.,
Vongpunsawad,S. and Poovorawan,Y.
TITLE Direct Submission
JOURNAL Submitted (13-DEC-2016) Department of Pediatrics, Center of
Excellence in Clinical Virology, Faculty of Medicine, Chulalongkorn
University, Bangkok 10330, Thailand
COMMENT ##Assembly-Data-START##
Assembly Method :: DNASTAR-Lasergene v. 6
Sequencing Technology :: Sanger dideoxy sequencing
##Assembly-Data-END##
FEATURES Location/Qualifiers
source 1..651
/organism="Human respiratory syncytial virus A"
/mol_type="viral cRNA"
/isolate="TH-CU/C5068/2014"
/host="Homo sapiens"
/db_xref="taxon:208893"
/country="Thailand"
/collection_date="10-Sep-2014"
/note="group: A"
CDS <1..651
/note="G protein"
/codon_start=1
/product="attachment glycoprotein"
/protein_id="APY20362"
/translation="GTTSQSTTILASTTPSAESTPQSTTVKIKNTTTTQILPSKPTTK
QRQNKPQNKPNNDFHFEVFNFVPCSICSNNPTCWAICKRIPNKKPGKKTTTKPTKKPT
LKTTKKDPKPQTTKPKEVLTTKPTGKPTINTTKTNIRTTLLTSNTKGNPEHTSQKETL
HSTTSEGHLSPSQVHTTSGQEETLHSTTSESYLSPSQVYTTSEYLSQSLSSSNTTK"
ORIGIN
1 ggaactacat cacaatccac caccatacta gcttcaacaa caccaagtgc tgagtcaacc
61 ccacaatcca caacagtcaa gatcaaaaac acaacaacaa cccaaatatt acctagcaaa
121 cccaccacaa aacaacgcca aaataaacca caaaacaaac ccaacaatga ttttcacttt
181 gaagtgttca attttgtacc ctgcagcata tgcagcaaca atccaacctg ctgggccatc
241 tgcaagagaa taccaaacaa aaaacctgga aagaaaacca ccaccaagcc cacaaaaaaa
301 ccaaccctca agacaaccaa aaaagatccc aaacctcaaa ccacaaaacc aaaggaagta
361 ctcacaacca agcctacagg aaagccaacc atcaacacca ctaaaacaaa catcagaact
421 acactgctca cctccaacac caaaggaaat ccagaacaca caagtcaaaa ggaaaccctc
481 cactcaacca cctccgaagg ccatctaagc ccatcacaag tccatacaac atccggtcaa
541 gaggaaaccc tccactcaac cacctccgaa agctatctaa gcccatcaca agtctataca
601 acatccgagt acctatcaca atctctatct tcatccaaca caacaaaatg a
//
LOCUS KY328022 651 bp cRNA linear VRL 12-DEC-2016
DEFINITION Human respiratory syncytial virus A isolate TH-CU/C5052/2014
attachment glycoprotein gene, partial cds.
ACCESSION KY328022
VERSION KY328022
KEYWORDS .
SOURCE Human respiratory syncytial virus A

```
  ORGANISM  Human respiratory syncytial virus A
            Viruses; ssRNA viruses; ssRNA negative-strand viruses;
            Mononegavirales; Pneumoviridae; Orthopneumovirus.
REFERENCE   1  (bases 1 to 651)
  AUTHORS   Thongpan,I., Mauleekoonphairoj,J., Vichiwattana,P., Korkong,S.,
            Vongpunsawad,S. and Poovorawan,Y.
  TITLE     Molecular Characterization of Respiratory Syncytial Virus in
            Thailand, 2012-2015
  JOURNAL   Unpublished
REFERENCE   2  (bases 1 to 651)
  AUTHORS   Thongpan,I., Mauleekoonphairoj,J., Vichiwattana,P., Korkong,S.,
            Vongpunsawad,S. and Poovorawan,Y.
  TITLE     Direct Submission
  JOURNAL   Submitted (13-DEC-2016) Department of Pediatrics, Center of
            Excellence in Clinical Virology, Faculty of Medicine, Chulalongkorn
            University, Bangkok 10330, Thailand
COMMENT     ##Assembly-Data-START##
            Assembly Method       :: DNASTAR-Lasergene v. 6
            Sequencing Technology :: Sanger dideoxy sequencing
            ##Assembly-Data-END##
FEATURES             Location/Qualifiers
     source          1..651
                     /organism="Human respiratory syncytial virus A"
                     /mol_type="viral cRNA"
                     /isolate="TH-CU/C5052/2014"
                     /host="Homo sapiens"
                     /db_xref="taxon:208893"
                     /country="Thailand"
                     /collection_date="27-Aug-2014"
                     /note="group: A"
     CDS             <1..651
                     /note="G protein"
                     /codon_start=1
                     /product="attachment glycoprotein"
                     /protein_id="APY20363"
                     /translation="GTTLQSTTILASTTPSAESTPQSTTVKIINTTTTQILPSKPTTK
                     QRQNKPQNKPNNDFHFEVFNFVPCSICSNNPTCWAICKRIPNKKPGKKTTTKPTKKPT
                     LKTTKKDPKPQTTKPKEALTTKPTGKPTINTTKTNIRTTLLTSNTKGNPEHTSQKETL
                     HSTTSEGHPSPSQVHTTSGQEETLHSTTSESYPSPSQVYTTSEYPSQSPSSSNTTK"
ORIGIN
        1 ggaactacat tacaatccac caccatacta gcttcaacaa caccaagtgc tgagtcaacc
       61 ccacaatcca caacagtcaa gatcataaac acaacaacaa cccaaatatt acctagcaaa
      121 cccaccacaa aacaacgcca aaataaacca caaaacaaac ccaacaatga ttttcacttt
      181 gaagtgttca attttgtacc ctgcagcata tgcagcaaca atccaacctg ctgggccatc
      241 tgcaagagaa taccaaacaa aaaacctgga aagaaaacca ccaccaagcc cacaaaaaaa
      301 ccaaccctca agacaaccaa aaaagatccc aaacctcaaa ccacaaaacc aaaggaagca
      361 ctcacaacca agcccacagg aaagccaacc atcaacacca ccaaaacaaa catcagaact
      421 acactgctca cctccaacac caaaggaaat ccagaacaca caagtcaaaa ggaaaccctc
      481 cactcaacca cctccgaagg ccatccaagc ccatcacaag tccatacaac atccggtcaa
      541 gaggaaaccc tccactcaac cacctccgaa agctatccaa gcccatcaca agtctataca
      601 acatccgagt acccatcaca atctccatct tcatccaaca caacaaaatg a
//
LOCUS       KY328023                 651 bp    cRNA    linear   VRL 12-DEC-2016
DEFINITION  Human respiratory syncytial virus A isolate TH-CU/C5158/2014
```

attachment glycoprotein gene, partial cds.
ACCESSION KY328023
VERSION KY328023
KEYWORDS .
SOURCE Human respiratory syncytial virus A
ORGANISM Human respiratory syncytial virus A
Viruses; ssRNA viruses; ssRNA negative-strand viruses;
Mononegavirales; Pneumoviridae; Orthopneumovirus.
REFERENCE 1 (bases 1 to 651)
AUTHORS Thongpan,I., Mauleekoonphairoj,J., Vichiwattana,P., Korkong,S.,
Vongpunsawad,S. and Poovorawan,Y.
TITLE Molecular Characterization of Respiratory Syncytial Virus in
Thailand, 2012-2015
JOURNAL Unpublished
REFERENCE 2 (bases 1 to 651)
AUTHORS Thongpan,I., Mauleekoonphairoj,J., Vichiwattana,P., Korkong,S.,
Vongpunsawad,S. and Poovorawan,Y.
TITLE Direct Submission
JOURNAL Submitted (13-DEC-2016) Department of Pediatrics, Center of
Excellence in Clinical Virology, Faculty of Medicine, Chulalongkorn
University, Bangkok 10330, Thailand
COMMENT ##Assembly-Data-START##
Assembly Method :: DNASTAR-Lasergene v. 6
Sequencing Technology :: Sanger dideoxy sequencing
##Assembly-Data-END##
FEATURES Location/Qualifiers
source 1..651
/organism="Human respiratory syncytial virus A"
/mol_type="viral cRNA"
/isolate="TH-CU/C5158/2014"
/host="Homo sapiens"
/db_xref="taxon:208893"
/country="Thailand"
/collection_date="08-Oct-2014"
/note="group: A"
CDS <1..651
/note="G protein"
/codon_start=1
/product="attachment glycoprotein"
/protein_id="APY20364"
/translation="GTTLQSTTILASTTPSAESTPQSTTVKIINTTTTQILPSKPTTK
QRQNKPQNKPNNDFHFEVFNFVPCSICSNNPTCWAICKRIPNKKPGKKTTTKPTKKPT
LKTTKKDPKPQTTKPKEALTTKPTGKPTINTTKTNIRTTLLTSNTKGNPEHTSQKETL
HSTTSEGHLSPSQVHTTSGQEETLHSTTSESYLSPSQVYTTSEYLSQSLSSSNTTK"
ORIGIN
1 ggaactacat tacaatccac caccatacta gcttcaacaa caccaagtgc tgagtcaacc
61 ccacaatcca caacagtcaa gatcataaac acaacaacaa cccaaatatt acctagcaaa
121 cccaccacaa aacaacgcca aaataaacca caaaacaaac ccaacaatga ttttcacttt
181 gaagtgttca attttgtacc ctgcagcata tgcagcaaca atccaacctg ctgggccatc
241 tgcaagagaa taccaaacaa aaaacctgga aagaaaacca ccaccaagcc cacaaaaaaa
301 ccaaccctca agacaaccaa aaaagatccc aaacctcaaa ccacaaaacc aaaggaagca
361 ctcacaacca agcctacagg aaagccaacc atcaacacca ctaaaacaaa catcagaact
421 acactgctca cctccaacac caaaggaaat ccagaacaca caagtcaaaa ggaaaccctc
481 cactcaacca cctccgaagg ccatctaagc ccatcacaag tccatacaac atccggtcaa

541 gaggaaaccc tccactcaac cacctccgaa agctatctaa gcccatcaca agtctataca
601 acatccgagt acctatcaca atctctatct tcatccaaca caacaaaatg a
//
LOCUS KY328024 651 bp cRNA linear VRL 12-DEC-2016
DEFINITION Human respiratory syncytial virus A isolate TH-CU/C5079/2014
attachment glycoprotein gene, partial cds.
ACCESSION KY328024
VERSION KY328024
KEYWORDS .
SOURCE Human respiratory syncytial virus A
ORGANISM Human respiratory syncytial virus A
Viruses; ssRNA viruses; ssRNA negative-strand viruses;
Mononegavirales; Pneumoviridae; Orthopneumovirus.
REFERENCE 1 (bases 1 to 651)
AUTHORS Thongpan,I., Mauleekoonphairoj,J., Vichiwattana,P., Korkong,S.,
Vongpunsawad,S. and Poovorawan,Y.
TITLE Molecular Characterization of Respiratory Syncytial Virus in
Thailand, 2012-2015
JOURNAL Unpublished
REFERENCE 2 (bases 1 to 651)
AUTHORS Thongpan,I., Mauleekoonphairoj,J., Vichiwattana,P., Korkong,S.,
Vongpunsawad,S. and Poovorawan,Y.
TITLE Direct Submission
JOURNAL Submitted (13-DEC-2016) Department of Pediatrics, Center of
Excellence in Clinical Virology, Faculty of Medicine, Chulalongkorn
University, Bangkok 10330, Thailand
COMMENT ##Assembly-Data-START##
Assembly Method :: DNASTAR-Lasergene v. 6
Sequencing Technology :: Sanger dideoxy sequencing
##Assembly-Data-END##
FEATURES Location/Qualifiers
source 1..651
/organism="Human respiratory syncytial virus A"
/mol_type="viral cRNA"
/isolate="TH-CU/C5079/2014"
/host="Homo sapiens"
/db_xref="taxon:208893"
/country="Thailand"
/collection_date="10-Sep-2014"
/note="group: A"
CDS <1..651
/note="G protein"
/codon_start=1
/product="attachment glycoprotein"
/protein_id="APY20365"
/translation="GTTLQSTTILASTTPSAESTPQSTTVKIINTTTTQILPSKPTTK
QRQNKPQNKPNNDFHFEVFNFVPCSICSNNPTCWAICKRIPNKKPGKKTTTKPTKKPT
LKTTKKDPKPQTTKPKEALTTKPTGKPTINTTKTNIRTTLLTSNTKGNPEHTSQKETL
HSTTSEGHLSPSQVHTTSGQEETLHSTTSESYLSPSQVYTTSEYLSQSLSSSNTTK"
ORIGIN
1 ggaactacat tacaatccac caccatacta gcttcaacaa caccaagtgc tgagtcaacc
61 ccacaatcca caacagtcaa gatcataaac acaacaacaa cccaaatatt acctagcaaa
121 cccaccacaa aacaacgcca aaataaacca caaaacaaac ccaacaatga ttttcacttt
181 gaagtgttca attttgtacc ctgcagcata tgcagcaaca atccaacctg ctgggccatc

```
     241 tgcaagagaa taccaaacaa aaaacctgga aagaaaacca ccaccaagcc cacaaaaaaa
     301 ccaaccctca agacaaccaa aaaagatccc aaacctcaaa ccacaaaacc aaaggaagca
     361 ctcacaacca agcctacagg aaagccaacc atcaacacca ctaaaacaaa catcagaact
     421 acactgctca cctccaacac caaaggaaat ccagaacaca caagtcaaaa ggaaaccctc
     481 cactcaacca cctccgaagg ccatctaagc ccatcacaag tccatacaac atccggtcaa
     541 gaggaaaccc tccactcaac cacctccgaa agctatctaa gcccatcaca agtctataca
     601 acatccgagt acctatcaca atctctatct tcatccaaca caacaaaatg a
//
LOCUS       KY328025                 651 bp    cRNA    linear   VRL 12-DEC-2016
DEFINITION  Human respiratory syncytial virus A isolate TH-CU/B12797/2015
            attachment glycoprotein gene, partial cds.
ACCESSION   KY328025
VERSION     KY328025
KEYWORDS    .
SOURCE      Human respiratory syncytial virus A
  ORGANISM  Human respiratory syncytial virus A
            Viruses; ssRNA viruses; ssRNA negative-strand viruses;
            Mononegavirales; Pneumoviridae; Orthopneumovirus.
REFERENCE   1  (bases 1 to 651)
  AUTHORS   Thongpan,I., Mauleekoonphairoj,J., Vichiwattana,P., Korkong,S.,
            Vongpunsawad,S. and Poovorawan,Y.
  TITLE     Molecular Characterization of Respiratory Syncytial Virus in
            Thailand, 2012-2015
  JOURNAL   Unpublished
REFERENCE   2  (bases 1 to 651)
  AUTHORS   Thongpan,I., Mauleekoonphairoj,J., Vichiwattana,P., Korkong,S.,
            Vongpunsawad,S. and Poovorawan,Y.
  TITLE     Direct Submission
  JOURNAL   Submitted (13-DEC-2016) Department of Pediatrics, Center of
            Excellence in Clinical Virology, Faculty of Medicine, Chulalongkorn
            University, Bangkok 10330, Thailand
COMMENT     ##Assembly-Data-START##
            Assembly Method       :: DNASTAR-Lasergene v. 6
            Sequencing Technology :: Sanger dideoxy sequencing
            ##Assembly-Data-END##
FEATURES             Location/Qualifiers
     source          1..651
                     /organism="Human respiratory syncytial virus A"
                     /mol_type="viral cRNA"
                     /isolate="TH-CU/B12797/2015"
                     /host="Homo sapiens"
                     /db_xref="taxon:208893"
                     /country="Thailand"
                     /collection_date="01-Sep-2015"
                     /note="group: A"
     CDS             <1..651
                     /note="G protein"
                     /codon_start=1
                     /product="attachment glycoprotein"
                     /protein_id="APY20366"
                     /translation="GTTSQSTTILASTTPSAESTPQSTTVKIKNTTTTQILPSKPTTK
                     QRQNKPQNKPNNDFHFEVFNFVPCSICSNNPTCWAICKRIPNKKPGKKTTTKPTKKPT
                     LKTTKKDPNPQTTKPKEVLTTKPTRKPTINTTKTNIRTTLLTSNTKGNPEHTSQEETL
                     HSTTSKGYLSPSQVYTTSGQEETLHSTTSEGYLSPSQVYTTSEYLSQSLSSSNTTK"
```

ORIGIN
1 ggaactacat cacaatccac caccatacta gcttcaacaa caccaagtgc tgagtcaacc
61 ccgcaatcca caacagtcaa gatcaaaaac acaacaacaa cccaaatatt acccagcaaa
121 cccaccacaa aacaacgcca aaataaacca caaaacaaac ccaacaatga ttttcacttt
181 gaagtgttca attttgtacc ctgcagcata tgcagcaaca atccaacctg ctgggccatc
241 tgcaagagaa taccaaacaa aaaacctgga aagaaaacca ccaccaagcc cacaaaaaaa
301 ccaaccctca aaacaaccaa aaaagatccc aaccctcaaa ccacaaaacc aaaggaagta
361 ctcactacca agcctacaag aaagccaacc atcaacacca ctaaaacaaa catcagaact
421 acactgctca cctccaacac caaaggaaat ccagaacaca caagtcaaga ggaaaccctc
481 cactcaacca cctccaaagg ctatctaagc ccatcccaag tctatacaac atccggtcaa
541 gaggaaaccc tccactcaac cacctccgaa ggctatctaa gcccatcaca agtctataca
601 acatccgagt acctatcaca atctctatct tcatccaaca caacaaaatg a
//
LOCUS KY328026 651 bp cRNA linear VRL 12-DEC-2016
DEFINITION Human respiratory syncytial virus A isolate TH-CU/C6247/2015
attachment glycoprotein gene, partial cds.
ACCESSION KY328026
VERSION KY328026
KEYWORDS .
SOURCE Human respiratory syncytial virus A
ORGANISM Human respiratory syncytial virus A
Viruses; ssRNA viruses; ssRNA negative-strand viruses;
Mononegavirales; Pneumoviridae; Orthopneumovirus.
REFERENCE 1 (bases 1 to 651)
AUTHORS Thongpan,I., Mauleekoonphairoj,J., Vichiwattana,P., Korkong,S.,
Vongpunsawad,S. and Poovorawan,Y.
TITLE Molecular Characterization of Respiratory Syncytial Virus in
Thailand, 2012-2015
JOURNAL Unpublished
REFERENCE 2 (bases 1 to 651)
AUTHORS Thongpan,I., Mauleekoonphairoj,J., Vichiwattana,P., Korkong,S.,
Vongpunsawad,S. and Poovorawan,Y.
TITLE Direct Submission
JOURNAL Submitted (13-DEC-2016) Department of Pediatrics, Center of
Excellence in Clinical Virology, Faculty of Medicine, Chulalongkorn
University, Bangkok 10330, Thailand
COMMENT ##Assembly-Data-START##
Assembly Method :: DNASTAR-Lasergene v. 6
Sequencing Technology :: Sanger dideoxy sequencing
##Assembly-Data-END##
FEATURES Location/Qualifiers
source 1..651
/organism="Human respiratory syncytial virus A"
/mol_type="viral cRNA"
/isolate="TH-CU/C6247/2015"
/host="Homo sapiens"
/db_xref="taxon:208893"
/country="Thailand"
/collection_date="19-Nov-2015"
/note="group: A"
CDS <1..651
/note="G protein"
/codon_start=1
/product="attachment glycoprotein"

```
                     /protein_id="APY20367"
                     /translation="GTTSQSTTILASTTPSAESTPQSTTVKIKNTTTTQILPSKPTTK
                     QRQNKPQNKPNNDFHFEVFNFVPCSICSNNPTCWAICKRIPNKKPGKKTTTKPTKKPT
                     LKTTKIDPNPQTTKPKEVLTTKPTRKPTINTTKTNIRTTLLTSNTKGNPEHTSQEETL
                     HSTTSKGYLSPSQVYTTSGQEETLHSTTSEGYLSPSQVYTTSEYLSQSLSSSNTTK"
ORIGIN
        1 ggaactacat cacaatccac caccatacta gcttcaacaa caccaagtgc tgagtcaacc
       61 ccgcaatcca caacagtcaa gatcaaaaac acaacaacaa cccaaatatt acccagcaaa
      121 cccaccacaa aacaacgcca aaataaacca caaaacaaac ccaacaatga ttttcacttt
      181 gaagtgttca attttgtacc ctgcagcata tgcagcaaca atccaacctg ctgggccatc
      241 tgcaagagaa taccaaacaa aaaacctgga aagaaaacca ccaccaagcc cacaaaaaaa
      301 ccaaccctca aaacaaccaa aatagatccc aaccctcaaa ccacaaaacc aaaggaagta
      361 ctcactacca agcctacaag aaagccaacc atcaacacca ctaaaacaaa catcagaact
      421 acactgctca cctccaacac caaaggaaat ccagaacaca caagtcaaga ggaaaccctc
      481 cactcaacca cctccaaagg ctatctaagc ccatcccaag tctatacaac atccggtcaa
      541 gaggaaaccc tccactcaac cacctccgaa ggctatctaa gcccatcaca agtctataca
      601 acatccgagt acctatcaca atctctatct tcatccaaca caacaaaatg a
//
LOCUS       KY328027                 651 bp    cRNA    linear   VRL 12-DEC-2016
DEFINITION  Human respiratory syncytial virus A isolate TH-CU/B12631/2015
            attachment glycoprotein gene, partial cds.
ACCESSION   KY328027
VERSION     KY328027
KEYWORDS    .
SOURCE      Human respiratory syncytial virus A
  ORGANISM  Human respiratory syncytial virus A
            Viruses; ssRNA viruses; ssRNA negative-strand viruses;
            Mononegavirales; Pneumoviridae; Orthopneumovirus.
REFERENCE   1  (bases 1 to 651)
  AUTHORS   Thongpan,I., Mauleekoonphairoj,J., Vichiwattana,P., Korkong,S.,
            Vongpunsawad,S. and Poovorawan,Y.
  TITLE     Molecular Characterization of Respiratory Syncytial Virus in
            Thailand, 2012-2015
  JOURNAL   Unpublished
REFERENCE   2  (bases 1 to 651)
  AUTHORS   Thongpan,I., Mauleekoonphairoj,J., Vichiwattana,P., Korkong,S.,
            Vongpunsawad,S. and Poovorawan,Y.
  TITLE     Direct Submission
  JOURNAL   Submitted (13-DEC-2016) Department of Pediatrics, Center of
            Excellence in Clinical Virology, Faculty of Medicine, Chulalongkorn
            University, Bangkok 10330, Thailand
COMMENT     ##Assembly-Data-START##
            Assembly Method       :: DNASTAR-Lasergene v. 6
            Sequencing Technology :: Sanger dideoxy sequencing
            ##Assembly-Data-END##
FEATURES             Location/Qualifiers
     source          1..651
                     /organism="Human respiratory syncytial virus A"
                     /mol_type="viral cRNA"
                     /isolate="TH-CU/B12631/2015"
                     /host="Homo sapiens"
                     /db_xref="taxon:208893"
                     /country="Thailand"
                     /collection_date="14-Aug-2015"
```

```
                     /note="group: A"
     CDS             <1..651
                     /note="G protein"
                     /codon_start=1
                     /product="attachment glycoprotein"
                     /protein_id="APY20368"
                     /translation="GTTSQSTTILASTTPSAESTPQSTTVKIKNTTTTQILPSKPTTK
                     QRQNKPQNKPNNDFHFEVFNFVPCSICSNNPTCWAICKRIPNKKPGKKTTTKPTKKPT
                     LKTTKKDPNPQTTKPKEVLTTKPTRKPTINTTKTNIRTTLLTSNTKGNPEHTSQEETL
                     HSTTSKGYLSPSQVYTTSGQEETLHSTTSEGYLSPSQVYTTSEYLSQSLSSSNTTK"
ORIGIN
        1 ggaactacat cacaatccac caccatacta gcttcaacaa caccaagtgc tgagtcaacc
       61 ccgcaatcca caacagtcaa gatcaaaaac acaacaacaa cccaaatatt acccagcaaa
      121 cccaccacaa aacaacgcca aaataaacca caaaacaaac ccaacaatga ttttcacttt
      181 gaagtgttca attttgtacc ctgcagcata tgcagcaaca atccaacctg ctgggccatc
      241 tgcaagagaa taccaaacaa aaaacctgga aagaaaacca ccaccaagcc cacaaaaaaa
      301 ccaaccctca aaacaaccaa aaaagatccc aaccctcaaa ccacaaaacc aaaggaagta
      361 ctcactacca agcctacaag aaagccaacc atcaacacca ctaaaacaaa catcagaact
      421 acactgctca cctccaacac caaaggaaat ccagaacaca caagtcaaga ggaaaccctc
      481 cactcaacca cctccaaagg ctatctaagc ccatcccaag tctatacaac atccggtcaa
      541 gaggaaaccc tccactcaac cacctccgaa ggctatctaa gcccatcaca agtctataca
      601 acatccgagt acctatcaca atctctatct tcatccaaca caacaaaatg a
//
LOCUS       KY328028                 651 bp    cRNA    linear   VRL 12-DEC-2016
DEFINITION  Human respiratory syncytial virus A isolate TH-CU/C6221/2015
            attachment glycoprotein gene, partial cds.
ACCESSION   KY328028
VERSION     KY328028
KEYWORDS    .
SOURCE      Human respiratory syncytial virus A
  ORGANISM  Human respiratory syncytial virus A
            Viruses; ssRNA viruses; ssRNA negative-strand viruses;
            Mononegavirales; Pneumoviridae; Orthopneumovirus.
REFERENCE   1  (bases 1 to 651)
  AUTHORS   Thongpan,I., Mauleekoonphairoj,J., Vichiwattana,P., Korkong,S.,
            Vongpunsawad,S. and Poovorawan,Y.
  TITLE     Molecular Characterization of Respiratory Syncytial Virus in
            Thailand, 2012-2015
  JOURNAL   Unpublished
REFERENCE   2  (bases 1 to 651)
  AUTHORS   Thongpan,I., Mauleekoonphairoj,J., Vichiwattana,P., Korkong,S.,
            Vongpunsawad,S. and Poovorawan,Y.
  TITLE     Direct Submission
  JOURNAL   Submitted (13-DEC-2016) Department of Pediatrics, Center of
            Excellence in Clinical Virology, Faculty of Medicine, Chulalongkorn
            University, Bangkok 10330, Thailand
COMMENT     ##Assembly-Data-START##
            Assembly Method       :: DNASTAR-Lasergene v. 6
            Sequencing Technology :: Sanger dideoxy sequencing
            ##Assembly-Data-END##
FEATURES             Location/Qualifiers
     source          1..651
                     /organism="Human respiratory syncytial virus A"
                     /mol_type="viral cRNA"
```

```
                     /isolate="TH-CU/C6221/2015"
                     /host="Homo sapiens"
                     /db_xref="taxon:208893"
                     /country="Thailand"
                     /collection_date="04-Nov-2015"
                     /note="group: A"
     CDS             <1..651
                     /note="G protein"
                     /codon_start=1
                     /product="attachment glycoprotein"
                     /protein_id="APY20369"
                     /translation="GTTSQSTTILASTTPSAESTPQSTTVKIKNTTTTQILPSKPTTK
                     QRQNKPQNKPNNDFHFEVFNFVPCSICSNNPTCWAICKRIPNKKPGKKTTTKPTKKPT
                     LKTTKKDPNPQTTKPKEVLTTKPTRKPTINTTKTNVITTLLTSNTKGNPEHTSQEETL
                     HSTTSKGYLSPSQVYTTSGQEETLHSTTSEGYLSPSQVYTTSEYLSQSLSSSNTTK"
ORIGIN
        1 ggaactacat cacaatccac caccatacta gcttcaacaa caccaagtgc tgagtcaacc
       61 ccgcaatcca caacagtcaa gatcaaaaac acaacaacaa cccaaatatt acccagcaaa
      121 cccaccacaa aacaacgcca aaataaacca caaaacaaac ccaacaatga ttttcacttt
      181 gaagtgttca attttgtacc ctgcagcata tgcagcaaca atccaacctg ctgggccatc
      241 tgcaagagaa taccaaacaa aaaacctgga aagaaaacca ccaccaagcc cacaaaaaaa
      301 ccaaccctca aaacaaccaa aaaagatccc aaccctcaaa ccacaaaacc aaaggaagta
      361 ctcactacca agcctacaag aaagccaacc atcaacacca ctaaaacaaa cgtcataact
      421 acactgctca cctccaacac caaaggaaat ccagaacaca caagtcaaga ggaaaccctc
      481 cactcaacca cctccaaagg ctatctaagc ccatcccaag tctatacaac atccggtcaa
      541 gaggaaaccc tccactcaac cacctccgaa ggctatctaa gcccatcaca agtctataca
      601 acatccgagt acctatcaca atctctatct tcatccaaca caacaaaatg a
//
LOCUS       KY328029                 651 bp    cRNA    linear   VRL 12-DEC-2016
DEFINITION  Human respiratory syncytial virus A isolate TH-CU/B12259/2015
            attachment glycoprotein gene, partial cds.
ACCESSION   KY328029
VERSION     KY328029
KEYWORDS    .
SOURCE      Human respiratory syncytial virus A
  ORGANISM  Human respiratory syncytial virus A
            Viruses; ssRNA viruses; ssRNA negative-strand viruses;
            Mononegavirales; Pneumoviridae; Orthopneumovirus.
REFERENCE   1  (bases 1 to 651)
  AUTHORS   Thongpan,I., Mauleekoonphairoj,J., Vichiwattana,P., Korkong,S.,
            Vongpunsawad,S. and Poovorawan,Y.
  TITLE     Molecular Characterization of Respiratory Syncytial Virus in
            Thailand, 2012-2015
  JOURNAL   Unpublished
REFERENCE   2  (bases 1 to 651)
  AUTHORS   Thongpan,I., Mauleekoonphairoj,J., Vichiwattana,P., Korkong,S.,
            Vongpunsawad,S. and Poovorawan,Y.
  TITLE     Direct Submission
  JOURNAL   Submitted (13-DEC-2016) Department of Pediatrics, Center of
            Excellence in Clinical Virology, Faculty of Medicine, Chulalongkorn
            University, Bangkok 10330, Thailand
COMMENT     ##Assembly-Data-START##
            Assembly Method       :: DNASTAR-Lasergene v. 6
            Sequencing Technology :: Sanger dideoxy sequencing
```

```
        ##Assembly-Data-END##
FEATURES             Location/Qualifiers
     source          1..651
                     /organism="Human respiratory syncytial virus A"
                     /mol_type="viral cRNA"
                     /isolate="TH-CU/B12259/2015"
                     /host="Homo sapiens"
                     /db_xref="taxon:208893"
                     /country="Thailand"
                     /collection_date="15-Jul-2015"
                     /note="group: A"
     CDS             <1..651
                     /note="G protein"
                     /codon_start=1
                     /product="attachment glycoprotein"
                     /protein_id="APY20370"
                     /translation="GTTSQSTTILASTTPSAESTPQSTTVKIKNTTTTQILPSKPTTK
                     QRQNKPQNKPNNDFHFEVFNFVPCSICSNNPTCWAICKRIPNKKPGKKTTTKPTKKPT
                     LKTTKKDPNPQTTKPKEVLTTKPTRKPTINTTKTNIRTTLLTSNTKGNPEHTSQEETL
                     HSTTSKGYPSPSQVHTTSGQEETLHSTTSEGYLSPSQVYTTSEYLSQSLSSSNTTK"
ORIGIN
        1 ggaactacat cacaatccac caccatacta gcttcaacaa caccaagtgc tgagtcaacc
       61 ccgcaatcca caacagtcaa gatcaaaaac acaacaacaa cccaaatatt acccagcaaa
      121 cccaccacaa aacaacgcca aaataaacca caaaacaaac ccaacaatga ttttcacttt
      181 gaagtgttca attttgtacc ctgcagcata tgcagcaaca atccaacctg ctgggccatc
      241 tgcaaaagaa taccaaacaa aaaacctgga aagaaaacca ccaccaagcc cacaaaaaaa
      301 ccaaccctca aaacaaccaa aaaagatccc aaccctcaaa ccacaaaacc aaaggaagta
      361 ctcactacca agcctacaag aaagccaacc atcaacacca ctaaaacaaa catcagaact
      421 acactgctca cctccaacac caaaggaaat ccagaacaca caagtcaaga ggaaaccctc
      481 cactcaacca cctccaaagg ctatccaagc ccatcccaag tccatacaac atccggtcaa
      541 gaggaaaccc tccactcaac cacctccgaa ggctatctaa gcccatcaca agtctacaca
      601 acatccgagt acctatcaca atctctatct tcatccaaca caacaaaatg a
//
LOCUS       KY328030                 651 bp    cRNA    linear   VRL 12-DEC-2016
DEFINITION  Human respiratory syncytial virus A isolate TH-CU/C6233/2015
            attachment glycoprotein gene, partial cds.
ACCESSION   KY328030
VERSION     KY328030
KEYWORDS    .
SOURCE      Human respiratory syncytial virus A
  ORGANISM  Human respiratory syncytial virus A
            Viruses; ssRNA viruses; ssRNA negative-strand viruses;
            Mononegavirales; Pneumoviridae; Orthopneumovirus.
REFERENCE   1  (bases 1 to 651)
  AUTHORS   Thongpan,I., Mauleekoonphairoj,J., Vichiwattana,P., Korkong,S.,
            Vongpunsawad,S. and Poovorawan,Y.
  TITLE     Molecular Characterization of Respiratory Syncytial Virus in
            Thailand, 2012-2015
  JOURNAL   Unpublished
REFERENCE   2  (bases 1 to 651)
  AUTHORS   Thongpan,I., Mauleekoonphairoj,J., Vichiwattana,P., Korkong,S.,
            Vongpunsawad,S. and Poovorawan,Y.
  TITLE     Direct Submission
  JOURNAL   Submitted (13-DEC-2016) Department of Pediatrics, Center of
```

```
            Excellence in Clinical Virology, Faculty of Medicine, Chulalongkorn
            University, Bangkok 10330, Thailand
COMMENT     ##Assembly-Data-START##
            Assembly Method       :: DNASTAR-Lasergene v. 6
            Sequencing Technology :: Sanger dideoxy sequencing
            ##Assembly-Data-END##
FEATURES             Location/Qualifiers
     source          1..651
                     /organism="Human respiratory syncytial virus A"
                     /mol_type="viral cRNA"
                     /isolate="TH-CU/C6233/2015"
                     /host="Homo sapiens"
                     /db_xref="taxon:208893"
                     /country="Thailand"
                     /collection_date="04-Nov-2015"
                     /note="group: A"
     CDS             <1..651
                     /note="G protein"
                     /codon_start=1
                     /product="attachment glycoprotein"
                     /protein_id="APY20371"
                     /translation="GTTSQSTTILASTTPSAESTPQSTTVKIKNTTTTQILPSKPTTK
                     QRQNKPQNKPNNDFHFEVFNFVPCSICSNNPTCWAICKRIPNKKPGKKTTTKPTKKPT
                     LKTTKKDPNPQTTKPKEVLTTKPTGKPTINTTKTNIRTTLLTSNTKGNSEYTSQEETL
                     HSTTYKGYLSPSQVYTTSGQEETLHSTTSEGYLSSSQVYTISEYLSQSPSSSNTTK"
ORIGIN
        1 ggaactacat cacaatccac caccatacta gcttcaacaa caccaagtgc tgagtcaacc
       61 ccacaatcca caacagtcaa gatcaaaaac acaacaacaa cccaaatatt acctagcaaa
      121 cccaccacaa aacaacgcca aaataaacca caaaacaaac ccaacaatga ttttcacttt
      181 gaagtgttca attttgtacc ctgcagcata tgcagcaaca atccaacctg ctgggccatc
      241 tgcaagagaa tacccaacaa aaaacctgga aagaaaacca ccaccaagcc cacaaaaaaa
      301 ccaaccctca aaacaaccaa aaaagatccc aatcctcaaa ccacaaaacc aaaggaagta
      361 ctcactacca agcctacagg aaagccaacc atcaacacca ctaaaacaaa catcagaact
      421 acactgctca cctccaacac caaaggaaat tcagaataca caagtcaaga ggaaaccctc
      481 cactcaacca cctacaaagg ctatctaagc ccatcccaag tctatacaac atccggtcaa
      541 gaggaaaccc tccactcaac cacctccgaa ggctatctaa gctcatcaca agtctataca
      601 atatccgagt acctatcaca atctccatct tcatccaaca caacaaaatg a
//
LOCUS       KY328031                 651 bp    cRNA    linear   VRL 12-DEC-2016
DEFINITION  Human respiratory syncytial virus A isolate TH-CU/C5087/2014
            attachment glycoprotein gene, partial cds.
ACCESSION   KY328031
VERSION     KY328031
KEYWORDS    .
SOURCE      Human respiratory syncytial virus A
  ORGANISM  Human respiratory syncytial virus A
            Viruses; ssRNA viruses; ssRNA negative-strand viruses;
            Mononegavirales; Pneumoviridae; Orthopneumovirus.
REFERENCE   1  (bases 1 to 651)
  AUTHORS   Thongpan,I., Mauleekoonphairoj,J., Vichiwattana,P., Korkong,S.,
            Vongpunsawad,S. and Poovorawan,Y.
  TITLE     Molecular Characterization of Respiratory Syncytial Virus in
            Thailand, 2012-2015
  JOURNAL   Unpublished
```

REFERENCE   2 (bases 1 to 651)
  AUTHORS   Thongpan,I., Mauleekoonphairoj,J., Vichiwattana,P., Korkong,S.,
            Vongpunsawad,S. and Poovorawan,Y.
  TITLE   Direct Submission
  JOURNAL   Submitted (13-DEC-2016) Department of Pediatrics, Center of
            Excellence in Clinical Virology, Faculty of Medicine, Chulalongkorn
            University, Bangkok 10330, Thailand
COMMENT   ##Assembly-Data-START##
            Assembly Method   :: DNASTAR-Lasergene v. 6
            Sequencing Technology :: Sanger dideoxy sequencing
            ##Assembly-Data-END##
FEATURES   Location/Qualifiers
     source   1..651
                     /organism="Human respiratory syncytial virus A"
                     /mol_type="viral cRNA"
                     /isolate="TH-CU/C5087/2014"
                     /host="Homo sapiens"
                     /db_xref="taxon:208893"
                     /country="Thailand"
                     /collection_date="10-Sep-2014"
                     /note="group: A"
     CDS   <1..651
                     /note="G protein"
                     /codon_start=1
                     /product="attachment glycoprotein"
                     /protein_id="APY20372"
                     /translation="GTTSQSTTILASTTPSAESTPQSTTVKIKNTTTTQILPSKPTTK
                     QRQNKPQNKPNNDFHFEVFNFVPCSICSNNPTCWAICKRIPNKKPGKKTTTKPTKKPT
                     LKTTKKDPNPQTTKPKEVLTTKPTGKPTINTTKTNIRTTLLTSNTKGNPEYTSQEETL
                     HSTTYKGYPSPSQVHTTSGQEETLHSTTSEGYPSPSQVYTTSEYLSQSLSSSNTTK"
ORIGIN
        1 ggaactacat cacaatccac caccatacta gcttcaacaa caccaagtgc tgagtcaacc
       61 ccacaatcca caacagtcaa gatcaaaaac acaacaacaa cccaaatatt acctagcaaa
      121 cccaccacaa aacaacgcca aaataaacca caaaacaaac ccaacaatga ttttcacttt
      181 gaagtgttca attttgtacc ctgcagcata tgcagcaaca atccaacctg ctgggccatc
      241 tgcaagagaa taccaaacaa aaaacctgga aagaaaacca ccaccaagcc cacaaaaaaa
      301 ccaaccctca aaacaaccaa aaaagatccc aaccctcaaa ccacaaaacc aaaggaagta
      361 ctcactacca agcctacagg aaagccaacc atcaacacca ctaaaacaaa catcagaact
      421 acactgctca cctccaacac caaaggaaat ccagaataca caagtcaaga ggaaaccctc
      481 cactcaacca cctacaaagg ctatccaagc ccatcccaag tccatacaac atccggtcaa
      541 gaggaaaccc tccactcaac cacctccgaa ggctatccaa gcccatcaca agtctataca
      601 acatccgagt acctatcaca atctctatct tcatccaaca caacaaaatg a
//
LOCUS   KY328032   651 bp   cRNA   linear   VRL 12-DEC-2016
DEFINITION Human respiratory syncytial virus A isolate TH-CU/C5091/2014
            attachment glycoprotein gene, partial cds.
ACCESSION   KY328032
VERSION   KY328032
KEYWORDS   .
SOURCE   Human respiratory syncytial virus A
  ORGANISM Human respiratory syncytial virus A
            Viruses; ssRNA viruses; ssRNA negative-strand viruses;
            Mononegavirales; Pneumoviridae; Orthopneumovirus.
REFERENCE   1 (bases 1 to 651)

```
  AUTHORS   Thongpan,I., Mauleekoonphairoj,J., Vichiwattana,P., Korkong,S.,
            Vongpunsawad,S. and Poovorawan,Y.
  TITLE     Molecular Characterization of Respiratory Syncytial Virus in
            Thailand, 2012-2015
  JOURNAL   Unpublished
REFERENCE   2  (bases 1 to 651)
  AUTHORS   Thongpan,I., Mauleekoonphairoj,J., Vichiwattana,P., Korkong,S.,
            Vongpunsawad,S. and Poovorawan,Y.
  TITLE     Direct Submission
  JOURNAL   Submitted (13-DEC-2016) Department of Pediatrics, Center of
            Excellence in Clinical Virology, Faculty of Medicine, Chulalongkorn
            University, Bangkok 10330, Thailand
COMMENT     ##Assembly-Data-START##
            Assembly Method       :: DNASTAR-Lasergene v. 6
            Sequencing Technology :: Sanger dideoxy sequencing
            ##Assembly-Data-END##
FEATURES             Location/Qualifiers
     source          1..651
                     /organism="Human respiratory syncytial virus A"
                     /mol_type="viral cRNA"
                     /isolate="TH-CU/C5091/2014"
                     /host="Homo sapiens"
                     /db_xref="taxon:208893"
                     /country="Thailand"
                     /collection_date="10-Sep-2014"
                     /note="group: A"
     CDS             <1..651
                     /note="G protein"
                     /codon_start=1
                     /product="attachment glycoprotein"
                     /protein_id="APY20373"
                     /translation="GTTSQSTTILASTTPSAESTPQSTTVKIKNTTTTQILPSKPTTK
                     QRQNKPQNKPNNDFHFEVFNFVPCSICSNNPTCWAICKRIPNKKPGKKTTTKPTKKPT
                     LKTTKKDPNPQTTKPKEVLTTKPTGKPTINTTKTNIRTTLLTSNTKGNPEYTSQEETL
                     HSTTYKGYPSPSQVHTTSGQEETLHSTTSEGYPSPSQVYTTSEYLSQSLSSSNTTK"
ORIGIN
        1 ggaactacat cacaatccac caccatacta gcttcaacaa caccaagtgc tgagtcaacc
       61 ccacaatcca caacagtcaa gatcaaaaac acaacaacaa cccaaatatt acctagcaaa
      121 cccaccacaa aacaacgcca aaataaacca caaaacaaac ccaacaatga ttttcacttt
      181 gaagtgttca attttgtacc ctgcagcata tgcagcaaca atccaacctg ctgggccatc
      241 tgcaagagaa taccaaacaa aaaacctgga aagaaaacca ccaccaagcc cacaaaaaaa
      301 ccaaccctca aaacaaccaa aaaagatccc aaccctcaaa ccacaaaacc aaaggaagta
      361 ctcactacca agcctacagg aaagccaacc atcaacacca ctaaaacaaa catcagaact
      421 acactgctca cctccaacac caaaggaaat ccagaataca caagtcaaga ggaaaccctc
      481 cactcaacca cctacaaagg ctatccaagc ccatcccaag tccatacaac atccggtcaa
      541 gaggaaaccc tccactcaac cacctccgaa ggctatccaa gcccatcaca agtctataca
      601 acatccgagt acctatcaca atctctatct tcatccaaca caacaaaatg a
//
LOCUS       KY328033                 651 bp    cRNA    linear   VRL 12-DEC-2016
DEFINITION  Human respiratory syncytial virus A isolate ON1/TH-CU/B12563/2015
            attachment glycoprotein gene, partial cds.
ACCESSION   KY328033
VERSION     KY328033
KEYWORDS    .
```

SOURCE Human respiratory syncytial virus A
ORGANISM Human respiratory syncytial virus A
Viruses; ssRNA viruses; ssRNA negative-strand viruses;
Mononegavirales; Pneumoviridae; Orthopneumovirus.
REFERENCE 1 (bases 1 to 651)
AUTHORS Thongpan,I., Mauleekoonphairoj,J., Vichiwattana,P., Korkong,S.,
Vongpunsawad,S. and Poovorawan,Y.
TITLE Molecular Characterization of Respiratory Syncytial Virus in
Thailand, 2012-2015
JOURNAL Unpublished
REFERENCE 2 (bases 1 to 651)
AUTHORS Thongpan,I., Mauleekoonphairoj,J., Vichiwattana,P., Korkong,S.,
Vongpunsawad,S. and Poovorawan,Y.
TITLE Direct Submission
JOURNAL Submitted (13-DEC-2016) Department of Pediatrics, Center of
Excellence in Clinical Virology, Faculty of Medicine, Chulalongkorn
University, Bangkok 10330, Thailand
COMMENT ##Assembly-Data-START##
Assembly Method :: DNASTAR-Lasergene v. 6
Sequencing Technology :: Sanger dideoxy sequencing
##Assembly-Data-END##
FEATURES Location/Qualifiers
source 1..651
/organism="Human respiratory syncytial virus A"
/mol_type="viral cRNA"
/isolate="ON1/TH-CU/B12563/2015"
/host="Homo sapiens"
/db_xref="taxon:208893"
/country="Thailand"
/collection_date="16-Aug-2015"
/note="group: A"
CDS <1..651
/note="G protein"
/codon_start=1
/product="attachment glycoprotein"
/protein_id="APY20374"
/translation="GTTSQSTTILASTTPSAESTPQSTTVKIKNTTTTQILPSKPTTK
QRQNKPQNKPNNDFHFEVFNFVPCSICSNNPTCWAICKRIPNKKPGKKTTTKPTKKPT
LKTTKKDPKPQTTKPKEVLTTKPTGKPTINTTKTNIRTTLLTSNTKGNPEHTSQEETL
HSTTSEGYLSPSQVYTTSGQEETLHSTTSEGYLSPSQVYTTSEYLSQSLSSSNTTK"
ORIGIN
1 ggaactacat cacaatccac caccatacta gcttcaacaa caccaagtgc tgagtcaacc
61 ccacaatcca caacagtcaa gatcaaaaac acaacaacaa ctcaaatatt acctagcaaa
121 cccaccacaa aacaacgcca aaataaacca caaaacaaac ccaacaatga ttttcacttt
181 gaagtgttca attttgtacc ctgcagcata tgcagcaaca atccaacctg ctgggccatc
241 tgcaagagaa taccaaacaa aaaacctgga aagaaaacca ccaccaagcc cacaaaaaaa
301 ccaaccctca aaacaaccaa aaaagatccc aaacctcaaa ccacaaaacc aaaggaagta
361 ctcactacca agcctacagg aaagccaacc atcaacacca ctaaaacaaa catcagaact
421 acactgctca cctccaacac caaaggaaat ccagaacaca caagtcaaga ggaaaccctc
481 cactcaacca cctccgaagg ctatctaagc ccatcacaag tctatacaac atccggtcaa
541 gaggaaaccc tccactcaac cacctccgaa ggctatctaa gcccatcaca agtctataca
601 acatccgagt acctatcaca atctctatct tcatccaaca caacaaaatg a
//
LOCUS KY328034 651 bp cRNA linear VRL 12-DEC-2016

DEFINITION Human respiratory syncytial virus A isolate TH-CU/B12963/2015
attachment glycoprotein gene, partial cds.
ACCESSION   KY328034
VERSION   KY328034
KEYWORDS   .
SOURCE   Human respiratory syncytial virus A
  ORGANISM Human respiratory syncytial virus A
Viruses; ssRNA viruses; ssRNA negative-strand viruses;
Mononegavirales; Pneumoviridae; Orthopneumovirus.
REFERENCE   1 (bases 1 to 651)
  AUTHORS   Thongpan,I., Mauleekoonphairoj,J., Vichiwattana,P., Korkong,S.,
Vongpunsawad,S. and Poovorawan,Y.
  TITLE   Molecular Characterization of Respiratory Syncytial Virus in
Thailand, 2012-2015
  JOURNAL   Unpublished
REFERENCE   2 (bases 1 to 651)
  AUTHORS   Thongpan,I., Mauleekoonphairoj,J., Vichiwattana,P., Korkong,S.,
Vongpunsawad,S. and Poovorawan,Y.
  TITLE   Direct Submission
  JOURNAL   Submitted (13-DEC-2016) Department of Pediatrics, Center of
Excellence in Clinical Virology, Faculty of Medicine, Chulalongkorn
University, Bangkok 10330, Thailand
COMMENT   ##Assembly-Data-START##
Assembly Method   :: DNASTAR-Lasergene v. 6
Sequencing Technology :: Sanger dideoxy sequencing
##Assembly-Data-END##
FEATURES   Location/Qualifiers
     source   1..651
/organism="Human respiratory syncytial virus A"
/mol_type="viral cRNA"
/isolate="TH-CU/B12963/2015"
/host="Homo sapiens"
/db_xref="taxon:208893"
/country="Thailand"
/collection_date="19-Sep-2015"
/note="group: A"
     CDS   <1..651
/note="G protein"
/codon_start=1
/product="attachment glycoprotein"
/protein_id="APY20375"
/translation="GTTSQSTTILASTTPSAESTPQSTTIKIKNTTTTQILPSKPTTK
QRQNKPQNKPNNDFHFEVFNFVPCSICSNNPTCWAICKRIPNKKPGKKTTTKPTKKQT
LKTTKKDPKPQTTKPKEVLTTKPTGKPTINTTKTNVRTTLITSNTKGNPEHTSQEETL
HSTTSEGYPSPSQVYTTSGQEETLHSTTSEGYPSPSQAHTTSEYPSQSLSSSNTAK"
ORIGIN
1 ggaactacat cacaatccac caccatacta gcttcaacaa caccaagtgc tgagtcaacc
61 ccacaatcca caacaatcaa gatcaaaaac acaacaacaa cccaaatatt acctagcaaa
121 cccaccacaa aacaacgcca aaataaacca caaaacaaac ccaacaatga ttttcacttt
181 gaagtgttca attttgtacc ctgcagcata tgcagcaaca atccaacctg ctgggccatc
241 tgcaagagaa taccaaacaa gaaacctgga aagaaaacca ccaccaagcc cacaaagaaa
301 caaaccctca agacaaccaa aaaagatccc aaacctcaaa ccacaaaacc aaaggaagta
361 ctcactacca agcctacagg aaagccaacc atcaacacca ctaaaacaaa cgtcagaact
421 acactgatca cctccaacac caaaggaaat ccagaacaca caagtcaaga ggaaaccctc

```
     481 cactcaacca cctccgaagg ctatccaagc ccatcacaag tctacacaac atccggtcaa
     541 gaggaaaccc tccactcaac cacctccgaa ggttatccaa gcccatcaca agcccataca
     601 acatccgagt acccatcaca atctctatct tcatccaaca cagcaaaatg a
//
LOCUS       KY328035                 651 bp    cRNA    linear   VRL 12-DEC-2016
DEFINITION  Human respiratory syncytial virus A isolate TH-CU/B12477/2015
            attachment glycoprotein gene, partial cds.
ACCESSION   KY328035
VERSION     KY328035
KEYWORDS    .
SOURCE      Human respiratory syncytial virus A
  ORGANISM  Human respiratory syncytial virus A
            Viruses; ssRNA viruses; ssRNA negative-strand viruses;
            Mononegavirales; Pneumoviridae; Orthopneumovirus.
REFERENCE   1  (bases 1 to 651)
  AUTHORS   Thongpan,I., Mauleekoonphairoj,J., Vichiwattana,P., Korkong,S.,
            Vongpunsawad,S. and Poovorawan,Y.
  TITLE     Molecular Characterization of Respiratory Syncytial Virus in
            Thailand, 2012-2015
  JOURNAL   Unpublished
REFERENCE   2  (bases 1 to 651)
  AUTHORS   Thongpan,I., Mauleekoonphairoj,J., Vichiwattana,P., Korkong,S.,
            Vongpunsawad,S. and Poovorawan,Y.
  TITLE     Direct Submission
  JOURNAL   Submitted (13-DEC-2016) Department of Pediatrics, Center of
            Excellence in Clinical Virology, Faculty of Medicine, Chulalongkorn
            University, Bangkok 10330, Thailand
COMMENT     ##Assembly-Data-START##
            Assembly Method       :: DNASTAR-Lasergene v. 6
            Sequencing Technology :: Sanger dideoxy sequencing
            ##Assembly-Data-END##
FEATURES             Location/Qualifiers
     source          1..651
                     /organism="Human respiratory syncytial virus A"
                     /mol_type="viral cRNA"
                     /isolate="TH-CU/B12477/2015"
                     /host="Homo sapiens"
                     /db_xref="taxon:208893"
                     /country="Thailand"
                     /collection_date="04-Aug-2015"
                     /note="group: A"
     CDS             <1..651
                     /note="G protein"
                     /codon_start=1
                     /product="attachment glycoprotein"
                     /protein_id="APY20376"
                     /translation="GTTSQSTTILASTTPSAESTPQSTTVKIKNTTTTQILPSKPTTK
                     QRQNKPQNKPNNDFHFEVFNFVPCSICSNNPTCWAICKRIPNKKPGKKTTTKPTKKQT
                     LKTTKKDPKPQTTKPKEVLTTKPTGKPTINTTKTNIRTTLITSNTKGNPEHTSQEETL
                     HSTTSEGYPSPSQVYTTSGQEETLHSTTSEGYPSPSQAHTTSEYPSQSLSSSNTAK"
ORIGIN
        1 ggaactacat cacaatccac caccatacta gcttcaacaa caccaagtgc tgagtcaacc
       61 ccacaatcca caacagtcaa gatcaaaaac acaacaacaa cccaaatatt acctagcaaa
      121 cccaccacaa aacaacgcca aaataaacca caaaacaaac ccaacaatga ttttcacttt
```

```
      181 gaagtgttca attttgtacc ctgcagcata tgcagcaaca atccaacctg ctgggccatc
      241 tgcaagagaa taccaaacaa gaaacctgga aagaaaacca ccaccaagcc cacaaagaaa
      301 caaaccctca agacaaccaa aaaagatccc aaacctcaaa ccacaaaacc aaaggaagta
      361 ctcactacca agcctacagg aaagccaacc atcaacacca ctaaaacaaa catcagaact
      421 acactgatca cctccaacac caaaggaaat ccagaacaca caagtcaaga ggaaaccctc
      481 cactcaacca cctccgaagg ctatccaagc ccatcacaag tctacacaac atccggtcaa
      541 gaggaaaccc tccactcaac cacctccgaa ggttatccaa gcccatcaca agcccataca
      601 acatccgagt acccatcaca atctctatct tcatccaaca cagcaaaatg a
//
LOCUS       KY328036               651 bp    cRNA    linear   VRL 12-DEC-2016
DEFINITION  Human respiratory syncytial virus A isolate TH-CU/B12696/2015
            attachment glycoprotein gene, partial cds.
ACCESSION   KY328036
VERSION     KY328036
KEYWORDS    .
SOURCE      Human respiratory syncytial virus A
  ORGANISM  Human respiratory syncytial virus A
            Viruses; ssRNA viruses; ssRNA negative-strand viruses;
            Mononegavirales; Pneumoviridae; Orthopneumovirus.
REFERENCE   1  (bases 1 to 651)
  AUTHORS   Thongpan,I., Mauleekoonphairoj,J., Vichiwattana,P., Korkong,S.,
            Vongpunsawad,S. and Poovorawan,Y.
  TITLE     Molecular Characterization of Respiratory Syncytial Virus in
            Thailand, 2012-2015
  JOURNAL   Unpublished
REFERENCE   2  (bases 1 to 651)
  AUTHORS   Thongpan,I., Mauleekoonphairoj,J., Vichiwattana,P., Korkong,S.,
            Vongpunsawad,S. and Poovorawan,Y.
  TITLE     Direct Submission
  JOURNAL   Submitted (13-DEC-2016) Department of Pediatrics, Center of
            Excellence in Clinical Virology, Faculty of Medicine, Chulalongkorn
            University, Bangkok 10330, Thailand
COMMENT     ##Assembly-Data-START##
            Assembly Method       :: DNASTAR-Lasergene v. 6
            Sequencing Technology :: Sanger dideoxy sequencing
            ##Assembly-Data-END##
FEATURES             Location/Qualifiers
     source          1..651
                     /organism="Human respiratory syncytial virus A"
                     /mol_type="viral cRNA"
                     /isolate="TH-CU/B12696/2015"
                     /host="Homo sapiens"
                     /db_xref="taxon:208893"
                     /country="Thailand"
                     /collection_date="22-Aug-2015"
                     /note="group: A"
     CDS             <1..651
                     /note="G protein"
                     /codon_start=1
                     /product="attachment glycoprotein"
                     /protein_id="APY20377"
                     /translation="GTTSQSTTILASTTPSAESTPQSTTVKIKNTTTTQILPSKPTTK
                     QRQNKPQNKPNNDFHFEVFNFVPCSICSNNPTCWAICKRIPNKKPGKKTTTKPTKKQT
                     LKTTKKDPKPQTTKPKEVLTTKPTGKPTINTTKTNIRTTLLTSNTKGNPEHTSQEETL
```

HSTTSEGYPSPSQVYTTSGQEETLHSTTSEGYPSPSQAHTTSEYPSQSLSSSNTAK"
ORIGIN
```
        1 ggaactacat cacaatccac caccatacta gcttcaacaa caccaagtgc tgagtcaacc
       61 ccacaatcca caacagtcaa gatcaaaaac acaacaacaa cccaaatatt acctagcaaa
      121 cccaccacaa aacaacgcca aaataaacca caaaacaaac ccaacaatga ttttcacttt
      181 gaagtgttca attttgtacc ctgcagcata tgcagcaaca atccaacctg ctgggccatc
      241 tgcaagagaa taccaaacaa gaaacctgga aagaaaacca ccaccaagcc cacaaagaaa
      301 caaaccctca agacaaccaa aaaagatccc aaacctcaaa ccacaaaacc aaaggaagta
      361 ctcactacca agcctacagg aaagccaacc atcaacacca ctaaaacaaa catcagaact
      421 acactgctca cctccaacac caaaggaaat ccagaacaca caagtcaaga ggaaaccctc
      481 cactcaacca cctccgaagg ctatccaagc ccatcacaag tctacacaac atccggtcaa
      541 gaggaaaccc tccactcaac cacctccgaa ggttatccaa gcccatcaca agcccataca
      601 acatccgagt acccatcaca atctctatct tcatccaaca cagcaaaatg a
```
//
LOCUS   KY328037   651 bp   cRNA   linear   VRL 12-DEC-2016
DEFINITION Human respiratory syncytial virus A isolate TH-CU/B10980/2014
            attachment glycoprotein gene, partial cds.
ACCESSION   KY328037
VERSION   KY328037
KEYWORDS   .
SOURCE   Human respiratory syncytial virus A
  ORGANISM Human respiratory syncytial virus A
            Viruses; ssRNA viruses; ssRNA negative-strand viruses;
            Mononegavirales; Pneumoviridae; Orthopneumovirus.
REFERENCE   1 (bases 1 to 651)
  AUTHORS   Thongpan,I., Mauleekoonphairoj,J., Vichiwattana,P., Korkong,S.,
            Vongpunsawad,S. and Poovorawan,Y.
  TITLE   Molecular Characterization of Respiratory Syncytial Virus in
            Thailand, 2012-2015
  JOURNAL   Unpublished
REFERENCE   2 (bases 1 to 651)
  AUTHORS   Thongpan,I., Mauleekoonphairoj,J., Vichiwattana,P., Korkong,S.,
            Vongpunsawad,S. and Poovorawan,Y.
  TITLE   Direct Submission
  JOURNAL   Submitted (13-DEC-2016) Department of Pediatrics, Center of
            Excellence in Clinical Virology, Faculty of Medicine, Chulalongkorn
            University, Bangkok 10330, Thailand
COMMENT   ##Assembly-Data-START##
            Assembly Method   :: DNASTAR-Lasergene v. 6
            Sequencing Technology :: Sanger dideoxy sequencing
            ##Assembly-Data-END##
FEATURES   Location/Qualifiers
     source   1..651
                     /organism="Human respiratory syncytial virus A"
                     /mol_type="viral cRNA"
                     /isolate="TH-CU/B10980/2014"
                     /host="Homo sapiens"
                     /db_xref="taxon:208893"
                     /country="Thailand"
                     /collection_date="11-Sep-2014"
                     /note="group: A"
     CDS   <1..651
                     /note="G protein"
                     /codon_start=1

```
                     /product="attachment glycoprotein"
                     /protein_id="APY20378"
                     /translation="GTTSQSTTILASTTLSAESTPQSTTVKIKNTTTTQILPSKPTTK
                     QRQNKPQNKPNNDFHFEVFNFVPCSICSNNPTCWAICKRIPNKKPGKKTTTKPTKKPT
                     LKTTKKDPKPQTTKPKKVLTTKPTGKPTINTTKTNIRTTLLTSNTKGNPEHTSQEETL
                     HSTTSEGYPSPSQVYTTSGQEETLHSTTSEGYPSPSQVHTTSEYLSQSLSSSNTTK"
ORIGIN
        1 ggaactacat cacaatccac caccatacta gcttcaacaa cattaagtgc tgagtcaacc
       61 ccacaatcca caacagtcaa gatcaaaaac acaacaacaa cccaaatatt acctagcaaa
      121 cccaccacaa aacaacgcca aaataaacca caaaacaaac ccaacaatga ttttcacttt
      181 gaagtgttca attttgtacc ctgcagcata tgcagcaaca atccaacctg ctgggccatc
      241 tgcaagagaa taccaaacaa aaaacctgga aagaaaacca ccaccaagcc cacaaaaaaa
      301 ccaaccctca agacaaccaa aaaagatccc aaacctcaaa ccacaaaacc aaagaaagta
      361 ctcactacca agcctacagg aaagccaacc atcaacacca ctaaaacaaa catcagaact
      421 acactgctca cctccaacac caaaggaaat ccagaacaca caagtcaaga ggaaaccctc
      481 cactcaacca cctccgaagg ctatccaagc ccatcacaag tctatacaac atccggtcaa
      541 gaggaaaccc tccactcaac cacctccgaa ggctatccaa gcccatcaca agtccataca
      601 acatccgagt acctatcaca atctctatct tcatccaaca caacaaaatg a
//
LOCUS       KY328038                 651 bp    cRNA    linear   VRL 12-DEC-2016
DEFINITION  Human respiratory syncytial virus A isolate TH-CU/CB122/2013
            attachment glycoprotein gene, partial cds.
ACCESSION   KY328038
VERSION     KY328038
KEYWORDS    .
SOURCE      Human respiratory syncytial virus A
  ORGANISM  Human respiratory syncytial virus A
            Viruses; ssRNA viruses; ssRNA negative-strand viruses;
            Mononegavirales; Pneumoviridae; Orthopneumovirus.
REFERENCE   1  (bases 1 to 651)
  AUTHORS   Thongpan,I., Mauleekoonphairoj,J., Vichiwattana,P., Korkong,S.,
            Vongpunsawad,S. and Poovorawan,Y.
  TITLE     Molecular Characterization of Respiratory Syncytial Virus in
            Thailand, 2012-2015
  JOURNAL   Unpublished
REFERENCE   2  (bases 1 to 651)
  AUTHORS   Thongpan,I., Mauleekoonphairoj,J., Vichiwattana,P., Korkong,S.,
            Vongpunsawad,S. and Poovorawan,Y.
  TITLE     Direct Submission
  JOURNAL   Submitted (13-DEC-2016) Department of Pediatrics, Center of
            Excellence in Clinical Virology, Faculty of Medicine, Chulalongkorn
            University, Bangkok 10330, Thailand
COMMENT     ##Assembly-Data-START##
            Assembly Method       :: DNASTAR-Lasergene v. 6
            Sequencing Technology :: Sanger dideoxy sequencing
            ##Assembly-Data-END##
FEATURES             Location/Qualifiers
     source          1..651
                     /organism="Human respiratory syncytial virus A"
                     /mol_type="viral cRNA"
                     /isolate="TH-CU/CB122/2013"
                     /host="Homo sapiens"
                     /db_xref="taxon:208893"
                     /country="Thailand"
```

/collection_date="01-Nov-2013"
/note="group: A"
CDS <1..651
/note="G protein"
/codon_start=1
/product="attachment glycoprotein"
/protein_id="APY20379"
/translation="GTTSQSTTILASTTSSAESTPQSTTVKIKNTTTTQILPSKPTTK
QRQNKPQNKPNNDFHFEVFNFVPCSICSNNPTCWAICKRIPNKKPGKKTTTKPTKKPT
LKTTKKDPKPQTTKPKKVLTTKPTGKPTINTTKTNIRTTLLTSNTKGNPEHTSQEETL
HSTTSEGYPSPSQVYTTSGQEETLHSTTSEGYPSPSQVHTTSEYLSQSLSSSNTTK"
ORIGIN
1 ggaactacat cacaatccac caccatacta gcttcaacaa catcaagtgc tgagtcaacc
61 ccacaatcca caacagtcaa gatcaaaaac acaacaacaa cccaaatatt acctagcaaa
121 cccaccacaa aacaacgcca aaataaacca caaaacaaac ccaacaatga ttttcacttt
181 gaagtgttca attttgtacc ctgcagcata tgcagcaaca atccaacctg ctgggccatc
241 tgcaagagaa taccaaacaa aaaacctgga aagaaaacca ccaccaagcc cacaaaaaaa
301 ccaaccctca agacaaccaa aaaagatccc aaacctcaaa ccacaaaacc aaagaaagta
361 ctcactacca agcctacagg aaagccaacc atcaacacca ctaaaacaaa catcagaact
421 acactgctca cctccaacac caaaggaaat ccagaacaca caagtcaaga ggaaaccctc
481 cactcaacca cctccgaagg ctatccaagc ccatcacaag tctatacaac atccggtcaa
541 gaggaaaccc tccactcaac cacctccgaa ggctatccaa gcccatcaca agtccataca
601 acatccgagt acctatcaca atctctatct tcatccaaca caacaaaatg a
//
LOCUS KY328039 651 bp cRNA linear VRL 12-DEC-2016
DEFINITION Human respiratory syncytial virus A isolate TH-CU/CB119/2013
attachment glycoprotein gene, partial cds.
ACCESSION KY328039
VERSION KY328039
KEYWORDS .
SOURCE Human respiratory syncytial virus A
ORGANISM Human respiratory syncytial virus A
Viruses; ssRNA viruses; ssRNA negative-strand viruses;
Mononegavirales; Pneumoviridae; Orthopneumovirus.
REFERENCE 1 (bases 1 to 651)
AUTHORS Thongpan,I., Mauleekoonphairoj,J., Vichiwattana,P., Korkong,S.,
Vongpunsawad,S. and Poovorawan,Y.
TITLE Molecular Characterization of Respiratory Syncytial Virus in
Thailand, 2012-2015
JOURNAL Unpublished
REFERENCE 2 (bases 1 to 651)
AUTHORS Thongpan,I., Mauleekoonphairoj,J., Vichiwattana,P., Korkong,S.,
Vongpunsawad,S. and Poovorawan,Y.
TITLE Direct Submission
JOURNAL Submitted (13-DEC-2016) Department of Pediatrics, Center of
Excellence in Clinical Virology, Faculty of Medicine, Chulalongkorn
University, Bangkok 10330, Thailand
COMMENT ##Assembly-Data-START##
Assembly Method :: DNASTAR-Lasergene v. 6
Sequencing Technology :: Sanger dideoxy sequencing
##Assembly-Data-END##
FEATURES Location/Qualifiers
source 1..651
/organism="Human respiratory syncytial virus A"

```
                     /mol_type="viral cRNA"
                     /isolate="TH-CU/CB119/2013"
                     /host="Homo sapiens"
                     /db_xref="taxon:208893"
                     /country="Thailand"
                     /collection_date="01-Nov-2013"
                     /note="group: A"
     CDS             <1..651
                     /note="G protein"
                     /codon_start=1
                     /product="attachment glycoprotein"
                     /protein_id="APY20380"
                     /translation="GTTSQSTTILASTTSSAESTPQSTTVKIKNTTTTQILPSKPTTK
                     QRQNKPQNKPNNDFHFEVFNFVPCSICSNNPTCWAICKRIPNKKPGKKTTTKPTKKPT
                     LKTTKKDPKPQTTKPKKVLTTKPTGKPTINTTKTNIRTTLLTSNTKGNPEHTSQEETL
                     HSTTSEGYPSPSQVYTTSGQEETLHSTTSEGYPSPSQVHTTSEYLSQSLSSSNTTK"
ORIGIN
        1 ggaactacat cacaatccac caccatacta gcttcaacaa catcaagtgc tgagtcaacc
       61 ccacaatcca caacagtcaa gatcaaaaac acaacaacaa cccaaatatt acctagcaaa
      121 cccaccacaa aacaacgcca aaataaacca caaaacaaac ccaacaatga ttttcacttt
      181 gaagtgttca attttgtacc ctgcagcata tgcagcaaca atccaacctg ctgggccatc
      241 tgcaagagaa taccaaacaa aaaacctgga aagaaaacca ccaccaagcc cacaaaaaaa
      301 ccaaccctca agacaaccaa aaaagatccc aaacctcaaa ccacaaaacc aaagaaagta
      361 ctcactacca agcctacagg aaagccaacc atcaacacca ctaaaacaaa catcagaact
      421 acactgctca cctccaacac caaaggaaat ccagaacaca caagtcaaga ggaaaccctc
      481 cactcaacca cctccgaagg ctatccaagc ccatcacaag tctatacaac atccggtcaa
      541 gaggaaaccc tccactcaac cacctccgaa ggctatccaa gcccatcaca agtccataca
      601 acatccgagt acctatcaca atctctatct tcatccaaca caacaaaatg a
//
LOCUS       KY328040                 651 bp    cRNA    linear   VRL 12-DEC-2016
DEFINITION  Human respiratory syncytial virus A isolate TH-CU/CB124/2013
            attachment glycoprotein gene, partial cds.
ACCESSION   KY328040
VERSION     KY328040
KEYWORDS    .
SOURCE      Human respiratory syncytial virus A
  ORGANISM  Human respiratory syncytial virus A
            Viruses; ssRNA viruses; ssRNA negative-strand viruses;
            Mononegavirales; Pneumoviridae; Orthopneumovirus.
REFERENCE   1  (bases 1 to 651)
  AUTHORS   Thongpan,I., Mauleekoonphairoj,J., Vichiwattana,P., Korkong,S.,
            Vongpunsawad,S. and Poovorawan,Y.
  TITLE     Molecular Characterization of Respiratory Syncytial Virus in
            Thailand, 2012-2015
  JOURNAL   Unpublished
REFERENCE   2  (bases 1 to 651)
  AUTHORS   Thongpan,I., Mauleekoonphairoj,J., Vichiwattana,P., Korkong,S.,
            Vongpunsawad,S. and Poovorawan,Y.
  TITLE     Direct Submission
  JOURNAL   Submitted (13-DEC-2016) Department of Pediatrics, Center of
            Excellence in Clinical Virology, Faculty of Medicine, Chulalongkorn
            University, Bangkok 10330, Thailand
COMMENT     ##Assembly-Data-START##
            Assembly Method       :: DNASTAR-Lasergene v. 6
```

Sequencing Technology :: Sanger dideoxy sequencing
##Assembly-Data-END##
FEATURES Location/Qualifiers
source 1..651
/organism="Human respiratory syncytial virus A"
/mol_type="viral cRNA"
/isolate="TH-CU/CB124/2013"
/host="Homo sapiens"
/db_xref="taxon:208893"
/country="Thailand"
/collection_date="01-Nov-2013"
/note="group: A"
CDS <1..651
/note="G protein"
/codon_start=1
/product="attachment glycoprotein"
/protein_id="APY20381"
/translation="GTTSQSTTILASTTSSAESTPQSTTVKIKNTTTTQILPSKPTTK
QRQNKPQNKPNNDFHFEVFNFVPCSICSNNPTCWAICKRIPNKKPGKKTTTKPTKKPT
LKTTKKDPKPQTTKPKKVLTTKPTGKPTINTTKTNIRTTLLTSNTKGNPEHTSQEETL
HSTTSEGYPSPSQVYTTSGQEETLHSTTSEGYPSPSQVHTTSEYLSQSLSSSNTTK"
ORIGIN
1 ggaactacat cacaatccac caccatacta gcttcaacaa catcaagtgc tgagtcaacc
61 ccacaatcca caacagtcaa gatcaaaaac acaacaacaa cccaaatatt acctagcaaa
121 cccaccacaa aacaacgcca aaataaacca caaaacaaac ccaacaatga ttttcacttt
181 gaagtgttca attttgtacc ctgcagcata tgcagcaaca atccaacctg ctgggccatc
241 tgcaagagaa taccaaacaa aaaacctgga aagaaaacca ccaccaagcc cacaaaaaaa
301 ccaaccctca agacaaccaa aaaagatccc aaacctcaaa ccacaaaacc aaagaaagta
361 ctcactacca agcctacagg aaagccaacc atcaacacca ctaaaacaaa catcagaact
421 acactgctca cctccaacac caaaggaaat ccagaacaca caagtcaaga ggaaaccctc
481 cactcaacca cctccgaagg ctatccaagc ccatcacaag tctatacaac atccggtcaa
541 gaggaaaccc tccactcaac cacctccgaa ggctatccaa gcccatcaca agtccataca
601 acatccgagt acctatcaca atctctatct tcatccaaca caacaaaatg a
//
LOCUS KY328041 651 bp cRNA linear VRL 12-DEC-2016
DEFINITION Human respiratory syncytial virus A isolate TH-CU/CB135/2013
attachment glycoprotein gene, partial cds.
ACCESSION KY328041
VERSION KY328041
KEYWORDS .
SOURCE Human respiratory syncytial virus A
ORGANISM Human respiratory syncytial virus A
Viruses; ssRNA viruses; ssRNA negative-strand viruses;
Mononegavirales; Pneumoviridae; Orthopneumovirus.
REFERENCE 1 (bases 1 to 651)
AUTHORS Thongpan,I., Mauleekoonphairoj,J., Vichiwattana,P., Korkong,S.,
Vongpunsawad,S. and Poovorawan,Y.
TITLE Molecular Characterization of Respiratory Syncytial Virus in
Thailand, 2012-2015
JOURNAL Unpublished
REFERENCE 2 (bases 1 to 651)
AUTHORS Thongpan,I., Mauleekoonphairoj,J., Vichiwattana,P., Korkong,S.,
Vongpunsawad,S. and Poovorawan,Y.
TITLE Direct Submission

```
  JOURNAL   Submitted (13-DEC-2016) Department of Pediatrics, Center of
            Excellence in Clinical Virology, Faculty of Medicine, Chulalongkorn
            University, Bangkok 10330, Thailand
COMMENT     ##Assembly-Data-START##
            Assembly Method       :: DNASTAR-Lasergene v. 6
            Sequencing Technology :: Sanger dideoxy sequencing
            ##Assembly-Data-END##
FEATURES             Location/Qualifiers
     source          1..651
                     /organism="Human respiratory syncytial virus A"
                     /mol_type="viral cRNA"
                     /isolate="TH-CU/CB135/2013"
                     /host="Homo sapiens"
                     /db_xref="taxon:208893"
                     /country="Thailand"
                     /collection_date="01-Nov-2013"
                     /note="group: A"
     CDS             <1..651
                     /note="G protein"
                     /codon_start=1
                     /product="attachment glycoprotein"
                     /protein_id="APY20382"
                     /translation="GTTSQSTTILASTTSSAESTPQSTTVKIKNTTTTQILPSKPTTK
                     QRQNKPQNKPNNDFHFEVFNFVPCSICSNNPTCWAICKRIPNKKPGKKTTTKPTKKPT
                     LKTTKKDPKPQTTKPKKVLTTKPTGKPTINTTKTNIRTTLLTSNTKGNPEHTSQEETL
                     HSTTSEGYPSPSQVYTTSGQEETLHSTTSEGYPSPSQVHTTSEYLSQSLSSSNTTK"
ORIGIN      
        1 ggaactacat cacaatccac caccatacta gcttcaacaa catcaagtgc tgagtcaacc
       61 ccacaatcca caacagtcaa gatcaaaaac acaacaacaa cccaaatatt acctagcaaa
      121 cccaccacaa aacaacgcca aaataaacca caaaacaaac ccaacaatga ttttcacttt
      181 gaagtgttca attttgtacc ctgcagcata tgcagcaaca atccaacctg ctgggccatc
      241 tgcaagagaa taccaaacaa aaaacctgga aagaaaacca ccaccaagcc cacaaaaaaa
      301 ccaaccctca agacaaccaa aaaagatccc aaacctcaaa ccacaaaacc aaagaaagta
      361 ctcactacca agcctacagg aaagccaacc atcaacacca ctaaaacaaa catcagaact
      421 acactgctca cctccaacac caaaggaaat ccagaacaca caagtcaaga ggaaaccctc
      481 cactcaacca cctccgaagg ctatccaagc ccatcacaag tctatacaac atccggtcaa
      541 gaggaaaccc tccactcaac cacctccgaa ggctatccaa gcccatcaca agtccataca
      601 acatccgagt acctatcaca atctctatct tcatccaaca caacaaaatg a
//
LOCUS       KY328042                 651 bp    cRNA    linear   VRL 12-DEC-2016
DEFINITION  Human respiratory syncytial virus A isolate TH-CU/CB164/2014
            attachment glycoprotein gene, partial cds.
ACCESSION   KY328042
VERSION     KY328042
KEYWORDS    .
SOURCE      Human respiratory syncytial virus A
  ORGANISM  Human respiratory syncytial virus A
            Viruses; ssRNA viruses; ssRNA negative-strand viruses;
            Mononegavirales; Pneumoviridae; Orthopneumovirus.
REFERENCE   1  (bases 1 to 651)
  AUTHORS   Thongpan,I., Mauleekoonphairoj,J., Vichiwattana,P., Korkong,S.,
            Vongpunsawad,S. and Poovorawan,Y.
  TITLE     Molecular Characterization of Respiratory Syncytial Virus in
            Thailand, 2012-2015
```

```
  JOURNAL   Unpublished
REFERENCE   2  (bases 1 to 651)
  AUTHORS   Thongpan,I., Mauleekoonphairoj,J., Vichiwattana,P., Korkong,S.,
            Vongpunsawad,S. and Poovorawan,Y.
  TITLE     Direct Submission
  JOURNAL   Submitted (13-DEC-2016) Department of Pediatrics, Center of
            Excellence in Clinical Virology, Faculty of Medicine, Chulalongkorn
            University, Bangkok 10330, Thailand
COMMENT     ##Assembly-Data-START##
            Assembly Method       :: DNASTAR-Lasergene v. 6
            Sequencing Technology :: Sanger dideoxy sequencing
            ##Assembly-Data-END##
FEATURES             Location/Qualifiers
     source          1..651
                     /organism="Human respiratory syncytial virus A"
                     /mol_type="viral cRNA"
                     /isolate="TH-CU/CB164/2014"
                     /host="Homo sapiens"
                     /db_xref="taxon:208893"
                     /country="Thailand"
                     /collection_date="01-Jan-2014"
                     /note="group: A"
     CDS             <1..651
                     /note="G protein"
                     /codon_start=1
                     /product="attachment glycoprotein"
                     /protein_id="APY20383"
                     /translation="GTTSQSTTILASTTSSAESTPQSTTVKIKNTTTTQILPSKPTTK
                     QRQNKPQNKPNNDFHFEVFNFVPCSICSNNPTCWAICKRIPNKKPGKKTTTKPTKKPT
                     LKTTKKDPKPQTTKPKKVLTTKPTGKPTINTTKTNIRTTLLTSNTKGNPEHTSQEETL
                     HSTTSEGYPSPSQVYTTSGQEETLHSTTSEGYPSPSQVHTTSEYLSQSLSSSNTTK"
ORIGIN
        1 ggaactacat cacaatccac caccatacta gcttcaacaa catcaagtgc tgagtcaacc
       61 ccacaatcca caacagtcaa gatcaaaaac acaacaacaa cccaaatatt acctagcaaa
      121 cccaccacaa aacaacgcca aaataaacca caaaacaaac ccaacaatga ttttcacttt
      181 gaagtgttca attttgtacc ctgcagcata tgcagcaaca atccaacctg ctgggccatc
      241 tgcaagagaa taccaaacaa aaaacctgga aagaaaacca ccaccaagcc cacaaaaaaa
      301 ccaaccctca agacaaccaa aaaagatccc aaacctcaaa ccacaaaacc aaagaaagta
      361 ctcactacca agcctacagg aaagccaacc atcaacacca ctaaaacaaa catcagaact
      421 acactgctca cctccaacac caaaggaaat ccagaacaca caagtcaaga ggaaaccctc
      481 cactcaacca cctccgaagg ctatccaagc ccatcacaag tctatacaac atccggtcaa
      541 gaggaaaccc tccactcaac cacctccgaa ggctatccaa gcccatcaca agtccataca
      601 acatccgagt acctatcaca atctctatct tcatccaaca caacaaaatg a
//
LOCUS       KY328043                 651 bp    cRNA    linear   VRL 12-DEC-2016
DEFINITION  Human respiratory syncytial virus A isolate TH-CU/CB49/2013
            attachment glycoprotein gene, partial cds.
ACCESSION   KY328043
VERSION     KY328043
KEYWORDS    .
SOURCE      Human respiratory syncytial virus A
  ORGANISM  Human respiratory syncytial virus A
            Viruses; ssRNA viruses; ssRNA negative-strand viruses;
            Mononegavirales; Pneumoviridae; Orthopneumovirus.
```

REFERENCE 1 (bases 1 to 651)
AUTHORS Thongpan,I., Mauleekoonphairoj,J., Vichiwattana,P., Korkong,S.,
Vongpunsawad,S. and Poovorawan,Y.
TITLE Molecular Characterization of Respiratory Syncytial Virus in
Thailand, 2012-2015
JOURNAL Unpublished
REFERENCE 2 (bases 1 to 651)
AUTHORS Thongpan,I., Mauleekoonphairoj,J., Vichiwattana,P., Korkong,S.,
Vongpunsawad,S. and Poovorawan,Y.
TITLE Direct Submission
JOURNAL Submitted (13-DEC-2016) Department of Pediatrics, Center of
Excellence in Clinical Virology, Faculty of Medicine, Chulalongkorn
University, Bangkok 10330, Thailand
COMMENT ##Assembly-Data-START##
Assembly Method :: DNASTAR-Lasergene v. 6
Sequencing Technology :: Sanger dideoxy sequencing
##Assembly-Data-END##
FEATURES Location/Qualifiers
source 1..651
/organism="Human respiratory syncytial virus A"
/mol_type="viral cRNA"
/isolate="TH-CU/CB49/2013"
/host="Homo sapiens"
/db_xref="taxon:208893"
/country="Thailand"
/collection_date="01-Jul-2013"
/note="group: A"
CDS <1..651
/note="G protein"
/codon_start=1
/product="attachment glycoprotein"
/protein_id="APY20384"
/translation="GTTSQSTTILASTTSSAESTPQSTTVKIKNTTTTQILPSKPTTK
QRQNKPQNKPNNDFHFEVFNFVPCSICSNNPTCWAICKRIPNKKPGKKTTTKPTKKPT
LKTTKKDPKPQTTKPKKVLTTKPTGKPTINTTKTNIRTTLLTSNTKGNPEHTSQEETL
HSTTSEGYPSPSQVYTTSGQEETLHSTTSEGYPSPSQVHTTSEYLSQSLSSSNTTK"
ORIGIN
1 ggaactacat cacaatccac caccatacta gcttcaacaa catcaagtgc tgagtcaacc
61 ccacaatcca caacagtcaa gatcaaaaac acaacaacaa cccaaatatt acctagcaaa
121 cccaccacaa aacaacgcca aaataaacca caaaacaaac ccaacaatga ttttcacttt
181 gaagtgttca attttgtacc ctgcagcata tgcagcaaca atccaacctg ctgggccatc
241 tgcaagagaa taccaaacaa aaaacctgga aagaaaacca ctaccaagcc cacaaaaaaa
301 ccaaccctca agacaaccaa aaaagatccc aaacctcaaa ccacaaaacc aaagaaagta
361 ctcactacca agcctacagg aaagccaacc atcaacacca ctaaaacaaa catcagaact
421 acactgctca cctccaacac caaaggaaat ccagaacaca caagtcaaga ggaaaccctc
481 cactcaacca cctccgaagg ctatccaagc ccatcacaag tctatacaac atccggtcaa
541 gaggaaaccc tccactcaac cacctccgaa ggctatccaa gcccatcaca agtccataca
601 acatccgagt acctatcaca atctctatct tcatccaaca caacaaaatg a
//
LOCUS KY328044 651 bp cRNA linear VRL 12-DEC-2016
DEFINITION Human respiratory syncytial virus A isolate TH-CU/B12405/2015
attachment glycoprotein gene, partial cds.
ACCESSION KY328044
VERSION KY328044

```
KEYWORDS    .
SOURCE      Human respiratory syncytial virus A
  ORGANISM  Human respiratory syncytial virus A
            Viruses; ssRNA viruses; ssRNA negative-strand viruses;
            Mononegavirales; Pneumoviridae; Orthopneumovirus.
REFERENCE   1  (bases 1 to 651)
  AUTHORS   Thongpan,I., Mauleekoonphairoj,J., Vichiwattana,P., Korkong,S.,
            Vongpunsawad,S. and Poovorawan,Y.
  TITLE     Molecular Characterization of Respiratory Syncytial Virus in
            Thailand, 2012-2015
  JOURNAL   Unpublished
REFERENCE   2  (bases 1 to 651)
  AUTHORS   Thongpan,I., Mauleekoonphairoj,J., Vichiwattana,P., Korkong,S.,
            Vongpunsawad,S. and Poovorawan,Y.
  TITLE     Direct Submission
  JOURNAL   Submitted (13-DEC-2016) Department of Pediatrics, Center of
            Excellence in Clinical Virology, Faculty of Medicine, Chulalongkorn
            University, Bangkok 10330, Thailand
COMMENT     ##Assembly-Data-START##
            Assembly Method       :: DNASTAR-Lasergene v. 6
            Sequencing Technology :: Sanger dideoxy sequencing
            ##Assembly-Data-END##
FEATURES             Location/Qualifiers
     source          1..651
                     /organism="Human respiratory syncytial virus A"
                     /mol_type="viral cRNA"
                     /isolate="TH-CU/B12405/2015"
                     /host="Homo sapiens"
                     /db_xref="taxon:208893"
                     /country="Thailand"
                     /collection_date="30-Jul-2015"
                     /note="group: A"
     CDS             <1..651
                     /note="G protein"
                     /codon_start=1
                     /product="attachment glycoprotein"
                     /protein_id="APY20385"
                     /translation="GTTSQSTTILASTTPSAESTPQSTTVKIKNTTTTQILPSKPTTK
                     QRQNKPQNKPNNDFHFEVFNFVPCSICSNNPTCWAICKRIPNKKPGKKTTTKPTKKPT
                     LKTTKKDPKPQTTKPKEVLTTKPTGKPTINTTKTNIRTTLLTSNTKGNPEHTSQEETL
                     HSTTSEGYLSPSQVYTTSGQEETLHSTTSEGYLSPSQVYTTSEYLSQSLSSSNTTK"
ORIGIN
        1 ggaactacat cacaatccac caccatacta gcttcaacaa caccaagtgc tgagtcaacc
       61 ccacaatcca caacagtcaa gatcaaaaac acaacaacaa cccaaatatt acctagcaaa
      121 cccaccacaa aacaacgcca aaataaacca caaaacaaac ccaacaatga ttttcacttt
      181 gaagtgttca attttgtacc ctgcagcata tgcagcaaca atccaacctg ctgggccatc
      241 tgcaagagaa taccaaacaa aaaacctgga aagaaaacca ccaccaagcc cacaaaaaaa
      301 ccaaccctca agacaaccaa aaaagatccc aaacctcaaa ccacaaaacc aaaggaagta
      361 ctcactacca agcctacagg aaagccaacc atcaacacca ccaaaacaaa catcagaact
      421 acactgctca cctccaacac caaaggaaat ccagaacaca caagtcaaga ggaaaccctc
      481 cactcaacca cctccgaagg ctatctaagc ccatcccaag tctatacaac atccggtcaa
      541 gaggaaaccc tccactcaac cacctccgaa ggctatctaa gcccatcaca agtctataca
      601 acatccgagt acctatcaca atctctatct tcatccaaca caacaaaatg a
//
```

```
LOCUS       KY328045                 651 bp    cRNA    linear   VRL 12-DEC-2016
DEFINITION  Human respiratory syncytial virus A isolate TH-CU/B12546/2015
            attachment glycoprotein gene, partial cds.
ACCESSION   KY328045
VERSION     KY328045
KEYWORDS    .
SOURCE      Human respiratory syncytial virus A
  ORGANISM  Human respiratory syncytial virus A
            Viruses; ssRNA viruses; ssRNA negative-strand viruses;
            Mononegavirales; Pneumoviridae; Orthopneumovirus.
REFERENCE   1  (bases 1 to 651)
  AUTHORS   Thongpan,I., Mauleekoonphairoj,J., Vichiwattana,P., Korkong,S.,
            Vongpunsawad,S. and Poovorawan,Y.
  TITLE     Molecular Characterization of Respiratory Syncytial Virus in
            Thailand, 2012-2015
  JOURNAL   Unpublished
REFERENCE   2  (bases 1 to 651)
  AUTHORS   Thongpan,I., Mauleekoonphairoj,J., Vichiwattana,P., Korkong,S.,
            Vongpunsawad,S. and Poovorawan,Y.
  TITLE     Direct Submission
  JOURNAL   Submitted (13-DEC-2016) Department of Pediatrics, Center of
            Excellence in Clinical Virology, Faculty of Medicine, Chulalongkorn
            University, Bangkok 10330, Thailand
COMMENT     ##Assembly-Data-START##
            Assembly Method       :: DNASTAR-Lasergene v. 6
            Sequencing Technology :: Sanger dideoxy sequencing
            ##Assembly-Data-END##
FEATURES             Location/Qualifiers
     source          1..651
                     /organism="Human respiratory syncytial virus A"
                     /mol_type="viral cRNA"
                     /isolate="TH-CU/B12546/2015"
                     /host="Homo sapiens"
                     /db_xref="taxon:208893"
                     /country="Thailand"
                     /collection_date="11-Aug-2015"
                     /note="group: A"
     CDS             <1..651
                     /note="G protein"
                     /codon_start=1
                     /product="attachment glycoprotein"
                     /protein_id="APY20386"
                     /translation="GTTSQSTTILASTTPSAESTPQSTTVKIKNTTTTQILPSKPTTK
                     QRQNKPQNKPNNDFHFEVFNFVPCSICSNNPTCWAICKRIPNKKPGKKTTTKPTKKPT
                     LKTTKKDPKPQTTKPKEVLTTKPTGKPTINTTKTNIRTTLLTSNTKGNPEHTSQEETL
                     HSTTSEGYLSPSQVYTTSGQEETLHSTTSEGYLSPSQVYTTSEYLSQSLSSSNTTK"
ORIGIN
        1 ggaactacat cacaatccac caccatacta gcttcaacaa caccaagtgc tgagtcaacc
       61 ccacaatcca caacagtcaa gatcaaaaac acaacaacaa cccaaatatt acctagcaaa
      121 cccaccacaa aacaacgcca aaataaacca caaaacaaac ccaacaatga ttttcacttt
      181 gaagtgttca attttgtacc ctgcagcata tgcagcaaca atccaacctg ctgggccatc
      241 tgcaagagaa taccaaacaa aaaacctgga aagaaaacca ccaccaagcc cacaaaaaaa
      301 ccaaccctca agacaaccaa aaaagatccc aaacctcaaa ccacaaaacc aaaggaagta
      361 ctcactacca agcctacagg aaagccaacc atcaacacca ccaaaacaaa catcagaact
```

421 acactgctca cctccaacac caaaggaaat ccagaacaca caagtcaaga ggaaaccctc
481 cactcaacca cctccgaagg ctatctaagc ccatcccaag tctatacaac atccggtcaa
541 gaggaaaccc tccactcaac cacctccgaa ggctatctaa gcccatcaca agtctataca
601 acatccgagt acctatcaca atctctatct tcatccaaca caacaaaatg a
//
LOCUS KY328046 651 bp cRNA linear VRL 12-DEC-2016
DEFINITION Human respiratory syncytial virus A isolate TH-CU/C6214/2015
attachment glycoprotein gene, partial cds.
ACCESSION KY328046
VERSION KY328046
KEYWORDS .
SOURCE Human respiratory syncytial virus A
ORGANISM Human respiratory syncytial virus A
Viruses; ssRNA viruses; ssRNA negative-strand viruses;
Mononegavirales; Pneumoviridae; Orthopneumovirus.
REFERENCE 1 (bases 1 to 651)
AUTHORS Thongpan,I., Mauleekoonphairoj,J., Vichiwattana,P., Korkong,S.,
Vongpunsawad,S. and Poovorawan,Y.
TITLE Molecular Characterization of Respiratory Syncytial Virus in
Thailand, 2012-2015
JOURNAL Unpublished
REFERENCE 2 (bases 1 to 651)
AUTHORS Thongpan,I., Mauleekoonphairoj,J., Vichiwattana,P., Korkong,S.,
Vongpunsawad,S. and Poovorawan,Y.
TITLE Direct Submission
JOURNAL Submitted (13-DEC-2016) Department of Pediatrics, Center of
Excellence in Clinical Virology, Faculty of Medicine, Chulalongkorn
University, Bangkok 10330, Thailand
COMMENT ##Assembly-Data-START##
Assembly Method :: DNASTAR-Lasergene v. 6
Sequencing Technology :: Sanger dideoxy sequencing
##Assembly-Data-END##
FEATURES Location/Qualifiers
source 1..651
/organism="Human respiratory syncytial virus A"
/mol_type="viral cRNA"
/isolate="TH-CU/C6214/2015"
/host="Homo sapiens"
/db_xref="taxon:208893"
/country="Thailand"
/collection_date="04-Nov-2015"
/note="group: A"
CDS <1..651
/note="G protein"
/codon_start=1
/product="attachment glycoprotein"
/protein_id="APY20387"
/translation="GTTSQSTTILASTTPSAESTPQSTTVKIKNTTTTQILPSKPTTK
QRQNKPQNKPNNDFHFEVFNFVPCSICSNNPTCWAICKRIPNKKPGKKTTTKPTKKPT
LKTTKKDPKPQTTKPKEVLTTKPTGKPTINTTKTNIRTTLLTSNTKGNPEHTSQEETL
HSTTSEGYLSPSQVYTTSGQEETLHSTTSEGYLSPSQVYTTSEYLSQSLSSSNTTK"
ORIGIN
1 ggaactacat cacaatccac caccatacta gcttcaacaa caccaagtgc tgagtcaacc
61 ccacaatcca caacagtcaa gatcaaaaac acaacaacaa cccaaatatt acctagcaaa

```
     121 cccaccacaa aacaacgcca aaataaacca caaaacaaac ccaacaatga ttttcacttt
     181 gaagtgttca attttgtacc ctgcagcata tgcagcaaca atccaacctg ctgggccatc
     241 tgcaagagaa taccaaacaa aaaacctgga aagaaaacca ccaccaagcc cacaaaaaaa
     301 ccaaccctca agacaaccaa aaaagatccc aaacctcaaa ccacaaaacc aaaggaagta
     361 ctcactacca agcctacagg aaagccaacc atcaacacca ccaaaacaaa catcagaacc
     421 acactgctca cctccaacac caaaggaaat ccagaacaca caagtcaaga ggaaaccctc
     481 cactcaacca cctccgaagg ctatctaagc ccatcccaag tctatacaac atccggtcaa
     541 gaggaaaccc tccactcaac cacctccgaa ggctatctaa gcccatcaca agtctataca
     601 acatccgagt acctatcaca atctctatct tcatccaaca caacaaaatg a
//
LOCUS       KY328047                 651 bp    cRNA    linear   VRL 12-DEC-2016
DEFINITION  Human respiratory syncytial virus A isolate TH-CU/B12568/2015
            attachment glycoprotein gene, partial cds.
ACCESSION   KY328047
VERSION     KY328047
KEYWORDS    .
SOURCE      Human respiratory syncytial virus A
  ORGANISM  Human respiratory syncytial virus A
            Viruses; ssRNA viruses; ssRNA negative-strand viruses;
            Mononegavirales; Pneumoviridae; Orthopneumovirus.
REFERENCE   1  (bases 1 to 651)
  AUTHORS   Thongpan,I., Mauleekoonphairoj,J., Vichiwattana,P., Korkong,S.,
            Vongpunsawad,S. and Poovorawan,Y.
  TITLE     Molecular Characterization of Respiratory Syncytial Virus in
            Thailand, 2012-2015
  JOURNAL   Unpublished
REFERENCE   2  (bases 1 to 651)
  AUTHORS   Thongpan,I., Mauleekoonphairoj,J., Vichiwattana,P., Korkong,S.,
            Vongpunsawad,S. and Poovorawan,Y.
  TITLE     Direct Submission
  JOURNAL   Submitted (13-DEC-2016) Department of Pediatrics, Center of
            Excellence in Clinical Virology, Faculty of Medicine, Chulalongkorn
            University, Bangkok 10330, Thailand
COMMENT     ##Assembly-Data-START##
            Assembly Method       :: DNASTAR-Lasergene v. 6
            Sequencing Technology :: Sanger dideoxy sequencing
            ##Assembly-Data-END##
FEATURES             Location/Qualifiers
     source          1..651
                     /organism="Human respiratory syncytial virus A"
                     /mol_type="viral cRNA"
                     /isolate="TH-CU/B12568/2015"
                     /host="Homo sapiens"
                     /db_xref="taxon:208893"
                     /country="Thailand"
                     /collection_date="16-Aug-2015"
                     /note="group: A"
     CDS             <1..651
                     /note="G protein"
                     /codon_start=1
                     /product="attachment glycoprotein"
                     /protein_id="APY20388"
                     /translation="GTTSQSTTILASTTPSAESTPQSTTVKIKNTTTTQILPSKPTTK
                     QRQNKPQNKPNNDFHFEVFNFVPCSICSNNPTCWAICKRIPNKKPGKKTTTKPTKKPT
```

LKTTKKDPKPQTTKTKEALTTKPTGKPTINTTKTNIRTTLLTSNTKGNPEHTSQEEIL
HSTTSEGYPSPSQVYTTSGQEETLHSTTSEGYPSPSQVYTTSEYLSQSLSSSNTTK"

```
ORIGIN
        1 ggaactacat cacaatccac caccatacta gcttcaacaa caccaagtgc tgagtcaacc
       61 ccacaatcca caacagtcaa gatcaaaaac acaacaacaa cccaaatatt acctagcaaa
      121 cccaccacaa aacaacgcca aaacaaacca caaaacaaac ccaacaatga ttttcacttt
      181 gaagtgttca attttgtacc ctgcagcata tgcagcaaca atccaacctg ctgggccatc
      241 tgcaagagaa taccaaacaa aaaacctgga aagaaaacca ccaccaagcc cacaaaaaaa
      301 ccaaccctca agacaaccaa aaaagatccc aaacctcaaa ccacaaaaac aaaggaagca
      361 ctcaccacca agcctacagg aaagccaacc atcaacacca ccaaaacaaa catcagaact
      421 acactgctca cctccaacac caaaggaaat ccagaacaca caagtcaaga ggaaatcctc
      481 cactcaacca cctccgaagg ctatccaagc ccatcccaag tctacacaac atccggtcaa
      541 gaggaaaccc tccactcaac cacctccgaa ggctatccaa gcccatcaca agtctataca
      601 acatccgagt acctatcaca atctctatct tcatccaaca caacaaaatg a
//
LOCUS       KY328048                 651 bp    cRNA    linear   VRL 12-DEC-2016
DEFINITION  Human respiratory syncytial virus A isolate TH-CU/B12664/2015
            attachment glycoprotein gene, partial cds.
ACCESSION   KY328048
VERSION     KY328048
KEYWORDS    .
SOURCE      Human respiratory syncytial virus A
  ORGANISM  Human respiratory syncytial virus A
            Viruses; ssRNA viruses; ssRNA negative-strand viruses;
            Mononegavirales; Pneumoviridae; Orthopneumovirus.
REFERENCE   1  (bases 1 to 651)
  AUTHORS   Thongpan,I., Mauleekoonphairoj,J., Vichiwattana,P., Korkong,S.,
            Vongpunsawad,S. and Poovorawan,Y.
  TITLE     Molecular Characterization of Respiratory Syncytial Virus in
            Thailand, 2012-2015
  JOURNAL   Unpublished
REFERENCE   2  (bases 1 to 651)
  AUTHORS   Thongpan,I., Mauleekoonphairoj,J., Vichiwattana,P., Korkong,S.,
            Vongpunsawad,S. and Poovorawan,Y.
  TITLE     Direct Submission
  JOURNAL   Submitted (13-DEC-2016) Department of Pediatrics, Center of
            Excellence in Clinical Virology, Faculty of Medicine, Chulalongkorn
            University, Bangkok 10330, Thailand
COMMENT     ##Assembly-Data-START##
            Assembly Method       :: DNASTAR-Lasergene v. 6
            Sequencing Technology :: Sanger dideoxy sequencing
            ##Assembly-Data-END##
FEATURES             Location/Qualifiers
     source          1..651
                     /organism="Human respiratory syncytial virus A"
                     /mol_type="viral cRNA"
                     /isolate="TH-CU/B12664/2015"
                     /host="Homo sapiens"
                     /db_xref="taxon:208893"
                     /country="Thailand"
                     /collection_date="19-Aug-2015"
                     /note="group: A"
     CDS             <1..651
                     /note="G protein"
```

```
                     /codon_start=1
                     /product="attachment glycoprotein"
                     /protein_id="APY20389"
                     /translation="GTTSQSTTILASTTPSAESTPQSTTVKIKNTTTTQILPSKPTTK
                     QRQNKPQNKPNNDFHFEVFNFVPCSICSNNPTCWAICKRIPNKKPGKKTTTKPTKKPT
                     LKTTKKDPKPQTTKTKEALTTKPTGKPTINTTKTNIRTTLLTSNTKGNPEHTSQEEII
                     HSTTSEGYPSPSQVYTTSGQEETLHSTTSEGYPSPSQVYTTSEYLSQSLSSSNTTK"
ORIGIN
        1 ggaactacat cacaatccac caccatacta gcttcaacaa caccaagtgc tgagtcaacc
       61 ccacaatcca caacagtcaa gatcaaaaac acaacaacaa cccaaatatt acctagcaaa
      121 cccaccacaa aacaacgcca aaacaaacca caaaacaaac ccaacaatga ttttcacttt
      181 gaagtgttca attttgtacc ctgcagcata tgcagcaaca atccaacctg ctgggccatc
      241 tgcaagagaa taccaaacaa aaaacctgga aagaaaacca ccaccaagcc cacaaaaaaa
      301 ccaaccctca agacaaccaa aaaagatccc aaacctcaaa ccacaaaaac aaaggaagca
      361 ctcaccacca agcctacagg aaagccaacc atcaacacca ccaaaacaaa catcagaact
      421 acactgctca cctccaacac caaaggaaat ccagaacaca caagtcaaga ggaaatcatc
      481 cactcaacca cctccgaagg ctatccaagc ccatcccaag tctacacaac atccggtcaa
      541 gaggaaaccc tccactcaac cacctccgaa ggctatccaa gcccatcaca agtctataca
      601 acatccgagt acctatcaca atctctatct tcatccaaca caacaaaatg a
//
LOCUS       KY328049                 601 bp    cRNA    linear   VRL 13-DEC-2016
DEFINITION  Human respiratory syncytial virus A isolate ON2/TH-CU/B10640/2014
            attachment glycoprotein gene, partial cds.
ACCESSION   KY328049
VERSION     KY328049
KEYWORDS    .
SOURCE      Human respiratory syncytial virus A
  ORGANISM  Human respiratory syncytial virus A
            Viruses; ssRNA viruses; ssRNA negative-strand viruses;
            Mononegavirales; Pneumoviridae; Orthopneumovirus.
REFERENCE   1  (bases 1 to 601)
  AUTHORS   Thongpan,I., Mauleekoonphairoj,J., Vichiwattana,P., Korkong,S.,
            Vongpunsawad,S. and Poovorawan,Y.
  TITLE     Molecular Characterization of Respiratory Syncytial Virus in
            Thailand, 2012-2015
  JOURNAL   Unpublished
REFERENCE   2  (bases 1 to 601)
  AUTHORS   Thongpan,I., Mauleekoonphairoj,J., Vichiwattana,P., Korkong,S.,
            Vongpunsawad,S. and Poovorawan,Y.
  TITLE     Direct Submission
  JOURNAL   Submitted (13-DEC-2016) Department of Pediatrics, Center of
            Excellence in Clinical Virology, Faculty of Medicine, Chulalongkorn
            University, Bangkok 10330, Thailand
COMMENT     ##Assembly-Data-START##
            Assembly Method       :: DNASTAR-Lasergene v. 6
            Sequencing Technology :: Sanger dideoxy sequencing
            ##Assembly-Data-END##
FEATURES             Location/Qualifiers
     source          1..601
                     /organism="Human respiratory syncytial virus A"
                     /mol_type="viral cRNA"
                     /isolate="ON2/TH-CU/B10640/2014"
                     /host="Homo sapiens"
                     /db_xref="taxon:208893"
```

/country="Thailand"
/collection_date="12-Aug-2014"
/note="group: A"
CDS <1..>601
/note="G protein"
/codon_start=1
/product="attachment glycoprotein"
/protein_id="APY20390"
/translation="ISFSNLSGTTSQSTTILASTTPSAESTPQSTTVKIKNTTTTQIL
PSKPTTKQRQNKPQNKPNNDFHFEVFNFVPCSICSNNPTCWAICKRIPNKKPGKKTTT
KPTKKPTLKTTKKDPKPQTTKPKEVLTTKPTGRPTINTTKTNIRTTLLTSNTKGNPEH
TSQEETLHSTTSEGYLSPSHVYTTSGQEETLHSTTSEGYL"
ORIGIN
1 atcagcttct ccaatctgtc cggaactaca tcacaatcca ccaccatact agcttcaaca
61 acaccaagtg ctgagtcaac cccacaatcc acaacagtca agatcaaaaa cacaacaaca
121 acccaaatat tacctagcaa acccaccaca aaacaacgcc aaaataaacc acaaaacaaa
181 cccaacaatg attttcactt tgaagtgttc aattttgtac cctgcagcat atgcagcaac
241 aatccaacct gctgggccat ctgcaagaga ataccaaaca aaaaacctgg aaagaaaacc
301 accaccaagc ccacaaaaaa accaaccctc aagacaacca aaaaagatcc caaacctcaa
361 accacaaaac caaaggaagt actcactacc aagcccacag gaaggccaac catcaacacc
421 actaaaacaa acatcagaac tacactgctc acctccaaca ccaaaggaaa tccagaacac
481 acaagtcaag aggaaaccct ccactcaacc acctccgaag gctatctaag cccatcccat
541 gtctatacaa catccgggca agaggaaacc ctccactcaa ccacctccga aggctatcta
601 a
//
LOCUS KY328050 648 bp cRNA linear VRL 13-DEC-2016
DEFINITION Human respiratory syncytial virus A isolate TH-CU/B10806/2014
attachment glycoprotein gene, partial cds.
ACCESSION KY328050
VERSION KY328050
KEYWORDS .
SOURCE Human respiratory syncytial virus A
ORGANISM Human respiratory syncytial virus A
Viruses; ssRNA viruses; ssRNA negative-strand viruses;
Mononegavirales; Pneumoviridae; Orthopneumovirus.
REFERENCE 1 (bases 1 to 648)
AUTHORS Thongpan,I., Mauleekoonphairoj,J., Vichiwattana,P., Korkong,S.,
Vongpunsawad,S. and Poovorawan,Y.
TITLE Molecular Characterization of Respiratory Syncytial Virus in
Thailand, 2012-2015
JOURNAL Unpublished
REFERENCE 2 (bases 1 to 648)
AUTHORS Thongpan,I., Mauleekoonphairoj,J., Vichiwattana,P., Korkong,S.,
Vongpunsawad,S. and Poovorawan,Y.
TITLE Direct Submission
JOURNAL Submitted (13-DEC-2016) Department of Pediatrics, Center of
Excellence in Clinical Virology, Faculty of Medicine, Chulalongkorn
University, Bangkok 10330, Thailand
COMMENT ##Assembly-Data-START##
Assembly Method :: DNASTAR-Lasergene v. 6
Sequencing Technology :: Sanger dideoxy sequencing
##Assembly-Data-END##
FEATURES Location/Qualifiers
source 1..648

```
                     /organism="Human respiratory syncytial virus A"
                     /mol_type="viral cRNA"
                     /isolate="TH-CU/B10806/2014"
                     /host="Homo sapiens"
                     /db_xref="taxon:208893"
                     /country="Thailand"
                     /collection_date="27-Aug-2014"
                     /note="group: A"
     CDS             <1..>648
                     /note="G protein"
                     /codon_start=1
                     /product="attachment glycoprotein"
                     /protein_id="APY20391"
                     /translation="QNPQLGISFSNLSGTTSQSTTILASTTPSAESTPQSTTVKIKNT
                     TTTQILPSKTTTKQHQNKPQNKPNNDFHFEVFNFVPCSICSNNPTCWTICKRIPNKKP
                     GKKTTTKPTKKPTLKTTKKDPKPQTTKPKEVLTTKPTGKPTINTTKTNIRTTLLTSNT
                     KGNPEHTSQEETLHSTTSEGYLSPSQVYTTSGQEETLHSTTSEGYLSPSQVYTTSG"
ORIGIN
        1 cagaatcccc agcttggaat cagcttctcc aatctgtccg gaactacatc acaatccacc
       61 accatactag cttcaacaac accaagtgct gagtcaaccc cacaatccac aacagtcaag
      121 atcaaaaaca caacaacaac ccaaatatta cctagcaaaa ccaccacaaa acaacaccaa
      181 aataaaccac aaaacaaacc caacaatgat tttcactttg aagtgttcaa ttttgtaccc
      241 tgcagcatat gcagcaacaa tccaacctgc tggaccatct gcaagagaat accaaacaaa
      301 aaacctggaa agaaaaccac caccaagccc acaaaaaaac caaccctcaa gacaaccaaa
      361 aaagatccca aacctcaaac cacaaaacca aaggaagtac tcactaccaa gcctacagga
      421 aagccaacca tcaacaccac taaaacaaac atcagaacta cactgctcac ctccaacacc
      481 aaaggaaatc cagaacacac aagtcaagag gaaaccctcc actcaaccac ctccgaaggc
      541 tatctaagcc catcacaagt ctatacaaca tccggtcaag aggaaaccct ccactcaacc
      601 acctccgaag gttatctaag cccatcacaa gtctatacaa cctccgga
//
LOCUS       KY328051                 653 bp    cRNA    linear   VRL 13-DEC-2016
DEFINITION  Human respiratory syncytial virus A isolate TH-CU/B10995/2014
            attachment glycoprotein gene, partial cds.
ACCESSION   KY328051
VERSION     KY328051
KEYWORDS    .
SOURCE      Human respiratory syncytial virus A
  ORGANISM  Human respiratory syncytial virus A
            Viruses; ssRNA viruses; ssRNA negative-strand viruses;
            Mononegavirales; Pneumoviridae; Orthopneumovirus.
REFERENCE   1  (bases 1 to 653)
  AUTHORS   Thongpan,I., Mauleekoonphairoj,J., Vichiwattana,P., Korkong,S.,
            Vongpunsawad,S. and Poovorawan,Y.
  TITLE     Molecular Characterization of Respiratory Syncytial Virus in
            Thailand, 2012-2015
  JOURNAL   Unpublished
REFERENCE   2  (bases 1 to 653)
  AUTHORS   Thongpan,I., Mauleekoonphairoj,J., Vichiwattana,P., Korkong,S.,
            Vongpunsawad,S. and Poovorawan,Y.
  TITLE     Direct Submission
  JOURNAL   Submitted (13-DEC-2016) Department of Pediatrics, Center of
            Excellence in Clinical Virology, Faculty of Medicine, Chulalongkorn
            University, Bangkok 10330, Thailand
COMMENT     ##Assembly-Data-START##
```

```
          Assembly Method       :: DNASTAR-Lasergene v. 6
          Sequencing Technology :: Sanger dideoxy sequencing
          ##Assembly-Data-END##
FEATURES             Location/Qualifiers
     source          1..653
                     /organism="Human respiratory syncytial virus A"
                     /mol_type="viral cRNA"
                     /isolate="TH-CU/B10995/2014"
                     /host="Homo sapiens"
                     /db_xref="taxon:208893"
                     /country="Thailand"
                     /collection_date="13-Sep-2014"
                     /note="group: A"
     CDS             <1..>653
                     /note="G protein"
                     /codon_start=1
                     /product="attachment glycoprotein"
                     /protein_id="APY20392"
                     /translation="PQLGISFSNLSGTTSQSTTILASTTPSAESTPQSTTVKIKNTTT
                     TQILPSKPTTKQRQNKPQNKPNNDFHFEVFNFVPCSICSNNPTCWAICKRIPNKKPGK
                     KTTTKPTKKPTLKTTKKDPKPQTTKPKEVLTTKPTGKPTINTTKTNIRTTLLTSNTKG
                     NPEHTSQEETLHSTTSEGYLSPSQVYTTSGQEETLHSTTSEGYLSPSQVYTTSEYLS"
ORIGIN
        1 ccccagcttg gaatcagctt ctccaatctg tccggaacta catcacaatc caccaccata
       61 ctagcttcaa caacaccaag tgctgagtca accccacaat ccacaacagt caagatcaaa
      121 aacacaacaa caacccaaat attacctagc aaacccacca caaaacaacg ccaaaataaa
      181 ccacaaaaca aacccaacaa tgattttcac tttgaagtgt tcaattttgt accctgcagc
      241 atatgcagca acaatccaac ctgctgggcc atctgcaaga gaataccaaa caaaaaacct
      301 ggaaagaaaa ccaccaccaa gcccacaaaa aaaccaaccc tcaagacaac caaaaaagat
      361 cccaaacctc aaaccacaaa accaaaggaa gtactcacta ccaagcctac aggaaagcca
      421 accatcaaca ccactaaaac aaacatcaga actacactgc tcacctccaa caccaaagga
      481 aatccagaac acacaagtca agaggaaacc ctccactcaa ccacctccga aggctatcta
      541 agcccatcac aagtctatac aacatccggt caagaggaaa ccctccactc aaccacctcc
      601 gaaggctatc taagcccatc acaagtctat acaacatccg agtacctatc aca
//
LOCUS       KY328052                 333 bp    cRNA    linear   VRL 13-DEC-2016
DEFINITION  Human respiratory syncytial virus A isolate TH-CU/B12350/2015
            attachment glycoprotein gene, partial cds.
ACCESSION   KY328052
VERSION     KY328052
KEYWORDS    .
SOURCE      Human respiratory syncytial virus A
  ORGANISM  Human respiratory syncytial virus A
            Viruses; ssRNA viruses; ssRNA negative-strand viruses;
            Mononegavirales; Pneumoviridae; Orthopneumovirus.
REFERENCE   1  (bases 1 to 333)
  AUTHORS   Thongpan,I., Mauleekoonphairoj,J., Vichiwattana,P., Korkong,S.,
            Vongpunsawad,S. and Poovorawan,Y.
  TITLE     Molecular Characterization of Respiratory Syncytial Virus in
            Thailand, 2012-2015
  JOURNAL   Unpublished
REFERENCE   2  (bases 1 to 333)
  AUTHORS   Thongpan,I., Mauleekoonphairoj,J., Vichiwattana,P., Korkong,S.,
            Vongpunsawad,S. and Poovorawan,Y.
```

```
  TITLE     Direct Submission
  JOURNAL   Submitted (13-DEC-2016) Department of Pediatrics, Center of
            Excellence in Clinical Virology, Faculty of Medicine, Chulalongkorn
            University, Bangkok 10330, Thailand
COMMENT     ##Assembly-Data-START##
            Assembly Method       :: DNASTAR-Lasergene v. 6
            Sequencing Technology :: Sanger dideoxy sequencing
            ##Assembly-Data-END##
FEATURES             Location/Qualifiers
     source          1..333
                     /organism="Human respiratory syncytial virus A"
                     /mol_type="viral cRNA"
                     /isolate="TH-CU/B12350/2015"
                     /host="Homo sapiens"
                     /db_xref="taxon:208893"
                     /country="Thailand"
                     /collection_date="10-Aug-2015"
                     /note="group: A"
     CDS             <1..333
                     /note="G protein"
                     /codon_start=1
                     /product="attachment glycoprotein"
                     /protein_id="APY20393"
                     /translation="KKDPNPQTTKPKEVLTTKPTRKPTINTTKTNIRTTLLTSNTKGN
                     PEHTSQEETLHSTTSKGHPSPSQVHTTSGQEETLHSTTSEGHPSPSQVYTTSEYLSQS
                     PSSSNTTK"
ORIGIN
        1 aaaaaagatc ccaaccctca aaccacaaaa ccaaaggaag tactcactac caagcctaca
       61 agaaagccaa ccatcaacac caccaaaaca aacatcagaa ctacactgct cacctccaac
      121 accaaaggaa atccagaaca cacaagtcaa gaggaaaccc tccactcaac cacctccaaa
      181 ggccatccaa gcccatccca agtccacaca acatccggtc aagaggaaac cctccactca
      241 accacctccg aaggccatcc aagcccatca caagtctata caacatccga gtacctatca
      301 caatctccat cttcatccaa cacaacaaaa tga
//
LOCUS       KY328053                 699 bp    cRNA    linear   VRL 13-DEC-2016
DEFINITION  Human respiratory syncytial virus A isolate ON2/TH-CU/B12560/2015
            attachment glycoprotein gene, partial cds.
ACCESSION   KY328053
VERSION     KY328053
KEYWORDS    .
SOURCE      Human respiratory syncytial virus A
  ORGANISM  Human respiratory syncytial virus A
            Viruses; ssRNA viruses; ssRNA negative-strand viruses;
            Mononegavirales; Pneumoviridae; Orthopneumovirus.
REFERENCE   1  (bases 1 to 699)
  AUTHORS   Thongpan,I., Mauleekoonphairoj,J., Vichiwattana,P., Korkong,S.,
            Vongpunsawad,S. and Poovorawan,Y.
  TITLE     Molecular Characterization of Respiratory Syncytial Virus in
            Thailand, 2012-2015
  JOURNAL   Unpublished
REFERENCE   2  (bases 1 to 699)
  AUTHORS   Thongpan,I., Mauleekoonphairoj,J., Vichiwattana,P., Korkong,S.,
            Vongpunsawad,S. and Poovorawan,Y.
  TITLE     Direct Submission
```

```
  JOURNAL   Submitted (13-DEC-2016) Department of Pediatrics, Center of
            Excellence in Clinical Virology, Faculty of Medicine, Chulalongkorn
            University, Bangkok 10330, Thailand
COMMENT     ##Assembly-Data-START##
            Assembly Method       :: DNASTAR-Lasergene v. 6
            Sequencing Technology :: Sanger dideoxy sequencing
            ##Assembly-Data-END##
FEATURES             Location/Qualifiers
     source          1..699
                     /organism="Human respiratory syncytial virus A"
                     /mol_type="viral cRNA"
                     /isolate="ON2/TH-CU/B12560/2015"
                     /host="Homo sapiens"
                     /db_xref="taxon:208893"
                     /country="Thailand"
                     /collection_date="16-Aug-2015"
                     /note="group: A"
     CDS             <1..699
                     /note="G protein"
                     /codon_start=1
                     /product="attachment glycoprotein"
                     /protein_id="APY20394"
                     /translation="YLTQNPQLGISFSNLSGTTSQSTTILASTTPSAESTPQSTTVKI
                     INTTTTQILPSKPTTKQRQNKPQNKPNNDFHFEVFNFVPCSICSNNPTCWAICKRIPN
                     KKPGKKTTTKPTKKPTLKTTKKDPKPQTTKPKEVLTTKPTGKPTINTTKTNSRTTLLT
                     SNTKGNPEHTSQKETLHSTTSEGYLSPSQVYTTSGQEETLHSTTSEGYPSPSQVYTTS
                     EYLSQSLSSSNTTK"
ORIGIN
        1 tacctcaccc agaatcccca gcttggaatc agcttctcca atctgtccgg aactacatca
       61 caatccacca ccatactagc ttcaacaaca ccaagtgctg agtcaacccc acaatccaca
      121 acagtcaaga tcataaacac aacaacaacc caaatattac ctagcaaacc caccacaaaa
      181 caacgccaaa ataaaccaca aaacaaaccc aacaatgatt ttcactttga agtgttcaat
      241 tttgtaccct gcagcatatg tagcaacaat ccaacctgct gggccatctg caagagaata
      301 ccaaacaaaa aacctggaaa gaaaaccacc accaagccca caaaaaaacc aaccctcaag
      361 acaaccaaaa aagatcccaa acctcaaacc acaaaaccaa aggaagtact cactaccaag
      421 cctacaggaa agccaaccat caacaccact aaaacaaaca gcagaactac actgctcacc
      481 tccaacacca aaggaaatcc agaacacaca agtcaaaagg aaaccctcca ctcaaccacc
      541 tccgaaggct atctaagccc atcacaagtc tatacaacat ccggtcaaga ggaaaccctc
      601 cactcaacca cctccgaagg ctatccaagc ccatcacaag tctatacaac atccgagtac
      661 ctatcacaat ctctatcttc atccaacaca acaaaatga
//
LOCUS       KY328054                 624 bp    cRNA    linear   VRL 13-DEC-2016
DEFINITION  Human respiratory syncytial virus A isolate TH-CU/B10618/2014
            attachment glycoprotein gene, partial cds.
ACCESSION   KY328054
VERSION     KY328054
KEYWORDS    .
SOURCE      Human respiratory syncytial virus A
  ORGANISM  Human respiratory syncytial virus A
            Viruses; ssRNA viruses; ssRNA negative-strand viruses;
            Mononegavirales; Pneumoviridae; Orthopneumovirus.
REFERENCE   1  (bases 1 to 624)
  AUTHORS   Thongpan,I., Mauleekoonphairoj,J., Vichiwattana,P., Korkong,S.,
            Vongpunsawad,S. and Poovorawan,Y.
```

TITLE Molecular Characterization of Respiratory Syncytial Virus in
Thailand, 2012-2015
JOURNAL Unpublished
REFERENCE 2 (bases 1 to 624)
AUTHORS Thongpan,I., Mauleekoonphairoj,J., Vichiwattana,P., Korkong,S.,
Vongpunsawad,S. and Poovorawan,Y.
TITLE Direct Submission
JOURNAL Submitted (13-DEC-2016) Department of Pediatrics, Center of
Excellence in Clinical Virology, Faculty of Medicine, Chulalongkorn
University, Bangkok 10330, Thailand
COMMENT ##Assembly-Data-START##
Assembly Method :: DNASTAR-Lasergene v. 6
Sequencing Technology :: Sanger dideoxy sequencing
##Assembly-Data-END##
FEATURES Location/Qualifiers
source 1..624
/organism="Human respiratory syncytial virus A"
/mol_type="viral cRNA"
/isolate="TH-CU/B10618/2014"
/host="Homo sapiens"
/db_xref="taxon:208893"
/country="Thailand"
/collection_date="08-Aug-2014"
/note="group: A"
CDS <1..624
/note="G protein"
/codon_start=1
/product="attachment glycoprotein"
/protein_id="APY20395"
/translation="LASTTPSAESTPQSTTVKIKNTTTTQILPSKPTTKQRQNKPQNK
PNNDFHFEVFNFVPCSICSNNPTCWAICKRIPNKKPGKKTTTKPTKKPTLKTTKKDPK
PQTTKPKEVLTTKPTGRPTINTTKTNIRTTLFTSNTKGNPEHTSQEETLHSTTSEGYL
SPSQVYTTFGQEETLHSTTSEGYLSSSQVYTTSEYLSQSLSSSNTAK"
ORIGIN
1 ctagcttcaa caacaccaag tgctgagtca accccacaat ccacaacagt caagatcaaa
61 aacacaacaa caacccaaat attacctagc aaacccacca caaaacaacg ccaaaataaa
121 ccacaaaaca aacccaacaa tgattttcac tttgaagtgt tcaattttgt accctgcagc
181 atatgcagca acaatccaac ctgctgggcc atctgcaaga gaataccaaa caaaaaacct
241 ggaaagaaaa ccaccaccaa gcccacaaaa aaaccaaccc tcaagacaac caaaaaagat
301 cccaaacctc aaaccacaaa accaaaggaa gtactcacta ccaagcccac aggaaggcca
361 accatcaaca ccactaaaac aaacatcaga actacactgt tcacctccaa caccaaagga
421 aatccagaac acacaagtca agaggaaacc ctccactcaa ccacctccga aggctatcta
481 agcccatccc aagtctatac aacattcggt caagaggaaa ccctccactc aaccacctcc
541 gaaggctatc taagctcatc acaagtctat acaacatccg agtacctatc acaatctcta
601 tcttcatcca acacagcaaa atga
//
LOCUS KY328055 603 bp cRNA linear VRL 13-DEC-2016
DEFINITION Human respiratory syncytial virus B isolate TH-CU/B10608/2014
attachment glycoprotein gene, partial cds.
ACCESSION KY328055
VERSION KY328055
KEYWORDS .
SOURCE Human respiratory syncytial virus B
ORGANISM Human respiratory syncytial virus B

Viruses; ssRNA viruses; ssRNA negative-strand viruses;
Mononegavirales; Pneumoviridae; Orthopneumovirus.
REFERENCE 1 (bases 1 to 603)
AUTHORS Thongpan,I., Mauleekoonphairoj,J., Vichiwattana,P., Korkong,S.,
Vongpunsawad,S. and Poovorawan,Y.
TITLE Molecular Characterization of Respiratory Syncytial Virus in
Thailand, 2012-2015
JOURNAL Unpublished
REFERENCE 2 (bases 1 to 603)
AUTHORS Thongpan,I., Mauleekoonphairoj,J., Vichiwattana,P., Korkong,S.,
Vongpunsawad,S. and Poovorawan,Y.
TITLE Direct Submission
JOURNAL Submitted (13-DEC-2016) Department of Pediatrics, Center of
Excellence in Clinical Virology, Faculty of Medicine, Chulalongkorn
University, Bangkok 10330, Thailand
COMMENT ##Assembly-Data-START##
Assembly Method :: DNASTAR-Lasergene v. 6
Sequencing Technology :: Sanger dideoxy sequencing
##Assembly-Data-END##
FEATURES Location/Qualifiers
source 1..603
/organism="Human respiratory syncytial virus B"
/mol_type="viral cRNA"
/isolate="TH-CU/B10608/2014"
/host="Homo sapiens"
/db_xref="taxon:208895"
/country="Thailand"
/collection_date="04-Aug-2014"
/note="group: B"
CDS <1..603
/note="G protein"
/codon_start=1
/product="attachment glycoprotein"
/protein_id="APY20396"
/translation="IHTNSATISPNTKSETHHTTAQTKGTTSTPTQNNKPSTKPRPKN
PPKKDDYHFEVFNFVPCSICGNNQLCKSICKTIPSNKPKKKPTTKPTNKPPTKTTNKR
DPKTLAKTPKKETTINPTKKPTPKTTERDTSTPQSTVLDITTSKHTERDTSTSQSIVL
DTTTSKHTTQQQSLYSTTPENTPNSTQTPTASEASTSNST"
ORIGIN
1 atccacacaa actcagccac aatatcaccc aatacaaaat cagaaacaca ccatacaaca
61 gcacaaacca aaggcacaac ctctactcca acacagaaca acaagccaag cacaaaacca
121 cgtccaaaaa atccaccaaa aaaagatgat taccattttg aagtgttcaa ctttgttccc
181 tgtagtatat gtggcaacaa tcaactctgc aaatccattt gcaaaacaat accaagcaat
241 aaaccaaaga aaaaaccaac tacaaaaccc acaaacaaac cacctaccaa aaccacaaac
301 aaaagagacc ccaaaacact agccaaaaca ccgaaaaaag aaaccaccat taacccaaca
361 aaaaaaccaa cccccaagac tacagaaaga gacaccagca ccccacaatc cactgtgctc
421 gacataacca catcaaaaca cacagaaaga gacaccagca cctcacaatc cattgtgctt
481 gacacaacca catcaaaaca cacaacccaa cagcaatctc tctactcaac cacccccgaa
541 aacacaccca actccacgca aacacccaca gcatccgagg cctccacatc aaattctacc
601 taa
//
LOCUS KY328056 603 bp cRNA linear VRL 13-DEC-2016
DEFINITION Human respiratory syncytial virus B isolate TH-CU/B11014/2014
attachment glycoprotein gene, partial cds.

ACCESSION KY328056
VERSION KY328056
KEYWORDS .
SOURCE Human respiratory syncytial virus B
ORGANISM Human respiratory syncytial virus B
Viruses; ssRNA viruses; ssRNA negative-strand viruses;
Mononegavirales; Pneumoviridae; Orthopneumovirus.
REFERENCE 1 (bases 1 to 603)
AUTHORS Thongpan,I., Mauleekoonphairoj,J., Vichiwattana,P., Korkong,S.,
Vongpunsawad,S. and Poovorawan,Y.
TITLE Molecular Characterization of Respiratory Syncytial Virus in
Thailand, 2012-2015
JOURNAL Unpublished
REFERENCE 2 (bases 1 to 603)
AUTHORS Thongpan,I., Mauleekoonphairoj,J., Vichiwattana,P., Korkong,S.,
Vongpunsawad,S. and Poovorawan,Y.
TITLE Direct Submission
JOURNAL Submitted (13-DEC-2016) Department of Pediatrics, Center of
Excellence in Clinical Virology, Faculty of Medicine, Chulalongkorn
University, Bangkok 10330, Thailand
COMMENT ##Assembly-Data-START##
Assembly Method :: DNASTAR-Lasergene v. 6
Sequencing Technology :: Sanger dideoxy sequencing
##Assembly-Data-END##
FEATURES Location/Qualifiers
source 1..603
/organism="Human respiratory syncytial virus B"
/mol_type="viral cRNA"
/isolate="TH-CU/B11014/2014"
/host="Homo sapiens"
/db_xref="taxon:208895"
/country="Thailand"
/collection_date="23-Sep-2014"
/note="group: B"
CDS <1..603
/note="G protein"
/codon_start=1
/product="attachment glycoprotein"
/protein_id="APY20397"
/translation="IHTNSATISPNTKSETHHTTAQTKGTTSTPTQNNKPSTKPRPKN
PPKKDDYHFEVFNFVPCSICGNNQLCKSICKTIPSNKPKKKPTTKPTNKPPTKTTNKR
DPKTLAKTPKKETTINPTKKPTPKTTERDTSTPQSTVLDITTSKHTERDTSTSQSIVL
DTTTSKHTTQQQSLYSTTPENTPNSTQTPTASEPSTSNST"
ORIGIN
1 atccacacaa actcagccac aatatcaccc aatacaaaat cagaaacaca ccatacaaca
61 gcacaaacca aaggcacaac ctctactcca acacagaaca acaagccaag cacaaaacca
121 cgtccaaaaa atccaccaaa aaaagatgat taccattttg aagtgttcaa ctttgttccc
181 tgtagtatat gtggcaacaa tcaactctgc aaatccattt gcaaaacaat accaagcaat
241 aaaccaaaga aaaaaccaac tacaaaaccc acaaacaaac cacctaccaa aaccacaaac
301 aaaagagacc ccaaaacact agccaaaaca ccgaaaaaag aaaccaccat taacccaaca
361 aaaaaaccaa cccccaagac tacagaaaga gacaccagca ccccacaatc cactgtgctc
421 gacataacca catcaaaaca cacagaaaga gacaccagca cctcacaatc cattgtgctt
481 gacacaacca catcaaaaca cacaacccaa cagcaatctc tctactcaac cacccccgaa
541 aacacaccca actccacgca aacacccaca gcatccgagc cctccacatc aaattctacc

```
     601 taa
//
LOCUS       KY328057                 603 bp    cRNA    linear   VRL 13-DEC-2016
DEFINITION  Human respiratory syncytial virus B isolate TH-CU/B10808/2014
            attachment glycoprotein gene, partial cds.
ACCESSION   KY328057
VERSION     KY328057
KEYWORDS    .
SOURCE      Human respiratory syncytial virus B
  ORGANISM  Human respiratory syncytial virus B
            Viruses; ssRNA viruses; ssRNA negative-strand viruses;
            Mononegavirales; Pneumoviridae; Orthopneumovirus.
REFERENCE   1  (bases 1 to 603)
  AUTHORS   Thongpan,I., Mauleekoonphairoj,J., Vichiwattana,P., Korkong,S.,
            Vongpunsawad,S. and Poovorawan,Y.
  TITLE     Molecular Characterization of Respiratory Syncytial Virus in
            Thailand, 2012-2015
  JOURNAL   Unpublished
REFERENCE   2  (bases 1 to 603)
  AUTHORS   Thongpan,I., Mauleekoonphairoj,J., Vichiwattana,P., Korkong,S.,
            Vongpunsawad,S. and Poovorawan,Y.
  TITLE     Direct Submission
  JOURNAL   Submitted (13-DEC-2016) Department of Pediatrics, Center of
            Excellence in Clinical Virology, Faculty of Medicine, Chulalongkorn
            University, Bangkok 10330, Thailand
COMMENT     ##Assembly-Data-START##
            Assembly Method       :: DNASTAR-Lasergene v. 6
            Sequencing Technology :: Sanger dideoxy sequencing
            ##Assembly-Data-END##
FEATURES             Location/Qualifiers
     source          1..603
                     /organism="Human respiratory syncytial virus B"
                     /mol_type="viral cRNA"
                     /isolate="TH-CU/B10808/2014"
                     /host="Homo sapiens"
                     /db_xref="taxon:208895"
                     /country="Thailand"
                     /collection_date="28-Aug-2014"
                     /note="group: B"
     CDS             <1..603
                     /note="G protein"
                     /codon_start=1
                     /product="attachment glycoprotein"
                     /protein_id="APY20398"
                     /translation="IHTNSATISPNTKSETHHTTAQTKGTTSTPTQNNKPSTKPRPKN
                     PPKKDDYHFEVFNFVPCSICGNNQLCKSICKTIPSNKPKKKPTTKPTNKPPTKTTNKR
                     DPKTLAKTPKKETTINPTKKPTPKTTERDTSTPQSTVLDITTSKHTERDTSTSQSIVL
                     DTTTSKHTTQQQSLYSTTPENTPNSTQTPTASEPSTSNST"
ORIGIN
        1 atccacacaa actcagccac aatatcaccc aatacaaaat cagaaacaca ccatacaaca
       61 gcacaaacca aaggcacaac ctctactcca acacagaaca acaagccaag cacaaaacca
      121 cgtccaaaaa atccaccaaa aaaagatgat taccattttg aagtgttcaa ctttgttccc
      181 tgtagtatat gtggcaacaa tcaactctgc aaatccattt gcaaaacaat accaagcaat
      241 aaaccaaaga aaaaaccaac tacaaaaccc acaaacaaac cacctaccaa aaccacaaac
```

```
     301 aaaagagacc ccaaaacact agccaaaaca ccgaaaaaag aaaccaccat taacccaaca
     361 aaaaaaccaa cccccaagac tacagaaaga gacaccagca ccccacaatc cactgtgctc
     421 gacataacca catcaaaaca cacagaaaga gacaccagca cctcacaatc cattgtgctt
     481 gacacaacca catcaaaaca cacaacccaa cagcaatctc tctactcaac cacccccgaa
     541 aacacaccca actccacgca aacacccaca gcatccgagc cctccacatc aaattctacc
     601 taa
//
LOCUS       KY328058                 597 bp    cRNA    linear   VRL 13-DEC-2016
DEFINITION  Human respiratory syncytial virus B isolate TH-CU/C5180/2014
            attachment glycoprotein gene, partial cds.
ACCESSION   KY328058
VERSION     KY328058
KEYWORDS    .
SOURCE      Human respiratory syncytial virus B
  ORGANISM  Human respiratory syncytial virus B
            Viruses; ssRNA viruses; ssRNA negative-strand viruses;
            Mononegavirales; Pneumoviridae; Orthopneumovirus.
REFERENCE   1  (bases 1 to 597)
  AUTHORS   Thongpan,I., Mauleekoonphairoj,J., Vichiwattana,P., Korkong,S.,
            Vongpunsawad,S. and Poovorawan,Y.
  TITLE     Molecular Characterization of Respiratory Syncytial Virus in
            Thailand, 2012-2015
  JOURNAL   Unpublished
REFERENCE   2  (bases 1 to 597)
  AUTHORS   Thongpan,I., Mauleekoonphairoj,J., Vichiwattana,P., Korkong,S.,
            Vongpunsawad,S. and Poovorawan,Y.
  TITLE     Direct Submission
  JOURNAL   Submitted (13-DEC-2016) Department of Pediatrics, Center of
            Excellence in Clinical Virology, Faculty of Medicine, Chulalongkorn
            University, Bangkok 10330, Thailand
COMMENT     ##Assembly-Data-START##
            Assembly Method       :: DNASTAR-Lasergene v. 6
            Sequencing Technology :: Sanger dideoxy sequencing
            ##Assembly-Data-END##
FEATURES             Location/Qualifiers
     source          1..597
                     /organism="Human respiratory syncytial virus B"
                     /mol_type="viral cRNA"
                     /isolate="TH-CU/C5180/2014"
                     /host="Homo sapiens"
                     /db_xref="taxon:208895"
                     /country="Thailand"
                     /collection_date="30-Oct-2014"
                     /note="group: B"
     CDS             <1..597
                     /note="G protein"
                     /codon_start=1
                     /product="attachment glycoprotein"
                     /protein_id="APY20399"
                     /translation="TNSATISPNTKSETHHTTAQTKGTTSTPTQNNKPSTKPRPKNPP
                     KKDDYHFEVFNFVPCSICGNNQLCKSICKTIPSNKPKKKPTTKPTNKPPTKTTNKRDP
                     KTLAKTPKKETTINPTKKPTPKTTERDTSTPQSTVLDITTSKHTERDTSTSQSIALDT
                     TTSKHTTQQQSLYSTTPENTPNSTQTPTASEPSTSNST"
ORIGIN
```

```
        1 acaaactcag ccacaatatc gcccaataca aaatcagaaa cacaccatac aacagcacaa
       61 accaaaggca caacctctac tccaacacag aacaacaagc caagcacaaa accacgtcca
      121 aaaaatccac caaaaaaaga tgattaccat tttgaagtgt tcaactttgt tccctgtagt
      181 atatgtggca acaatcaact ctgcaaatcc atttgcaaaa caataccaag caataaacca
      241 aagaaaaaac caactacaaa acccacaaac aaaccaccta ccaaaaccac aaacaaaaga
      301 gaccccaaaa cactagccaa aacaccgaag aaagaaacca ccattaaccc aacaaaaaaa
      361 ccaacccccca agactacaga aagagacacc agcaccccac aatccactgt gctcgacata
      421 accacatcaa aacacacaga aagagacacc agcacctcac aatccattgc gcttgacaca
      481 accacatcaa aacacacaac ccaacagcaa tctctctact caaccacccc cgaaaacaca
      541 cccaactcca cacaaacacc cacagcatcc gagccctcca catcaaattc tacctaa
//
LOCUS       KY328059                 603 bp    cRNA    linear   VRL 13-DEC-2016
DEFINITION  Human respiratory syncytial virus B isolate TH-CU/C5066/2014
            attachment glycoprotein gene, partial cds.
ACCESSION   KY328059
VERSION     KY328059
KEYWORDS    .
SOURCE      Human respiratory syncytial virus B
  ORGANISM  Human respiratory syncytial virus B
            Viruses; ssRNA viruses; ssRNA negative-strand viruses;
            Mononegavirales; Pneumoviridae; Orthopneumovirus.
REFERENCE   1  (bases 1 to 603)
  AUTHORS   Thongpan,I., Mauleekoonphairoj,J., Vichiwattana,P., Korkong,S.,
            Vongpunsawad,S. and Poovorawan,Y.
  TITLE     Molecular Characterization of Respiratory Syncytial Virus in
            Thailand, 2012-2015
  JOURNAL   Unpublished
REFERENCE   2  (bases 1 to 603)
  AUTHORS   Thongpan,I., Mauleekoonphairoj,J., Vichiwattana,P., Korkong,S.,
            Vongpunsawad,S. and Poovorawan,Y.
  TITLE     Direct Submission
  JOURNAL   Submitted (13-DEC-2016) Department of Pediatrics, Center of
            Excellence in Clinical Virology, Faculty of Medicine, Chulalongkorn
            University, Bangkok 10330, Thailand
COMMENT     ##Assembly-Data-START##
            Assembly Method       :: DNASTAR-Lasergene v. 6
            Sequencing Technology :: Sanger dideoxy sequencing
            ##Assembly-Data-END##
FEATURES             Location/Qualifiers
     source          1..603
                     /organism="Human respiratory syncytial virus B"
                     /mol_type="viral cRNA"
                     /isolate="TH-CU/C5066/2014"
                     /host="Homo sapiens"
                     /db_xref="taxon:208895"
                     /country="Thailand"
                     /collection_date="10-Sep-2014"
                     /note="group: B"
     CDS             <1..603
                     /note="G protein"
                     /codon_start=1
                     /product="attachment glycoprotein"
                     /protein_id="APY20400"
                     /translation="IHTNSATISPNTKSETHHTTAQTKGTTSTPTQNNKPSTKPRPKN
```

```
                 PPKKDDYHFEVFNFVPCSICGNNQLCKSICKTIPSNKPKKKPTTKPTNKPPTKTTNKR
                 DPKTLAKTPKKETTINPTKKPTPKTTERDTSTPQSTVLDITTSKHTERDTSTSQSIAL
                 DTTTSKHTTQQQSLHSTTPENTPNSTQTPAASEPSTSNST"
ORIGIN
        1 atccacacaa actcagccac aatatcaccc aatacaaaat cagaaacaca ccatacaaca
       61 gcacaaacca aaggcacaac ctctactcca acacagaaca acaagccaag cacaaaacca
      121 cgtccaaaaa atccaccaaa aaaagatgat taccattttg aagtattcaa ctttgttccc
      181 tgtagtatat gtggcaacaa tcaactctgc aaatccattt gcaaaacaat accaagcaat
      241 aaaccaaaga aaaaaccaac tacaaaaccc acaaacaaac cacctaccaa aaccacaaac
      301 aaaagagacc ccaaaacact agccaaaaca ccgaaaaaag aaaccaccat taacccaaca
      361 aaaaaaccaa cccccaagac tacagaaaga gacaccagca ccccacaatc cactgtgctc
      421 gacataacca catcaaaaca cacagaaaga gataccagca cctcacaatc cattgcgctt
      481 gacacaacca catcaaaaca cacaacccaa cagcaatctc tccactcaac cacccccgaa
      541 aacacaccca actccacaca aacacccgca gcatccgagc cctccacatc aaattctacc
      601 taa
//
LOCUS       KY328060                 603 bp    cRNA    linear   VRL 13-DEC-2016
DEFINITION  Human respiratory syncytial virus B isolate TH-CU/C5182/2014
            attachment glycoprotein gene, partial cds.
ACCESSION   KY328060
VERSION     KY328060
KEYWORDS    .
SOURCE      Human respiratory syncytial virus B
  ORGANISM  Human respiratory syncytial virus B
            Viruses; ssRNA viruses; ssRNA negative-strand viruses;
            Mononegavirales; Pneumoviridae; Orthopneumovirus.
REFERENCE   1  (bases 1 to 603)
  AUTHORS   Thongpan,I., Mauleekoonphairoj,J., Vichiwattana,P., Korkong,S.,
            Vongpunsawad,S. and Poovorawan,Y.
  TITLE     Molecular Characterization of Respiratory Syncytial Virus in
            Thailand, 2012-2015
  JOURNAL   Unpublished
REFERENCE   2  (bases 1 to 603)
  AUTHORS   Thongpan,I., Mauleekoonphairoj,J., Vichiwattana,P., Korkong,S.,
            Vongpunsawad,S. and Poovorawan,Y.
  TITLE     Direct Submission
  JOURNAL   Submitted (13-DEC-2016) Department of Pediatrics, Center of
            Excellence in Clinical Virology, Faculty of Medicine, Chulalongkorn
            University, Bangkok 10330, Thailand
COMMENT     ##Assembly-Data-START##
            Assembly Method       :: DNASTAR-Lasergene v. 6
            Sequencing Technology :: Sanger dideoxy sequencing
            ##Assembly-Data-END##
FEATURES             Location/Qualifiers
     source          1..603
                     /organism="Human respiratory syncytial virus B"
                     /mol_type="viral cRNA"
                     /isolate="TH-CU/C5182/2014"
                     /host="Homo sapiens"
                     /db_xref="taxon:208895"
                     /country="Thailand"
                     /collection_date="01-Oct-2014"
                     /note="group: B"
     CDS             <1..603
```

```
                     /note="G protein"
                     /codon_start=1
                     /product="attachment glycoprotein"
                     /protein_id="APY20401"
                     /translation="IHTNSATISPNTKSETHHTTAQTKGTTSTPTQNNKPSTKPRPKN
                     PPKKDDYHFEVFNFVPCSICGNNQLCKSICKTIPSNKPKKKPTTKPTNKPPTKTTNKR
                     DPKTLAKTPKKETTINPTKKPTPKTTERDTSTPQSTVLDITTSKHTERDTSTSQSIAL
                     DTTTSKHTTQQQSLHSTTPENTPNSTQTPAASEPSTSNST"
ORIGIN
        1 atccacacaa actcagccac aatatcaccc aatacaaaat cagaaacaca ccatacaaca
       61 gcacaaacca aaggcacaac ctctactcca acacagaaca acaagccaag cacaaaacca
      121 cgtccaaaaa atccaccaaa aaaagatgat taccattttg aagtgttcaa ctttgttccc
      181 tgtagtatat gtggcaacaa tcaactctgc aaatccattt gcaaaacaat accaagcaat
      241 aaaccaaaga aaaaaccaac tacaaaaccc acaaacaaac cacctaccaa aaccacaaac
      301 aaaagagacc ccaaaacact agccaaaaca ccgaaaaaag aaaccaccat taacccaaca
      361 aaaaaaccaa cccccaagac tacagaaaga gacaccagca ccccacaatc cactgtgctc
      421 gacataacca catcaaaaca cacagaaaga gataccagca cctcacaatc cattgcgctt
      481 gacacaacca catcaaaaca cacaacccaa cagcaatctc tccactcaac cacccccgaa
      541 aacacaccca actccacaca aacacccgca gcatccgagc cctccacatc aaattctacc
      601 taa
//
LOCUS       KY328061                 603 bp    cRNA    linear   VRL 13-DEC-2016
DEFINITION  Human respiratory syncytial virus B isolate TH-CU/B10635/2014
            attachment glycoprotein gene, partial cds.
ACCESSION   KY328061
VERSION     KY328061
KEYWORDS    .
SOURCE      Human respiratory syncytial virus B
  ORGANISM  Human respiratory syncytial virus B
            Viruses; ssRNA viruses; ssRNA negative-strand viruses;
            Mononegavirales; Pneumoviridae; Orthopneumovirus.
REFERENCE   1  (bases 1 to 603)
  AUTHORS   Thongpan,I., Mauleekoonphairoj,J., Vichiwattana,P., Korkong,S.,
            Vongpunsawad,S. and Poovorawan,Y.
  TITLE     Molecular Characterization of Respiratory Syncytial Virus in
            Thailand, 2012-2015
  JOURNAL   Unpublished
REFERENCE   2  (bases 1 to 603)
  AUTHORS   Thongpan,I., Mauleekoonphairoj,J., Vichiwattana,P., Korkong,S.,
            Vongpunsawad,S. and Poovorawan,Y.
  TITLE     Direct Submission
  JOURNAL   Submitted (13-DEC-2016) Department of Pediatrics, Center of
            Excellence in Clinical Virology, Faculty of Medicine, Chulalongkorn
            University, Bangkok 10330, Thailand
COMMENT     ##Assembly-Data-START##
            Assembly Method       :: DNASTAR-Lasergene v. 6
            Sequencing Technology :: Sanger dideoxy sequencing
            ##Assembly-Data-END##
FEATURES             Location/Qualifiers
     source          1..603
                     /organism="Human respiratory syncytial virus B"
                     /mol_type="viral cRNA"
                     /isolate="TH-CU/B10635/2014"
                     /host="Homo sapiens"
```

/db_xref="taxon:208895"
/country="Thailand"
/collection_date="11-Aug-2014"
/note="group: B"
CDS <1..603
/note="G protein"
/codon_start=1
/product="attachment glycoprotein"
/protein_id="APY20402"
/translation="IHTNSATISPNTKSETHHTTAQTKGTTSTPTQNNKPSTKPRPKN
PPKKDDYHFEVFNFVPCSICGNNQLCKSICKTIPSNKPKKKPTTKPTNKPPTKTTNKR
DPKTLAKTPKKETTINPTKKPTPKTTERDTSTPQSTVLDITTSKHTERDTSTSQSIAL
DTTTSKHTTQQQSLHSTTPENTPNSTQTPAASEPSTSNST"
ORIGIN
1 atccacacaa actcagccac aatatcaccc aatacaaaat cagaaacaca ccatacaaca
61 gcacaaacca aaggcacaac ctctactcca acacagaaca acaagccaag cacaaaacca
121 cgtccaaaaa atccaccaaa aaaagatgat taccattttg aagtgttcaa ctttgttccc
181 tgtagtatat gtggcaacaa tcaactctgc aaatccattt gcaaaacaat accaagcaat
241 aaaccaaaga aaaaaccaac tacaaaaccc acaaacaaac cacctaccaa aaccacaaac
301 aaaagagacc ccaaaacact agccaaaaca ccgaaaaaag aaaccaccat taacccaaca
361 aaaaaaccaa cccccaagac tacagaaaga gacaccagca ccccacaatc cactgtgctc
421 gacataacca catcaaaaca cacagaaaga gataccagca cctcacaatc cattgcgctt
481 gacacaacca catcaaaaca cacaacccaa cagcaatctc tccactcaac cacccccgaa
541 aacacaccca actccacaca aacacccgca gcatccgagc cctccacatc aaattctacc
601 taa
//
LOCUS KY328062 603 bp cRNA linear VRL 13-DEC-2016
DEFINITION Human respiratory syncytial virus B isolate TH-CU/C5239/2014
attachment glycoprotein gene, partial cds.
ACCESSION KY328062
VERSION KY328062
KEYWORDS .
SOURCE Human respiratory syncytial virus B
ORGANISM Human respiratory syncytial virus B
Viruses; ssRNA viruses; ssRNA negative-strand viruses;
Mononegavirales; Pneumoviridae; Orthopneumovirus.
REFERENCE 1 (bases 1 to 603)
AUTHORS Thongpan,I., Mauleekoonphairoj,J., Vichiwattana,P., Korkong,S.,
Vongpunsawad,S. and Poovorawan,Y.
TITLE Molecular Characterization of Respiratory Syncytial Virus in
Thailand, 2012-2015
JOURNAL Unpublished
REFERENCE 2 (bases 1 to 603)
AUTHORS Thongpan,I., Mauleekoonphairoj,J., Vichiwattana,P., Korkong,S.,
Vongpunsawad,S. and Poovorawan,Y.
TITLE Direct Submission
JOURNAL Submitted (13-DEC-2016) Department of Pediatrics, Center of
Excellence in Clinical Virology, Faculty of Medicine, Chulalongkorn
University, Bangkok 10330, Thailand
COMMENT ##Assembly-Data-START##
Assembly Method :: DNASTAR-Lasergene v. 6
Sequencing Technology :: Sanger dideoxy sequencing
##Assembly-Data-END##
FEATURES Location/Qualifiers

```
     source          1..603
                     /organism="Human respiratory syncytial virus B"
                     /mol_type="viral cRNA"
                     /isolate="TH-CU/C5239/2014"
                     /host="Homo sapiens"
                     /db_xref="taxon:208895"
                     /country="Thailand"
                     /collection_date="01-Nov-2014"
                     /note="group: B"
     CDS             <1..603
                     /note="G protein"
                     /codon_start=1
                     /product="attachment glycoprotein"
                     /protein_id="APY20403"
                     /translation="IHTNSATISPNTKSETHHTTAQTKGTTSTPTQNNKPSTKPRPKN
                     PPKKDDYHFEVFNFVPCSICGNNQLCKSICKTIPSNKPKKKPTTKPTNKPPTKTTNKR
                     DPKTLAKTPKKETTINPTKKPTPKTTERDTSTPQSTVLDITTSKHTERDTSTSQSIAL
                     DTTTSKHTTQQQSLHSTTPENTPNSTQTPAASEPSTSNST"
ORIGIN
        1 atccacacaa actcagccac aatatcaccc aatacaaaat cagaaacaca ccatacaaca
       61 gcacaaacca aaggcacaac ctctactcca acacagaaca acaagccaag cacaaaacca
      121 cgtccaaaaa atccaccaaa aaaagatgat taccattttg aagtgttcaa ctttgttccc
      181 tgtagtatat gtggcaacaa tcaactctgc aaatccattt gcaaaacaat accaagcaat
      241 aaaccaaaga aaaaaccaac tacaaaaccc acaaacaaac cacctaccaa aaccacaaac
      301 aaaagagacc ccaaaacact agccaaaaca ccgaaaaaag aaaccaccat taacccaaca
      361 aaaaaaccaa cccccaagac tacagaaaga gacaccagca ccccacaatc cactgtgctc
      421 gacataacca catcaaaaca cacagaaaga gataccagca cctcacaatc cattgcgctt
      481 gacacaacca catcaaaaca cacaacccaa cagcaatctc tccactcaac cacccccgaa
      541 aacacaccca actccacaca aacacccgca gcatccgagc cctccacatc aaattctacc
      601 taa
//
LOCUS       KY328063                 603 bp    cRNA    linear   VRL 13-DEC-2016
DEFINITION  Human respiratory syncytial virus B isolate TH-CU/C5205/2014
            attachment glycoprotein gene, partial cds.
ACCESSION   KY328063
VERSION     KY328063
KEYWORDS    .
SOURCE      Human respiratory syncytial virus B
  ORGANISM  Human respiratory syncytial virus B
            Viruses; ssRNA viruses; ssRNA negative-strand viruses;
            Mononegavirales; Pneumoviridae; Orthopneumovirus.
REFERENCE   1  (bases 1 to 603)
  AUTHORS   Thongpan,I., Mauleekoonphairoj,J., Vichiwattana,P., Korkong,S.,
            Vongpunsawad,S. and Poovorawan,Y.
  TITLE     Molecular Characterization of Respiratory Syncytial Virus in
            Thailand, 2012-2015
  JOURNAL   Unpublished
REFERENCE   2  (bases 1 to 603)
  AUTHORS   Thongpan,I., Mauleekoonphairoj,J., Vichiwattana,P., Korkong,S.,
            Vongpunsawad,S. and Poovorawan,Y.
  TITLE     Direct Submission
  JOURNAL   Submitted (13-DEC-2016) Department of Pediatrics, Center of
            Excellence in Clinical Virology, Faculty of Medicine, Chulalongkorn
            University, Bangkok 10330, Thailand
```

COMMENT   ##Assembly-Data-START##
            Assembly Method   :: DNASTAR-Lasergene v. 6
            Sequencing Technology :: Sanger dideoxy sequencing
            ##Assembly-Data-END##
FEATURES   Location/Qualifiers
     source   1..603
                     /organism="Human respiratory syncytial virus B"
                     /mol_type="viral cRNA"
                     /isolate="TH-CU/C5205/2014"
                     /host="Homo sapiens"
                     /db_xref="taxon:208895"
                     /country="Thailand"
                     /collection_date="01-Oct-2014"
                     /note="group: B"
     CDS   <1..603
                     /note="G protein"
                     /codon_start=1
                     /product="attachment glycoprotein"
                     /protein_id="APY20404"
                     /translation="IHTNSATISPNTKSETHHTTAQTKGTTSTPTQNNKPSTKPRPKN
                     PPKKDDYHFEVFNFVPCSICGNNQLCKSICKTIPSNKPKKKPTTKPTNKPPTKTTNKR
                     DPKTLAKTPKKETTINPTKKPTPKTTERDTSTPQSTVLDITTSKHTERDTSTSQSIVP
                     DTTTSKHTTQQQSLHSTTPENTPNSTQTPTASEPSTSNST"
ORIGIN
        1 atccacacaa actcagccac aatatcaccc aatacaaaat cagaaacaca ccatacaaca
       61 gcacaaacca aaggcacaac ctctactcca acacagaaca acaagccaag cacaaaacca
      121 cgtccaaaaa atccaccaaa aaaagatgat taccattttg aagtgttcaa ctttgttccc
      181 tgtagtatat gtggcaacaa tcaactctgc aaatccattt gcaaaacaat accaagcaat
      241 aaaccaaaga aaaaaccaac tacaaaaccc acaaacaaac cacctaccaa aaccacaaac
      301 aaaagagacc ccaaaacact agccaaaaca ccgaaaaaag aaaccaccat taacccaaca
      361 aaaaaaccaa cccccaagac tacagaaaga gacaccagca ccccacaatc cactgtgcta
      421 gacataacca catcaaaaca cacagaaaga gacaccagca cctcacaatc cattgtgccc
      481 gacacaacca catcaaaaca cacaacccaa cagcaatctc tccactcaac cacccccgaa
      541 aacacaccca actccacgca aacacccaca gcatccgagc cctccacatc aaattctacc
      601 taa
//
LOCUS   KY328064   603 bp   cRNA   linear   VRL 13-DEC-2016
DEFINITION Human respiratory syncytial virus B isolate TH-CU/C5212/2014
            attachment glycoprotein gene, partial cds.
ACCESSION   KY328064
VERSION   KY328064
KEYWORDS   .
SOURCE   Human respiratory syncytial virus B
  ORGANISM Human respiratory syncytial virus B
            Viruses; ssRNA viruses; ssRNA negative-strand viruses;
            Mononegavirales; Pneumoviridae; Orthopneumovirus.
REFERENCE   1 (bases 1 to 603)
  AUTHORS   Thongpan,I., Mauleekoonphairoj,J., Vichiwattana,P., Korkong,S.,
            Vongpunsawad,S. and Poovorawan,Y.
  TITLE   Molecular Characterization of Respiratory Syncytial Virus in
            Thailand, 2012-2015
  JOURNAL   Unpublished
REFERENCE   2 (bases 1 to 603)
  AUTHORS   Thongpan,I., Mauleekoonphairoj,J., Vichiwattana,P., Korkong,S.,

```
            Vongpunsawad,S. and Poovorawan,Y.
  TITLE     Direct Submission
  JOURNAL   Submitted (13-DEC-2016) Department of Pediatrics, Center of
            Excellence in Clinical Virology, Faculty of Medicine, Chulalongkorn
            University, Bangkok 10330, Thailand
COMMENT     ##Assembly-Data-START##
            Assembly Method       :: DNASTAR-Lasergene v. 6
            Sequencing Technology :: Sanger dideoxy sequencing
            ##Assembly-Data-END##
FEATURES             Location/Qualifiers
     source          1..603
                     /organism="Human respiratory syncytial virus B"
                     /mol_type="viral cRNA"
                     /isolate="TH-CU/C5212/2014"
                     /host="Homo sapiens"
                     /db_xref="taxon:208895"
                     /country="Thailand"
                     /collection_date="01-Nov-2014"
                     /note="group: B"
     CDS             <1..603
                     /note="G protein"
                     /codon_start=1
                     /product="attachment glycoprotein"
                     /protein_id="APY20405"
                     /translation="IHTNSATISPNTKSETHHTTAQTKGTTSTPTQNNKPSTKPRPKN
                     PPKKDDYHFEVFNFVPCSICGNNQLCKSICKTIPSNKPKKKPTTKPTNKPPTKTTNKR
                     DPKTLAKTPKKETTINPTKKPTPKTTERDTSTPQSTVLDITTSKHTERDTSTSQSIVP
                     DTTTSKHTTQQQSLHSTTPENTPNSTQTPTASEPSTSNST"
ORIGIN
        1 atccacacaa actcagccac aatatcaccc aatacaaaat cagaaacaca ccatacaaca
       61 gcacaaacca aaggcacaac ctctactcca acacagaaca acaagccaag cacaaaacca
      121 cgtccaaaaa atccaccaaa aaaagatgat taccattttg aagtgttcaa ctttgttccc
      181 tgtagtatat gtggcaacaa tcaactctgc aaatccattt gcaaaacaat accaagcaat
      241 aaaccaaaga aaaaaccaac tacaaaaccc acaaacaaac cacctaccaa aaccacaaac
      301 aaaagagacc ccaaaacact agccaaaaca ccgaaaaaag aaaccaccat taacccaaca
      361 aaaaaaccaa cccccaagac tacagaaaga gacaccagca ccccacaatc cactgtgcta
      421 gacataacca catcaaaaca cacagaaaga gacaccagca cctcacaatc cattgtgccc
      481 gacacaacca catcaaaaca cacaacccaa cagcaatctc tccactcaac cacccccgaa
      541 aacacaccca actccacgca aacacccaca gcatccgagc cctccacatc aaattctacc
      601 taa
//
LOCUS       KY328065                 603 bp    cRNA    linear   VRL 13-DEC-2016
DEFINITION  Human respiratory syncytial virus B isolate TH-CU/B12236/2015
            attachment glycoprotein gene, partial cds.
ACCESSION   KY328065
VERSION     KY328065
KEYWORDS    .
SOURCE      Human respiratory syncytial virus B
  ORGANISM  Human respiratory syncytial virus B
            Viruses; ssRNA viruses; ssRNA negative-strand viruses;
            Mononegavirales; Pneumoviridae; Orthopneumovirus.
REFERENCE   1  (bases 1 to 603)
  AUTHORS   Thongpan,I., Mauleekoonphairoj,J., Vichiwattana,P., Korkong,S.,
            Vongpunsawad,S. and Poovorawan,Y.
```

```
  TITLE     Molecular Characterization of Respiratory Syncytial Virus in
            Thailand, 2012-2015
  JOURNAL   Unpublished
REFERENCE   2  (bases 1 to 603)
  AUTHORS   Thongpan,I., Mauleekoonphairoj,J., Vichiwattana,P., Korkong,S.,
            Vongpunsawad,S. and Poovorawan,Y.
  TITLE     Direct Submission
  JOURNAL   Submitted (13-DEC-2016) Department of Pediatrics, Center of
            Excellence in Clinical Virology, Faculty of Medicine, Chulalongkorn
            University, Bangkok 10330, Thailand
COMMENT     ##Assembly-Data-START##
            Assembly Method       :: DNASTAR-Lasergene v. 6
            Sequencing Technology :: Sanger dideoxy sequencing
            ##Assembly-Data-END##
FEATURES             Location/Qualifiers
     source          1..603
                     /organism="Human respiratory syncytial virus B"
                     /mol_type="viral cRNA"
                     /isolate="TH-CU/B12236/2015"
                     /host="Homo sapiens"
                     /db_xref="taxon:208895"
                     /country="Thailand"
                     /collection_date="12-Jul-2015"
                     /note="group: B"
     CDS             <1..603
                     /note="G protein"
                     /codon_start=1
                     /product="attachment glycoprotein"
                     /protein_id="APY20406"
                     /translation="IHTNSATISPNTKSETHHTTAQTKGTTSTPTQNNKPSTKPRPKN
                     PPKKDDYHFEVFNFVPCSICGNNQLCKSICKTIPSNKPKKKPTTKPTNKPPTKTTNKR
                     DPKTLAKTPKKETTINPTKKPTPKTTERDSSTPQSTVLDITTSKHTERDTSTSQSIAL
                     DTTTSKHTTQQQSLYSTTPENTPNSTQTPTASEPSTSNST"
ORIGIN
        1 atccacacaa actcagccac aatatcaccc aatacaaaat cagaaacaca ccatacaaca
       61 gcacaaacca aaggcacaac ctctactcca acacagaaca acaagccaag cacaaaacca
      121 cgtccaaaaa atccaccaaa aaaagatgat taccattttg aagtgttcaa ctttgttccc
      181 tgtagtatat gtggcaacaa tcaactttgc aaatccattt gcaaaacaat accaagcaat
      241 aaaccaaaga aaaaaccaac tacaaaaccc acaaacaaac cacctaccaa aaccacaaac
      301 aaaagggacc ccaaaacact agccaaaaca ccgaaaaaag aaaccaccat taacccaaca
      361 aaaaaaccaa cccccaagac tacagaaaga gactccagca ccccacaatc cactgtgctc
      421 gacataacca catcaaaaca cacagaaaga gacaccagca cctcacaatc cattgcgctt
      481 gacacaacca catcaaaaca cacaacccaa cagcaatctc tctactcaac cacccccgaa
      541 aacacaccca actccacgca aacacccaca gcatccgagc cctccacatc aaattctacc
      601 taa
//
LOCUS       KY328066                 603 bp    cRNA    linear   VRL 13-DEC-2016
DEFINITION  Human respiratory syncytial virus B isolate TH-CU/B12411/2015
            attachment glycoprotein gene, partial cds.
ACCESSION   KY328066
VERSION     KY328066
KEYWORDS    .
SOURCE      Human respiratory syncytial virus B
  ORGANISM  Human respiratory syncytial virus B
```

```
            Viruses; ssRNA viruses; ssRNA negative-strand viruses;
            Mononegavirales; Pneumoviridae; Orthopneumovirus.
REFERENCE   1  (bases 1 to 603)
  AUTHORS   Thongpan,I., Mauleekoonphairoj,J., Vichiwattana,P., Korkong,S.,
            Vongpunsawad,S. and Poovorawan,Y.
  TITLE     Molecular Characterization of Respiratory Syncytial Virus in
            Thailand, 2012-2015
  JOURNAL   Unpublished
REFERENCE   2  (bases 1 to 603)
  AUTHORS   Thongpan,I., Mauleekoonphairoj,J., Vichiwattana,P., Korkong,S.,
            Vongpunsawad,S. and Poovorawan,Y.
  TITLE     Direct Submission
  JOURNAL   Submitted (13-DEC-2016) Department of Pediatrics, Center of
            Excellence in Clinical Virology, Faculty of Medicine, Chulalongkorn
            University, Bangkok 10330, Thailand
COMMENT     ##Assembly-Data-START##
            Assembly Method       :: DNASTAR-Lasergene v. 6
            Sequencing Technology :: Sanger dideoxy sequencing
            ##Assembly-Data-END##
FEATURES             Location/Qualifiers
     source          1..603
                     /organism="Human respiratory syncytial virus B"
                     /mol_type="viral cRNA"
                     /isolate="TH-CU/B12411/2015"
                     /host="Homo sapiens"
                     /db_xref="taxon:208895"
                     /country="Thailand"
                     /collection_date="23-Jul-2015"
                     /note="group: B"
     CDS             <1..603
                     /note="G protein"
                     /codon_start=1
                     /product="attachment glycoprotein"
                     /protein_id="APY20407"
                     /translation="IHTNSATISPNTKSETHHTTAQTKGTTSTPTQNNKPSTKPRPKN
                     PPKKDDYHFEVFNFVPCSICGNNQLCKSICKTIPSNKPKKKPTTKPTNKPPTKTTNKR
                     DPKTLAKTPKKETTINPTKKPTPKTTERDSSTPQSTVLDITTSKHTERDTSTSQSIAL
                     DTTTSKHTTQQQSLYSTTPENTPNSTQTPTASEPSTSNST"
ORIGIN
        1 atccacacaa actcagccac aatatcaccc aatacaaaat cagaaacaca ccatacaaca
       61 gcacaaacca aaggcacaac ctctactcca acacagaaca acaagccaag cacaaaacca
      121 cgtccaaaaa atccaccaaa aaaagatgat taccattttg aagtgttcaa ctttgttccc
      181 tgtagtatat gtggcaacaa tcaactttgc aaatccattt gcaaaacaat accaagcaat
      241 aaaccaaaga aaaaaccaac tacaaaaccc acaaacaaac cacctaccaa aaccacaaac
      301 aaaagggacc ccaaaacact agccaaaaca ccgaaaaaag aaaccaccat taacccaaca
      361 aaaaaaccaa cccccaagac tacagaaaga gactccagca ccccacaatc cactgtgctc
      421 gacataacca catcaaaaca cacagaaaga gacaccagca cctcacaatc cattgcgctt
      481 gacacaacca catcaaaaca cacaacccaa cagcaatctc tctactcaac cacccccgaa
      541 aacacaccca actccacgca aacacccaca gcatccgagc cctccacatc aaattctacc
      601 taa
//
LOCUS       KY328067                 603 bp    cRNA    linear   VRL 13-DEC-2016
DEFINITION  Human respiratory syncytial virus B isolate TH-CU/B12543/2015
            attachment glycoprotein gene, partial cds.
```

```
ACCESSION   KY328067
VERSION     KY328067
KEYWORDS    .
SOURCE      Human respiratory syncytial virus B
  ORGANISM  Human respiratory syncytial virus B
            Viruses; ssRNA viruses; ssRNA negative-strand viruses;
            Mononegavirales; Pneumoviridae; Orthopneumovirus.
REFERENCE   1  (bases 1 to 603)
  AUTHORS   Thongpan,I., Mauleekoonphairoj,J., Vichiwattana,P., Korkong,S.,
            Vongpunsawad,S. and Poovorawan,Y.
  TITLE     Molecular Characterization of Respiratory Syncytial Virus in
            Thailand, 2012-2015
  JOURNAL   Unpublished
REFERENCE   2  (bases 1 to 603)
  AUTHORS   Thongpan,I., Mauleekoonphairoj,J., Vichiwattana,P., Korkong,S.,
            Vongpunsawad,S. and Poovorawan,Y.
  TITLE     Direct Submission
  JOURNAL   Submitted (13-DEC-2016) Department of Pediatrics, Center of
            Excellence in Clinical Virology, Faculty of Medicine, Chulalongkorn
            University, Bangkok 10330, Thailand
COMMENT     ##Assembly-Data-START##
            Assembly Method       :: DNASTAR-Lasergene v. 6
            Sequencing Technology :: Sanger dideoxy sequencing
            ##Assembly-Data-END##
FEATURES             Location/Qualifiers
     source          1..603
                     /organism="Human respiratory syncytial virus B"
                     /mol_type="viral cRNA"
                     /isolate="TH-CU/B12543/2015"
                     /host="Homo sapiens"
                     /db_xref="taxon:208895"
                     /country="Thailand"
                     /collection_date="11-Aug-2015"
                     /note="group: B"
     CDS             <1..603
                     /note="G protein"
                     /codon_start=1
                     /product="attachment glycoprotein"
                     /protein_id="APY20408"
                     /translation="IHTNSATISPNTKSETHHTTAQTKGTTSTPTQNNKPSTKPRPKN
                     PPKKDDYHFEVFNFVPCSICGNNQLCKSICKTIPSNKPKKKPTTKPTNKPPTKTTNKR
                     DPKTLAKTPKKETTINPTKKPTPKTTERDSSTPQSTVLDITTSKHTERDTSTSQSIAL
                     DTTTSKHTTQQQSLYSTTPENTPNSTQTPTASEPSTSNST"
ORIGIN
        1 atccacacaa actcagccac aatatcaccc aatacaaaat cagaaacaca ccatacaaca
       61 gcacaaacca aaggcacaac ctctactcca acacagaaca acaagccaag cacaaaacca
      121 cgtccaaaaa atccaccaaa aaaagatgat taccattttg aagtgttcaa ctttgttccc
      181 tgtagtatat gtggcaacaa tcaactttgc aaatccattt gcaaaacaat accaagcaat
      241 aaaccaaaga aaaaaccaac tacaaaaccc acaaacaaac cacctaccaa aaccacaaac
      301 aaaagggacc ccaaaacact agccaaaaca ccgaaaaaag aaaccaccat taacccaaca
      361 aaaaaaccaa cccccaagac tacagaaaga gactccagca ccccacaatc cactgtgctc
      421 gacataacca catcaaaaca cacagaaaga gacaccagca cctcacaatc cattgcgctt
      481 gacacaacca catcaaaaca cacaacccaa cagcaatctc tctactcaac cacccccgaa
      541 aacacaccca actccacgca aacacccaca gcatccgagc cctccacatc aaattctacc
```

601 taa
//
LOCUS   KY328068   603 bp   cRNA   linear   VRL 13-DEC-2016
DEFINITION Human respiratory syncytial virus B isolate TH-CU/B12474/2015
            attachment glycoprotein gene, partial cds.
ACCESSION   KY328068
VERSION   KY328068
KEYWORDS   .
SOURCE   Human respiratory syncytial virus B
  ORGANISM Human respiratory syncytial virus B
            Viruses; ssRNA viruses; ssRNA negative-strand viruses;
            Mononegavirales; Pneumoviridae; Orthopneumovirus.
REFERENCE   1 (bases 1 to 603)
  AUTHORS   Thongpan,I., Mauleekoonphairoj,J., Vichiwattana,P., Korkong,S.,
            Vongpunsawad,S. and Poovorawan,Y.
  TITLE   Molecular Characterization of Respiratory Syncytial Virus in
            Thailand, 2012-2015
  JOURNAL   Unpublished
REFERENCE   2 (bases 1 to 603)
  AUTHORS   Thongpan,I., Mauleekoonphairoj,J., Vichiwattana,P., Korkong,S.,
            Vongpunsawad,S. and Poovorawan,Y.
  TITLE   Direct Submission
  JOURNAL   Submitted (13-DEC-2016) Department of Pediatrics, Center of
            Excellence in Clinical Virology, Faculty of Medicine, Chulalongkorn
            University, Bangkok 10330, Thailand
COMMENT   ##Assembly-Data-START##
            Assembly Method   :: DNASTAR-Lasergene v. 6
            Sequencing Technology :: Sanger dideoxy sequencing
            ##Assembly-Data-END##
FEATURES   Location/Qualifiers
     source   1..603
                     /organism="Human respiratory syncytial virus B"
                     /mol_type="viral cRNA"
                     /isolate="TH-CU/B12474/2015"
                     /host="Homo sapiens"
                     /db_xref="taxon:208895"
                     /country="Thailand"
                     /collection_date="04-Aug-2015"
                     /note="group: B"
     CDS   <1..603
                     /note="G protein"
                     /codon_start=1
                     /product="attachment glycoprotein"
                     /protein_id="APY20409"
                     /translation="IHTNSATISPNTKSETHHTTAQTKGTTSTPTQNNKPSTKPRPKN
                     PPKKDDYHFEVFNFVPCSICGNNQLCKSICKTIPSNKPKKKPTTKPTNKPPTKTTNKR
                     DPKTLAKTPKKETTINPTKKPTPKTTERDSSTPQSTLLDITTSKHTERDTSTSQSIAL
                     DTTTSKHTTQQQSLYSTTPENTPNSTQTPTASEPSTSNST"
ORIGIN
        1 atccacacaa actcagccac aatatcaccc aatacaaaat cagaaacgca ccatacaaca
       61 gcacaaacca aaggcacaac ctctactcca acacagaaca acaagccaag cacaaaacca
      121 cgtccaaaaa atccaccaaa aaaagatgat taccattttg aagtgttcaa ctttgttccc
      181 tgtagtatat gtggcaacaa tcaactttgc aaatccattt gcaaaacaat accaagcaat
      241 aaaccaaaga aaaaaccaac tacaaaaccc acaaacaaac cacctaccaa aaccacaaac

```
      301 aaaagggacc ccaaaacact agccaaaaca ccgaaaaaag aaaccaccat taacccaaca
      361 aaaaaaccaa cccccaagac tacagaaaga gactccagca ccccacaatc cactctgctc
      421 gacataacca catcaaaaca cacagaaaga gacaccagca cctcacaatc cattgcgctt
      481 gacacaacca catcaaaaca cacaacccaa cagcaatctc tctactcaac cacccccgaa
      541 aacacaccca actccacgca aacacccaca gcatccgagc cctccacatc aaattctacc
      601 taa
//
LOCUS       KY328069                 603 bp    cRNA    linear   VRL 13-DEC-2016
DEFINITION  Human respiratory syncytial virus B isolate TH-CU/B12487/2015
            attachment glycoprotein gene, partial cds.
ACCESSION   KY328069
VERSION     KY328069
KEYWORDS    .
SOURCE      Human respiratory syncytial virus B
  ORGANISM  Human respiratory syncytial virus B
            Viruses; ssRNA viruses; ssRNA negative-strand viruses;
            Mononegavirales; Pneumoviridae; Orthopneumovirus.
REFERENCE   1  (bases 1 to 603)
  AUTHORS   Thongpan,I., Mauleekoonphairoj,J., Vichiwattana,P., Korkong,S.,
            Vongpunsawad,S. and Poovorawan,Y.
  TITLE     Molecular Characterization of Respiratory Syncytial Virus in
            Thailand, 2012-2015
  JOURNAL   Unpublished
REFERENCE   2  (bases 1 to 603)
  AUTHORS   Thongpan,I., Mauleekoonphairoj,J., Vichiwattana,P., Korkong,S.,
            Vongpunsawad,S. and Poovorawan,Y.
  TITLE     Direct Submission
  JOURNAL   Submitted (13-DEC-2016) Department of Pediatrics, Center of
            Excellence in Clinical Virology, Faculty of Medicine, Chulalongkorn
            University, Bangkok 10330, Thailand
COMMENT     ##Assembly-Data-START##
            Assembly Method       :: DNASTAR-Lasergene v. 6
            Sequencing Technology :: Sanger dideoxy sequencing
            ##Assembly-Data-END##
FEATURES             Location/Qualifiers
     source          1..603
                     /organism="Human respiratory syncytial virus B"
                     /mol_type="viral cRNA"
                     /isolate="TH-CU/B12487/2015"
                     /host="Homo sapiens"
                     /db_xref="taxon:208895"
                     /country="Thailand"
                     /collection_date="05-Aug-2015"
                     /note="group: B"
     CDS             <1..603
                     /note="G protein"
                     /codon_start=1
                     /product="attachment glycoprotein"
                     /protein_id="APY20410"
                     /translation="IHTNSATISPNTKSETHHTTAQTKGTTSTPTQNNKPSTKPRPKN
                     PPKKDDYHFEVFNFVPCSICGNNQLCKSICKTIPSNKPKKKPTTKPTNKPPTKTTNKR
                     DPKTLAKTPKKETTINPTKKPTPKTTERDSSTPQSTLLDITTSKHTERDTSTSQSIAL
                     DTTTSKHTTQQQSLYSTTPENTPNSTQTPTASEPSTSNST"
ORIGIN
```

```
        1 atccacacaa actcagccac aatatcaccc aatacaaaat cagaaacgca ccatacaaca
       61 gcacaaacca aaggcacaac ctctactcca acacagaaca acaagccaag cacaaaacca
      121 cgtccaaaaa atccaccaaa aaaagatgat taccattttg aagtgttcaa ctttgttccc
      181 tgtagtatat gtggcaacaa tcaactttgc aaatccattt gcaaaacaat accaagcaat
      241 aaaccaaaga aaaaaccaac tacaaaaccc acaaacaaac cacctaccaa aaccacaaac
      301 aaaagggacc ccaaaacact agccaaaaca ccgaaaaaag aaaccaccat taacccaaca
      361 aaaaaaccaa cccccaagac tacagaaaga gactccagca ccccacaatc cactctgctc
      421 gacataacca catcaaaaca cacagaaaga gacaccagca cctcacaatc cattgcgctt
      481 gacacaacca catcaaaaca cacaacccaa cagcaatctc tctactcaac cacccccgaa
      541 aacacaccca actccacgca aacacccaca gcatccgagc cctccacatc aaattctacc
      601 taa
//
LOCUS       KY328070                 603 bp    cRNA    linear   VRL 13-DEC-2016
DEFINITION  Human respiratory syncytial virus B isolate TH-CU/B12719/2015
            attachment glycoprotein gene, partial cds.
ACCESSION   KY328070
VERSION     KY328070
KEYWORDS    .
SOURCE      Human respiratory syncytial virus B
  ORGANISM  Human respiratory syncytial virus B
            Viruses; ssRNA viruses; ssRNA negative-strand viruses;
            Mononegavirales; Pneumoviridae; Orthopneumovirus.
REFERENCE   1  (bases 1 to 603)
  AUTHORS   Thongpan,I., Mauleekoonphairoj,J., Vichiwattana,P., Korkong,S.,
            Vongpunsawad,S. and Poovorawan,Y.
  TITLE     Molecular Characterization of Respiratory Syncytial Virus in
            Thailand, 2012-2015
  JOURNAL   Unpublished
REFERENCE   2  (bases 1 to 603)
  AUTHORS   Thongpan,I., Mauleekoonphairoj,J., Vichiwattana,P., Korkong,S.,
            Vongpunsawad,S. and Poovorawan,Y.
  TITLE     Direct Submission
  JOURNAL   Submitted (13-DEC-2016) Department of Pediatrics, Center of
            Excellence in Clinical Virology, Faculty of Medicine, Chulalongkorn
            University, Bangkok 10330, Thailand
COMMENT     ##Assembly-Data-START##
            Assembly Method       :: DNASTAR-Lasergene v. 6
            Sequencing Technology :: Sanger dideoxy sequencing
            ##Assembly-Data-END##
FEATURES             Location/Qualifiers
     source          1..603
                     /organism="Human respiratory syncytial virus B"
                     /mol_type="viral cRNA"
                     /isolate="TH-CU/B12719/2015"
                     /host="Homo sapiens"
                     /db_xref="taxon:208895"
                     /country="Thailand"
                     /collection_date="25-Aug-2015"
                     /note="group: B"
     CDS             <1..603
                     /note="G protein"
                     /codon_start=1
                     /product="attachment glycoprotein"
                     /protein_id="APY20411"
```

/translation="IHTNSATISPNTKSETHHTTAQTKGTTSTPTQNNKPSTKPRPKN
PPKKDDYHFEVFNFVPCSICGNNQLCKSICKTIPSNKPKKKPTTKPTNKPPTKTTNKR
DPKTLAKTPKKENTINPTKKPTPKTTERDTSTPQSTVLDITTSKHTERDTSTSQSIAL
DTTTSKHTTQQQSLYSTTPENTPNSTQTPTASEPSTSNST"
ORIGIN
1 atccacacaa actcagccac aatatcaccc aatacaaaat cagaaacaca ccatacaaca
61 gcacaaacca aaggcacaac ctctactcca acacagaaca acaagccaag cacaaaacca
121 cgtccaaaaa atccaccaaa aaaagatgat taccattttg aagtgttcaa ctttgttccc
181 tgtagtatat gtggcaacaa tcaactctgc aaatccattt gcaaaacaat accaagcaat
241 aaaccaaaga aaaaaccaac tacaaaaccc acaaacaaac cacctaccaa aactacaaac
301 aaaagagacc ccaaaacact agccaagaca ccgaaaaaag aaaacaccat taacccaaca
361 aaaaaaccaa cccccaagac tacagaaaga gacaccagca ccccacaatc cactgtgctc
421 gacataacca catcaaaaca cacagaaaga gacaccagca cctcacaatc cattgcgctt
481 gacacaacca catcaaaaca cacaacccaa cagcaatctc tctactcaac cacccccgaa
541 aacacaccca actccacaca aacacccaca gcatccgagc cctccacatc aaattctacc
601 taa
//
LOCUS KY328071 603 bp cRNA linear VRL 13-DEC-2016
DEFINITION Human respiratory syncytial virus B isolate TH-CU/B12619/2015
attachment glycoprotein gene, partial cds.
ACCESSION KY328071
VERSION KY328071
KEYWORDS .
SOURCE Human respiratory syncytial virus B
ORGANISM Human respiratory syncytial virus B
Viruses; ssRNA viruses; ssRNA negative-strand viruses;
Mononegavirales; Pneumoviridae; Orthopneumovirus.
REFERENCE 1 (bases 1 to 603)
AUTHORS Thongpan,I., Mauleekoonphairoj,J., Vichiwattana,P., Korkong,S.,
Vongpunsawad,S. and Poovorawan,Y.
TITLE Molecular Characterization of Respiratory Syncytial Virus in
Thailand, 2012-2015
JOURNAL Unpublished
REFERENCE 2 (bases 1 to 603)
AUTHORS Thongpan,I., Mauleekoonphairoj,J., Vichiwattana,P., Korkong,S.,
Vongpunsawad,S. and Poovorawan,Y.
TITLE Direct Submission
JOURNAL Submitted (13-DEC-2016) Department of Pediatrics, Center of
Excellence in Clinical Virology, Faculty of Medicine, Chulalongkorn
University, Bangkok 10330, Thailand
COMMENT ##Assembly-Data-START##
Assembly Method :: DNASTAR-Lasergene v. 6
Sequencing Technology :: Sanger dideoxy sequencing
##Assembly-Data-END##
FEATURES Location/Qualifiers
source 1..603
/organism="Human respiratory syncytial virus B"
/mol_type="viral cRNA"
/isolate="TH-CU/B12619/2015"
/host="Homo sapiens"
/db_xref="taxon:208895"
/country="Thailand"
/collection_date="13-Aug-2015"
/note="group: B"

CDS <1..603
/note="G protein"
/codon_start=1
/product="attachment glycoprotein"
/protein_id="APY20412"
/translation="IHTNSTTISPNTKSETHHTTAQTKGTTSTPTQNNKPSTKPRPKN
PPKKDDYHFEVFNFVPCSICGNNQLCKSICKTIPSNKPKKKPTTKPTNKPPTKTTNKR
DPKTLAKTPKKENTINPTKKPTPKTTERDTSTPQSTVLDITTSKHTERDTSTSQSIAL
DTTTSKHTTQQQSLYSTTPENTPNSTQTPTASEPSTSNST"

ORIGIN
1 atccacacaa actcaaccac aatatcaccc aatacaaaat cagaaacaca ccatacaaca
61 gcacaaacca aaggcacaac ctctactcca acacagaaca acaagccaag cacaaaacca
121 cgtccaaaaa atccaccaaa aaaagatgat taccattttg aagtgttcaa ctttgttccc
181 tgtagtatat gtggcaacaa tcaactctgc aaatccatct gcaaaacaat accaagcaat
241 aaaccaaaga aaaaaccaac tacaaaaccc acaaacaaac cacctaccaa aactacaaac
301 aaaagagacc ccaaaacact agccaaaaca ccgaaaaaag aaaacaccat taacccaaca
361 aaaaaaccaa cccccaagac tacagaaaga gacaccagca ccccacaatc cactgtgctc
421 gacataacca catcaaaaca cacagaaaga gacaccagca cctcacaatc cattgcgctt
481 gacacaacca catcaaaaca cacaacccaa cagcaatctc tctactcaac cacccccgaa
541 aacacaccca actccacaca aacacccaca gcatccgagc cctccacatc aaattctacc
601 taa

//

LOCUS KY328072 603 bp cRNA linear VRL 13-DEC-2016
DEFINITION Human respiratory syncytial virus B isolate TH-CU/B12626/2015
attachment glycoprotein gene, partial cds.
ACCESSION KY328072
VERSION KY328072
KEYWORDS .
SOURCE Human respiratory syncytial virus B
ORGANISM Human respiratory syncytial virus B
Viruses; ssRNA viruses; ssRNA negative-strand viruses;
Mononegavirales; Pneumoviridae; Orthopneumovirus.
REFERENCE 1 (bases 1 to 603)
AUTHORS Thongpan,I., Mauleekoonphairoj,J., Vichiwattana,P., Korkong,S.,
Vongpunsawad,S. and Poovorawan,Y.
TITLE Molecular Characterization of Respiratory Syncytial Virus in
Thailand, 2012-2015
JOURNAL Unpublished
REFERENCE 2 (bases 1 to 603)
AUTHORS Thongpan,I., Mauleekoonphairoj,J., Vichiwattana,P., Korkong,S.,
Vongpunsawad,S. and Poovorawan,Y.
TITLE Direct Submission
JOURNAL Submitted (13-DEC-2016) Department of Pediatrics, Center of
Excellence in Clinical Virology, Faculty of Medicine, Chulalongkorn
University, Bangkok 10330, Thailand
COMMENT ##Assembly-Data-START##
Assembly Method :: DNASTAR-Lasergene v. 6
Sequencing Technology :: Sanger dideoxy sequencing
##Assembly-Data-END##
FEATURES Location/Qualifiers
source 1..603
/organism="Human respiratory syncytial virus B"
/mol_type="viral cRNA"
/isolate="TH-CU/B12626/2015"

```
                     /host="Homo sapiens"
                     /db_xref="taxon:208895"
                     /country="Thailand"
                     /collection_date="14-Aug-2015"
                     /note="group: B"
     CDS             <1..603
                     /note="G protein"
                     /codon_start=1
                     /product="attachment glycoprotein"
                     /protein_id="APY20413"
                     /translation="IHTNSTTISPNTKSETHHTTAQTKGTTSTPTQNNKPSTKPRPKN
                     PPKKDDYHFEVFNFVPCSICGNNQLCKSICKTIPSNKPKKKPTTKPTNKPPTKTTNKR
                     DPKTLAKTPKKENTINPTKKPTPKTTERDTSTPQSTVLDITTSKHTERDTSTSQSIAL
                     DTTTSKHTTQQQSLYSTTPENTPNSTQTPTASEPSTSNST"
ORIGIN      
        1 atccacacaa actcaaccac aatatcaccc aatacaaaat cagaaacaca ccatacaaca
       61 gcacaaacca aaggcacaac ctctactcca acacagaaca acaagccaag cacaaaacca
      121 cgtccaaaaa atccaccaaa aaaagatgat taccattttg aagtgttcaa ctttgttccc
      181 tgtagtatat gtggcaacaa tcaactctgc aaatccatct gcaaaacaat accaagcaat
      241 aaaccaaaga aaaaaccaac tacaaaaccc acaaacaaac cacctaccaa aactacaaac
      301 aaaagagacc ccaaaacact agccaaaaca ccgaaaaaag aaaacaccat taacccaaca
      361 aaaaaaccaa cccccaagac tacagaaaga gacaccagca ccccacaatc cactgtgctc
      421 gacataacca catcaaaaca cacagaaaga gacaccagca cctcacaatc cattgcgctt
      481 gacacaacca catcaaaaca cacaacccaa cagcaatctc tctactcaac cacccccgaa
      541 aacacaccca actccacaca aacacccaca gcatccgagc cctccacatc aaattctacc
      601 taa
//
LOCUS       KY328073                 624 bp    cRNA    linear   VRL 13-DEC-2016
DEFINITION  Human respiratory syncytial virus B isolate TH-CU/C6153/2015
            attachment glycoprotein gene, partial cds.
ACCESSION   KY328073
VERSION     KY328073
KEYWORDS    .
SOURCE      Human respiratory syncytial virus B
  ORGANISM  Human respiratory syncytial virus B
            Viruses; ssRNA viruses; ssRNA negative-strand viruses;
            Mononegavirales; Pneumoviridae; Orthopneumovirus.
REFERENCE   1  (bases 1 to 624)
  AUTHORS   Thongpan,I., Mauleekoonphairoj,J., Vichiwattana,P., Korkong,S.,
            Vongpunsawad,S. and Poovorawan,Y.
  TITLE     Molecular Characterization of Respiratory Syncytial Virus in
            Thailand, 2012-2015
  JOURNAL   Unpublished
REFERENCE   2  (bases 1 to 624)
  AUTHORS   Thongpan,I., Mauleekoonphairoj,J., Vichiwattana,P., Korkong,S.,
            Vongpunsawad,S. and Poovorawan,Y.
  TITLE     Direct Submission
  JOURNAL   Submitted (13-DEC-2016) Department of Pediatrics, Center of
            Excellence in Clinical Virology, Faculty of Medicine, Chulalongkorn
            University, Bangkok 10330, Thailand
COMMENT     ##Assembly-Data-START##
            Assembly Method       :: DNASTAR-Lasergene v. 6
            Sequencing Technology :: Sanger dideoxy sequencing
            ##Assembly-Data-END##
```

```
FEATURES             Location/Qualifiers
     source          1..624
                     /organism="Human respiratory syncytial virus B"
                     /mol_type="viral cRNA"
                     /isolate="TH-CU/C6153/2015"
                     /host="Homo sapiens"
                     /db_xref="taxon:208895"
                     /country="Thailand"
                     /collection_date="08-Oct-2015"
                     /note="group: B"
     CDS             <1..624
                     /note="G protein"
                     /codon_start=1
                     /product="attachment glycoprotein"
                     /protein_id="APY20414"
                     /translation="IHTNSATISPNTKSETHHTTAQTKGTTSTPTQNNKPSTKPRPKN
                     PPKKDDYHFEVFNFVPCSICGNNQLCKSICKTIPSNKPKKKPTTKPTNKPPTKTTNKR
                     DPKTLAKTPKKENTINPTKKPTPKTTERDTSTPQSTVLDITTSKHTERDTSTSQSIAL
                     DTTTSKHTTQQQSLYSTTPENTPNSTQTPTASEPSTSNSTQRLQSYA"
ORIGIN
        1 atccacacaa actcagccac aatatcaccc aatacaaaat cagaaacaca ccatacaaca
       61 gcacaaacca aaggcacaac ctctactcca acacagaaca acaagccaag cacaaaacca
      121 cgtccaaaaa atccaccaaa aaaagatgat taccattttg aagtgttcaa ctttgttccc
      181 tgtagtatat gtggcaacaa tcaactctgc aaatccattt gcaaaacaat accaagcaat
      241 aaaccaaaga aaaaaccaac tacaaaaccc acaaacaaac cacctaccaa aaccacaaac
      301 aaaagagacc ccaaaacact agccaaaaca ccgaaaaaag aaaacaccat taacccaaca
      361 aaaaaaccaa cccccaagac tacagaaaga gacaccagca ccccacaatc cactgtgctc
      421 gacataacca catcaaaaca cacagaaaga gacaccagca cctcacaatc cattgcactt
      481 gacacaacca catcaaaaca cacaacccaa cagcaatctc tctactcaac cacccccgaa
      541 aacacaccca actccacaca aacacccaca gcatccgagc cctccacatc aaactctacc
      601 caaagactcc agtcatatgc ctag
//
LOCUS       KY328074                 624 bp    cRNA    linear   VRL 13-DEC-2016
DEFINITION  Human respiratory syncytial virus B isolate TH-CU/C6262/2015
            attachment glycoprotein gene, partial cds.
ACCESSION   KY328074
VERSION     KY328074
KEYWORDS    .
SOURCE      Human respiratory syncytial virus B
  ORGANISM  Human respiratory syncytial virus B
            Viruses; ssRNA viruses; ssRNA negative-strand viruses;
            Mononegavirales; Pneumoviridae; Orthopneumovirus.
REFERENCE   1  (bases 1 to 624)
  AUTHORS   Thongpan,I., Mauleekoonphairoj,J., Vichiwattana,P., Korkong,S.,
            Vongpunsawad,S. and Poovorawan,Y.
  TITLE     Molecular Characterization of Respiratory Syncytial Virus in
            Thailand, 2012-2015
  JOURNAL   Unpublished
REFERENCE   2  (bases 1 to 624)
  AUTHORS   Thongpan,I., Mauleekoonphairoj,J., Vichiwattana,P., Korkong,S.,
            Vongpunsawad,S. and Poovorawan,Y.
  TITLE     Direct Submission
  JOURNAL   Submitted (13-DEC-2016) Department of Pediatrics, Center of
            Excellence in Clinical Virology, Faculty of Medicine, Chulalongkorn
```

University, Bangkok 10330, Thailand
COMMENT   ##Assembly-Data-START##
Assembly Method   :: DNASTAR-Lasergene v. 6
Sequencing Technology :: Sanger dideoxy sequencing
##Assembly-Data-END##
FEATURES   Location/Qualifiers
source   1..624
/organism="Human respiratory syncytial virus B"
/mol_type="viral cRNA"
/isolate="TH-CU/C6262/2015"
/host="Homo sapiens"
/db_xref="taxon:208895"
/country="Thailand"
/collection_date="19-Nov-2015"
/note="group: B"
CDS   <1..624
/note="G protein"
/codon_start=1
/product="attachment glycoprotein"
/protein_id="APY20415"
/translation="IHTNSATISPNTKSETHHTTAQTKGTTSTPTQNNKPSTKPRPKN
PPKKDDYHFEVFNFVPCSICGNNQLCKSICKTIPSNKPKKKPTTKPTNKPPTKTTNKR
DPKTLAKTPKKENTINPTKKPTPKTTERDTSTPQSTVLDITTSKHTERDTSTSQSIAL
DTTTSKHTTQQQSLYSTTPENTPNSTQTPTASEPSTSNSTQRLQSYA"
ORIGIN
1 atccacacaa actcagccac aatatcaccc aatacaaaat cagaaacaca ccatacaaca
61 gcacaaacca aaggcacaac ctctactcca acacagaaca acaagccaag cacaaaacca
121 cgtccaaaaa atccaccaaa aaaagatgat taccattttg aagtgttcaa ctttgttccc
181 tgtagtatat gtggcaacaa tcaactctgc aaatccattt gcaaaacaat accaagcaat
241 aaaccaaaga aaaaaccaac tacaaaaccc acaaacaaac cacctaccaa aaccacaaac
301 aaaagagacc ccaaaacact agccaaaaca ccgaaaaaag aaaacaccat taacccaaca
361 aaaaaaccaa cccccaagac tacagaaaga gacaccagca ccccacaatc cactgtgctc
421 gacataacca catcaaaaca cacagaaaga gacaccagca cctcacaatc cattgcactt
481 gacacaacca catcaaaaca cacaacccaa cagcaatctc tctactcaac cacccccgaa
541 aacacaccca actccacaca aacacccaca gcatccgagc cctccacatc aaactctacc
601 caaagactcc agtcatatgc ctag
//
LOCUS   KY328075   624 bp   cRNA   linear   VRL 13-DEC-2016
DEFINITION Human respiratory syncytial virus B isolate TH-CU/B12557/2015
attachment glycoprotein gene, partial cds.
ACCESSION   KY328075
VERSION   KY328075
KEYWORDS   .
SOURCE   Human respiratory syncytial virus B
ORGANISM Human respiratory syncytial virus B
Viruses; ssRNA viruses; ssRNA negative-strand viruses;
Mononegavirales; Pneumoviridae; Orthopneumovirus.
REFERENCE   1 (bases 1 to 624)
AUTHORS   Thongpan,I., Mauleekoonphairoj,J., Vichiwattana,P., Korkong,S.,
Vongpunsawad,S. and Poovorawan,Y.
TITLE   Molecular Characterization of Respiratory Syncytial Virus in
Thailand, 2012-2015
JOURNAL   Unpublished
REFERENCE   2 (bases 1 to 624)

```
  AUTHORS   Thongpan,I., Mauleekoonphairoj,J., Vichiwattana,P., Korkong,S.,
            Vongpunsawad,S. and Poovorawan,Y.
  TITLE     Direct Submission
  JOURNAL   Submitted (13-DEC-2016) Department of Pediatrics, Center of
            Excellence in Clinical Virology, Faculty of Medicine, Chulalongkorn
            University, Bangkok 10330, Thailand
COMMENT     ##Assembly-Data-START##
            Assembly Method       :: DNASTAR-Lasergene v. 6
            Sequencing Technology :: Sanger dideoxy sequencing
            ##Assembly-Data-END##
FEATURES             Location/Qualifiers
     source          1..624
                     /organism="Human respiratory syncytial virus B"
                     /mol_type="viral cRNA"
                     /isolate="TH-CU/B12557/2015"
                     /host="Homo sapiens"
                     /db_xref="taxon:208895"
                     /country="Thailand"
                     /collection_date="16-Aug-2015"
                     /note="group: B"
     CDS             <1..624
                     /note="G protein"
                     /codon_start=1
                     /product="attachment glycoprotein"
                     /protein_id="APY20416"
                     /translation="IHTNSATISPNTKSETHHTTAQTKGTTSTPTQNNKPSTKPRPKN
                     PPKKDDYHFEVFNFVPCSICGNNQLCKSICKTIPSNKPKKKPTTKPTNKPPTKTTNKR
                     DPKTLAKTPKKENTINPTKKPTPKTTERDTSTPQSTVLDITTSKHTERDTSTPQSIAL
                     DTTTSKHTTQQQSLHSTTPENTPNSTQTPTASEPSTSNSTQRLQSYA"
ORIGIN
        1 atccacacaa actcagccac aatatcaccc aatacaaaat cagaaacaca ccatacaaca
       61 gcacaaacca aaggcacaac ctctactcca acacagaaca acaagccaag cacaaaacca
      121 cgtccaaaaa atccaccaaa aaaagatgat taccattttg aagtgttcaa ctttgttccc
      181 tgtagtatat gtggcaacaa tcaactctgc aaatccattt gcaaaacaat accaagcaat
      241 aaaccaaaga aaaaaccaac tacaaaaccc acaaacaaac cacctaccaa aaccacaaac
      301 aaaagagacc ccaaaacact agccaaaaca ccgaaaaaag aaaacaccat taacccaaca
      361 aaaaaaccaa cccccaagac tacagaaaga gacaccagca ccccacaatc cactgtgctc
      421 gacataacca catcaaaaca cacagaaaga gacaccagca ccccacaatc cattgcgctt
      481 gacacaacca catcaaaaca cacaacccaa cagcaatctc tccactcaac cacccccgaa
      541 aacacaccca actccacaca aacacccaca gcatccgagc cctccacatc aaattccacc
      601 caaagactcc agtcatatgc ttag
//
LOCUS       KY328076                 624 bp    cRNA    linear   VRL 13-DEC-2016
DEFINITION  Human respiratory syncytial virus B isolate TH-CU/B12728/2015
            attachment glycoprotein gene, partial cds.
ACCESSION   KY328076
VERSION     KY328076
KEYWORDS    .
SOURCE      Human respiratory syncytial virus B
  ORGANISM  Human respiratory syncytial virus B
            Viruses; ssRNA viruses; ssRNA negative-strand viruses;
            Mononegavirales; Pneumoviridae; Orthopneumovirus.
REFERENCE   1  (bases 1 to 624)
  AUTHORS   Thongpan,I., Mauleekoonphairoj,J., Vichiwattana,P., Korkong,S.,
```

Vongpunsawad,S. and Poovorawan,Y.
TITLE   Molecular Characterization of Respiratory Syncytial Virus in
Thailand, 2012-2015
JOURNAL   Unpublished
REFERENCE   2 (bases 1 to 624)
AUTHORS   Thongpan,I., Mauleekoonphairoj,J., Vichiwattana,P., Korkong,S.,
Vongpunsawad,S. and Poovorawan,Y.
TITLE   Direct Submission
JOURNAL   Submitted (13-DEC-2016) Department of Pediatrics, Center of
Excellence in Clinical Virology, Faculty of Medicine, Chulalongkorn
University, Bangkok 10330, Thailand
COMMENT   ##Assembly-Data-START##
Assembly Method   :: DNASTAR-Lasergene v. 6
Sequencing Technology :: Sanger dideoxy sequencing
##Assembly-Data-END##
FEATURES   Location/Qualifiers
source   1..624
/organism="Human respiratory syncytial virus B"
/mol_type="viral cRNA"
/isolate="TH-CU/B12728/2015"
/host="Homo sapiens"
/db_xref="taxon:208895"
/country="Thailand"
/collection_date="26-Aug-2015"
/note="group: B"
CDS   <1..624
/note="G protein"
/codon_start=1
/product="attachment glycoprotein"
/protein_id="APY20417"
/translation="IHTNSATISPNTKSETHHTTAQTKGTTSTPTQNNKPSTKPRPKN
PPKKDDYHFEVFNFVPCSICGNNQLCKSICKTIPSNKPKKKPTTKPTNKPPTKTTNKR
DPKTLAKTPKKENTINPTKKPTPKTTERDTSTPQSTVLDITTSKHTERDTSTPQSIAL
DTTTSKHTTQQQSLHSTTPENTPNSTQTPTASEPSTSNSTQRLQSYA"
ORIGIN
1 atccacacaa actcagccac aatatcaccc aatacaaaat cagaaacaca ccatacaaca
61 gcacaaacca aaggcacaac ctctactcca acacagaaca acaagccaag cacaaaacca
121 cgtccaaaaa atccaccaaa aaaagatgat taccattttg aagtgttcaa ctttgttccc
181 tgtagtatat gtggcaacaa tcaactctgc aaatccattt gcaaaacaat accaagcaat
241 aaaccaaaga aaaaaccaac tacaaaaccc acaaacaaac cacctaccaa aaccacaaac
301 aaaagagacc ccaaaacact agccaaaaca ccgaaaaaag aaaacaccat taacccaaca
361 aaaaaaccaa cccccaagac tacagaaaga gacaccagca ccccacaatc cactgtgctc
421 gacataacca catcaaaaca cacagaaaga gacaccagca ccccacaatc cattgcgctt
481 gacacaacca catcaaaaca cacaacccaa cagcaatctc tccactcaac cacccccgaa
541 aacacaccca actccacaca aacacccaca gcatccgagc cctccacatc aaattccacc
601 caaagactcc agtcatatgc ttag
//
LOCUS   KY328077   603 bp   cRNA   linear   VRL 13-DEC-2016
DEFINITION Human respiratory syncytial virus B isolate TH-CU/B12360/2015
attachment glycoprotein gene, partial cds.
ACCESSION   KY328077
VERSION   KY328077
KEYWORDS   .
SOURCE   Human respiratory syncytial virus B

```
  ORGANISM  Human respiratory syncytial virus B
            Viruses; ssRNA viruses; ssRNA negative-strand viruses;
            Mononegavirales; Pneumoviridae; Orthopneumovirus.
REFERENCE   1  (bases 1 to 603)
  AUTHORS   Thongpan,I., Mauleekoonphairoj,J., Vichiwattana,P., Korkong,S.,
            Vongpunsawad,S. and Poovorawan,Y.
  TITLE     Molecular Characterization of Respiratory Syncytial Virus in
            Thailand, 2012-2015
  JOURNAL   Unpublished
REFERENCE   2  (bases 1 to 603)
  AUTHORS   Thongpan,I., Mauleekoonphairoj,J., Vichiwattana,P., Korkong,S.,
            Vongpunsawad,S. and Poovorawan,Y.
  TITLE     Direct Submission
  JOURNAL   Submitted (13-DEC-2016) Department of Pediatrics, Center of
            Excellence in Clinical Virology, Faculty of Medicine, Chulalongkorn
            University, Bangkok 10330, Thailand
COMMENT     ##Assembly-Data-START##
            Assembly Method       :: DNASTAR-Lasergene v. 6
            Sequencing Technology :: Sanger dideoxy sequencing
            ##Assembly-Data-END##
FEATURES             Location/Qualifiers
     source          1..603
                     /organism="Human respiratory syncytial virus B"
                     /mol_type="viral cRNA"
                     /isolate="TH-CU/B12360/2015"
                     /host="Homo sapiens"
                     /db_xref="taxon:208895"
                     /country="Thailand"
                     /collection_date="12-Jul-2015"
                     /note="group: B"
     CDS             <1..603
                     /note="G protein"
                     /codon_start=1
                     /product="attachment glycoprotein"
                     /protein_id="APY20418"
                     /translation="IHTNSATISPNTKSETHHTTAQTKSTTSTPTQNNKPSTKPRPKN
                     PPKKDDYHFEVFNFVPCSICGNNQLCKSICKTIPSNKPKKKPTTKPTNKPPTKTTNKR
                     DPKTLAKTPKKETTINPTKKPTPKTTERDTSTPQSTVLDITTSKHTERDTSTSQSIAL
                     DTTTSKHTTQQQSLYSTTPENTPNSTQTPTASEPSTSNST"
ORIGIN
        1 atccacacaa actcagccac aatatcaccc aatacaaaat cagaaacaca ccatacaaca
       61 gcacaaacca aaagcacaac ctctactcca acacagaaca acaagccgag cacaaaacca
      121 cgtccaaaaa atccaccaaa aaaagatgat taccattttg aagtgttcaa ctttgttccc
      181 tgtagtatat gtggcaacaa tcaactctgc aaatccattt gcaaaacaat accaagcaac
      241 aaaccaaaga aaaaaccaac tacaaaaccc acaaacaaac cacctactaa aaccacaaac
      301 aaaagagacc ccaaaacact agccaaaaca ccgaaaaaag aaaccaccat taacccaaca
      361 aaaaaaccaa cccccaagac tacagaaaga gacaccagca ccccacaatc cactgtgctc
      421 gacataacca catcaaaaca cacagaaaga gacaccagca cctcacaatc cattgcgctt
      481 gacacaacca catcaaaaca cacaacccaa cagcaatctc tctactcaac cacccccgaa
      541 aacacaccca actccacaca aacacccaca gcatccgagc cctccacatc aaattctacc
      601 taa
//
LOCUS       KY328078                 603 bp    cRNA    linear   VRL 13-DEC-2016
DEFINITION  Human respiratory syncytial virus B isolate TH-CU/C6207/2015
```

```
            attachment glycoprotein gene, partial cds.
ACCESSION   KY328078
VERSION     KY328078
KEYWORDS    .
SOURCE      Human respiratory syncytial virus B
  ORGANISM  Human respiratory syncytial virus B
            Viruses; ssRNA viruses; ssRNA negative-strand viruses;
            Mononegavirales; Pneumoviridae; Orthopneumovirus.
REFERENCE   1  (bases 1 to 603)
  AUTHORS   Thongpan,I., Mauleekoonphairoj,J., Vichiwattana,P., Korkong,S.,
            Vongpunsawad,S. and Poovorawan,Y.
  TITLE     Molecular Characterization of Respiratory Syncytial Virus in
            Thailand, 2012-2015
  JOURNAL   Unpublished
REFERENCE   2  (bases 1 to 603)
  AUTHORS   Thongpan,I., Mauleekoonphairoj,J., Vichiwattana,P., Korkong,S.,
            Vongpunsawad,S. and Poovorawan,Y.
  TITLE     Direct Submission
  JOURNAL   Submitted (13-DEC-2016) Department of Pediatrics, Center of
            Excellence in Clinical Virology, Faculty of Medicine, Chulalongkorn
            University, Bangkok 10330, Thailand
COMMENT     ##Assembly-Data-START##
            Assembly Method       :: DNASTAR-Lasergene v. 6
            Sequencing Technology :: Sanger dideoxy sequencing
            ##Assembly-Data-END##
FEATURES             Location/Qualifiers
     source          1..603
                     /organism="Human respiratory syncytial virus B"
                     /mol_type="viral cRNA"
                     /isolate="TH-CU/C6207/2015"
                     /host="Homo sapiens"
                     /db_xref="taxon:208895"
                     /country="Thailand"
                     /collection_date="29-Oct-2015"
                     /note="group: B"
     CDS             <1..603
                     /note="G protein"
                     /codon_start=1
                     /product="attachment glycoprotein"
                     /protein_id="APY20419"
                     /translation="IHTNSATISPNTKSETHHTTAQTKSTTSTPTQNNKPSTKPRPKN
                     PPKKDDYHFEVFNFVPCSICGNNQLCKSICKTIPSNKPKKKPTTKPTNKPPTKTTNKR
                     DPKTLAKTPKKETTINPTKKPTPKTTERDISTPQSTVLDITTSKHTERDTSTSQSIAL
                     DTTTSKHTTQQQSLYSTTPENTPNSTQTPTASEPSTSNST"
ORIGIN
        1 atccacacaa actcagccac aatatcaccc aatacaaaat cagaaacaca ccatacaaca
       61 gcacaaacca aaagcacaac ctctactcca acacagaaca acaagccgag cacaaaacca
      121 cgtccaaaaa atccaccaaa aaaagatgat taccattttg aagtgttcaa ctttgttccc
      181 tgtagtatat gtggcaacaa tcaactctgc aaatccattt gcaaaacaat accaagcaac
      241 aaaccaaaga aaaaaccaac tacaaaaccc acaaacaaac cacctactaa aaccacaaac
      301 aaaagagacc ccaaaacact agccaaaaca ccgaaaaaag aaaccaccat taacccaaca
      361 aaaaaaccaa cccccaagac tacagaaaga gacatcagca ccccacaatc cactgtgctc
      421 gacataacca catcaaaaca cacagaaaga gacaccagca cctcacaatc cattgcgctt
      481 gacacaacca catcaaaaca cacaacccaa cagcaatctc tctactcaac cacccccgaa
```

```
     541 aacacaccca actccacaca aacacccaca gcatccgagc cctccacatc aaattctacc
     601 taa
//
LOCUS       KY328079                 603 bp    cRNA    linear   VRL 13-DEC-2016
DEFINITION  Human respiratory syncytial virus B isolate TH-CU/C6249/2015
            attachment glycoprotein gene, partial cds.
ACCESSION   KY328079
VERSION     KY328079
KEYWORDS    .
SOURCE      Human respiratory syncytial virus B
  ORGANISM  Human respiratory syncytial virus B
            Viruses; ssRNA viruses; ssRNA negative-strand viruses;
            Mononegavirales; Pneumoviridae; Orthopneumovirus.
REFERENCE   1  (bases 1 to 603)
  AUTHORS   Thongpan,I., Mauleekoonphairoj,J., Vichiwattana,P., Korkong,S.,
            Vongpunsawad,S. and Poovorawan,Y.
  TITLE     Molecular Characterization of Respiratory Syncytial Virus in
            Thailand, 2012-2015
  JOURNAL   Unpublished
REFERENCE   2  (bases 1 to 603)
  AUTHORS   Thongpan,I., Mauleekoonphairoj,J., Vichiwattana,P., Korkong,S.,
            Vongpunsawad,S. and Poovorawan,Y.
  TITLE     Direct Submission
  JOURNAL   Submitted (13-DEC-2016) Department of Pediatrics, Center of
            Excellence in Clinical Virology, Faculty of Medicine, Chulalongkorn
            University, Bangkok 10330, Thailand
COMMENT     ##Assembly-Data-START##
            Assembly Method       :: DNASTAR-Lasergene v. 6
            Sequencing Technology :: Sanger dideoxy sequencing
            ##Assembly-Data-END##
FEATURES             Location/Qualifiers
     source          1..603
                     /organism="Human respiratory syncytial virus B"
                     /mol_type="viral cRNA"
                     /isolate="TH-CU/C6249/2015"
                     /host="Homo sapiens"
                     /db_xref="taxon:208895"
                     /country="Thailand"
                     /collection_date="19-Nov-2015"
                     /note="group: B"
     CDS             <1..603
                     /note="G protein"
                     /codon_start=1
                     /product="attachment glycoprotein"
                     /protein_id="APY20420"
                     /translation="IHTNSATISPNTKSETHHTTAQTKSTTSTPTQNNKPSTKPRPKN
                     PPKKDDYHFEVFNFVPCSICGNNQLCKSICKTIPSNKPKKKPTTKPTNKPPTKTTNKR
                     DPKTLAKTPKKETTINPTKKPTPKTTERDISTPQSTVLDITTSKHTERDTSTSQSIAL
                     DTTTSKHTTQQQSLYSTTPENTPNSTQTPTASEPSTSNST"
ORIGIN
        1 atccacacaa actcagccac aatatcaccc aatacaaaat cagaaacaca ccatacaaca
       61 gcacaaacca aaagcacaac ctctactcca acacagaaca acaagccgag cacaaaacca
      121 cgtccaaaaa atccaccaaa aaaagatgat taccattttg aagtgttcaa ctttgttccc
      181 tgtagtatat gtggcaacaa tcaactctgc aaatccattt gcaaaacaat accaagcaac
```

241 aaaccaaaga aaaaaccaac tacaaaaccc acaaacaaac cacctactaa aaccacaaac
301 aaaagagacc ccaaaacact agccaaaaca ccgaaaaaag aaaccaccat taacccaaca
361 aaaaaaccaa cccccaagac tacagaaaga gacatcagca ccccacaatc cactgtgctc
421 gacataacca catcaaaaca cacagaaaga gacaccagca cctcacaatc cattgcgctt
481 gacacaacca catcaaaaca cacaacccaa cagcaatctc tctactcaac cacccccgaa
541 aacacaccca actccacaca aacacccaca gcatccgagc cctccacatc aaattctacc
601 taa
//
LOCUS KY328080 603 bp cRNA linear VRL 13-DEC-2016
DEFINITION Human respiratory syncytial virus B isolate TH-CU/C6217/2015
attachment glycoprotein gene, partial cds.
ACCESSION KY328080
VERSION KY328080
KEYWORDS .
SOURCE Human respiratory syncytial virus B
ORGANISM Human respiratory syncytial virus B
Viruses; ssRNA viruses; ssRNA negative-strand viruses;
Mononegavirales; Pneumoviridae; Orthopneumovirus.
REFERENCE 1 (bases 1 to 603)
AUTHORS Thongpan,I., Mauleekoonphairoj,J., Vichiwattana,P., Korkong,S.,
Vongpunsawad,S. and Poovorawan,Y.
TITLE Molecular Characterization of Respiratory Syncytial Virus in
Thailand, 2012-2015
JOURNAL Unpublished
REFERENCE 2 (bases 1 to 603)
AUTHORS Thongpan,I., Mauleekoonphairoj,J., Vichiwattana,P., Korkong,S.,
Vongpunsawad,S. and Poovorawan,Y.
TITLE Direct Submission
JOURNAL Submitted (13-DEC-2016) Department of Pediatrics, Center of
Excellence in Clinical Virology, Faculty of Medicine, Chulalongkorn
University, Bangkok 10330, Thailand
COMMENT ##Assembly-Data-START##
Assembly Method :: DNASTAR-Lasergene v. 6
Sequencing Technology :: Sanger dideoxy sequencing
##Assembly-Data-END##
FEATURES Location/Qualifiers
source 1..603
/organism="Human respiratory syncytial virus B"
/mol_type="viral cRNA"
/isolate="TH-CU/C6217/2015"
/host="Homo sapiens"
/db_xref="taxon:208895"
/country="Thailand"
/collection_date="04-Nov-2015"
/note="group: B"
CDS <1..603
/note="G protein"
/codon_start=1
/product="attachment glycoprotein"
/protein_id="APY20421"
/translation="IHTNSATISPNTKSETHHTTAQTKSTTSTPTQNNKPSTKPRPKN
PPKKDDYHFEVFNFVPCSICGNNQLCKSICKTIPSNKPKKKPTTKPTNKPPTKTTNKR
DPKTLAKTPKKETTINPTKKPTPKTTERDISTPQSTVLDITTSKHTERDTSTSQSIAL
DTTTSKHTTQQQSLYSTTPENTPNSTQTPTASEPSTSNST"

```
ORIGIN
        1 atccacacaa actcagccac aatatcaccc aatacaaaat cagaaacaca ccatacaaca
       61 gcacaaacca aaagcacaac ctctactcca acacagaaca acaagccgag cacaaaacca
      121 cgtccaaaaa atccaccaaa aaaagatgat taccattttg aagtgttcaa ctttgttccc
      181 tgtagtatat gtggcaacaa tcaactctgc aaatccattt gcaaaacaat accaagcaac
      241 aaaccaaaga aaaaaccaac tacaaaaccc acaaacaaac cacctactaa aaccacaaac
      301 aaaagagacc ccaaaacact agccaaaaca ccgaaaaaag aaaccaccat taacccaaca
      361 aaaaaaccaa cccccaagac tacagaaaga gacatcagca ccccacaatc cactgtgctc
      421 gacataacca catcaaaaca cacagaaaga gacaccagca cctcacaatc cattgcgctt
      481 gacacaacca catcaaaaca cacaacccaa cagcaatctc tctactcaac cacccccgaa
      541 aacacaccca actccacaca aacacccaca gcatccgagc cctccacatc aaattctacc
      601 taa
//
LOCUS       KY328081                 603 bp    cRNA    linear   VRL 13-DEC-2016
DEFINITION  Human respiratory syncytial virus B isolate TH-CU/C6250/2015
            attachment glycoprotein gene, partial cds.
ACCESSION   KY328081
VERSION     KY328081
KEYWORDS    .
SOURCE      Human respiratory syncytial virus B
  ORGANISM  Human respiratory syncytial virus B
            Viruses; ssRNA viruses; ssRNA negative-strand viruses;
            Mononegavirales; Pneumoviridae; Orthopneumovirus.
REFERENCE   1  (bases 1 to 603)
  AUTHORS   Thongpan,I., Mauleekoonphairoj,J., Vichiwattana,P., Korkong,S.,
            Vongpunsawad,S. and Poovorawan,Y.
  TITLE     Molecular Characterization of Respiratory Syncytial Virus in
            Thailand, 2012-2015
  JOURNAL   Unpublished
REFERENCE   2  (bases 1 to 603)
  AUTHORS   Thongpan,I., Mauleekoonphairoj,J., Vichiwattana,P., Korkong,S.,
            Vongpunsawad,S. and Poovorawan,Y.
  TITLE     Direct Submission
  JOURNAL   Submitted (13-DEC-2016) Department of Pediatrics, Center of
            Excellence in Clinical Virology, Faculty of Medicine, Chulalongkorn
            University, Bangkok 10330, Thailand
COMMENT     ##Assembly-Data-START##
            Assembly Method       :: DNASTAR-Lasergene v. 6
            Sequencing Technology :: Sanger dideoxy sequencing
            ##Assembly-Data-END##
FEATURES             Location/Qualifiers
     source          1..603
                     /organism="Human respiratory syncytial virus B"
                     /mol_type="viral cRNA"
                     /isolate="TH-CU/C6250/2015"
                     /host="Homo sapiens"
                     /db_xref="taxon:208895"
                     /country="Thailand"
                     /collection_date="19-Nov-2015"
                     /note="group: B"
     CDS             <1..603
                     /note="G protein"
                     /codon_start=1
                     /product="attachment glycoprotein"
```

/protein_id="APY20422"
/translation="IHTNSATISPNTKSETHHTTAQTKSTTSTPTQNNKPSTKPRPKN
PPKKDDYHFEVFNFVPCSICGNNQLCKSICKTIPSNKPKKKPTTKPTNKPPTKTTNKR
DPKTLAKTPKKETTINPTKKPTPKTTERDISTPQSTVLDITTSKHTERDTSTSQSIAL
DTTTSKHTTQQQSLYSTTPENTPNSTQTPTASEPSTSNST"
ORIGIN
1 atccacacaa actcagccac aatatcaccc aatacaaaat cagaaacaca ccatacaaca
61 gcacaaacca aaagcacaac ctctactcca acacagaaca acaagccgag cacaaaacca
121 cgtccaaaaa atccaccaaa aaaagatgat taccattttg aagtgttcaa ctttgttccc
181 tgtagtatat gtggcaacaa tcaactctgc aaatccattt gcaaaacaat accaagcaac
241 aaaccaaaga aaaaaccaac tacaaaaccc acaaacaaac cacctactaa aaccacaaac
301 aaaagagacc ccaaaacact agccaaaaca ccgaaaaaag aaaccaccat taacccaaca
361 aaaaaaccaa cccccaagac tacagaaaga gacatcagca ccccacaatc cactgtgctc
421 gacataacca catcaaaaca cacagaaaga gacaccagca cctcacaatc cattgcgctt
481 gacacaacca catcaaaaca cacaacccaa cagcaatctc tctactcaac cacccccgaa
541 aacacaccca actccacaca aacacccaca gcatccgagc cctccacatc aaattctacc
601 taa
//
LOCUS KY328082 603 bp cRNA linear VRL 13-DEC-2016
DEFINITION Human respiratory syncytial virus B isolate TH-CU/C6226/2015
attachment glycoprotein gene, partial cds.
ACCESSION KY328082
VERSION KY328082
KEYWORDS .
SOURCE Human respiratory syncytial virus B
ORGANISM Human respiratory syncytial virus B
Viruses; ssRNA viruses; ssRNA negative-strand viruses;
Mononegavirales; Pneumoviridae; Orthopneumovirus.
REFERENCE 1 (bases 1 to 603)
AUTHORS Thongpan,I., Mauleekoonphairoj,J., Vichiwattana,P., Korkong,S.,
Vongpunsawad,S. and Poovorawan,Y.
TITLE Molecular Characterization of Respiratory Syncytial Virus in
Thailand, 2012-2015
JOURNAL Unpublished
REFERENCE 2 (bases 1 to 603)
AUTHORS Thongpan,I., Mauleekoonphairoj,J., Vichiwattana,P., Korkong,S.,
Vongpunsawad,S. and Poovorawan,Y.
TITLE Direct Submission
JOURNAL Submitted (13-DEC-2016) Department of Pediatrics, Center of
Excellence in Clinical Virology, Faculty of Medicine, Chulalongkorn
University, Bangkok 10330, Thailand
COMMENT ##Assembly-Data-START##
Assembly Method :: DNASTAR-Lasergene v. 6
Sequencing Technology :: Sanger dideoxy sequencing
##Assembly-Data-END##
FEATURES Location/Qualifiers
source 1..603
/organism="Human respiratory syncytial virus B"
/mol_type="viral cRNA"
/isolate="TH-CU/C6226/2015"
/host="Homo sapiens"
/db_xref="taxon:208895"
/country="Thailand"
/collection_date="04-Nov-2015"

```
                     /note="group: B"
     CDS             <1..603
                     /note="G protein"
                     /codon_start=1
                     /product="attachment glycoprotein"
                     /protein_id="APY20423"
                     /translation="IHTNSATISPNTKSETHHTTAQTKSTTSTPTQNNKPSTKPRPKN
                     PPKKDDYHFEVFNFVPCSICGNNQLCKSICKTIPSNKPKKKPTTKPTNKPPTKTTNKR
                     DPKTLAKTPKKETTINPTKKPTPKTTERDISTPQSTVLDITTSKHTERDTSTSQSIAL
                     DTTTSKHTTQQQSLYSTTPENTPNSTQTPTASEPSTSNST"
ORIGIN      
        1 atccacacaa actcagccac aatatcaccc aatacaaaat cagaaacaca ccatacaaca
       61 gcacaaacca aaagcacaac ctctactcca acacagaaca acaagccgag cacaaaacca
      121 cgtccaaaaa atccaccaaa aaaagatgat taccattttg aagtgttcaa ctttgttccc
      181 tgtagtatat gtggcaacaa tcaactctgc aaatccattt gcaaaacaat accaagcaac
      241 aaaccaaaga aaaaaccaac tacaaaaccc acaaacaaac cacctactaa aaccacaaac
      301 aaaagagacc ccaaaacact agccaaaaca ccgaaaaaag aaaccaccat taacccaaca
      361 aaaaaaccaa cccccaagac tacagaaaga gacatcagca ccccacaatc cactgtgctc
      421 gacataacca catcaaaaca cacagaaaga gacaccagca cctcacaatc cattgcgctt
      481 gacacaacca catcaaaaca cacaacccaa cagcaatctc tctactcaac cacccccgaa
      541 aacacaccca actccacaca aacacccaca gcatccgagc cctccacatc aaattctacc
      601 taa
//
LOCUS       KY328083                 603 bp    cRNA    linear   VRL 13-DEC-2016
DEFINITION  Human respiratory syncytial virus B isolate B/TH-CU/B12563/2015
            attachment glycoprotein gene, partial cds.
ACCESSION   KY328083
VERSION     KY328083
KEYWORDS    .
SOURCE      Human respiratory syncytial virus B
  ORGANISM  Human respiratory syncytial virus B
            Viruses; ssRNA viruses; ssRNA negative-strand viruses;
            Mononegavirales; Pneumoviridae; Orthopneumovirus.
REFERENCE   1  (bases 1 to 603)
  AUTHORS   Thongpan,I., Mauleekoonphairoj,J., Vichiwattana,P., Korkong,S.,
            Vongpunsawad,S. and Poovorawan,Y.
  TITLE     Molecular Characterization of Respiratory Syncytial Virus in
            Thailand, 2012-2015
  JOURNAL   Unpublished
REFERENCE   2  (bases 1 to 603)
  AUTHORS   Thongpan,I., Mauleekoonphairoj,J., Vichiwattana,P., Korkong,S.,
            Vongpunsawad,S. and Poovorawan,Y.
  TITLE     Direct Submission
  JOURNAL   Submitted (13-DEC-2016) Department of Pediatrics, Center of
            Excellence in Clinical Virology, Faculty of Medicine, Chulalongkorn
            University, Bangkok 10330, Thailand
COMMENT     ##Assembly-Data-START##
            Assembly Method       :: DNASTAR-Lasergene v. 6
            Sequencing Technology :: Sanger dideoxy sequencing
            ##Assembly-Data-END##
FEATURES             Location/Qualifiers
     source          1..603
                     /organism="Human respiratory syncytial virus B"
                     /mol_type="viral cRNA"
```

```
                     /isolate="B/TH-CU/B12563/2015"
                     /host="Homo sapiens"
                     /db_xref="taxon:208895"
                     /country="Thailand"
                     /collection_date="16-Aug-2015"
                     /note="group: B"
     CDS             <1..603
                     /note="G protein"
                     /codon_start=1
                     /product="attachment glycoprotein"
                     /protein_id="APY20424"
                     /translation="IHTNSATISPNTKSETHHTTAQTKSTTSTPTQNNKPSTKPRPKN
                     PPKKDDYHFEVFNFVPCSICGNNQLCKSICKTIPSNKPKKKPTTKPTNKPPTKTTNKR
                     DPKTLAKTPKKETTINPTKKPTPKTTERDISTPQSTVLNITTSKHTERDTSTSQSIAL
                     DTTTSKHTTQQQSLYSTTPENTPNSTQTPTASEPSTSNST"
ORIGIN
        1 atccacacaa actcagccac aatatcaccc aatacaaaat cagaaacaca ccatacaaca
       61 gcacaaacca aaagcacaac ctctactcca acacagaaca acaagccgag cacaaaacca
      121 cgtccaaaaa atccaccaaa aaaagatgat taccattttg aagtgttcaa ctttgttccc
      181 tgtagtatat gtggcaacaa tcaactctgc aaatccattt gcaaaacaat accaagcaac
      241 aaaccaaaga aaaaaccaac tacaaaaccc acaaacaaac cacctactaa aaccacaaac
      301 aaaagagacc ccaaaacact agccaaaaca ccgaaaaaag aaaccaccat taacccaaca
      361 aaaaaaccaa cccccaagac tacagaaaga gacatcagca ccccacaatc cactgtgctc
      421 aacataacca catcaaaaca cacagaaaga gacaccagca cctcacaatc cattgcgctt
      481 gacacaacca catcaaaaca cacaacccaa cagcaatctc tctactcaac cacccccgaa
      541 aacacaccca actccacaca aacacccaca gcatccgagc cctccacatc aaattctacc
      601 taa
//
LOCUS       KY328084                 603 bp    cRNA    linear   VRL 13-DEC-2016
DEFINITION  Human respiratory syncytial virus B isolate TH-CU/C6552/2015
            attachment glycoprotein gene, partial cds.
ACCESSION   KY328084
VERSION     KY328084
KEYWORDS    .
SOURCE      Human respiratory syncytial virus B
  ORGANISM  Human respiratory syncytial virus B
            Viruses; ssRNA viruses; ssRNA negative-strand viruses;
            Mononegavirales; Pneumoviridae; Orthopneumovirus.
REFERENCE   1  (bases 1 to 603)
  AUTHORS   Thongpan,I., Mauleekoonphairoj,J., Vichiwattana,P., Korkong,S.,
            Vongpunsawad,S. and Poovorawan,Y.
  TITLE     Molecular Characterization of Respiratory Syncytial Virus in
            Thailand, 2012-2015
  JOURNAL   Unpublished
REFERENCE   2  (bases 1 to 603)
  AUTHORS   Thongpan,I., Mauleekoonphairoj,J., Vichiwattana,P., Korkong,S.,
            Vongpunsawad,S. and Poovorawan,Y.
  TITLE     Direct Submission
  JOURNAL   Submitted (13-DEC-2016) Department of Pediatrics, Center of
            Excellence in Clinical Virology, Faculty of Medicine, Chulalongkorn
            University, Bangkok 10330, Thailand
COMMENT     ##Assembly-Data-START##
            Assembly Method       :: DNASTAR-Lasergene v. 6
            Sequencing Technology :: Sanger dideoxy sequencing
```

```
          ##Assembly-Data-END##
FEATURES             Location/Qualifiers
     source          1..603
                     /organism="Human respiratory syncytial virus B"
                     /mol_type="viral cRNA"
                     /isolate="TH-CU/C6552/2015"
                     /host="Homo sapiens"
                     /db_xref="taxon:208895"
                     /country="Thailand"
                     /collection_date="19-Nov-2015"
                     /note="group: B"
     CDS             <1..603
                     /note="G protein"
                     /codon_start=1
                     /product="attachment glycoprotein"
                     /protein_id="APY20425"
                     /translation="IHTNSATISPNTKSETHHTTAQTKSTTSTPTQNNKPSTKPRPKN
                     SPKKDDYHFEVFNFVPCSICGNNQLCKSICKTIPSNKPKKKPTTKPTNKPPTKTTNKR
                     DPKTLAKTPKKETTINPTKKPTPKTTERDISTPQSTVLDITTSKHTERDTSTSQSIAL
                     DTTTSKHTTQQQSLYSTTPENTPNSTQTPTASEPSTSNST"
ORIGIN
        1 atccacacaa actcagccac aatatcaccc aatacaaaat cagaaacaca ccatacaaca
       61 gcacaaacca aaagcacaac ctctactcca acacaaaaca acaagccgag cacaaaacca
      121 cgtccaaaaa attcaccaaa aaaagatgat taccattttg aagtgttcaa ctttgttccc
      181 tgtagtatat gtggcaacaa tcaactctgc aaatccattt gcaaaacaat accaagcaac
      241 aaaccaaaga aaaaaccaac tacaaaaccc acaaacaaac cacctactaa aaccacaaac
      301 aaaagagacc ccaaaacact agccaaaaca ccgaaaaaag aaaccaccat taacccaaca
      361 aaaaaaccaa cccccaagac tacagaaaga gacatcagca ccccacaatc cactgtgctc
      421 gacataacca catcaaaaca cacagaaaga gacaccagca cctcacaatc cattgcgctt
      481 gacacaacca catcaaaaca cacaacccaa cagcaatctc tctactcaac cacccccgaa
      541 aacacaccca actccacaca aacacccaca gcatccgagc cctccacatc aaattctacc
      601 taa
//
LOCUS       KY328085                 603 bp    cRNA    linear   VRL 13-DEC-2016
DEFINITION  Human respiratory syncytial virus B isolate TH-CU/B10639/2014
            attachment glycoprotein gene, partial cds.
ACCESSION   KY328085
VERSION     KY328085
KEYWORDS    .
SOURCE      Human respiratory syncytial virus B
  ORGANISM  Human respiratory syncytial virus B
            Viruses; ssRNA viruses; ssRNA negative-strand viruses;
            Mononegavirales; Pneumoviridae; Orthopneumovirus.
REFERENCE   1  (bases 1 to 603)
  AUTHORS   Thongpan,I., Mauleekoonphairoj,J., Vichiwattana,P., Korkong,S.,
            Vongpunsawad,S. and Poovorawan,Y.
  TITLE     Molecular Characterization of Respiratory Syncytial Virus in
            Thailand, 2012-2015
  JOURNAL   Unpublished
REFERENCE   2  (bases 1 to 603)
  AUTHORS   Thongpan,I., Mauleekoonphairoj,J., Vichiwattana,P., Korkong,S.,
            Vongpunsawad,S. and Poovorawan,Y.
  TITLE     Direct Submission
  JOURNAL   Submitted (13-DEC-2016) Department of Pediatrics, Center of
```

```
            Excellence in Clinical Virology, Faculty of Medicine, Chulalongkorn
            University, Bangkok 10330, Thailand
COMMENT     ##Assembly-Data-START##
            Assembly Method       :: DNASTAR-Lasergene v. 6
            Sequencing Technology :: Sanger dideoxy sequencing
            ##Assembly-Data-END##
FEATURES             Location/Qualifiers
     source          1..603
                     /organism="Human respiratory syncytial virus B"
                     /mol_type="viral cRNA"
                     /isolate="TH-CU/B10639/2014"
                     /host="Homo sapiens"
                     /db_xref="taxon:208895"
                     /country="Thailand"
                     /collection_date="12-Aug-2014"
                     /note="group: B"
     CDS             <1..603
                     /note="G protein"
                     /codon_start=1
                     /product="attachment glycoprotein"
                     /protein_id="APY20426"
                     /translation="IHTNSATISPNTKSETHHTTAQTKGTTSTSTQNNKPSTKPRPKN
                     PPKKDDYHFEVFNFVPCSICGNNQLCKSICKTIPSNKPKKKPTTKPTNKPPTKTTNKR
                     DPKTLAKTPKKENTINPTKEPTPKTTERDTSTPQSTVLDITTSKHTERDTSTSQSIVL
                     DTTTSKHTTQQQSLYSTTPENTPNSTQTPTASEPSTSNST"
ORIGIN
        1 atccacacaa actcagccac aatatcaccc aatacaaaat cagaaacaca ccatacaaca
       61 gcacaaacca aaggcacaac ctctacttca acacagaaca acaagccaag cacaaaacca
      121 cgtccaaaaa atccaccaaa aaaagatgat taccattttg aagtgttcaa ctttgttccc
      181 tgtagtatat gtggcaacaa tcaactctgc aaatccattt gcaaaacaat accaagcaat
      241 aaaccaaaga aaaaaccaac tacaaaaccc acaaacaaac cacctaccaa aaccacaaac
      301 aaaagagacc ccaaaacact agccaaaaca ccgaaaaaag aaaacaccat taacccaaca
      361 aaagaaccaa ccccaaagac tacagaaaga gacaccagca ccccacaatc cactgtgctc
      421 gacataacca catcaaaaca cacagaaaga gacaccagca cctcacaatc cattgtgctt
      481 gacacaacca catcaaaaca cacaacccaa cagcaatctc tctactcaac cacccccgaa
      541 aacacaccca actccacaca aacacccaca gcatccgagc cctccacatc aaattctacc
      601 taa
//
LOCUS       KY328086                 603 bp    cRNA    linear   VRL 13-DEC-2016
DEFINITION  Human respiratory syncytial virus B isolate B/TH-CU/B10640/2014
            attachment glycoprotein gene, partial cds.
ACCESSION   KY328086
VERSION     KY328086
KEYWORDS    .
SOURCE      Human respiratory syncytial virus B
  ORGANISM  Human respiratory syncytial virus B
            Viruses; ssRNA viruses; ssRNA negative-strand viruses;
            Mononegavirales; Pneumoviridae; Orthopneumovirus.
REFERENCE   1  (bases 1 to 603)
  AUTHORS   Thongpan,I., Mauleekoonphairoj,J., Vichiwattana,P., Korkong,S.,
            Vongpunsawad,S. and Poovorawan,Y.
  TITLE     Molecular Characterization of Respiratory Syncytial Virus in
            Thailand, 2012-2015
  JOURNAL   Unpublished
```

```
REFERENCE   2  (bases 1 to 603)
  AUTHORS   Thongpan,I., Mauleekoonphairoj,J., Vichiwattana,P., Korkong,S.,
            Vongpunsawad,S. and Poovorawan,Y.
  TITLE     Direct Submission
  JOURNAL   Submitted (13-DEC-2016) Department of Pediatrics, Center of
            Excellence in Clinical Virology, Faculty of Medicine, Chulalongkorn
            University, Bangkok 10330, Thailand
COMMENT     ##Assembly-Data-START##
            Assembly Method       :: DNASTAR-Lasergene v. 6
            Sequencing Technology :: Sanger dideoxy sequencing
            ##Assembly-Data-END##
FEATURES             Location/Qualifiers
     source          1..603
                     /organism="Human respiratory syncytial virus B"
                     /mol_type="viral cRNA"
                     /isolate="B/TH-CU/B10640/2014"
                     /host="Homo sapiens"
                     /db_xref="taxon:208895"
                     /country="Thailand"
                     /collection_date="12-Aug-2014"
                     /note="group: B"
     CDS             <1..603
                     /note="G protein"
                     /codon_start=1
                     /product="attachment glycoprotein"
                     /protein_id="APY20427"
                     /translation="IHTNSATISPNTKSETHHTTAQTKGTTSTSTQNNKPSTKPRPKN
                     PPKKDDYHFEVFNFVPCSICGNNQLCKSICKTIPSNKPKKKPTTKPTNKPPTKTTNKR
                     DPKTLAKTPKKENTINPTKEPTPKTTERDTSTPQSTVLDITTSKHTERDTSTSQSIVL
                     DTTTSKHTTQQQSLYSTTPENTPNSTQTPTASEPSTSNST"
ORIGIN
        1 atccacacaa actcagccac aatatcaccc aatacaaaat cagaaacaca ccatacaaca
       61 gcacaaacca aaggcacaac ctctacttca acacagaaca acaagccaag cacaaaacca
      121 cgtccaaaaa atccaccaaa aaaagatgat taccattttg aagtgttcaa ctttgttccc
      181 tgtagtatat gtggcaacaa tcaactctgc aaatccattt gcaaaacaat accaagcaat
      241 aaaccaaaga aaaaaccaac tacaaaaccc acaaacaaac cacctaccaa aaccacaaac
      301 aaaagagacc ccaaaacact agccaaaaca ccgaaaaaag aaaacaccat taacccaaca
      361 aaagaaccaa ccccaaagac tacagaaaga gacaccagca ccccacaatc cactgtgctc
      421 gacataacca catcaaaaca cacagaaaga gacaccagca cctcacaatc cattgtgctt
      481 gacacaacca catcaaaaca cacaacccaa cagcaatctc tctactcaac cacccccgaa
      541 aacacaccca actccacaca aacacccaca gcatccgagc cctccacatc aaattctacc
      601 taa
//
LOCUS       KY328087                 603 bp    cRNA    linear   VRL 13-DEC-2016
DEFINITION  Human respiratory syncytial virus B isolate TH-CU/C5164/2014
            attachment glycoprotein gene, partial cds.
ACCESSION   KY328087
VERSION     KY328087
KEYWORDS    .
SOURCE      Human respiratory syncytial virus B
  ORGANISM  Human respiratory syncytial virus B
            Viruses; ssRNA viruses; ssRNA negative-strand viruses;
            Mononegavirales; Pneumoviridae; Orthopneumovirus.
REFERENCE   1  (bases 1 to 603)
```

```
  AUTHORS   Thongpan,I., Mauleekoonphairoj,J., Vichiwattana,P., Korkong,S.,
            Vongpunsawad,S. and Poovorawan,Y.
  TITLE     Molecular Characterization of Respiratory Syncytial Virus in
            Thailand, 2012-2015
  JOURNAL   Unpublished
REFERENCE   2  (bases 1 to 603)
  AUTHORS   Thongpan,I., Mauleekoonphairoj,J., Vichiwattana,P., Korkong,S.,
            Vongpunsawad,S. and Poovorawan,Y.
  TITLE     Direct Submission
  JOURNAL   Submitted (13-DEC-2016) Department of Pediatrics, Center of
            Excellence in Clinical Virology, Faculty of Medicine, Chulalongkorn
            University, Bangkok 10330, Thailand
COMMENT     ##Assembly-Data-START##
            Assembly Method       :: DNASTAR-Lasergene v. 6
            Sequencing Technology :: Sanger dideoxy sequencing
            ##Assembly-Data-END##
FEATURES             Location/Qualifiers
     source          1..603
                     /organism="Human respiratory syncytial virus B"
                     /mol_type="viral cRNA"
                     /isolate="TH-CU/C5164/2014"
                     /host="Homo sapiens"
                     /db_xref="taxon:208895"
                     /country="Thailand"
                     /collection_date="01-Oct-2014"
                     /note="group: B"
     CDS             <1..603
                     /note="G protein"
                     /codon_start=1
                     /product="attachment glycoprotein"
                     /protein_id="APY20428"
                     /translation="IHTNSATISPNTKSETHHTTAQTKGTTSTPTQNNKPSTKPRPKN
                     PPKKDDYHFEVFNFVPCSICGNNQLCKSICKTIPSNKPKKKPTTKPTNKPTTKTTNKR
                     DLKTLAKTPKKETTINPTKKPTPKTTERDTSTPQSTVLDITTSKHTERDTSTSQSIAL
                     DTTTSKHTTQQQSLYSTTPENTPNSTQTPTASEPSTSNST"
ORIGIN
        1 atccacacaa actcagccac aatatcaccc aatacaaaat cagaaacaca ccatacaaca
       61 gcacaaacca aaggcacaac ctctactcca acacagaaca acaagccaag cacaaaacca
      121 cgtccaaaaa atccaccaaa aaaagatgat tatcattttg aagtgttcaa ctttgttccc
      181 tgtagtatat gtggcaacaa tcaactctgc aaatccattt gcaaaacaat accaagcaat
      241 aaaccaaaga aaaaaccaac tacaaaaccc acaaacaaac caactaccaa aaccacaaac
      301 aaaagagacc tcaaaacact agccaaaaca ccgaaaaaag aaaccaccat taacccaaca
      361 aaaaaaccaa cccccaagac tacagaaaga gacaccagca ccccacaatc cactgtgctc
      421 gacataacca catcaaaaca cacagaaaga gacaccagca cctcacaatc cattgcgctc
      481 gacacaacca catcaaaaca cacaacccaa cagcaatctc tctactcaac cacccccgaa
      541 aacacaccca actccacaca aacacccaca gcatccgagc cctccacatc aaattctacc
      601 taa
//
LOCUS       KY328088                 603 bp    cRNA    linear   VRL 13-DEC-2016
DEFINITION  Human respiratory syncytial virus B isolate TH-CU/B10981/2014
            attachment glycoprotein gene, partial cds.
ACCESSION   KY328088
VERSION     KY328088
KEYWORDS    .
```

```
SOURCE      Human respiratory syncytial virus B
  ORGANISM  Human respiratory syncytial virus B
            Viruses; ssRNA viruses; ssRNA negative-strand viruses;
            Mononegavirales; Pneumoviridae; Orthopneumovirus.
REFERENCE   1  (bases 1 to 603)
  AUTHORS   Thongpan,I., Mauleekoonphairoj,J., Vichiwattana,P., Korkong,S.,
            Vongpunsawad,S. and Poovorawan,Y.
  TITLE     Molecular Characterization of Respiratory Syncytial Virus in
            Thailand, 2012-2015
  JOURNAL   Unpublished
REFERENCE   2  (bases 1 to 603)
  AUTHORS   Thongpan,I., Mauleekoonphairoj,J., Vichiwattana,P., Korkong,S.,
            Vongpunsawad,S. and Poovorawan,Y.
  TITLE     Direct Submission
  JOURNAL   Submitted (13-DEC-2016) Department of Pediatrics, Center of
            Excellence in Clinical Virology, Faculty of Medicine, Chulalongkorn
            University, Bangkok 10330, Thailand
COMMENT     ##Assembly-Data-START##
            Assembly Method       :: DNASTAR-Lasergene v. 6
            Sequencing Technology :: Sanger dideoxy sequencing
            ##Assembly-Data-END##
FEATURES             Location/Qualifiers
     source          1..603
                     /organism="Human respiratory syncytial virus B"
                     /mol_type="viral cRNA"
                     /isolate="TH-CU/B10981/2014"
                     /host="Homo sapiens"
                     /db_xref="taxon:208895"
                     /country="Thailand"
                     /collection_date="11-Sep-2014"
                     /note="group: B"
     CDS             <1..603
                     /note="G protein"
                     /codon_start=1
                     /product="attachment glycoprotein"
                     /protein_id="APY20429"
                     /translation="IHTNSATISPNTKSETHHTTAQTKGTTSTPTQNNKPSTKPRPKN
                     PPKKDDYHFEVFNFVPCSICGNNQLCKSICKTIPSNKPKKKPTTKPTNKPTTKTTNKR
                     DLKTLAKTPKKETTINPTKKPTPKTTERDTSTPQSTVLDITTSKHTERDTSTSQSIAL
                     DTTTSKHTTQQQSLYSTTPENTPNSTQTPTASEPSTSNST"
ORIGIN
        1 atccacacaa actcagccac aatatcaccc aatacaaaat cagaaacaca ccatacaaca
       61 gcacaaacca aaggcacaac ctctactcca acacagaaca acaagccaag cacaaaacca
      121 cgtccaaaaa atccaccaaa aaaagatgat tatcattttg aagtgttcaa ctttgttccc
      181 tgtagtatat gtggcaacaa tcaactctgc aaatccattt gcaaaacaat accaagcaat
      241 aaaccaaaga aaaaaccaac tacaaaaccc acaaacaagc caactaccaa aaccacaaac
      301 aaaagagacc tcaaaacact agccaaaaca ccgaaaaaag aaaccaccat taacccaaca
      361 aaaaaaccaa cccccaagac tacagaaaga gacaccagca ccccacaatc cactgtgctc
      421 gacataacca catcaaaaca cacagaaaga gacaccagca cctcacaatc cattgcgctc
      481 gacacaacca catcaaaaca cacaacccaa cagcaatctc tctactcaac cacccccgaa
      541 aacacaccca actccacaca aacacccaca gcatccgagc cctccacatc aaattctacc
      601 taa
//
LOCUS       KY328089                 603 bp    cRNA    linear   VRL 13-DEC-2016
```

```
DEFINITION  Human respiratory syncytial virus B isolate TH-CU/C4085/2013
            attachment glycoprotein gene, partial cds.
ACCESSION   KY328089
VERSION     KY328089
KEYWORDS    .
SOURCE      Human respiratory syncytial virus B
  ORGANISM  Human respiratory syncytial virus B
            Viruses; ssRNA viruses; ssRNA negative-strand viruses;
            Mononegavirales; Pneumoviridae; Orthopneumovirus.
REFERENCE   1  (bases 1 to 603)
  AUTHORS   Thongpan,I., Mauleekoonphairoj,J., Vichiwattana,P., Korkong,S.,
            Vongpunsawad,S. and Poovorawan,Y.
  TITLE     Molecular Characterization of Respiratory Syncytial Virus in
            Thailand, 2012-2015
  JOURNAL   Unpublished
REFERENCE   2  (bases 1 to 603)
  AUTHORS   Thongpan,I., Mauleekoonphairoj,J., Vichiwattana,P., Korkong,S.,
            Vongpunsawad,S. and Poovorawan,Y.
  TITLE     Direct Submission
  JOURNAL   Submitted (13-DEC-2016) Department of Pediatrics, Center of
            Excellence in Clinical Virology, Faculty of Medicine, Chulalongkorn
            University, Bangkok 10330, Thailand
COMMENT     ##Assembly-Data-START##
            Assembly Method       :: DNASTAR-Lasergene v. 6
            Sequencing Technology :: Sanger dideoxy sequencing
            ##Assembly-Data-END##
FEATURES             Location/Qualifiers
     source          1..603
                     /organism="Human respiratory syncytial virus B"
                     /mol_type="viral cRNA"
                     /isolate="TH-CU/C4085/2013"
                     /host="Homo sapiens"
                     /db_xref="taxon:208895"
                     /country="Thailand"
                     /collection_date="01-Oct-2013"
                     /note="group: B"
     CDS             <1..603
                     /note="G protein"
                     /codon_start=1
                     /product="attachment glycoprotein"
                     /protein_id="APY20430"
                     /translation="IHTNSATISPNTKSETHHTTAQTKGRTSTPTQNNKPSTKPRPKN
                     PPKKDDYHFEVFNFVPCSICGNNQLCKSICKTIPSNKPKKKPTTKPTNKPPTKTTNKR
                     DPKTLAKTPKKETTINPTKKPTPKTTERDTSTPHSTVLDTTTSKHTERDTSTSQSIAL
                     DTTTSKHTTQQQSLYSTTPENTPNSTQTPTASEPSTSNST"
ORIGIN
        1 atccacacaa actcagccac aatatcacct aatacaaaat cagaaacaca ccatacaaca
       61 gcacaaacca aaggcagaac ctctactcca acacagaaca acaagccaag cacaaaacca
      121 cgtccaaaaa atccaccaaa aaaagatgat taccattttg aagtgttcaa ctttgttccc
      181 tgtagtatat gtggcaacaa tcaactctgc aaatccattt gcaaaacaat accaagcaat
      241 aaaccaaaga aaaaaccaac tacaaaaccc acaaacaaac cacctaccaa aaccacaaac
      301 aaaagagacc ccaaaacact agccaaaaca ccgaaaaaag aaaccaccat taacccaaca
      361 aaaaaaccaa cccccaagac cacagaaaga gacaccagca ccccacactc cactgtgctc
      421 gacacaacca catcaaaaca cacagaaaga gacaccagca cctcacaatc cattgcgctt
```

481 gacacaacca catcaaaaca cacaacccaa cagcaatctc tctactcaac cacccccgaa
541 aacacaccca actccacaca aacacccaca gcatccgagc cctccacatc aaattctacc
601 taa
//
LOCUS   KY328090   603 bp   cRNA   linear   VRL 13-DEC-2016
DEFINITION Human respiratory syncytial virus B isolate TH-CU/C4078/2013
attachment glycoprotein gene, partial cds.
ACCESSION   KY328090
VERSION   KY328090
KEYWORDS   .
SOURCE   Human respiratory syncytial virus B
ORGANISM Human respiratory syncytial virus B
Viruses; ssRNA viruses; ssRNA negative-strand viruses;
Mononegavirales; Pneumoviridae; Orthopneumovirus.
REFERENCE   1 (bases 1 to 603)
AUTHORS   Thongpan,I., Mauleekoonphairoj,J., Vichiwattana,P., Korkong,S.,
Vongpunsawad,S. and Poovorawan,Y.
TITLE   Molecular Characterization of Respiratory Syncytial Virus in
Thailand, 2012-2015
JOURNAL   Unpublished
REFERENCE   2 (bases 1 to 603)
AUTHORS   Thongpan,I., Mauleekoonphairoj,J., Vichiwattana,P., Korkong,S.,
Vongpunsawad,S. and Poovorawan,Y.
TITLE   Direct Submission
JOURNAL   Submitted (13-DEC-2016) Department of Pediatrics, Center of
Excellence in Clinical Virology, Faculty of Medicine, Chulalongkorn
University, Bangkok 10330, Thailand
COMMENT   ##Assembly-Data-START##
Assembly Method   :: DNASTAR-Lasergene v. 6
Sequencing Technology :: Sanger dideoxy sequencing
##Assembly-Data-END##
FEATURES   Location/Qualifiers
source   1..603
/organism="Human respiratory syncytial virus B"
/mol_type="viral cRNA"
/isolate="TH-CU/C4078/2013"
/host="Homo sapiens"
/db_xref="taxon:208895"
/country="Thailand"
/collection_date="01-Sep-2013"
/note="group: B"
CDS   <1..603
/note="G protein"
/codon_start=1
/product="attachment glycoprotein"
/protein_id="APY20431"
/translation="IHTNSATISPNTKSETHHTTAQTKGRTSTPTQNNKPSTKPRPKN
PPKKDDYHFEVFNFVPCSICGNNQLCKSICKTIPSNKPKKKPTTKPTNKPPTKTTNKR
DPKTLAKTPKKETTINPTKKPTPKTTERDTSTPQSTVLDTTTSKRTERDTSTSQSIAL
DTTTSKHTTQQQSLYSTTPENTPNSTQTPTASEPSTSNST"
ORIGIN
1 atccacacaa actcagccac aatatcacct aatacaaaat cagaaacaca ccatacaaca
61 gcacaaacca aaggcagaac ctctactcca acacagaaca acaagccaag cacaaaacca
121 cgtccaaaaa atccaccaaa aaaagatgat taccattttg aagtgttcaa ctttgttccc

```
     181 tgtagtatat gtggcaacaa tcaactctgc aaatccattt gcaaaacaat accaagcaat
     241 aaaccaaaga aaaaaccaac tacaaaaccc acaaacaaac cacctaccaa aaccacaaac
     301 aaaagagacc ccaaaacact agccaaaaca ccgaaaaaag aaaccaccat taacccaaca
     361 aaaaaaccaa cccccaagac cacagaaaga gacaccagca ccccacaatc cactgtgctc
     421 gacacaacca catcaaaacg cacagaaaga gacaccagca cctcacaatc cattgcgctt
     481 gacacaacca catcaaaaca cacaacccaa cagcaatctc tctactcaac cacccccgaa
     541 aacacaccca actccacaca aacacccaca gcatccgagc cctccacatc aaattctacc
     601 taa
//
LOCUS       KY328091                 603 bp    cRNA    linear   VRL 13-DEC-2016
DEFINITION  Human respiratory syncytial virus B isolate TH-CU410/2012
            attachment glycoprotein gene, partial cds.
ACCESSION   KY328091
VERSION     KY328091
KEYWORDS    .
SOURCE      Human respiratory syncytial virus B
  ORGANISM  Human respiratory syncytial virus B
            Viruses; ssRNA viruses; ssRNA negative-strand viruses;
            Mononegavirales; Pneumoviridae; Orthopneumovirus.
REFERENCE   1  (bases 1 to 603)
  AUTHORS   Thongpan,I., Mauleekoonphairoj,J., Vichiwattana,P., Korkong,S.,
            Vongpunsawad,S. and Poovorawan,Y.
  TITLE     Molecular Characterization of Respiratory Syncytial Virus in
            Thailand, 2012-2015
  JOURNAL   Unpublished
REFERENCE   2  (bases 1 to 603)
  AUTHORS   Thongpan,I., Mauleekoonphairoj,J., Vichiwattana,P., Korkong,S.,
            Vongpunsawad,S. and Poovorawan,Y.
  TITLE     Direct Submission
  JOURNAL   Submitted (13-DEC-2016) Department of Pediatrics, Center of
            Excellence in Clinical Virology, Faculty of Medicine, Chulalongkorn
            University, Bangkok 10330, Thailand
COMMENT     ##Assembly-Data-START##
            Assembly Method       :: DNASTAR-Lasergene v. 6
            Sequencing Technology :: Sanger dideoxy sequencing
            ##Assembly-Data-END##
FEATURES             Location/Qualifiers
     source          1..603
                     /organism="Human respiratory syncytial virus B"
                     /mol_type="viral cRNA"
                     /isolate="TH-CU410/2012"
                     /host="Homo sapiens"
                     /db_xref="taxon:208895"
                     /country="Thailand"
                     /collection_date="01-Aug-2012"
                     /note="group: B"
     CDS             <1..603
                     /note="G protein"
                     /codon_start=1
                     /product="attachment glycoprotein"
                     /protein_id="APY20432"
                     /translation="IHTNSATISPNTKSETHHTTAQTKGRTSTPTQNNKPSTKPRPKN
                     PPKKDDYHFEVFNFVPCSICGNNQLCKSICKTIPSNKPKKKPTTKPTNKPPTKTTNKR
                     DPKTLAKTPKKETTINPTKKPTPKTTERDTSTPQSTVLDTTTSKHTERDTSTSQSIAL
```

DTTTSKHTTQQQSLYSTTPENTPNSTQTPTASEPSTSNST"
ORIGIN
1 atccacacaa actcagccac aatatcacct aatacaaaat cagaaacaca ccatacaaca
61 gcacaaacca aaggcagaac ctctactcca acacagaaca acaagccaag cacaaaacca
121 cgtccaaaaa atccaccaaa aaaagatgat taccattttg aagtgttcaa ctttgttccc
181 tgtagtatat gtggcaacaa tcaactctgc aaatccattt gcaaaacaat accaagcaat
241 aaaccaaaga aaaaaccaac tacaaaaccc acaaacaaac cacctaccaa aaccacaaac
301 aaaagagacc ccaaaacact agccaaaaca ccgaaaaaag aaaccaccat taacccaaca
361 aaaaaaccaa cccccaagac cacagaaaga gacaccagca ccccacaatc cactgtgctc
421 gacacaacca catcaaaaca cacagaaaga gacaccagca cctcacaatc cattgcgctt
481 gacacaacca catcaaaaca cacaacccaa cagcaatctc tctactcaac cacccccgaa
541 aacacaccca actccacaca aacacccaca gcatccgagc cctccacatc aaattctacc
601 taa
//
LOCUS KY328092 603 bp cRNA linear VRL 13-DEC-2016
DEFINITION Human respiratory syncytial virus B isolate TH-CU468/2012
attachment glycoprotein gene, partial cds.
ACCESSION KY328092
VERSION KY328092
KEYWORDS .
SOURCE Human respiratory syncytial virus B
ORGANISM Human respiratory syncytial virus B
Viruses; ssRNA viruses; ssRNA negative-strand viruses;
Mononegavirales; Pneumoviridae; Orthopneumovirus.
REFERENCE 1 (bases 1 to 603)
AUTHORS Thongpan,I., Mauleekoonphairoj,J., Vichiwattana,P., Korkong,S.,
Vongpunsawad,S. and Poovorawan,Y.
TITLE Molecular Characterization of Respiratory Syncytial Virus in
Thailand, 2012-2015
JOURNAL Unpublished
REFERENCE 2 (bases 1 to 603)
AUTHORS Thongpan,I., Mauleekoonphairoj,J., Vichiwattana,P., Korkong,S.,
Vongpunsawad,S. and Poovorawan,Y.
TITLE Direct Submission
JOURNAL Submitted (13-DEC-2016) Department of Pediatrics, Center of
Excellence in Clinical Virology, Faculty of Medicine, Chulalongkorn
University, Bangkok 10330, Thailand
COMMENT ##Assembly-Data-START##
Assembly Method :: DNASTAR-Lasergene v. 6
Sequencing Technology :: Sanger dideoxy sequencing
##Assembly-Data-END##
FEATURES Location/Qualifiers
source 1..603
/organism="Human respiratory syncytial virus B"
/mol_type="viral cRNA"
/isolate="TH-CU468/2012"
/host="Homo sapiens"
/db_xref="taxon:208895"
/country="Thailand"
/collection_date="01-Sep-2012"
/note="group: B"
CDS <1..603
/note="G protein"
/codon_start=1

```
                     /product="attachment glycoprotein"
                     /protein_id="APY20433"
                     /translation="IHTNSATISPNTKSETHHTTAQTKGRTSTPTQNNKPSTKPRPKN
                     PPKKDDYHFEVFNFVPCSICGNNQLCKSICKTIPSNKPKKKPTTKPTNKPPTKTTNKR
                     DPKTLAKTPKKETTINPTKKPTPKTTERDTSTPQSTVLDTTTSKHTERDTSTSQSIAL
                     DTTTSKHTTQQQSLYSTTPENTPNSTQTPTASEPSTSNST"
ORIGIN
        1 atccacacaa actcagccac aatatcacct aatacaaaat cagaaacaca ccatacaaca
       61 gcacaaacca aaggcagaac ctctactcca acacagaaca acaagccaag cacaaaacca
      121 cgtccaaaaa atccaccaaa aaaagatgat taccattttg aagtgttcaa ctttgttccc
      181 tgtagtatat gtggcaacaa tcaactctgc aaatccattt gcaaaacaat accaagcaat
      241 aaaccaaaga aaaaaccaac tacaaaaccc acaaacaaac cacctaccaa aaccacaaac
      301 aaaagagacc ccaaaacact agccaaaaca ccgaaaaaag aaaccaccat taacccaaca
      361 aaaaaaccaa cccccaagac cacagaaaga gacaccagca ccccacaatc cactgtgctc
      421 gacacaacca catcaaaaca cacagaaaga gacaccagca cctcacaatc cattgcgctt
      481 gacacaacca catcaaaaca cacaacccaa cagcaatctc tctactcaac cacccccgaa
      541 aacacaccca actccacaca aacacccaca gcatccgagc cctccacatc aaattctacc
      601 taa
//
LOCUS       KY328093                 603 bp    cRNA    linear   VRL 13-DEC-2016
DEFINITION  Human respiratory syncytial virus B isolate TH-CU404/2012
            attachment glycoprotein gene, partial cds.
ACCESSION   KY328093
VERSION     KY328093
KEYWORDS    .
SOURCE      Human respiratory syncytial virus B
  ORGANISM  Human respiratory syncytial virus B
            Viruses; ssRNA viruses; ssRNA negative-strand viruses;
            Mononegavirales; Pneumoviridae; Orthopneumovirus.
REFERENCE   1  (bases 1 to 603)
  AUTHORS   Thongpan,I., Mauleekoonphairoj,J., Vichiwattana,P., Korkong,S.,
            Vongpunsawad,S. and Poovorawan,Y.
  TITLE     Molecular Characterization of Respiratory Syncytial Virus in
            Thailand, 2012-2015
  JOURNAL   Unpublished
REFERENCE   2  (bases 1 to 603)
  AUTHORS   Thongpan,I., Mauleekoonphairoj,J., Vichiwattana,P., Korkong,S.,
            Vongpunsawad,S. and Poovorawan,Y.
  TITLE     Direct Submission
  JOURNAL   Submitted (13-DEC-2016) Department of Pediatrics, Center of
            Excellence in Clinical Virology, Faculty of Medicine, Chulalongkorn
            University, Bangkok 10330, Thailand
COMMENT     ##Assembly-Data-START##
            Assembly Method       :: DNASTAR-Lasergene v. 6
            Sequencing Technology :: Sanger dideoxy sequencing
            ##Assembly-Data-END##
FEATURES             Location/Qualifiers
     source          1..603
                     /organism="Human respiratory syncytial virus B"
                     /mol_type="viral cRNA"
                     /isolate="TH-CU404/2012"
                     /host="Homo sapiens"
                     /db_xref="taxon:208895"
                     /country="Thailand"
```

```
                     /collection_date="01-Aug-2012"
                     /note="group: B"
     CDS             <1..603
                     /note="G protein"
                     /codon_start=1
                     /product="attachment glycoprotein"
                     /protein_id="APY20434"
                     /translation="IHTNSATISPNTKSETHHTTAQTKGRTSTPTQNNKPSTKPRPKN
                     PPKKDDYHFEVFNFVPCSICGNNQLCKSICKTIPSNKPKKKPTTKPTNKPPTKTTNKR
                     DPKTLAKTPKKETTINPTKKPTPKTTERDTSTPQSTVLDTTTSKHTERDTSTSQSIAL
                     DTTTSKHTTQQQSLYSTTPENTPNSTQTPTASEPSTSNST"
ORIGIN
        1 atccacacaa actcagccac aatatcacct aatacaaaat cagaaacaca ccatacaaca
       61 gcacaaacca aaggcagaac ctctactcca acacagaaca acaagccaag cacaaaacca
      121 cgtccaaaaa atccaccaaa aaaagatgat taccattttg aagtgttcaa ctttgttccc
      181 tgtagtatat gtggcaacaa tcaactctgc aaatccattt gcaaaacaat accaagcaat
      241 aaaccaaaga aaaaaccaac tacaaaaccc acaaacaaac cacctaccaa aaccacaaac
      301 aaaagagacc ccaaaacact agccaaaaca ccgaaaaaag aaaccaccat taacccaaca
      361 aaaaaaccaa cccccaagac cacagaaaga gacaccagca ccccacaatc cactgtgctc
      421 gacacaacca catcaaaaca cacagaaaga gacaccagca cctcacaatc cattgcgctt
      481 gacacaacca catcaaaaca cacaacccaa cagcaatctc tctactcaac cacccccgaa
      541 aacacaccca actccacaca aacacccaca gcatccgagc cctccacatc aaattctacc
      601 taa
//
LOCUS       KY328094                 603 bp    cRNA    linear   VRL 13-DEC-2016
DEFINITION  Human respiratory syncytial virus B isolate TH-CU/C4014/2013
            attachment glycoprotein gene, partial cds.
ACCESSION   KY328094
VERSION     KY328094
KEYWORDS    .
SOURCE      Human respiratory syncytial virus B
  ORGANISM  Human respiratory syncytial virus B
            Viruses; ssRNA viruses; ssRNA negative-strand viruses;
            Mononegavirales; Pneumoviridae; Orthopneumovirus.
REFERENCE   1  (bases 1 to 603)
  AUTHORS   Thongpan,I., Mauleekoonphairoj,J., Vichiwattana,P., Korkong,S.,
            Vongpunsawad,S. and Poovorawan,Y.
  TITLE     Molecular Characterization of Respiratory Syncytial Virus in
            Thailand, 2012-2015
  JOURNAL   Unpublished
REFERENCE   2  (bases 1 to 603)
  AUTHORS   Thongpan,I., Mauleekoonphairoj,J., Vichiwattana,P., Korkong,S.,
            Vongpunsawad,S. and Poovorawan,Y.
  TITLE     Direct Submission
  JOURNAL   Submitted (13-DEC-2016) Department of Pediatrics, Center of
            Excellence in Clinical Virology, Faculty of Medicine, Chulalongkorn
            University, Bangkok 10330, Thailand
COMMENT     ##Assembly-Data-START##
            Assembly Method       :: DNASTAR-Lasergene v. 6
            Sequencing Technology :: Sanger dideoxy sequencing
            ##Assembly-Data-END##
FEATURES             Location/Qualifiers
     source          1..603
                     /organism="Human respiratory syncytial virus B"
```

```
                     /mol_type="viral cRNA"
                     /isolate="TH-CU/C4014/2013"
                     /host="Homo sapiens"
                     /db_xref="taxon:208895"
                     /country="Thailand"
                     /collection_date="01-Aug-2013"
                     /note="group: B"
     CDS             <1..603
                     /note="G protein"
                     /codon_start=1
                     /product="attachment glycoprotein"
                     /protein_id="APY20435"
                     /translation="IHTNSATISPNTKSETHHTTAQTKGRTSTPTQNNKPSTKPRPKN
                     PPKKDDYHFEVFNFVPCSICGNNQLCKSICKTIPSNKPKKKPTTKPTNKPPTKTTNKR
                     DPKTLAKTPKKETTINPTKKPTPKTTERDTSTPQSTVLDTTTSKHTERDTSTSQSIAL
                     DTTTSKHTTQQQSLYSTTPENTPNSTQTPTASEPSTSNST"
ORIGIN
        1 atccacacaa actcagccac aatatcacct aatacaaaat cagaaacaca ccatacaaca
       61 gcacaaacca aaggcagaac ctctactcca acacagaaca acaagccaag cacaaaacca
      121 cgtccaaaaa atccaccaaa aaaagatgat taccattttg aagtgttcaa ctttgttccc
      181 tgtagtatat gtggcaacaa tcaactctgc aaatccattt gcaaaacaat accaagcaat
      241 aaaccaaaga aaaaaccaac tacaaaaccc acaaacaaac cacctaccaa aaccacaaac
      301 aaaagagacc ccaaaacact agccaaaaca ccgaaaaaag aaaccaccat taacccaaca
      361 aaaaaaccaa cccccaagac cacagaaaga gacaccagca ccccacaatc cactgtgctc
      421 gacacaacca catcaaaaca cacagaaaga gacaccagca cctcacaatc cattgcgctt
      481 gacacaacca catcaaaaca cacaacccaa cagcaatctc tctactcaac cacccccgaa
      541 aacacaccca actccacaca aacacccaca gcatccgagc cctccacatc aaattctacc
      601 taa
//
LOCUS       KY328095                 603 bp    cRNA    linear   VRL 13-DEC-2016
DEFINITION  Human respiratory syncytial virus B isolate TH-CU485/2012
            attachment glycoprotein gene, partial cds.
ACCESSION   KY328095
VERSION     KY328095
KEYWORDS    .
SOURCE      Human respiratory syncytial virus B
  ORGANISM  Human respiratory syncytial virus B
            Viruses; ssRNA viruses; ssRNA negative-strand viruses;
            Mononegavirales; Pneumoviridae; Orthopneumovirus.
REFERENCE   1  (bases 1 to 603)
  AUTHORS   Thongpan,I., Mauleekoonphairoj,J., Vichiwattana,P., Korkong,S.,
            Vongpunsawad,S. and Poovorawan,Y.
  TITLE     Molecular Characterization of Respiratory Syncytial Virus in
            Thailand, 2012-2015
  JOURNAL   Unpublished
REFERENCE   2  (bases 1 to 603)
  AUTHORS   Thongpan,I., Mauleekoonphairoj,J., Vichiwattana,P., Korkong,S.,
            Vongpunsawad,S. and Poovorawan,Y.
  TITLE     Direct Submission
  JOURNAL   Submitted (13-DEC-2016) Department of Pediatrics, Center of
            Excellence in Clinical Virology, Faculty of Medicine, Chulalongkorn
            University, Bangkok 10330, Thailand
COMMENT     ##Assembly-Data-START##
            Assembly Method       :: DNASTAR-Lasergene v. 6
```

```
           Sequencing Technology :: Sanger dideoxy sequencing
           ##Assembly-Data-END##
FEATURES             Location/Qualifiers
     source          1..603
                     /organism="Human respiratory syncytial virus B"
                     /mol_type="viral cRNA"
                     /isolate="TH-CU485/2012"
                     /host="Homo sapiens"
                     /db_xref="taxon:208895"
                     /country="Thailand"
                     /collection_date="01-Oct-2012"
                     /note="group: B"
     CDS             <1..603
                     /note="G protein"
                     /codon_start=1
                     /product="attachment glycoprotein"
                     /protein_id="APY20436"
                     /translation="IHTNSATISPNTKSETHHTTAQTKGRTSTPTQNNKPSTKPRPKN
                     PPKKDDYHFEVFNFVPCSICGNNQLCKSICKTIPSNKPKKKPTTKPTNKPPTKTTNKR
                     DPKTLAKTPKKETTINPTKKPTPKTTERDTSTPQSTVLDTTTSKHTERDTSTSQSIAL
                     DTTTSKHTTQQQSLYSTTPENTPNSTQTPTASEPSTSNST"
ORIGIN
        1 atccacacaa actcagccac aatatcacct aatacaaaat cagaaacaca ccatacaaca
       61 gcacaaacca aaggcagaac ctctactcca acacagaaca acaagccaag cacaaaacca
      121 cgtccaaaaa atccaccaaa aaaagatgat taccattttg aagtgttcaa ctttgttccc
      181 tgtagtatat gtggcaacaa tcaactctgc aaatccattt gcaaaacaat accaagcaat
      241 aaaccaaaga aaaaaccaac tacaaaaccc acaaacaaac cacctaccaa aaccacaaac
      301 aaaagagacc ccaaaacact agccaaaaca ccgaaaaaag aaaccaccat taacccaaca
      361 aaaaaaccaa cccccaagac cacagaaaga gacaccagca ccccacaatc cactgtgctc
      421 gacacaacca catcaaaaca cacagaaaga gacaccagca cctcacaatc cattgcgctt
      481 gacacaacca catcaaaaca cacaacccaa cagcaatctc tctactcaac cacccccgaa
      541 aacacaccca actccacaca aacacccaca gcatccgagc cctccacatc aaattctacc
      601 taa
//
LOCUS       KY328096                 603 bp    cRNA    linear   VRL 13-DEC-2016
DEFINITION  Human respiratory syncytial virus B isolate TH-CU405/2012
            attachment glycoprotein gene, partial cds.
ACCESSION   KY328096
VERSION     KY328096
KEYWORDS    .
SOURCE      Human respiratory syncytial virus B
  ORGANISM  Human respiratory syncytial virus B
            Viruses; ssRNA viruses; ssRNA negative-strand viruses;
            Mononegavirales; Pneumoviridae; Orthopneumovirus.
REFERENCE   1  (bases 1 to 603)
  AUTHORS   Thongpan,I., Mauleekoonphairoj,J., Vichiwattana,P., Korkong,S.,
            Vongpunsawad,S. and Poovorawan,Y.
  TITLE     Molecular Characterization of Respiratory Syncytial Virus in
            Thailand, 2012-2015
  JOURNAL   Unpublished
REFERENCE   2  (bases 1 to 603)
  AUTHORS   Thongpan,I., Mauleekoonphairoj,J., Vichiwattana,P., Korkong,S.,
            Vongpunsawad,S. and Poovorawan,Y.
  TITLE     Direct Submission
```

JOURNAL   Submitted (13-DEC-2016) Department of Pediatrics, Center of
Excellence in Clinical Virology, Faculty of Medicine, Chulalongkorn
University, Bangkok 10330, Thailand
COMMENT   ##Assembly-Data-START##
Assembly Method   :: DNASTAR-Lasergene v. 6
Sequencing Technology :: Sanger dideoxy sequencing
##Assembly-Data-END##
FEATURES   Location/Qualifiers
source   1..603
/organism="Human respiratory syncytial virus B"
/mol_type="viral cRNA"
/isolate="TH-CU405/2012"
/host="Homo sapiens"
/db_xref="taxon:208895"
/country="Thailand"
/collection_date="01-Aug-2012"
/note="group: B"
CDS   <1..603
/note="G protein"
/codon_start=1
/product="attachment glycoprotein"
/protein_id="APY20437"
/translation="IHTNSATISPNTKSETHHTTAQTKGRTSTPTQNNKPSTKPRPKN
PPKKDDYHFEVFNFVPCSICGNNQLCKSICKTIPSNKPKKKPTTKPTNKPPTKTTNKR
DPKTLAKTPKKETTINPTKKPTPKTTERDTSTPQSTVLDTTTSKHTERDTSTSQSIAL
DTTTSKHTTQQQSLYSTTPENTPNSTQTPTASEPSTSNST"
ORIGIN
1 atccacacaa actcagccac aatatcacct aatacaaaat cagaaacaca ccatacaaca
61 gcacaaacca aaggcagaac ctctactcca acacagaaca acaagccaag cacaaaacca
121 cgtccaaaaa atccaccaaa aaaagatgat taccattttg aagtgttcaa ctttgttccc
181 tgtagtatat gtggcaacaa tcaactctgc aaatccattt gcaaaacaat accaagcaat
241 aaaccaaaga aaaaaccaac tacaaaaccc acaaacaaac cacctaccaa aaccacaaac
301 aaaagagacc ccaaaacact agccaaaaca ccgaaaaaag aaaccaccat taacccaaca
361 aaaaaaccaa cccccaagac cacagaaaga gacaccagca ccccacaatc cactgtgctc
421 gacacaacca catcaaaaca cacagaaaga gacaccagca cctcacaatc cattgcgctt
481 gacacaacca catcaaaaca cacaacccaa cagcaatctc tctactcaac cacccccgaa
541 aacacaccca actccacaca aacacccaca gcatccgagc cctccacatc aaattctacc
601 taa
//
LOCUS   KY328097   603 bp   cRNA   linear   VRL 13-DEC-2016
DEFINITION Human respiratory syncytial virus B isolate TH-CU460/2012
attachment glycoprotein gene, partial cds.
ACCESSION   KY328097
VERSION   KY328097
KEYWORDS   .
SOURCE   Human respiratory syncytial virus B
ORGANISM Human respiratory syncytial virus B
Viruses; ssRNA viruses; ssRNA negative-strand viruses;
Mononegavirales; Pneumoviridae; Orthopneumovirus.
REFERENCE   1 (bases 1 to 603)
AUTHORS   Thongpan,I., Mauleekoonphairoj,J., Vichiwattana,P., Korkong,S.,
Vongpunsawad,S. and Poovorawan,Y.
TITLE   Molecular Characterization of Respiratory Syncytial Virus in
Thailand, 2012-2015

file:///D|/Final%20peerJ/GenBank%20KY327937-KY328148_.txt[3/10/2560 14:59:50]

```
  JOURNAL   Unpublished
REFERENCE   2  (bases 1 to 603)
  AUTHORS   Thongpan,I., Mauleekoonphairoj,J., Vichiwattana,P., Korkong,S.,
            Vongpunsawad,S. and Poovorawan,Y.
  TITLE     Direct Submission
  JOURNAL   Submitted (13-DEC-2016) Department of Pediatrics, Center of
            Excellence in Clinical Virology, Faculty of Medicine, Chulalongkorn
            University, Bangkok 10330, Thailand
COMMENT     ##Assembly-Data-START##
            Assembly Method       :: DNASTAR-Lasergene v. 6
            Sequencing Technology :: Sanger dideoxy sequencing
            ##Assembly-Data-END##
FEATURES             Location/Qualifiers
     source          1..603
                     /organism="Human respiratory syncytial virus B"
                     /mol_type="viral cRNA"
                     /isolate="TH-CU460/2012"
                     /host="Homo sapiens"
                     /db_xref="taxon:208895"
                     /country="Thailand"
                     /collection_date="01-Sep-2012"
                     /note="group: B"
     CDS             <1..603
                     /note="G protein"
                     /codon_start=1
                     /product="attachment glycoprotein"
                     /protein_id="APY20438"
                     /translation="IHTNSATISPNTKSETHHTTAQTKGRTSTPTQNNKPSTKPRPKN
                     PPKKDDYHFEVFNFVPCSICGNNQLCKSICKTIPSNKPKKKPTTKPTNKPPTKTTNKR
                     DPKTLAKTPKKETTINPTKKPTPKTTERDTSTPQSTVLDTTTSKHTERDTSTSQSIAL
                     DTTTSKHTTQQQSLYSTTPENTPNSTQTPTASEPSTSNST"
ORIGIN
        1 atccacacaa actcagccac aatatcacct aatacaaaat cagaaacaca ccatacaaca
       61 gcacaaacca aaggcagaac ctctactcca acacagaaca acaagccaag cacaaaacca
      121 cgtccaaaaa atccaccaaa aaaagatgat taccattttg aagtgttcaa ctttgttccc
      181 tgtagtatat gtggcaacaa tcaactctgc aaatccattt gcaaaacaat accaagcaat
      241 aaaccaaaga aaaaaccaac tacaaaaccc acaaacaaac cacctaccaa aaccacaaac
      301 aaaagagacc ccaaaacact agccaaaaca ccgaaaaaag aaaccaccat taacccaaca
      361 aaaaaaccaa cccccaagac cacagaaaga gacaccagca ccccacaatc cactgtgctc
      421 gacacaacca catcaaaaca cacagaaaga gacaccagca cctcacaatc cattgcgctt
      481 gacacaacca catcaaaaca cacaacccaa cagcaatctc tctactcaac cacccccgaa
      541 aacacaccca actccacaca aacacccaca gcatccgagc cctccacatc aaattctacc
      601 taa
//
LOCUS       KY328098                 603 bp    cRNA    linear   VRL 13-DEC-2016
DEFINITION  Human respiratory syncytial virus B isolate TH-CU/C4149/2013
            attachment glycoprotein gene, partial cds.
ACCESSION   KY328098
VERSION     KY328098
KEYWORDS    .
SOURCE      Human respiratory syncytial virus B
  ORGANISM  Human respiratory syncytial virus B
            Viruses; ssRNA viruses; ssRNA negative-strand viruses;
            Mononegavirales; Pneumoviridae; Orthopneumovirus.
```

```
REFERENCE   1  (bases 1 to 603)
  AUTHORS   Thongpan,I., Mauleekoonphairoj,J., Vichiwattana,P., Korkong,S.,
            Vongpunsawad,S. and Poovorawan,Y.
  TITLE     Molecular Characterization of Respiratory Syncytial Virus in
            Thailand, 2012-2015
  JOURNAL   Unpublished
REFERENCE   2  (bases 1 to 603)
  AUTHORS   Thongpan,I., Mauleekoonphairoj,J., Vichiwattana,P., Korkong,S.,
            Vongpunsawad,S. and Poovorawan,Y.
  TITLE     Direct Submission
  JOURNAL   Submitted (13-DEC-2016) Department of Pediatrics, Center of
            Excellence in Clinical Virology, Faculty of Medicine, Chulalongkorn
            University, Bangkok 10330, Thailand
COMMENT     ##Assembly-Data-START##
            Assembly Method       :: DNASTAR-Lasergene v. 6
            Sequencing Technology :: Sanger dideoxy sequencing
            ##Assembly-Data-END##
FEATURES             Location/Qualifiers
     source          1..603
                     /organism="Human respiratory syncytial virus B"
                     /mol_type="viral cRNA"
                     /isolate="TH-CU/C4149/2013"
                     /host="Homo sapiens"
                     /db_xref="taxon:208895"
                     /country="Thailand"
                     /collection_date="01-Sep-2013"
                     /note="group: B"
     CDS             <1..603
                     /note="G protein"
                     /codon_start=1
                     /product="attachment glycoprotein"
                     /protein_id="APY20439"
                     /translation="IHTNSATISPNTKSETHHTTAQTKGRTSTPTQNNKPSTKPRPKN
                     PPKKDDYHFEVFNFVPCSICGNNQLCKSICKTIPSNKPKKKPTTKPTNKPPTKTTNKR
                     DPKTLAKTPKKETTINPTKKPTPKTTERDTSTPQSTVLDTTTSKHTERDTSTSQSIAL
                     DTTTSKHTTQQQSLYSTTPENTPNSTQTPTASEPSTSNST"
ORIGIN      
        1 atccacacaa actcagccac aatatcacct aatacaaaat cagaaacaca ccatacaaca
       61 gcacaaacca aaggcagaac ctctactcca acacagaaca acaagccaag cacaaaacca
      121 cgtccaaaaa atccaccaaa aaaagatgat taccattttg aagtgttcaa ctttgttccc
      181 tgtagtatat gtggcaacaa tcaactctgc aaatccattt gcaaaacaat accaagcaat
      241 aaaccaaaga aaaaaccaac tacaaaaccc acaaacaaac cacctaccaa aaccacaaac
      301 aaaagagacc ccaaaacact agccaaaaca ccgaaaaaag aaaccaccat taacccaaca
      361 aaaaaaccaa cccccaagac cacagaaaga gacaccagca ccccacaatc cactgtgctc
      421 gacacaacca catcaaaaca cacagaaaga gacaccagca cctcacaatc cattgcgctt
      481 gacacaacca catcaaaaca cacaacccaa cagcaatctc tctactcaac cacccccgaa
      541 aacacaccca actccacaca aacacccaca gcatccgagc cctccacatc aaattctacc
      601 taa
//
LOCUS       KY328099                 603 bp    cRNA    linear   VRL 13-DEC-2016
DEFINITION  Human respiratory syncytial virus B isolate TH-CU/C3137/2012
            attachment glycoprotein gene, partial cds.
ACCESSION   KY328099
VERSION     KY328099
```

```
KEYWORDS    .
SOURCE      Human respiratory syncytial virus B
  ORGANISM  Human respiratory syncytial virus B
            Viruses; ssRNA viruses; ssRNA negative-strand viruses;
            Mononegavirales; Pneumoviridae; Orthopneumovirus.
REFERENCE   1  (bases 1 to 603)
  AUTHORS   Thongpan,I., Mauleekoonphairoj,J., Vichiwattana,P., Korkong,S.,
            Vongpunsawad,S. and Poovorawan,Y.
  TITLE     Molecular Characterization of Respiratory Syncytial Virus in
            Thailand, 2012-2015
  JOURNAL   Unpublished
REFERENCE   2  (bases 1 to 603)
  AUTHORS   Thongpan,I., Mauleekoonphairoj,J., Vichiwattana,P., Korkong,S.,
            Vongpunsawad,S. and Poovorawan,Y.
  TITLE     Direct Submission
  JOURNAL   Submitted (13-DEC-2016) Department of Pediatrics, Center of
            Excellence in Clinical Virology, Faculty of Medicine, Chulalongkorn
            University, Bangkok 10330, Thailand
COMMENT     ##Assembly-Data-START##
            Assembly Method       :: DNASTAR-Lasergene v. 6
            Sequencing Technology :: Sanger dideoxy sequencing
            ##Assembly-Data-END##
FEATURES             Location/Qualifiers
     source          1..603
                     /organism="Human respiratory syncytial virus B"
                     /mol_type="viral cRNA"
                     /isolate="TH-CU/C3137/2012"
                     /host="Homo sapiens"
                     /db_xref="taxon:208895"
                     /country="Thailand"
                     /collection_date="01-Aug-2012"
                     /note="group: B"
     CDS             <1..603
                     /note="G protein"
                     /codon_start=1
                     /product="attachment glycoprotein"
                     /protein_id="APY20440"
                     /translation="IHTNSATISPNTKSETHHTTAQTKGRTSTPTQNNKPSTKPRPKN
                     PPKKDDYHFEVFNFVPCSICGNNQLCKSICKTIPSNKPKKKPTTKPTNKPPTKTTNKR
                     DPKTLAKTPKKETTINPTKKPTPKTTERDTSTPQSTVLDTTTSKHTERDTSTSQSIAL
                     DTTTSKHTTQQQSLYSTTPENTPNSTQTPTASEPSTSNST"
ORIGIN
        1 atccacacaa actcagccac aatatcacct aatacaaaat cagaaacaca ccatacaaca
       61 gcacaaacca aaggcagaac ctctactcca acacagaaca acaagccaag cacaaaacca
      121 cgtccaaaaa atccaccaaa aaaagatgat taccattttg aagtgttcaa ctttgttccc
      181 tgtagtatat gtggcaacaa tcaactctgc aaatccattt gcaaaacaat accaagcaat
      241 aaaccaaaga aaaaaccaac tacaaaaccc acaaacaaac cacctaccaa aaccacaaac
      301 aaaagagacc ccaaaacact agccaaaaca ccgaaaaaag aaaccaccat taacccaaca
      361 aaaaaaccaa cccccaagac cacagaaaga gacaccagca ccccacaatc cactgtgctc
      421 gacacaacca catcaaaaca cacagaaaga gacaccagca cctcacaatc cattgcgctt
      481 gacacaacca catcaaaaca cacaacccaa cagcaatctc tctactcaac cacccccgaa
      541 aacacaccca actccacaca aacacccaca gcatccgagc cctccacatc aaattctacc
      601 taa
//
```

```
LOCUS       KY328100                 603 bp    cRNA    linear   VRL 13-DEC-2016
DEFINITION  Human respiratory syncytial virus B isolate TH-CU/C4153/2013
            attachment glycoprotein gene, partial cds.
ACCESSION   KY328100
VERSION     KY328100
KEYWORDS    .
SOURCE      Human respiratory syncytial virus B
  ORGANISM  Human respiratory syncytial virus B
            Viruses; ssRNA viruses; ssRNA negative-strand viruses;
            Mononegavirales; Pneumoviridae; Orthopneumovirus.
REFERENCE   1  (bases 1 to 603)
  AUTHORS   Thongpan,I., Mauleekoonphairoj,J., Vichiwattana,P., Korkong,S.,
            Vongpunsawad,S. and Poovorawan,Y.
  TITLE     Molecular Characterization of Respiratory Syncytial Virus in
            Thailand, 2012-2015
  JOURNAL   Unpublished
REFERENCE   2  (bases 1 to 603)
  AUTHORS   Thongpan,I., Mauleekoonphairoj,J., Vichiwattana,P., Korkong,S.,
            Vongpunsawad,S. and Poovorawan,Y.
  TITLE     Direct Submission
  JOURNAL   Submitted (13-DEC-2016) Department of Pediatrics, Center of
            Excellence in Clinical Virology, Faculty of Medicine, Chulalongkorn
            University, Bangkok 10330, Thailand
COMMENT     ##Assembly-Data-START##
            Assembly Method       :: DNASTAR-Lasergene v. 6
            Sequencing Technology :: Sanger dideoxy sequencing
            ##Assembly-Data-END##
FEATURES             Location/Qualifiers
     source          1..603
                     /organism="Human respiratory syncytial virus B"
                     /mol_type="viral cRNA"
                     /isolate="TH-CU/C4153/2013"
                     /host="Homo sapiens"
                     /db_xref="taxon:208895"
                     /country="Thailand"
                     /collection_date="01-Sep-2013"
                     /note="group: B"
     CDS             <1..603
                     /note="G protein"
                     /codon_start=1
                     /product="attachment glycoprotein"
                     /protein_id="APY20441"
                     /translation="IHTNSATISPNTKSETHHTTAQTKGRTSTPTQNNKPSTKPRPKN
                     PPKKDDYHFEVFNFVPCSICGNNQLCKSICKTIPSNKPKKKPTTKPTNKPPTKTTNKR
                     DPKTLAKTPKKETTINPTKKPTPKTTERDTSTPQSTVLDTTTSKHTERDTSTSQSIAL
                     DTTTSKHTTQQQSLYSTTPENTPNSTQTPTASEPSTSNST"
ORIGIN
        1 atccacacaa actcagccac aatatcacct aatacaaaat cagaaacaca ccatacaaca
       61 gcacaaacca aaggcagaac ctctactcca acacagaaca acaagccaag cacaaaacca
      121 cgtccaaaaa atccaccaaa aaaagatgat taccattttg aagtgttcaa ctttgttccc
      181 tgtagtatat gtggcaacaa tcaactctgc aaatccattt gcaaaacaat accaagcaat
      241 aaaccaaaga aaaaaccaac tacaaaaccc acaaacaaac cacctaccaa aaccacaaac
      301 aaaagagacc ccaaaacact agccaaaaca ccgaaaaaag aaaccaccat taacccaaca
      361 aaaaaaccaa cccccaagac cacagaaaga gacaccagca ccccacaatc cactgtgctc
```

```
     421 gacacaacca catcaaaaca cacagaaaga gacaccagca cctcacaatc cattgcgctt
     481 gacacaacca catcaaaaca cacaacccaa cagcaatctc tctactcaac cacccccgaa
     541 aacacaccca actccacaca aacacccaca gcatccgagc cctccacatc aaattctacc
     601 taa
//
LOCUS       KY328101                 603 bp    cRNA    linear   VRL 13-DEC-2016
DEFINITION  Human respiratory syncytial virus B isolate TH-CU/C4100/2013
            attachment glycoprotein gene, partial cds.
ACCESSION   KY328101
VERSION     KY328101
KEYWORDS    .
SOURCE      Human respiratory syncytial virus B
  ORGANISM  Human respiratory syncytial virus B
            Viruses; ssRNA viruses; ssRNA negative-strand viruses;
            Mononegavirales; Pneumoviridae; Orthopneumovirus.
REFERENCE   1  (bases 1 to 603)
  AUTHORS   Thongpan,I., Mauleekoonphairoj,J., Vichiwattana,P., Korkong,S.,
            Vongpunsawad,S. and Poovorawan,Y.
  TITLE     Molecular Characterization of Respiratory Syncytial Virus in
            Thailand, 2012-2015
  JOURNAL   Unpublished
REFERENCE   2  (bases 1 to 603)
  AUTHORS   Thongpan,I., Mauleekoonphairoj,J., Vichiwattana,P., Korkong,S.,
            Vongpunsawad,S. and Poovorawan,Y.
  TITLE     Direct Submission
  JOURNAL   Submitted (13-DEC-2016) Department of Pediatrics, Center of
            Excellence in Clinical Virology, Faculty of Medicine, Chulalongkorn
            University, Bangkok 10330, Thailand
COMMENT     ##Assembly-Data-START##
            Assembly Method       :: DNASTAR-Lasergene v. 6
            Sequencing Technology :: Sanger dideoxy sequencing
            ##Assembly-Data-END##
FEATURES             Location/Qualifiers
     source          1..603
                     /organism="Human respiratory syncytial virus B"
                     /mol_type="viral cRNA"
                     /isolate="TH-CU/C4100/2013"
                     /host="Homo sapiens"
                     /db_xref="taxon:208895"
                     /country="Thailand"
                     /collection_date="01-Sep-2013"
                     /note="group: B"
     CDS             <1..603
                     /note="G protein"
                     /codon_start=1
                     /product="attachment glycoprotein"
                     /protein_id="APY20442"
                     /translation="IHTNSATISPNTKSETHHTTAQTKGRTSTPTQNNKPSTKPRPKN
                     PPKKDDYHFEVFNFVPCSICGNNQLCKSICKTIPSNKPKKKPTTKPTNKPPTKTTNKR
                     DPKTLAKTPKKETTINPTKKPTPKTTERDTSTPQSTVLDTTTSKHTERDTSTSQSIAL
                     DTTTSKHTTQQQSLYSTTPENTPNSTQTPTASEPSTSNST"
ORIGIN
        1 atccacacaa actcagccac aatatcacct aatacaaaat cagaaacaca ccatacaaca
       61 gcacaaacca aaggcagaac ctctactcca acacagaaca acaagccaag cacaaaacca
```

121 cgtccaaaaa atccaccaaa aaaagatgat taccattttg aagtgttcaa ctttgttccc
181 tgtagtatat gtggcaacaa tcaactctgc aaatccattt gcaaaacaat accaagcaat
241 aaaccaaaga aaaaaccaac tacaaaaccc acaaacaaac cacctaccaa aaccacaaac
301 aaaagagacc ccaaaacact agccaaaaca ccgaaaaaag aaaccaccat taacccaaca
361 aaaaaaccaa cccccaagac cacagaaaga gacaccagca ccccacaatc cactgtgctc
421 gacacaacca catcaaaaca cacagaaaga gacaccagca cctcacaatc cattgcgctt
481 gacacaacca catcaaaaca cacaacccaa cagcaatctc tctactcaac cacccccgaa
541 aacacaccca actccacaca aacacccaca gcatccgagc cctccacatc aaattctacc
601 taa
//
LOCUS KY328102 585 bp cRNA linear VRL 13-DEC-2016
DEFINITION Human respiratory syncytial virus B isolate TH-CU407/2012
attachment glycoprotein gene, partial cds.
ACCESSION KY328102
VERSION KY328102
KEYWORDS .
SOURCE Human respiratory syncytial virus B
ORGANISM Human respiratory syncytial virus B
Viruses; ssRNA viruses; ssRNA negative-strand viruses;
Mononegavirales; Pneumoviridae; Orthopneumovirus.
REFERENCE 1 (bases 1 to 585)
AUTHORS Thongpan,I., Mauleekoonphairoj,J., Vichiwattana,P., Korkong,S.,
Vongpunsawad,S. and Poovorawan,Y.
TITLE Molecular Characterization of Respiratory Syncytial Virus in
Thailand, 2012-2015
JOURNAL Unpublished
REFERENCE 2 (bases 1 to 585)
AUTHORS Thongpan,I., Mauleekoonphairoj,J., Vichiwattana,P., Korkong,S.,
Vongpunsawad,S. and Poovorawan,Y.
TITLE Direct Submission
JOURNAL Submitted (13-DEC-2016) Department of Pediatrics, Center of
Excellence in Clinical Virology, Faculty of Medicine, Chulalongkorn
University, Bangkok 10330, Thailand
COMMENT ##Assembly-Data-START##
Assembly Method :: DNASTAR-Lasergene v. 6
Sequencing Technology :: Sanger dideoxy sequencing
##Assembly-Data-END##
FEATURES Location/Qualifiers
source 1..585
/organism="Human respiratory syncytial virus B"
/mol_type="viral cRNA"
/isolate="TH-CU407/2012"
/host="Homo sapiens"
/db_xref="taxon:208895"
/country="Thailand"
/collection_date="01-Aug-2012"
/note="group: B"
CDS <1..585
/note="G protein"
/codon_start=1
/product="attachment glycoprotein"
/protein_id="APY20443"
/translation="TISPNTKSETHHTTAQTKGRTSTPTQNNKPSTKPRPKNPPKKDD
YHFEVFNFVPCSICGNNQLCKSICKTIPSNKPKKKPTTKPTNKPPTKTTNKRDPKTLA

KTPKKETTINPTKKPTPKTTERDTSTPQSTVLDTTTSKHTERDTSTSQSIALDTTTSK
HTTQQQSLYSTTPENTPNSTQTPTASEPSTSNST"
ORIGIN
1 acaatatcac ctaatacaaa atcagaaaca caccatacaa cagcacaaac caaaggcaga
61 acctctactc caacacagaa caacaagcca agcacaaaac cacgtccaaa aaatccacca
121 aaaaaagatg attaccattt tgaagtgttc aactttgttc cctgtagtat atgtggcaac
181 aatcaactct gcaaatccat ttgcaaaaca ataccaagca ataaaccaaa gaaaaaacca
241 actacaaaac ccacaaacaa accacctacc aaaaccacaa acaaaagaga ccccaaaaca
301 ctagccaaaa caccgaaaaa agaaaccacc attaacccaa caaaaaaacc aacccccaag
361 accacagaaa gagacaccag caccccacaa tccactgtgc tcgacacaac cacatcaaaa
421 cacacagaaa gagacaccag cacctcacaa tccattgcgc ttgacacaac cacatcaaaa
481 cacacaaccc aacagcaatc tctctactca accaccccg aaaacacacc caactccaca
541 caaacaccca cagcatccga gccctccaca tcaaattcta cctaa
//
LOCUS KY328103 603 bp cRNA linear VRL 13-DEC-2016
DEFINITION Human respiratory syncytial virus B isolate TH-CU427/2012
attachment glycoprotein gene, partial cds.
ACCESSION KY328103
VERSION KY328103
KEYWORDS .
SOURCE Human respiratory syncytial virus B
ORGANISM Human respiratory syncytial virus B
Viruses; ssRNA viruses; ssRNA negative-strand viruses;
Mononegavirales; Pneumoviridae; Orthopneumovirus.
REFERENCE 1 (bases 1 to 603)
AUTHORS Thongpan,I., Mauleekoonphairoj,J., Vichiwattana,P., Korkong,S.,
Vongpunsawad,S. and Poovorawan,Y.
TITLE Molecular Characterization of Respiratory Syncytial Virus in
Thailand, 2012-2015
JOURNAL Unpublished
REFERENCE 2 (bases 1 to 603)
AUTHORS Thongpan,I., Mauleekoonphairoj,J., Vichiwattana,P., Korkong,S.,
Vongpunsawad,S. and Poovorawan,Y.
TITLE Direct Submission
JOURNAL Submitted (13-DEC-2016) Department of Pediatrics, Center of
Excellence in Clinical Virology, Faculty of Medicine, Chulalongkorn
University, Bangkok 10330, Thailand
COMMENT ##Assembly-Data-START##
Assembly Method :: DNASTAR-Lasergene v. 6
Sequencing Technology :: Sanger dideoxy sequencing
##Assembly-Data-END##
FEATURES Location/Qualifiers
source 1..603
/organism="Human respiratory syncytial virus B"
/mol_type="viral cRNA"
/isolate="TH-CU427/2012"
/host="Homo sapiens"
/db_xref="taxon:208895"
/country="Thailand"
/collection_date="01-Aug-2012"
/note="group: B"
CDS <1..603
/note="G protein"
/codon_start=1

/product="attachment glycoprotein"
/protein_id="APY20444"
/translation="IHTNSATISPNTKSETHHTTAQTKGRTSTPTQNNKPSTKPRPKN
PPKKDDYHFEVFNFVPCSICGNNQLCKSICKTIPSNKPKKKPTTKPTNKPPTKTTNKR
DPKTLAKTPKKETTINPTKKPTPKTTERDTSTPQSTVLDTTTSKHTERDTSTSQSIAL
DTTTSKHTTQQQSLYSTTPENTPNSTQTPTASEPSTSNST"

ORIGIN
```
        1 atccacacaa actcagccac aatatcacct aatacaaaat cagaaacaca ccatacaaca
       61 gcacaaacca aaggcagaac ctctactcca acacagaaca acaagccaag cacaaaacca
      121 cgtccaaaaa atccaccaaa aaaagatgat taccattttg aagtgttcaa ctttgttccc
      181 tgtagtatat gtggcaacaa tcaactctgc aaatccattt gcaaaacaat accaagcaat
      241 aaaccaaaga aaaaaccaac tacaaaaccc acaaacaaac cacctaccaa aaccacaaac
      301 aaaagagacc ccaaaacact agccaaaaca ccgaaaaaag aaaccaccat taacccaaca
      361 aaaaaaccaa cccccaagac cacagaaaga gacaccagca ccccacaatc cactgtgctc
      421 gacacaacca catcaaaaca cacagaaaga gacaccagca cctcacaatc cattgcgctt
      481 gacacaacca catcaaaaca cacaacccaa cagcaatctc tctactcaac cacccccgaa
      541 aacacaccca actccacaca aacacccaca gcatccgagc cctccacatc aaattctacc
      601 taa
//
```

LOCUS KY328104 603 bp cRNA linear VRL 13-DEC-2016
DEFINITION Human respiratory syncytial virus B isolate TH-CU483/2012
attachment glycoprotein gene, partial cds.
ACCESSION KY328104
VERSION KY328104
KEYWORDS .
SOURCE Human respiratory syncytial virus B
ORGANISM Human respiratory syncytial virus B
Viruses; ssRNA viruses; ssRNA negative-strand viruses;
Mononegavirales; Pneumoviridae; Orthopneumovirus.
REFERENCE 1 (bases 1 to 603)
AUTHORS Thongpan,I., Mauleekoonphairoj,J., Vichiwattana,P., Korkong,S.,
Vongpunsawad,S. and Poovorawan,Y.
TITLE Molecular Characterization of Respiratory Syncytial Virus in
Thailand, 2012-2015
JOURNAL Unpublished
REFERENCE 2 (bases 1 to 603)
AUTHORS Thongpan,I., Mauleekoonphairoj,J., Vichiwattana,P., Korkong,S.,
Vongpunsawad,S. and Poovorawan,Y.
TITLE Direct Submission
JOURNAL Submitted (13-DEC-2016) Department of Pediatrics, Center of
Excellence in Clinical Virology, Faculty of Medicine, Chulalongkorn
University, Bangkok 10330, Thailand
COMMENT ##Assembly-Data-START##
Assembly Method :: DNASTAR-Lasergene v. 6
Sequencing Technology :: Sanger dideoxy sequencing
##Assembly-Data-END##
FEATURES Location/Qualifiers
source 1..603
/organism="Human respiratory syncytial virus B"
/mol_type="viral cRNA"
/isolate="TH-CU483/2012"
/host="Homo sapiens"
/db_xref="taxon:208895"
/country="Thailand"

```
                     /collection_date="01-Oct-2012"
                     /note="group: B"
     CDS             <1..603
                     /note="G protein"
                     /codon_start=1
                     /product="attachment glycoprotein"
                     /protein_id="APY20445"
                     /translation="IHTNSATISPNTKSETHHTTAQTKGRTSTPTQNNKPSTKPRPKN
                     PPKKDDYHFEVFNFVPCSICGNNQLCKSICKTIPSNKPKKKPTTKPTNKPPTKTTNKR
                     DPKTLAKTPKKETTINPTKKPTPKTTERDTSTPQSTVLDTTTSKHTERDTSTSQSIAL
                     DTTTSKHTTQQQSLYSTTPENTPNSTQTPTASEPSTSNST"
ORIGIN
        1 atccacacaa actcagccac aatatcacct aatacaaaat cagaaacaca ccatacaaca
       61 gcacaaacca aaggcagaac ctctactcca acacagaaca acaagccaag cacaaaacca
      121 cgtccaaaaa atccaccaaa aaaagatgat taccattttg aagtgttcaa ctttgttccc
      181 tgtagtatat gtggcaacaa tcaactctgc aaatccattt gcaaaacaat accaagcaat
      241 aaaccaaaga aaaaaccaac tacaaaaccc acaaacaaac cacctaccaa aaccacaaac
      301 aaaagagacc ccaaaacact agccaaaaca ccgaaaaaag aaaccaccat taacccaaca
      361 aaaaaaccaa cccccaagac cacagaaaga gacaccagca ccccacaatc cactgtgctc
      421 gacacaacca catcaaaaca cacagaaaga gacaccagca cctcacaatc tattgcgctt
      481 gacacaacca catcaaaaca cacaacccaa cagcaatctc tctactcaac cacccccgaa
      541 aacacaccca actccacaca aacacccaca gcatccgagc cctccacatc aaattctacc
      601 taa
//
LOCUS       KY328105                 603 bp    cRNA    linear   VRL 13-DEC-2016
DEFINITION  Human respiratory syncytial virus B isolate TH-CU464/2012
            attachment glycoprotein gene, partial cds.
ACCESSION   KY328105
VERSION     KY328105
KEYWORDS    .
SOURCE      Human respiratory syncytial virus B
  ORGANISM  Human respiratory syncytial virus B
            Viruses; ssRNA viruses; ssRNA negative-strand viruses;
            Mononegavirales; Pneumoviridae; Orthopneumovirus.
REFERENCE   1  (bases 1 to 603)
  AUTHORS   Thongpan,I., Mauleekoonphairoj,J., Vichiwattana,P., Korkong,S.,
            Vongpunsawad,S. and Poovorawan,Y.
  TITLE     Molecular Characterization of Respiratory Syncytial Virus in
            Thailand, 2012-2015
  JOURNAL   Unpublished
REFERENCE   2  (bases 1 to 603)
  AUTHORS   Thongpan,I., Mauleekoonphairoj,J., Vichiwattana,P., Korkong,S.,
            Vongpunsawad,S. and Poovorawan,Y.
  TITLE     Direct Submission
  JOURNAL   Submitted (13-DEC-2016) Department of Pediatrics, Center of
            Excellence in Clinical Virology, Faculty of Medicine, Chulalongkorn
            University, Bangkok 10330, Thailand
COMMENT     ##Assembly-Data-START##
            Assembly Method       :: DNASTAR-Lasergene v. 6
            Sequencing Technology :: Sanger dideoxy sequencing
            ##Assembly-Data-END##
FEATURES             Location/Qualifiers
     source          1..603
                     /organism="Human respiratory syncytial virus B"
```

```
                     /mol_type="viral cRNA"
                     /isolate="TH-CU464/2012"
                     /host="Homo sapiens"
                     /db_xref="taxon:208895"
                     /country="Thailand"
                     /collection_date="01-Sep-2012"
                     /note="group: B"
     CDS             <1..603
                     /note="G protein"
                     /codon_start=1
                     /product="attachment glycoprotein"
                     /protein_id="APY20446"
                     /translation="IHTNSATISPNTKSETHHTTAQTKGRTSTPTQNNKPSTKPRPKN
                     PPKKDDYHFEVFNFVPCSICGNNQLCKSICKTIPSNKPKKKPTTKPTNKPPTKTTNKR
                     DPKTLAKTPKKETTINPTKKPTPKTTERDTSTPQSTVLDTTTSKHTERDTSTSQSIAL
                     DTTTSKHTTQQQSLYSTTPENTPNSTQTPTASEPSTSNST"
ORIGIN
        1 atccacacaa actcagccac aatatcacct aatacaaaat cagaaacaca ccatacaaca
       61 gcacaaacca aaggcagaac ctctactcca acacagaaca acaagccaag cacaaaacca
      121 cgtccaaaaa atccaccaaa aaaagatgat taccattttg aagtgttcaa ctttgttccc
      181 tgtagtatat gtggcaacaa tcaactctgc aaatccattt gcaaaacaat accaagcaat
      241 aaaccaaaga aaaaaccaac tacaaaaccc acaaacaaac cacctaccaa aaccacaaac
      301 aaaagagacc ccaaaacact agccaaaaca ccgaaaaaag aaaccaccat taacccaaca
      361 aaaaaaccaa cccccaagac cacagaaaga gacaccagca ccccacaatc cactgtgctc
      421 gacacaacca catcaaaaca cacagaaaga gacaccagca cctcacaatc tattgcgctt
      481 gacacaacca catcaaaaca cacaacccaa cagcaatctc tctactcaac cacccccgaa
      541 aacacaccca actccacaca aacacccaca gcatccgagc cctccacatc aaattctacc
      601 taa
//
LOCUS       KY328106                 600 bp    cRNA    linear   VRL 13-DEC-2016
DEFINITION  Human respiratory syncytial virus B isolate TH-CU474/2012
            attachment glycoprotein gene, partial cds.
ACCESSION   KY328106
VERSION     KY328106
KEYWORDS    .
SOURCE      Human respiratory syncytial virus B
  ORGANISM  Human respiratory syncytial virus B
            Viruses; ssRNA viruses; ssRNA negative-strand viruses;
            Mononegavirales; Pneumoviridae; Orthopneumovirus.
REFERENCE   1  (bases 1 to 600)
  AUTHORS   Thongpan,I., Mauleekoonphairoj,J., Vichiwattana,P., Korkong,S.,
            Vongpunsawad,S. and Poovorawan,Y.
  TITLE     Molecular Characterization of Respiratory Syncytial Virus in
            Thailand, 2012-2015
  JOURNAL   Unpublished
REFERENCE   2  (bases 1 to 600)
  AUTHORS   Thongpan,I., Mauleekoonphairoj,J., Vichiwattana,P., Korkong,S.,
            Vongpunsawad,S. and Poovorawan,Y.
  TITLE     Direct Submission
  JOURNAL   Submitted (13-DEC-2016) Department of Pediatrics, Center of
            Excellence in Clinical Virology, Faculty of Medicine, Chulalongkorn
            University, Bangkok 10330, Thailand
COMMENT     ##Assembly-Data-START##
            Assembly Method       :: DNASTAR-Lasergene v. 6
```

Sequencing Technology :: Sanger dideoxy sequencing
##Assembly-Data-END##
FEATURES Location/Qualifiers
source 1..600
/organism="Human respiratory syncytial virus B"
/mol_type="viral cRNA"
/isolate="TH-CU474/2012"
/host="Homo sapiens"
/db_xref="taxon:208895"
/country="Thailand"
/collection_date="01-Oct-2012"
/note="group: B"
CDS <1..600
/note="G protein"
/codon_start=1
/product="attachment glycoprotein"
/protein_id="APY20447"
/translation="HTNSATISPNTKSETHHTTAQTKGRTSTPTQNNKPSTKPRPKNP
PKKDDYHFEVFNFVPCSICGNNQLCKSICKTIPSNKPKKKPTTKPTNKPPTKTTNKRD
PKTLAKTPKKETTINPTKKPTPKTTERDTSTPQSTVLDTTTSKHTERDTSTSQSIALD
TTTSKHTTQQQSLYSTTPENTPNSTQTPTASEPSTSNST"
ORIGIN
1 cacacaaact cagccacaat atcacctaat acaaaatcag aaacacacca tacaacagca
61 caaaccaaag gcagaacctc tactccaaca cagaacaaca agccaagcac aaaaccacgt
121 ccaaaaaatc caccaaaaaa agatgattac cattttgaag tgttcaactt tgttccctgt
181 agtatatgtg gcaacaatca actctgcaaa tccatttgca aaacaatacc aagcaataaa
241 ccaaagaaaa aaccaactac aaaacccaca aacaaaccac ctaccaaaac cacaaacaaa
301 agagacccca aaacactagc caaaacaccg aaaaaagaaa ccaccattaa cccaacaaaa
361 aaaccaaccc ccaagaccac agaaagagac accagcaccc cacaatccac tgtgctcgac
421 acaaccacat caaaacacac agaaagagac accagcacct cacaatccat tgcgcttgac
481 acaaccacat caaaacacac aacccaacag caatctctct actcaaccac ccccgaaaac
541 acacccaact ccacacaaac acccacagca tccgagccct ccacatcaaa ttccacctaa
//
LOCUS KY328107 603 bp cRNA linear VRL 13-DEC-2016
DEFINITION Human respiratory syncytial virus B isolate TH-CU/C4157/2013
attachment glycoprotein gene, partial cds.
ACCESSION KY328107
VERSION KY328107
KEYWORDS .
SOURCE Human respiratory syncytial virus B
ORGANISM Human respiratory syncytial virus B
Viruses; ssRNA viruses; ssRNA negative-strand viruses;
Mononegavirales; Pneumoviridae; Orthopneumovirus.
REFERENCE 1 (bases 1 to 603)
AUTHORS Thongpan,I., Mauleekoonphairoj,J., Vichiwattana,P., Korkong,S.,
Vongpunsawad,S. and Poovorawan,Y.
TITLE Molecular Characterization of Respiratory Syncytial Virus in
Thailand, 2012-2015
JOURNAL Unpublished
REFERENCE 2 (bases 1 to 603)
AUTHORS Thongpan,I., Mauleekoonphairoj,J., Vichiwattana,P., Korkong,S.,
Vongpunsawad,S. and Poovorawan,Y.
TITLE Direct Submission
JOURNAL Submitted (13-DEC-2016) Department of Pediatrics, Center of

Excellence in Clinical Virology, Faculty of Medicine, Chulalongkorn
University, Bangkok 10330, Thailand
COMMENT ##Assembly-Data-START##
Assembly Method :: DNASTAR-Lasergene v. 6
Sequencing Technology :: Sanger dideoxy sequencing
##Assembly-Data-END##
FEATURES Location/Qualifiers
source 1..603
/organism="Human respiratory syncytial virus B"
/mol_type="viral cRNA"
/isolate="TH-CU/C4157/2013"
/host="Homo sapiens"
/db_xref="taxon:208895"
/country="Thailand"
/collection_date="01-Oct-2013"
/note="group: B"
CDS <1..603
/note="G protein"
/codon_start=1
/product="attachment glycoprotein"
/protein_id="APY20448"
/translation="IHTNSATISPNTKSETHHTTAQTKGRTSTPTQNNKPSTKPRPKN
PPKKDDYHFEVFNFVPCSICGNNQLCKSICKTIPSNKPKKKPTTKPTNKPPTKTTNKR
DPKTLAKTPKKETTINPTKKPTPKTTERDTSTPQSTVLDTTTSKHTERDTSTSQSIAL
DTTTSKHTTQQQSLYSTTPENTPNSTQTPTASEPSTSNST"
ORIGIN
1 atccacacaa actcagccac aatatcacct aatacaaaat cagaaacaca ccatacaaca
61 gcacaaacca aaggcagaac ctctactcca acacagaaca acaagccaag cacaaaacca
121 cgtccaaaaa atccaccaaa aaaagatgat taccattttg aagtgttcaa cttcgttccc
181 tgtagtatat gtggcaacaa tcaactctgc aaatccattt gcaaaacaat accaagcaat
241 aaaccaaaga aaaaaccaac cacaaaaccc acaaacaaac cacctaccaa aaccacaaac
301 aaaagagacc ccaaaacact agccaaaaca ccgaaaaaag aaaccaccat taacccaaca
361 aaaaaaccaa cccccaagac cacagaaaga gacaccagca ccccacaatc cactgtgctc
421 gacacaacca catcaaaaca cacagaaaga gacaccagca cctcacaatc cattgcgctt
481 gacacaacca catcaaaaca cacaacccaa cagcaatctc tctactcaac cacccccgaa
541 aacacaccca actccacaca aacacccaca gcatccgagc cctccacatc aaattccacc
601 taa
//
LOCUS KY328108 603 bp cRNA linear VRL 13-DEC-2016
DEFINITION Human respiratory syncytial virus B isolate TH-CU/CB105/2013
attachment glycoprotein gene, partial cds.
ACCESSION KY328108
VERSION KY328108
KEYWORDS .
SOURCE Human respiratory syncytial virus B
ORGANISM Human respiratory syncytial virus B
Viruses; ssRNA viruses; ssRNA negative-strand viruses;
Mononegavirales; Pneumoviridae; Orthopneumovirus.
REFERENCE 1 (bases 1 to 603)
AUTHORS Thongpan,I., Mauleekoonphairoj,J., Vichiwattana,P., Korkong,S.,
Vongpunsawad,S. and Poovorawan,Y.
TITLE Molecular Characterization of Respiratory Syncytial Virus in
Thailand, 2012-2015
JOURNAL Unpublished

```
REFERENCE   2  (bases 1 to 603)
  AUTHORS   Thongpan,I., Mauleekoonphairoj,J., Vichiwattana,P., Korkong,S.,
            Vongpunsawad,S. and Poovorawan,Y.
  TITLE     Direct Submission
  JOURNAL   Submitted (13-DEC-2016) Department of Pediatrics, Center of
            Excellence in Clinical Virology, Faculty of Medicine, Chulalongkorn
            University, Bangkok 10330, Thailand
COMMENT     ##Assembly-Data-START##
            Assembly Method       :: DNASTAR-Lasergene v. 6
            Sequencing Technology :: Sanger dideoxy sequencing
            ##Assembly-Data-END##
FEATURES             Location/Qualifiers
     source          1..603
                     /organism="Human respiratory syncytial virus B"
                     /mol_type="viral cRNA"
                     /isolate="TH-CU/CB105/2013"
                     /host="Homo sapiens"
                     /db_xref="taxon:208895"
                     /country="Thailand"
                     /collection_date="01-Oct-2013"
                     /note="group: B"
     CDS             <1..603
                     /note="G protein"
                     /codon_start=1
                     /product="attachment glycoprotein"
                     /protein_id="APY20449"
                     /translation="IHTNSATISPNTKSETHHTTAQTKGRTFTPTQNNKPSTKPRPKN
                     PPKKDDYHFEVFNFVPCSICGNNQLCKSICKTIPSNKPKKKPTTKPTNKPPTKTTNKR
                     DPKTLAKTPKKETTINPTKKPTPKTTERDTSTPQSTVLDTTTSKHTERDTSTSQSIAL
                     DTTTSKHTTQQQSLYSTTPENTPNSTQTPTASEPSTSNST"
ORIGIN
        1 atccacacaa actcagccac aatatcacct aatacaaaat cagaaacaca ccatacaaca
       61 gcacaaacca aaggcagaac ctttactcca acacagaaca acaagccaag cacaaaacca
      121 cgtccaaaaa atccaccaaa aaaagatgat taccattttg aagtgttcaa cttcgttccc
      181 tgtagtatat gtggcaacaa tcaactctgc aaatccattt gcaaaacaat accaagcaat
      241 aaaccaaaga aaaaaccaac cacaaaaccc acaaacaaac cacctaccaa aaccacaaac
      301 aaaagagacc ccaaaacact agccaaaaca ccgaaaaaag aaaccaccat taacccaaca
      361 aaaaaaccaa cccccaagac cacagaaaga gacaccagca ccccacaatc cactgtgctc
      421 gacacaacca catcaaaaca cacagaaaga gacaccagca cctcacaatc cattgcgctt
      481 gacacaacca catcaaaaca cacaacccaa cagcaatctc tctactcaac cacccccgaa
      541 aacacaccca actccacaca aacacccaca gcatccgagc cctccacatc aaattccacc
      601 taa
//
LOCUS       KY328109                 603 bp    cRNA    linear   VRL 13-DEC-2016
DEFINITION  Human respiratory syncytial virus B isolate TH-CU/CB125/2013
            attachment glycoprotein gene, partial cds.
ACCESSION   KY328109
VERSION     KY328109
KEYWORDS    .
SOURCE      Human respiratory syncytial virus B
  ORGANISM  Human respiratory syncytial virus B
            Viruses; ssRNA viruses; ssRNA negative-strand viruses;
            Mononegavirales; Pneumoviridae; Orthopneumovirus.
REFERENCE   1  (bases 1 to 603)
```

AUTHORS Thongpan,I., Mauleekoonphairoj,J., Vichiwattana,P., Korkong,S.,
Vongpunsawad,S. and Poovorawan,Y.
TITLE Molecular Characterization of Respiratory Syncytial Virus in
Thailand, 2012-2015
JOURNAL Unpublished
REFERENCE 2 (bases 1 to 603)
AUTHORS Thongpan,I., Mauleekoonphairoj,J., Vichiwattana,P., Korkong,S.,
Vongpunsawad,S. and Poovorawan,Y.
TITLE Direct Submission
JOURNAL Submitted (13-DEC-2016) Department of Pediatrics, Center of
Excellence in Clinical Virology, Faculty of Medicine, Chulalongkorn
University, Bangkok 10330, Thailand
COMMENT ##Assembly-Data-START##
Assembly Method :: DNASTAR-Lasergene v. 6
Sequencing Technology :: Sanger dideoxy sequencing
##Assembly-Data-END##
FEATURES Location/Qualifiers
source 1..603
/organism="Human respiratory syncytial virus B"
/mol_type="viral cRNA"
/isolate="TH-CU/CB125/2013"
/host="Homo sapiens"
/db_xref="taxon:208895"
/country="Thailand"
/collection_date="01-Nov-2013"
/note="group: B"
CDS <1..603
/note="G protein"
/codon_start=1
/product="attachment glycoprotein"
/protein_id="APY20450"
/translation="IHTNSATISPNTKSETHHTTAQTKGRTSTPTQNNKPSTKPRPKN
PPKKDDYHFEVFNFVPCSICGNNQLCKSICKTIPSNKPKKKPTTKPTNKPPTKTTNKR
DPKTLAKTPKKETTINPTKKPTPKTTERDTSTPQSTVLDTTTSKHTERDTSTSQSTAL
DTTTSKHTTQQQSLHSTTPENTPNSTQTPTASEPSTSNST"
ORIGIN
1 atccacacaa actcagccac aatatcacct aatacaaaat cagaaacaca ccatacaaca
61 gcacaaacca aaggcagaac ctctactcca acacagaaca acaagccaag cacaaaacca
121 cgtccaaaaa atccaccaaa aaaagatgat taccattttg aagtgttcaa cttcgttccc
181 tgtagtatat gtggcaacaa tcaactctgc aaatccattt gcaaaacaat accaagcaat
241 aaaccaaaga aaaaaccaac tacaaaaccc acaaacaaac cacctaccaa aaccacaaac
301 aaaagagacc ccaaaacact agccaaaaca ccgaaaaaag aaaccaccat taacccaaca
361 aaaaaaccaa cccccaagac cacagaaaga gacaccagca ccccacaatc cactgtgctc
421 gacacaacca catcaaaaca cacagaaaga gacaccagca cctcacaatc cactgcgctt
481 gacacaacca catcaaaaca cacaacccaa cagcaatctc tccactcaac cacccccgaa
541 aacacaccca actccacaca aacacccaca gcatccgagc cctccacatc aaattccacc
601 taa
//
LOCUS KY328110 603 bp cRNA linear VRL 13-DEC-2016
DEFINITION Human respiratory syncytial virus B isolate TH-CU/CB112/2013
attachment glycoprotein gene, partial cds.
ACCESSION KY328110
VERSION KY328110
KEYWORDS .

SOURCE Human respiratory syncytial virus B
ORGANISM Human respiratory syncytial virus B
Viruses; ssRNA viruses; ssRNA negative-strand viruses;
Mononegavirales; Pneumoviridae; Orthopneumovirus.
REFERENCE 1 (bases 1 to 603)
AUTHORS Thongpan,I., Mauleekoonphairoj,J., Vichiwattana,P., Korkong,S.,
Vongpunsawad,S. and Poovorawan,Y.
TITLE Molecular Characterization of Respiratory Syncytial Virus in
Thailand, 2012-2015
JOURNAL Unpublished
REFERENCE 2 (bases 1 to 603)
AUTHORS Thongpan,I., Mauleekoonphairoj,J., Vichiwattana,P., Korkong,S.,
Vongpunsawad,S. and Poovorawan,Y.
TITLE Direct Submission
JOURNAL Submitted (13-DEC-2016) Department of Pediatrics, Center of
Excellence in Clinical Virology, Faculty of Medicine, Chulalongkorn
University, Bangkok 10330, Thailand
COMMENT ##Assembly-Data-START##
Assembly Method :: DNASTAR-Lasergene v. 6
Sequencing Technology :: Sanger dideoxy sequencing
##Assembly-Data-END##
FEATURES Location/Qualifiers
source 1..603
/organism="Human respiratory syncytial virus B"
/mol_type="viral cRNA"
/isolate="TH-CU/CB112/2013"
/host="Homo sapiens"
/db_xref="taxon:208895"
/country="Thailand"
/collection_date="01-Nov-2013"
/note="group: B"
CDS <1..603
/note="G protein"
/codon_start=1
/product="attachment glycoprotein"
/protein_id="APY20451"
/translation="IYTNSATISPNTKSETHHTTAQTKGRTSTPTQNNKPSTKPRPKN
PPKKDDYHFEVFNFVPCSICGNNQLCKSICKTIPSNKPKKKPTTKPTNKPPTKTTNKR
DPKTLAKTPKKETTINPTKKPTPKTTERDTSTPQSTVLDTTTSKHTERDTSTSQSTAL
DTTTSKHTTQQQSLHSTTPENTPNSTQTPTASEPSTSNST"
ORIGIN
1 atctacacaa actcagccac aatatcacct aatacaaaat cagaaacaca ccatacaaca
61 gcacaaacca aaggcagaac ctctactcca acacagaaca acaagccaag cacaaaacca
121 cgtccaaaaa atccaccaaa aaaagatgat taccattttg aagtgttcaa cttcgttccc
181 tgtagtatat gtggcaacaa tcaactctgc aaatccattt gcaaaacaat accaagcaat
241 aaaccaaaga aaaaaccaac tacaaaaccc acaaacaaac cacctaccaa aaccacaaac
301 aaaagagacc ccaaaacact agccaaaaca ccgaaaaaag aaaccaccat taacccaaca
361 aaaaaaccaa cccccaagac cacagaaaga gacaccagca ccccacaatc cactgtgctc
421 gacacaacca catcaaaaca cacagaaaga gacaccagca cctcacaatc cactgcgctt
481 gacacaacca catcaaaaca cacaacccaa cagcaatctc tccactcaac cacccccgaa
541 aacacaccca actccacaca aacacccaca gcatccgagc cctccacatc aaattccacc
601 taa
//
LOCUS KY328111 597 bp cRNA linear VRL 13-DEC-2016

file:///D|/Final%20peerJ/GenBank%20KY327937-KY328148_.txt[3/10/2560 14:59:50]

```
DEFINITION  Human respiratory syncytial virus B isolate TH-CU/CB95/2013
            attachment glycoprotein gene, partial cds.
ACCESSION   KY328111
VERSION     KY328111
KEYWORDS    .
SOURCE      Human respiratory syncytial virus B
  ORGANISM  Human respiratory syncytial virus B
            Viruses; ssRNA viruses; ssRNA negative-strand viruses;
            Mononegavirales; Pneumoviridae; Orthopneumovirus.
REFERENCE   1  (bases 1 to 597)
  AUTHORS   Thongpan,I., Mauleekoonphairoj,J., Vichiwattana,P., Korkong,S.,
            Vongpunsawad,S. and Poovorawan,Y.
  TITLE     Molecular Characterization of Respiratory Syncytial Virus in
            Thailand, 2012-2015
  JOURNAL   Unpublished
REFERENCE   2  (bases 1 to 597)
  AUTHORS   Thongpan,I., Mauleekoonphairoj,J., Vichiwattana,P., Korkong,S.,
            Vongpunsawad,S. and Poovorawan,Y.
  TITLE     Direct Submission
  JOURNAL   Submitted (13-DEC-2016) Department of Pediatrics, Center of
            Excellence in Clinical Virology, Faculty of Medicine, Chulalongkorn
            University, Bangkok 10330, Thailand
COMMENT     ##Assembly-Data-START##
            Assembly Method       :: DNASTAR-Lasergene v. 6
            Sequencing Technology :: Sanger dideoxy sequencing
            ##Assembly-Data-END##
FEATURES             Location/Qualifiers
     source          1..597
                     /organism="Human respiratory syncytial virus B"
                     /mol_type="viral cRNA"
                     /isolate="TH-CU/CB95/2013"
                     /host="Homo sapiens"
                     /db_xref="taxon:208895"
                     /country="Thailand"
                     /collection_date="01-Sep-2013"
                     /note="group: B"
     CDS             <1..597
                     /note="G protein"
                     /codon_start=1
                     /product="attachment glycoprotein"
                     /protein_id="APY20452"
                     /translation="TNSATISPNTKSETHHTTAQTKGRTSTPTQNNKPSTKPRPKNPP
                     KKDDYHFEVFNFVPCSICGNNQLCKSICKTIPSNKPKKKPTTKPTNKPPTKTTNKRDP
                     KTLAKTPKKETTINPTKKPTPKTTERDTSTPQSTVLDTTTSKHTERDTSTSQSTALDT
                     TTSKHTTQQQSLHSTTPENTPNSTQTPTASEPSTSNST"
ORIGIN
        1 acaaactcag ccacaatatc acctaataca aaatcagaaa cacaccatac aacagcacaa
       61 accaaaggca gaacctctac tccaacacag aacaacaagc caagcacaaa accacgtcca
      121 aaaaatccac caaaaaaaga tgattaccat tttgaagtgt tcaacttcgt tccctgtagt
      181 atatgtggca acaatcaact ctgcaaatcc atctgcaaaa caataccaag caataaacca
      241 aagaaaaaac caactacaaa acccacaaac aaaccaccta ccaaaaccac aaacaaaaga
      301 gaccccaaaa cactagccaa aacaccgaaa aaagaaacca ccattaaccc aacaaaaaaa
      361 ccaaccccca agaccacaga aagagacacc agcacaccac aatccactgt gctcgacaca
      421 accacatcaa aacacacaga aagagacacc agcacctcac aatccactgc gcttgacaca
```

481 accacatcaa aacacacaac ccaacagcaa tctctccact caaccacccc cgaaaacaca
541 cccaactcca cacaaacacc cacagcatcc gagccctcca catcaaattc cacctaa
//
LOCUS KY328112 603 bp cRNA linear VRL 13-DEC-2016
DEFINITION Human respiratory syncytial virus B isolate TH-CU/C3173/2012
attachment glycoprotein gene, partial cds.
ACCESSION KY328112
VERSION KY328112
KEYWORDS .
SOURCE Human respiratory syncytial virus B
ORGANISM Human respiratory syncytial virus B
Viruses; ssRNA viruses; ssRNA negative-strand viruses;
Mononegavirales; Pneumoviridae; Orthopneumovirus.
REFERENCE 1 (bases 1 to 603)
AUTHORS Thongpan,I., Mauleekoonphairoj,J., Vichiwattana,P., Korkong,S.,
Vongpunsawad,S. and Poovorawan,Y.
TITLE Molecular Characterization of Respiratory Syncytial Virus in
Thailand, 2012-2015
JOURNAL Unpublished
REFERENCE 2 (bases 1 to 603)
AUTHORS Thongpan,I., Mauleekoonphairoj,J., Vichiwattana,P., Korkong,S.,
Vongpunsawad,S. and Poovorawan,Y.
TITLE Direct Submission
JOURNAL Submitted (13-DEC-2016) Department of Pediatrics, Center of
Excellence in Clinical Virology, Faculty of Medicine, Chulalongkorn
University, Bangkok 10330, Thailand
COMMENT ##Assembly-Data-START##
Assembly Method :: DNASTAR-Lasergene v. 6
Sequencing Technology :: Sanger dideoxy sequencing
##Assembly-Data-END##
FEATURES Location/Qualifiers
source 1..603
/organism="Human respiratory syncytial virus B"
/mol_type="viral cRNA"
/isolate="TH-CU/C3173/2012"
/host="Homo sapiens"
/db_xref="taxon:208895"
/country="Thailand"
/collection_date="01-Sep-2012"
/note="group: B"
CDS <1..603
/note="G protein"
/codon_start=1
/product="attachment glycoprotein"
/protein_id="APY20453"
/translation="IHTNSATISPNTKSETHHTTAQTKGRTSTPTQNNKPSTKPRPKN
PPKKDDYHFEVFNFVPCSICGNNQLCKSICKTIPSNKPKKKPTTKPTNKPPTKTTNKR
DPKTLAKTPKKETTINPTRKPTPKTTERDTSTPQSSVLDTTTSKHTERDTSTSQSIAL
DTTTSKHTTQQQSLYSTTPENTPNSTQTPTASEPSTSNST"
ORIGIN
1 atccacacaa actcagccac aatatcacct aatacaaaat cagaaacaca ccatacaaca
61 gcacaaacca aaggcagaac ctctactcca acacagaaca acaagccaag cacaaaacca
121 cgtccaaaaa atccaccaaa aaaagatgat taccattttg aagtgttcaa ctttgttccc
181 tgtagtatat gtggcaacaa tcaactctgc aaatccattt gcaaaacaat accaagcaat

```
     241 aaaccaaaga aaaaaccaac tacaaaaccc acaaacaaac cacctaccaa aaccacaaac
     301 aaaagagacc ccaaaacact agccaaaaca ccgaaaaaag aaaccaccat taacccaaca
     361 agaaaaccaa cccccaagac cacagaaaga gacaccagca ccccacaatc ctctgtgctc
     421 gacacaacca catcaaaaca cacagaaaga gacaccagca cctcacaatc cattgcgctt
     481 gacacaacca catcaaaaca cacaacccaa cagcaatctc tctactcaac cacccccgaa
     541 aacacaccca actccacaca aacacccaca gcatccgagc cctccacatc aaattctacc
     601 taa
//
LOCUS       KY328113                 603 bp    cRNA    linear   VRL 13-DEC-2016
DEFINITION  Human respiratory syncytial virus B isolate TH-CU476/2013
            attachment glycoprotein gene, partial cds.
ACCESSION   KY328113
VERSION     KY328113
KEYWORDS    .
SOURCE      Human respiratory syncytial virus B
  ORGANISM  Human respiratory syncytial virus B
            Viruses; ssRNA viruses; ssRNA negative-strand viruses;
            Mononegavirales; Pneumoviridae; Orthopneumovirus.
REFERENCE   1  (bases 1 to 603)
  AUTHORS   Thongpan,I., Mauleekoonphairoj,J., Vichiwattana,P., Korkong,S.,
            Vongpunsawad,S. and Poovorawan,Y.
  TITLE     Molecular Characterization of Respiratory Syncytial Virus in
            Thailand, 2012-2015
  JOURNAL   Unpublished
REFERENCE   2  (bases 1 to 603)
  AUTHORS   Thongpan,I., Mauleekoonphairoj,J., Vichiwattana,P., Korkong,S.,
            Vongpunsawad,S. and Poovorawan,Y.
  TITLE     Direct Submission
  JOURNAL   Submitted (13-DEC-2016) Department of Pediatrics, Center of
            Excellence in Clinical Virology, Faculty of Medicine, Chulalongkorn
            University, Bangkok 10330, Thailand
COMMENT     ##Assembly-Data-START##
            Assembly Method       :: DNASTAR-Lasergene v. 6
            Sequencing Technology :: Sanger dideoxy sequencing
            ##Assembly-Data-END##
FEATURES             Location/Qualifiers
     source          1..603
                     /organism="Human respiratory syncytial virus B"
                     /mol_type="viral cRNA"
                     /isolate="TH-CU476/2013"
                     /host="Homo sapiens"
                     /db_xref="taxon:208895"
                     /country="Thailand"
                     /collection_date="01-Oct-2012"
                     /note="group: B"
     CDS             <1..603
                     /note="G protein"
                     /codon_start=1
                     /product="attachment glycoprotein"
                     /protein_id="APY20454"
                     /translation="IHTNSATISPNTKSETHHTTAQTKGRTSTPTQNNKPSTKPRPKN
                     PPKKDDYHFEVFNFVPCSICGNNQLCKSICKTIPSNKPKKKPTTKPTNKPPTKTTNKR
                     DPKTLAKTPKKETTINPTKKPTPKTTERDTSTPHSTVLDTTTSKHTERDTSTSQSTAL
                     DTTTSKHTTQQQSLYSTTPENTPNSTQTPTASEPSTSNST"
```

```
ORIGIN
        1 atccacacaa actcagccac aatatcacct aatacaaaat cagaaacaca ccatacaaca
       61 gcacaaacca aaggcagaac ctctactcca acacagaaca acaagccaag cacaaaacca
      121 cgtccaaaaa atccaccaaa aaaagatgat taccattttg aagtgttcaa ctttgttccc
      181 tgtagtatat gtggcaacaa tcaactctgc aaatccattt gcaaaacaat accaagcaat
      241 aaaccaaaga aaaaaccaac tacaaaaccc acaaacaaac cacctaccaa aaccacaaac
      301 aaaagagacc ccaaaacact agccaaaaca ccgaaaaaag aaaccaccat taacccaaca
      361 aaaaaaccaa cccccaagac aacagaaaga gacaccagca ccccacactc cactgtgctc
      421 gacacaacca catcaaaaca cacagaaaga gacaccagca cctcacaatc cactgcgctt
      481 gacacaacca catcaaaaca cacaacccaa cagcaatctc tctactcaac cacccccgaa
      541 aacacaccca actccacaca aacacccaca gcatccgagc cctccacatc aaattccacc
      601 taa
//
LOCUS       KY328114                 603 bp    cRNA    linear   VRL 13-DEC-2016
DEFINITION  Human respiratory syncytial virus B isolate TH-CU/CB126/2013
            attachment glycoprotein gene, partial cds.
ACCESSION   KY328114
VERSION     KY328114
KEYWORDS    .
SOURCE      Human respiratory syncytial virus B
  ORGANISM  Human respiratory syncytial virus B
            Viruses; ssRNA viruses; ssRNA negative-strand viruses;
            Mononegavirales; Pneumoviridae; Orthopneumovirus.
REFERENCE   1  (bases 1 to 603)
  AUTHORS   Thongpan,I., Mauleekoonphairoj,J., Vichiwattana,P., Korkong,S.,
            Vongpunsawad,S. and Poovorawan,Y.
  TITLE     Molecular Characterization of Respiratory Syncytial Virus in
            Thailand, 2012-2015
  JOURNAL   Unpublished
REFERENCE   2  (bases 1 to 603)
  AUTHORS   Thongpan,I., Mauleekoonphairoj,J., Vichiwattana,P., Korkong,S.,
            Vongpunsawad,S. and Poovorawan,Y.
  TITLE     Direct Submission
  JOURNAL   Submitted (13-DEC-2016) Department of Pediatrics, Center of
            Excellence in Clinical Virology, Faculty of Medicine, Chulalongkorn
            University, Bangkok 10330, Thailand
COMMENT     ##Assembly-Data-START##
            Assembly Method       :: DNASTAR-Lasergene v. 6
            Sequencing Technology :: Sanger dideoxy sequencing
            ##Assembly-Data-END##
FEATURES             Location/Qualifiers
     source          1..603
                     /organism="Human respiratory syncytial virus B"
                     /mol_type="viral cRNA"
                     /isolate="TH-CU/CB126/2013"
                     /host="Homo sapiens"
                     /db_xref="taxon:208895"
                     /country="Thailand"
                     /collection_date="01-Nov-2013"
                     /note="group: B"
     CDS             <1..603
                     /note="G protein"
                     /codon_start=1
                     /product="attachment glycoprotein"
```

/protein_id="APY20455"
/translation="IHTNSATISPNTKSETHHTTAQTKGRTSTPTQNNKPSTKPRPKN
PPKKDDYHFEVFNFVPCSICGNNQLCKSICKTIPSNKPKKKPTTKPTNKPPTKTTNKS
DPKTLAKTPKKETTINPTKKPTPKTTERDTSTPQSTVLDTTTSKHTERDTSTPQSTAL
DTTTSKHTTQQQSLHSTTPENTPNSTQTPTASEPSTSNST"
ORIGIN
1 atccacacaa actcagccac aatatcacct aatacaaaat cagaaacaca ccatacaaca
61 gcacaaacca aaggcagaac ctctactcca acacagaaca acaagccaag cacaaaacca
121 cgtccaaaaa atccaccaaa aaaagatgat taccattttg aagtgttcaa cttcgttccc
181 tgtagtatat gtggcaacaa tcaactctgc aaatccatct gcaaaacaat accaagcaat
241 aaaccaaaga aaaaaccaac tacaaaaccc acaaacaaac cacctaccaa aaccacaaac
301 aaaagtgacc ccaaaacact agccaaaaca ccgaaaaaag aaaccaccat taacccaaca
361 aaaaaaccaa cccccaagac cacagaaaga gacaccagca ccccacaatc cactgtgctc
421 gacacaacca catcaaaaca cacagaaaga gacaccagca ccccacaatc cactgcactt
481 gacacaacca catcaaaaca cacaacccaa cagcaatctc tccactcaac cacccccgaa
541 aacacaccca actccacaca aacacccaca gcatccgagc cctccacatc aaattccacc
601 taa
//
LOCUS KY328115 603 bp cRNA linear VRL 13-DEC-2016
DEFINITION Human respiratory syncytial virus B isolate TH-CU/B10596/2014
attachment glycoprotein gene, partial cds.
ACCESSION KY328115
VERSION KY328115
KEYWORDS .
SOURCE Human respiratory syncytial virus B
ORGANISM Human respiratory syncytial virus B
Viruses; ssRNA viruses; ssRNA negative-strand viruses;
Mononegavirales; Pneumoviridae; Orthopneumovirus.
REFERENCE 1 (bases 1 to 603)
AUTHORS Thongpan,I., Mauleekoonphairoj,J., Vichiwattana,P., Korkong,S.,
Vongpunsawad,S. and Poovorawan,Y.
TITLE Molecular Characterization of Respiratory Syncytial Virus in
Thailand, 2012-2015
JOURNAL Unpublished
REFERENCE 2 (bases 1 to 603)
AUTHORS Thongpan,I., Mauleekoonphairoj,J., Vichiwattana,P., Korkong,S.,
Vongpunsawad,S. and Poovorawan,Y.
TITLE Direct Submission
JOURNAL Submitted (13-DEC-2016) Department of Pediatrics, Center of
Excellence in Clinical Virology, Faculty of Medicine, Chulalongkorn
University, Bangkok 10330, Thailand
COMMENT ##Assembly-Data-START##
Assembly Method :: DNASTAR-Lasergene v. 6
Sequencing Technology :: Sanger dideoxy sequencing
##Assembly-Data-END##
FEATURES Location/Qualifiers
source 1..603
/organism="Human respiratory syncytial virus B"
/mol_type="viral cRNA"
/isolate="TH-CU/B10596/2014"
/host="Homo sapiens"
/db_xref="taxon:208895"
/country="Thailand"
/collection_date="01-Aug-2014"

```
                     /note="group: B"
     CDS             <1..603
                     /note="G protein"
                     /codon_start=1
                     /product="attachment glycoprotein"
                     /protein_id="APY20456"
                     /translation="IHTNLATISPNTKSETHHTTAQTKGRTSTPTQKNKPSTKPRPKN
                     PPKKDDYHFEVFNFVPCSICGNNQLCKSICKTIPSNKPKKKPTTKPTNKPPTKTTNKR
                     DPKTLAKTPKKETTINPTKKPTPKTTERDTSTPQSTVLDTTTSKHTERDTSTSQSIAL
                     DTTTSKHTTQQQSLYSTTPENTPNSTQTPTASEPSTSNST"
ORIGIN
        1 atccacacaa acttagccac aatatcacct aatacaaaat cagaaacaca ccatacaaca
       61 gcacaaacca aaggcagaac ctctactcca acacagaaga acaagccaag cacaaaacca
      121 cgtccaaaaa atccaccaaa aaaagatgat taccattttg aagtgttcaa cttcgttccc
      181 tgtagtatat gtggcaacaa tcaactctgc aaatccattt gcaaaacaat accaagcaat
      241 aaaccaaaga aaaaaccaac tacaaaaccc acaaacaaac cacctaccaa aaccacaaac
      301 aaaagagacc ccaaaacact agccaaaaca ccgaaaaaag aaaccaccat taacccaaca
      361 aaaaaaccaa cccccaagac cacagaaaga gacaccagca ccccacaatc cactgtgctc
      421 gacacaacca catcaaaaca cacagaaaga gacaccagca cctcacaatc cattgcgctt
      481 gacacaacca catcaaaaca cacaacccaa cagcaatctc tctactcaac cacccccgaa
      541 aacacaccca actccacaca aacacccaca gcatccgagc cctccacatc aaactccacc
      601 taa
//
LOCUS       KY328116                 624 bp    cRNA    linear   VRL 13-DEC-2016
DEFINITION  Human respiratory syncytial virus B isolate TH-CU/B10598/2014
            attachment glycoprotein gene, partial cds.
ACCESSION   KY328116
VERSION     KY328116
KEYWORDS    .
SOURCE      Human respiratory syncytial virus B
  ORGANISM  Human respiratory syncytial virus B
            Viruses; ssRNA viruses; ssRNA negative-strand viruses;
            Mononegavirales; Pneumoviridae; Orthopneumovirus.
REFERENCE   1  (bases 1 to 624)
  AUTHORS   Thongpan,I., Mauleekoonphairoj,J., Vichiwattana,P., Korkong,S.,
            Vongpunsawad,S. and Poovorawan,Y.
  TITLE     Molecular Characterization of Respiratory Syncytial Virus in
            Thailand, 2012-2015
  JOURNAL   Unpublished
REFERENCE   2  (bases 1 to 624)
  AUTHORS   Thongpan,I., Mauleekoonphairoj,J., Vichiwattana,P., Korkong,S.,
            Vongpunsawad,S. and Poovorawan,Y.
  TITLE     Direct Submission
  JOURNAL   Submitted (13-DEC-2016) Department of Pediatrics, Center of
            Excellence in Clinical Virology, Faculty of Medicine, Chulalongkorn
            University, Bangkok 10330, Thailand
COMMENT     ##Assembly-Data-START##
            Assembly Method       :: DNASTAR-Lasergene v. 6
            Sequencing Technology :: Sanger dideoxy sequencing
            ##Assembly-Data-END##
FEATURES             Location/Qualifiers
     source          1..624
                     /organism="Human respiratory syncytial virus B"
                     /mol_type="viral cRNA"
```

/isolate="TH-CU/B10598/2014"
/host="Homo sapiens"
/db_xref="taxon:208895"
/country="Thailand"
/collection_date="02-Aug-2014"
/note="group: B"
CDS <1..624
/note="G protein"
/codon_start=1
/product="attachment glycoprotein"
/protein_id="APY20457"
/translation="IHTNLATISPNTKSETHHTTAQTKGRTSTPTQKNKPSTKPRPKN
PPKKDDYHFEVFNFVPCSICGNNQLCKSICKTIPSNKPKKKPTTKPTNKPPTKTTNKR
DPKTLAKTPKKETTINPTKKPTPKTTERDTSTPQSTVLDTTTSKHTERDTSTSQSIAL
DTTTSKHTTQQQSLYSTTPENTPNSTQTPTASEPSTSNSTQKLQSYA"
ORIGIN
1 atccacacaa acttagccac aatatcacct aatacaaaat cagaaacaca ccatacaaca
61 gcacaaacca aaggcagaac ctctactcca acacagaaga acaagccaag cacaaaacca
121 cgtccaaaaa atccaccaaa aaaagatgat taccattttg aagtgttcaa cttcgttccc
181 tgtagtatat gtggcaacaa tcaactctgc aaatccattt gcaaaacaat accaagcaat
241 aaaccaaaga aaaaaccaac tacaaaaccc acaaacaaac cacctaccaa aaccacaaac
301 aaaagagacc ccaaaacact agccaaaaca ccgaaaaaag aaaccaccat taacccaaca
361 aaaaaaccaa cccccaagac cacagaaaga gacaccagca ccccacaatc cactgtgctc
421 gacacaacca catcaaaaca cacagaaaga gacaccagca cctcacaatc cattgcgctt
481 gacacaacca catcaaaaca cacaacccaa cagcaatctc tctactcaac cacccccgaa
541 aacacaccca actccacaca aacacccaca gcatccgagc cctccacatc aaactccacc
601 caaaaactcc agtcatatgc ttag
//
LOCUS KY328117 603 bp cRNA linear VRL 13-DEC-2016
DEFINITION Human respiratory syncytial virus B isolate TH-CU/C4051/2013
attachment glycoprotein gene, partial cds.
ACCESSION KY328117
VERSION KY328117
KEYWORDS .
SOURCE Human respiratory syncytial virus B
ORGANISM Human respiratory syncytial virus B
Viruses; ssRNA viruses; ssRNA negative-strand viruses;
Mononegavirales; Pneumoviridae; Orthopneumovirus.
REFERENCE 1 (bases 1 to 603)
AUTHORS Thongpan,I., Mauleekoonphairoj,J., Vichiwattana,P., Korkong,S.,
Vongpunsawad,S. and Poovorawan,Y.
TITLE Molecular Characterization of Respiratory Syncytial Virus in
Thailand, 2012-2015
JOURNAL Unpublished
REFERENCE 2 (bases 1 to 603)
AUTHORS Thongpan,I., Mauleekoonphairoj,J., Vichiwattana,P., Korkong,S.,
Vongpunsawad,S. and Poovorawan,Y.
TITLE Direct Submission
JOURNAL Submitted (13-DEC-2016) Department of Pediatrics, Center of
Excellence in Clinical Virology, Faculty of Medicine, Chulalongkorn
University, Bangkok 10330, Thailand
COMMENT ##Assembly-Data-START##
Assembly Method :: DNASTAR-Lasergene v. 6
Sequencing Technology :: Sanger dideoxy sequencing

##Assembly-Data-END##
FEATURES   Location/Qualifiers
     source   1..603
                     /organism="Human respiratory syncytial virus B"
                     /mol_type="viral cRNA"
                     /isolate="TH-CU/C4051/2013"
                     /host="Homo sapiens"
                     /db_xref="taxon:208895"
                     /country="Thailand"
                     /collection_date="01-Aug-2013"
                     /note="group: B"
     CDS   <1..603
                     /note="G protein"
                     /codon_start=1
                     /product="attachment glycoprotein"
                     /protein_id="APY20458"
                     /translation="IHTNSATISPNTKSETHHTTAQTKGRTSTPTQNNKPSTKPRPKN
                     PPKKDDYHFEVFNFVPCNICGNNQLCKSICKTIPSNKPKKKPTTKPTNKPPTKTTNKR
                     DPKTLAKTPKKETTINPTKKPTPKTTERDTSTPQSTVLDTTTTKHTERDTSTPQSTAL
                     DTTTSKHTTQQQSLHSTTPENTPNSTQTPTAPEPSTSNST"
ORIGIN
        1 atccacacaa actcagccac aatatcacct aatacaaaat cagaaacaca ccatacaaca
       61 gcacaaacca aaggcagaac ctctactcca acacagaaca acaagccaag cacaaaacca
      121 cgtccaaaaa atccaccaaa aaaagatgat taccattttg aagtgttcaa cttcgttccc
      181 tgtaatatat gtggcaacaa tcaactctgc aaatccattt gcaaaacaat accaagcaat
      241 aaaccaaaga aaaaaccaac tacaaaaccc acaaacaaac cacctaccaa aaccacaaac
      301 aaaagagacc ccaaaacact agccaaaaca ccgaaaaaag aaaccaccat taacccaaca
      361 aaaaaaccaa cccccaagac cacagaaaga gacaccagca ccccacaatc cactgtgctc
      421 gacacaacca caacaaaaca cacagaaaga gacaccagca ccccacaatc cactgcgctt
      481 gacacaacca catcaaaaca cacaacccaa cagcaatctc tccactcaac cacccccgaa
      541 aacacaccca actccacaca aacacccaca gcacccgagc cctccacatc aaattccacc
      601 taa
//
LOCUS   KY328118   603 bp   cRNA   linear   VRL 13-DEC-2016
DEFINITION Human respiratory syncytial virus B isolate TH-CU/C4066/2013
            attachment glycoprotein gene, partial cds.
ACCESSION   KY328118
VERSION   KY328118
KEYWORDS   .
SOURCE   Human respiratory syncytial virus B
  ORGANISM Human respiratory syncytial virus B
            Viruses; ssRNA viruses; ssRNA negative-strand viruses;
            Mononegavirales; Pneumoviridae; Orthopneumovirus.
REFERENCE   1 (bases 1 to 603)
  AUTHORS   Thongpan,I., Mauleekoonphairoj,J., Vichiwattana,P., Korkong,S.,
            Vongpunsawad,S. and Poovorawan,Y.
  TITLE   Molecular Characterization of Respiratory Syncytial Virus in
            Thailand, 2012-2015
  JOURNAL   Unpublished
REFERENCE   2 (bases 1 to 603)
  AUTHORS   Thongpan,I., Mauleekoonphairoj,J., Vichiwattana,P., Korkong,S.,
            Vongpunsawad,S. and Poovorawan,Y.
  TITLE   Direct Submission
  JOURNAL   Submitted (13-DEC-2016) Department of Pediatrics, Center of

Excellence in Clinical Virology, Faculty of Medicine, Chulalongkorn
University, Bangkok 10330, Thailand
COMMENT ##Assembly-Data-START##
Assembly Method :: DNASTAR-Lasergene v. 6
Sequencing Technology :: Sanger dideoxy sequencing
##Assembly-Data-END##
FEATURES Location/Qualifiers
source 1..603
/organism="Human respiratory syncytial virus B"
/mol_type="viral cRNA"
/isolate="TH-CU/C4066/2013"
/host="Homo sapiens"
/db_xref="taxon:208895"
/country="Thailand"
/collection_date="01-Aug-2013"
/note="group: B"
CDS <1..603
/note="G protein"
/codon_start=1
/product="attachment glycoprotein"
/protein_id="APY20459"
/translation="IHTNSATISPNTKSETHHTTAQTKGRTSTPTQNNKPSTKPRPKN
PPKKDDYHFEVFNFVPCNICGNNQLCKSICKTIPSNKPKKKPTTKPTNKPPTKTTNKR
DPKTLAKTPKKETTINPTKKPTPKTTERDTSTPQSTVLDTTTTKHTERDTSTPQSTAL
DTTTSKHTTQQQSLHSTTPENTPNSTQTPTAPEPSTSNST"
ORIGIN
1 atccacacaa actcagccac aatatcacct aatacaaaat cagaaacaca ccatacaaca
61 gcacaaacca aaggcagaac ctctactcca acacagaaca acaagccaag cacaaaacca
121 cgtccaaaaa atccaccaaa aaaagatgat taccattttg aagtgttcaa cttcgttccc
181 tgtaatatat gtggcaacaa tcaactctgc aaatccattt gcaaaacaat accaagcaat
241 aaaccaaaga aaaaaccaac tacaaaaccc acaaacaaac cacctaccaa aaccacaaac
301 aaaagagacc ccaaaacact agccaaaaca ccgaaaaaag aaaccaccat taacccaaca
361 aaaaaaccaa cccccaagac cacagaaaga gacaccagca ccccacaatc cactgtgctc
421 gacacaacca caacaaaaca cacagaaaga gacaccagca ccccacaatc cactgcgctt
481 gacacaacca catcaaaaca cacaacccaa cagcaatctc tccactcaac cacccccgaa
541 aacacaccca actccacaca aacacccaca gcacccgagc cctccacatc aaattccacc
601 taa
//
LOCUS KY328119 603 bp cRNA linear VRL 13-DEC-2016
DEFINITION Human respiratory syncytial virus B isolate TH-CU/C4065/2013
attachment glycoprotein gene, partial cds.
ACCESSION KY328119
VERSION KY328119
KEYWORDS .
SOURCE Human respiratory syncytial virus B
ORGANISM Human respiratory syncytial virus B
Viruses; ssRNA viruses; ssRNA negative-strand viruses;
Mononegavirales; Pneumoviridae; Orthopneumovirus.
REFERENCE 1 (bases 1 to 603)
AUTHORS Thongpan,I., Mauleekoonphairoj,J., Vichiwattana,P., Korkong,S.,
Vongpunsawad,S. and Poovorawan,Y.
TITLE Molecular Characterization of Respiratory Syncytial Virus in
Thailand, 2012-2015
JOURNAL Unpublished

REFERENCE 2 (bases 1 to 603)
AUTHORS Thongpan,I., Mauleekoonphairoj,J., Vichiwattana,P., Korkong,S.,
Vongpunsawad,S. and Poovorawan,Y.
TITLE Direct Submission
JOURNAL Submitted (13-DEC-2016) Department of Pediatrics, Center of
Excellence in Clinical Virology, Faculty of Medicine, Chulalongkorn
University, Bangkok 10330, Thailand
COMMENT ##Assembly-Data-START##
Assembly Method :: DNASTAR-Lasergene v. 6
Sequencing Technology :: Sanger dideoxy sequencing
##Assembly-Data-END##
FEATURES Location/Qualifiers
source 1..603
/organism="Human respiratory syncytial virus B"
/mol_type="viral cRNA"
/isolate="TH-CU/C4065/2013"
/host="Homo sapiens"
/db_xref="taxon:208895"
/country="Thailand"
/collection_date="01-Aug-2013"
/note="group: B"
CDS <1..603
/note="G protein"
/codon_start=1
/product="attachment glycoprotein"
/protein_id="APY20460"
/translation="IHTNSATISPNTKSETHHTTAQTKGRTSTPTQNNKPSTKPRPKN
PPKKDDYHFEVFNFVPCNICGNNQLCKSICKTIPSNKPKKKPTTKPTNKPPTKTTNKR
DPKTLAKTPKKETTINPTKKPTPKTTERDTSTPQSTVLDTTTTKHTERDTSTPHSTAL
DTTTSKHTTQQQSLHSTTPENTPNSTQTPTAPEPSTSNST"
ORIGIN
1 atccacacaa actcagccac aatatcacct aatacaaaat cagaaacaca ccatacaaca
61 gcacaaacca aaggcagaac ctctactcca acacagaaca acaagccaag cacaaaacca
121 cgtccaaaaa atccaccaaa aaaagatgat taccattttg aagtgttcaa cttcgttccc
181 tgtaatatat gtggcaacaa tcaactctgc aaatccattt gcaaaacaat accaagcaat
241 aaaccaaaga aaaaaccaac tacaaaaccc acaaacaaac cacctaccaa aaccacaaac
301 aaaagagacc ccaaaacact agccaaaaca ccgaaaaaag aaaccaccat taacccaaca
361 aaaaaaccaa cccccaagac cacagaaaga gacaccagca ccccacaatc cactgtgctc
421 gacacaacca caacaaaaca cacagaaaga gacaccagca ccccacactc cactgcgctt
481 gacacaacca catcaaaaca cacaacccaa cagcaatctc tccactcaac cacccccgaa
541 aacacaccca actccacaca aacacccaca gcacccgagc cctccacatc aaattccacc
601 taa
//
LOCUS KY328120 600 bp cRNA linear VRL 13-DEC-2016
DEFINITION Human respiratory syncytial virus B isolate TH-CU/CB127/2013
attachment glycoprotein gene, partial cds.
ACCESSION KY328120
VERSION KY328120
KEYWORDS .
SOURCE Human respiratory syncytial virus B
ORGANISM Human respiratory syncytial virus B
Viruses; ssRNA viruses; ssRNA negative-strand viruses;
Mononegavirales; Pneumoviridae; Orthopneumovirus.
REFERENCE 1 (bases 1 to 600)

```
  AUTHORS   Thongpan,I., Mauleekoonphairoj,J., Vichiwattana,P., Korkong,S.,
            Vongpunsawad,S. and Poovorawan,Y.
  TITLE     Molecular Characterization of Respiratory Syncytial Virus in
            Thailand, 2012-2015
  JOURNAL   Unpublished
REFERENCE   2  (bases 1 to 600)
  AUTHORS   Thongpan,I., Mauleekoonphairoj,J., Vichiwattana,P., Korkong,S.,
            Vongpunsawad,S. and Poovorawan,Y.
  TITLE     Direct Submission
  JOURNAL   Submitted (13-DEC-2016) Department of Pediatrics, Center of
            Excellence in Clinical Virology, Faculty of Medicine, Chulalongkorn
            University, Bangkok 10330, Thailand
COMMENT     ##Assembly-Data-START##
            Assembly Method       :: DNASTAR-Lasergene v. 6
            Sequencing Technology :: Sanger dideoxy sequencing
            ##Assembly-Data-END##
FEATURES             Location/Qualifiers
     source          1..600
                     /organism="Human respiratory syncytial virus B"
                     /mol_type="viral cRNA"
                     /isolate="TH-CU/CB127/2013"
                     /host="Homo sapiens"
                     /db_xref="taxon:208895"
                     /country="Thailand"
                     /collection_date="01-Nov-2013"
                     /note="group: B"
     CDS             <1..600
                     /note="G protein"
                     /codon_start=1
                     /product="attachment glycoprotein"
                     /protein_id="APY20461"
                     /translation="HTNSATISPNTKSETHHTTAQTKGRTSTPTQNNKPSTKPRPKNP
                     PKKDDYHFEVFNFVPCSICGNNQLCKSICKTIPSNKPKKKPTTKPTNKPPTKTTNKRD
                     PKTLAKTPKKETTINPTKKPTPKTTERDTSTPQSTMLDTTTSKHTERDTSTSQSIALD
                     TTTSKHTTQQQSLYSTTPENTPNSTQTPTASEPTTSNST"
ORIGIN
        1 cacacaaact cagccacaat atcacccaat acaaaatcag aaacacacca tacaacagca
       61 caaaccaaag gcagaacctc cactccaaca cagaacaaca agccaagcac aaaaccacgt
      121 ccaaaaaatc caccaaaaaa agatgattac cattttgaag tgttcaactt cgttccctgt
      181 agtatatgtg gcaacaatca actctgcaaa tccatttgca aaacaatacc aagcaataaa
      241 ccaaagaaaa aaccaactac aaaacccaca aacaaaccac ccaccaaaac cacaaacaaa
      301 agagacccca aaacactagc caaaacaccg aaaaaagaaa ccaccattaa cccaacaaaa
      361 aaaccaaccc ccaagaccac agaaagagac accagcaccc cacaatccac tatgctcgac
      421 acaaccacat caaaacacac agaaagagac accagcacct cacaatccat tgcgcttgac
      481 acaaccacat caaaacacac aacccaacag caatctctct actcaaccac ccccgaaaac
      541 acacccaact ccacacaaac acccacagca tccgagccca ccacatcaaa ttccacctaa
//
LOCUS       KY328121                 603 bp    cRNA    linear   VRL 13-DEC-2016
DEFINITION  Human respiratory syncytial virus B isolate TH-CU/CB138/2013
            attachment glycoprotein gene, partial cds.
ACCESSION   KY328121
VERSION     KY328121
KEYWORDS    .
SOURCE      Human respiratory syncytial virus B
```

ORGANISM Human respiratory syncytial virus B
Viruses; ssRNA viruses; ssRNA negative-strand viruses;
Mononegavirales; Pneumoviridae; Orthopneumovirus.
REFERENCE   1 (bases 1 to 603)
AUTHORS   Thongpan,I., Mauleekoonphairoj,J., Vichiwattana,P., Korkong,S.,
Vongpunsawad,S. and Poovorawan,Y.
TITLE   Molecular Characterization of Respiratory Syncytial Virus in
Thailand, 2012-2015
JOURNAL   Unpublished
REFERENCE   2 (bases 1 to 603)
AUTHORS   Thongpan,I., Mauleekoonphairoj,J., Vichiwattana,P., Korkong,S.,
Vongpunsawad,S. and Poovorawan,Y.
TITLE   Direct Submission
JOURNAL   Submitted (13-DEC-2016) Department of Pediatrics, Center of
Excellence in Clinical Virology, Faculty of Medicine, Chulalongkorn
University, Bangkok 10330, Thailand
COMMENT   ##Assembly-Data-START##
Assembly Method   :: DNASTAR-Lasergene v. 6
Sequencing Technology :: Sanger dideoxy sequencing
##Assembly-Data-END##
FEATURES   Location/Qualifiers
source   1..603
/organism="Human respiratory syncytial virus B"
/mol_type="viral cRNA"
/isolate="TH-CU/CB138/2013"
/host="Homo sapiens"
/db_xref="taxon:208895"
/country="Thailand"
/collection_date="01-Nov-2013"
/note="group: B"
CDS   <1..603
/note="G protein"
/codon_start=1
/product="attachment glycoprotein"
/protein_id="APY20462"
/translation="IHTNSATISPNTKSETHHTTAQTKGRTSTPTQNNKPSTKPRPKN
PPKKDDYHFEVFNFVPCSICGNNQLCKSICKTIPSNKPKKKPTTKPTNKPPTKTTNKR
DPKTLAKTPKKETTINPTKKPTPKTTERDTSTPQSTMLDTTTSKHTERDTSTSQSIAL
DTTTSKHTTQQQSLYSTTPENTPNSTQTPTASEPTTSNST"
ORIGIN
1 atccacacaa actcagccac aatatcaccc aatacaaaat cagaaacaca ccatacaaca
61 gcacaaacca aaggcagaac ctccactcca acacagaaca acaagccaag cacaaaacca
121 cgtccaaaaa atccaccaaa aaaagatgat taccattttg aagtgttcaa cttcgttccc
181 tgtagtatat gtggcaacaa tcaactctgc aaatccattt gcaaaacaat accaagcaat
241 aaaccaaaga aaaaaccaac tacaaaaccc acaaacaaac cacccaccaa aaccacaaac
301 aaaagagacc ccaaaacact agccaaaaca ccgaaaaaag aaaccaccat taacccaaca
361 aaaaaaccaa cccccaagac cacagaaaga gacaccagca ccccacaatc cactatgctc
421 gacacaacca catcaaaaca cacagaaaga gacaccagca cctcacaatc cattgcgctt
481 gacacaacca catcaaaaca cacaacccaa cagcaatctc tctactcaac cacccccgaa
541 aacacaccca actccacaca aacacccaca gcatccgagc ccaccacatc aaattccacc
601 taa
//
LOCUS   KY328122   603 bp   cRNA   linear   VRL 13-DEC-2016
DEFINITION Human respiratory syncytial virus B isolate TH-CU/CB75/2013

attachment glycoprotein gene, partial cds.
ACCESSION KY328122
VERSION KY328122
KEYWORDS .
SOURCE Human respiratory syncytial virus B
ORGANISM Human respiratory syncytial virus B
Viruses; ssRNA viruses; ssRNA negative-strand viruses;
Mononegavirales; Pneumoviridae; Orthopneumovirus.
REFERENCE 1 (bases 1 to 603)
AUTHORS Thongpan,I., Mauleekoonphairoj,J., Vichiwattana,P., Korkong,S.,
Vongpunsawad,S. and Poovorawan,Y.
TITLE Molecular Characterization of Respiratory Syncytial Virus in
Thailand, 2012-2015
JOURNAL Unpublished
REFERENCE 2 (bases 1 to 603)
AUTHORS Thongpan,I., Mauleekoonphairoj,J., Vichiwattana,P., Korkong,S.,
Vongpunsawad,S. and Poovorawan,Y.
TITLE Direct Submission
JOURNAL Submitted (13-DEC-2016) Department of Pediatrics, Center of
Excellence in Clinical Virology, Faculty of Medicine, Chulalongkorn
University, Bangkok 10330, Thailand
COMMENT ##Assembly-Data-START##
Assembly Method :: DNASTAR-Lasergene v. 6
Sequencing Technology :: Sanger dideoxy sequencing
##Assembly-Data-END##
FEATURES Location/Qualifiers
source 1..603
/organism="Human respiratory syncytial virus B"
/mol_type="viral cRNA"
/isolate="TH-CU/CB75/2013"
/host="Homo sapiens"
/db_xref="taxon:208895"
/country="Thailand"
/collection_date="01-Aug-2013"
/note="group: B"
CDS <1..603
/note="G protein"
/codon_start=1
/product="attachment glycoprotein"
/protein_id="APY20463"
/translation="IHTNSATISPNTKSETHHTTAQTKGRTSTPTQNNKPSTKPRPKN
PPKKDDYHFEVFNFVPCSICGNNQLCKSICKTIPSNKPKKKPTTKPTNKPPTKTTNKR
DPKTLAKTPKKETTINPTKKPTPKTTERDTSTPQSTVLDTTTSKHTERDTSTSQSIAL
DTTTSKHTTQQQSLYSTTPENTPNSTQTPTASEPSTSNST"
ORIGIN
1 atccacacaa actcagccac aatatcaccc aatacaaaat cagaaacaca ccatacaaca
61 gcacaaacca aaggcagaac ctccactcca acacagaaca acaagccaag cacaaaacca
121 cgtccaaaaa atccaccaaa aaaagatgat taccattttg aagtgttcaa cttcgttccc
181 tgtagtatat gtggcaacaa tcaactctgc aaatccattt gcaaaacaat accaagcaat
241 aaaccaaaga aaaaaccaac tacaaaaccc acaaacaaac cacctaccaa aaccacaaac
301 aaaagagacc ccaaaacact agccaaaaca ccgaaaaaag aaaccaccat taacccaaca
361 aaaaaaccaa cccccaagac cacagaaaga gacaccagca ccccacaatc cactgtgctc
421 gacacaacca catcaaaaca cacagaaaga gacaccagca cctcacaatc cattgcgctt
481 gacactacca catcaaaaca cacaacccaa cagcaatctc tctactcaac cacccccgaa

541 aacacaccca actccacaca aacacccaca gcatccgagc cctccacatc aaattccacc
601 taa
//

```
LOCUS       KY328123                 600 bp    cRNA    linear   VRL 13-DEC-2016
DEFINITION  Human respiratory syncytial virus B isolate TH-CU/CB82/2013
            attachment glycoprotein gene, partial cds.
ACCESSION   KY328123
VERSION     KY328123
KEYWORDS    .
SOURCE      Human respiratory syncytial virus B
  ORGANISM  Human respiratory syncytial virus B
            Viruses; ssRNA viruses; ssRNA negative-strand viruses;
            Mononegavirales; Pneumoviridae; Orthopneumovirus.
REFERENCE   1  (bases 1 to 600)
  AUTHORS   Thongpan,I., Mauleekoonphairoj,J., Vichiwattana,P., Korkong,S.,
            Vongpunsawad,S. and Poovorawan,Y.
  TITLE     Molecular Characterization of Respiratory Syncytial Virus in
            Thailand, 2012-2015
  JOURNAL   Unpublished
REFERENCE   2  (bases 1 to 600)
  AUTHORS   Thongpan,I., Mauleekoonphairoj,J., Vichiwattana,P., Korkong,S.,
            Vongpunsawad,S. and Poovorawan,Y.
  TITLE     Direct Submission
  JOURNAL   Submitted (13-DEC-2016) Department of Pediatrics, Center of
            Excellence in Clinical Virology, Faculty of Medicine, Chulalongkorn
            University, Bangkok 10330, Thailand
COMMENT     ##Assembly-Data-START##
            Assembly Method       :: DNASTAR-Lasergene v. 6
            Sequencing Technology :: Sanger dideoxy sequencing
            ##Assembly-Data-END##
FEATURES             Location/Qualifiers
     source          1..600
                     /organism="Human respiratory syncytial virus B"
                     /mol_type="viral cRNA"
                     /isolate="TH-CU/CB82/2013"
                     /host="Homo sapiens"
                     /db_xref="taxon:208895"
                     /country="Thailand"
                     /collection_date="01-Aug-2013"
                     /note="group: B"
     CDS             <1..600
                     /note="G protein"
                     /codon_start=1
                     /product="attachment glycoprotein"
                     /protein_id="APY20464"
                     /translation="HTNSATISPNTKSETHHTTAQTKGRTSTPTQNNKPSTKPRPKNP
                     PKKDDYHFEVFNFVPCSICGNNQLCKSICKTIPSNKPKKKPTTKPTNKPPTKTTNKRD
                     PKTLAKTPKKETTINPTKKPTPKTTERDTSTPQSTVLDTTTSKHTERDTSTSQSIALD
                     TTTSKHTTQQQSLYSTTPENTPNSTQTPTASEPSTSNST"
ORIGIN
        1 cacacaaact cagccacaat atcacccaat acaaaatcag aaacacacca tacaacagca
       61 caaaccaaag gcagaacctc cactccaaca cagaacaaca agccaagcac aaaaccacgt
      121 ccaaaaaatc caccaaaaaa agatgattac cattttgaag tgttcaactt cgttccctgt
      181 agtatatgtg gcaacaatca actctgcaaa tccatttgca aaacaatacc aagcaataaa
```

```
      241 ccaaagaaaa aaccaactac aaaacccaca aacaaaccac ctaccaaaac cacaaacaaa
      301 agagacccca aaacactagc caaaacaccg aaaaaagaaa ccaccattaa cccaacaaaa
      361 aaaccaaccc ccaagaccac agaaagagac accagcaccc cacaatccac tgtgctcgac
      421 acaaccacat caaaacacac agaaagagac accagcacct cacaatccat tgcgcttgac
      481 actaccacat caaaacacac aacccaacag caatctctct actcaaccac ccccgaaaac
      541 acacccaact ccacacaaac acccacagca tccgagccct ccacatcaaa ttccacctaa
//
LOCUS       KY328124                 603 bp    cRNA    linear   VRL 13-DEC-2016
DEFINITION  Human respiratory syncytial virus B isolate TH-CU/CB71/2013
            attachment glycoprotein gene, partial cds.
ACCESSION   KY328124
VERSION     KY328124
KEYWORDS    .
SOURCE      Human respiratory syncytial virus B
  ORGANISM  Human respiratory syncytial virus B
            Viruses; ssRNA viruses; ssRNA negative-strand viruses;
            Mononegavirales; Pneumoviridae; Orthopneumovirus.
REFERENCE   1  (bases 1 to 603)
  AUTHORS   Thongpan,I., Mauleekoonphairoj,J., Vichiwattana,P., Korkong,S.,
            Vongpunsawad,S. and Poovorawan,Y.
  TITLE     Molecular Characterization of Respiratory Syncytial Virus in
            Thailand, 2012-2015
  JOURNAL   Unpublished
REFERENCE   2  (bases 1 to 603)
  AUTHORS   Thongpan,I., Mauleekoonphairoj,J., Vichiwattana,P., Korkong,S.,
            Vongpunsawad,S. and Poovorawan,Y.
  TITLE     Direct Submission
  JOURNAL   Submitted (13-DEC-2016) Department of Pediatrics, Center of
            Excellence in Clinical Virology, Faculty of Medicine, Chulalongkorn
            University, Bangkok 10330, Thailand
COMMENT     ##Assembly-Data-START##
            Assembly Method       :: DNASTAR-Lasergene v. 6
            Sequencing Technology :: Sanger dideoxy sequencing
            ##Assembly-Data-END##
FEATURES             Location/Qualifiers
     source          1..603
                     /organism="Human respiratory syncytial virus B"
                     /mol_type="viral cRNA"
                     /isolate="TH-CU/CB71/2013"
                     /host="Homo sapiens"
                     /db_xref="taxon:208895"
                     /country="Thailand"
                     /collection_date="01-Aug-2013"
                     /note="group: B"
     CDS             <1..603
                     /note="G protein"
                     /codon_start=1
                     /product="attachment glycoprotein"
                     /protein_id="APY20465"
                     /translation="IHTNSATISPNTKSETHHTTAQTKGRTSTPTQNNKPSTKPRPKN
                     PPKKDDYHFEVFNFVPCSICGNNQLCKSICKTIPSNKPKKKPTTKPTNKPPTKTTNKR
                     DPKTLAKTPKKETTINPTKKPTPKTTERDTSTPQSTVLDTTTSKHTERDTSTSQSIAL
                     DTTTSKHTTQQQSLYSTTPENTPNSTQTPTASEPSTSNST"
ORIGIN
```

```
        1 atccacacaa actcagccac aatatcaccc aatacaaaat cagaaacaca ccatacaaca
       61 gcacaaacca aaggcagaac ctccactcca acacagaaca acaagccaag cacaaaacca
      121 cgtccaaaaa atccaccaaa aaaagatgat taccattttg aagtgttcaa cttcgttccc
      181 tgtagtatat gtggcaacaa tcaactctgc aaatccattt gcaaaacaat accaagcaat
      241 aaaccaaaga aaaaaccaac tacaaaaccc acaaacaaac cacctaccaa aaccacaaac
      301 aaaagagacc ccaaaacact agccaaaaca ccgaaaaaag aaaccaccat taacccaaca
      361 aaaaaaccaa cccccaagac cacagaaaga gacaccagca ccccacaatc cactgtgctc
      421 gacacaacca catcaaaaca cacagaaaga gacaccagca cctcacaatc cattgcgctt
      481 gacactacca catcaaaaca cacaacccaa cagcaatctc tctactcaac cacccccgaa
      541 aacacaccca actccacaca aacacccaca gcatccgagc cctccacatc aaattccacc
      601 taa
//
LOCUS       KY328125                 603 bp    cRNA    linear   VRL 13-DEC-2016
DEFINITION  Human respiratory syncytial virus B isolate TH-CU/CB47/2013
            attachment glycoprotein gene, partial cds.
ACCESSION   KY328125
VERSION     KY328125
KEYWORDS    .
SOURCE      Human respiratory syncytial virus B
  ORGANISM  Human respiratory syncytial virus B
            Viruses; ssRNA viruses; ssRNA negative-strand viruses;
            Mononegavirales; Pneumoviridae; Orthopneumovirus.
REFERENCE   1  (bases 1 to 603)
  AUTHORS   Thongpan,I., Mauleekoonphairoj,J., Vichiwattana,P., Korkong,S.,
            Vongpunsawad,S. and Poovorawan,Y.
  TITLE     Molecular Characterization of Respiratory Syncytial Virus in
            Thailand, 2012-2015
  JOURNAL   Unpublished
REFERENCE   2  (bases 1 to 603)
  AUTHORS   Thongpan,I., Mauleekoonphairoj,J., Vichiwattana,P., Korkong,S.,
            Vongpunsawad,S. and Poovorawan,Y.
  TITLE     Direct Submission
  JOURNAL   Submitted (13-DEC-2016) Department of Pediatrics, Center of
            Excellence in Clinical Virology, Faculty of Medicine, Chulalongkorn
            University, Bangkok 10330, Thailand
COMMENT     ##Assembly-Data-START##
            Assembly Method       :: DNASTAR-Lasergene v. 6
            Sequencing Technology :: Sanger dideoxy sequencing
            ##Assembly-Data-END##
FEATURES             Location/Qualifiers
     source          1..603
                     /organism="Human respiratory syncytial virus B"
                     /mol_type="viral cRNA"
                     /isolate="TH-CU/CB47/2013"
                     /host="Homo sapiens"
                     /db_xref="taxon:208895"
                     /country="Thailand"
                     /collection_date="01-Jul-2013"
                     /note="group: B"
     CDS             <1..603
                     /note="G protein"
                     /codon_start=1
                     /product="attachment glycoprotein"
                     /protein_id="APY20466"
```

/translation="IHTNSATISPNTKSETHHTTAQTKGRTSTPTQNNKPSTKPRPKN
PPKKDDYHFEVFNFVPCSICGNNQLCKSICKTIPSNKPKKKPTTKPTNKPPTKTTNKR
DPKTLAKTPKKETTINPTKKPTPKTTERDTSTPQSTVLDTTTSKHTERDTSTSQSIAL
DTTTSKHTTQQQSLYSTTPENTPNSTQTPTASEPSTSNST"
ORIGIN
1 atccacacaa actcagccac aatatcaccc aatacaaaat cagaaacaca ccatacaaca
61 gcacaaacca aaggcagaac ctccactcca acacagaaca acaagccaag cacaaaacca
121 cgtccaaaaa atccaccaaa aaaagatgat taccattttg aagtgttcaa cttcgttccc
181 tgtagtatat gtggcaacaa tcaactctgc aaatccattt gcaaaacaat accaagcaat
241 aaaccaaaga aaaaaccaac tacaaaaccc acaaacaaac cacctaccaa aaccacaaac
301 aaaagagacc ccaaaacact agccaaaaca ccgaaaaaag aaaccaccat taacccaaca
361 aaaaaaccaa cccccaagac cacagaaaga gacaccagca ccccacaatc cactgtgctc
421 gacacaacca catcaaaaca cacagaaaga gacaccagca cctcacaatc cattgcgctt
481 gacactacca catcaaaaca cacaacccaa cagcaatctc tctactcaac cacccccgaa
541 aacacaccca actccacaca aacacccaca gcatccgagc cctccacatc aaattccacc
601 taa
//
LOCUS KY328126 603 bp cRNA linear VRL 13-DEC-2016
DEFINITION Human respiratory syncytial virus B isolate TH-CU/CB84/2013
attachment glycoprotein gene, partial cds.
ACCESSION KY328126
VERSION KY328126
KEYWORDS .
SOURCE Human respiratory syncytial virus B
ORGANISM Human respiratory syncytial virus B
Viruses; ssRNA viruses; ssRNA negative-strand viruses;
Mononegavirales; Pneumoviridae; Orthopneumovirus.
REFERENCE 1 (bases 1 to 603)
AUTHORS Thongpan,I., Mauleekoonphairoj,J., Vichiwattana,P., Korkong,S.,
Vongpunsawad,S. and Poovorawan,Y.
TITLE Molecular Characterization of Respiratory Syncytial Virus in
Thailand, 2012-2015
JOURNAL Unpublished
REFERENCE 2 (bases 1 to 603)
AUTHORS Thongpan,I., Mauleekoonphairoj,J., Vichiwattana,P., Korkong,S.,
Vongpunsawad,S. and Poovorawan,Y.
TITLE Direct Submission
JOURNAL Submitted (13-DEC-2016) Department of Pediatrics, Center of
Excellence in Clinical Virology, Faculty of Medicine, Chulalongkorn
University, Bangkok 10330, Thailand
COMMENT ##Assembly-Data-START##
Assembly Method :: DNASTAR-Lasergene v. 6
Sequencing Technology :: Sanger dideoxy sequencing
##Assembly-Data-END##
FEATURES Location/Qualifiers
source 1..603
/organism="Human respiratory syncytial virus B"
/mol_type="viral cRNA"
/isolate="TH-CU/CB84/2013"
/host="Homo sapiens"
/db_xref="taxon:208895"
/country="Thailand"
/collection_date="01-Aug-2013"
/note="group: B"

CDS <1..603
/note="G protein"
/codon_start=1
/product="attachment glycoprotein"
/protein_id="APY20467"
/translation="IHTNSATISPNTKSETHHTTAQTKGRTSTPTQNNKPSTKPRPKN
PPKKDDYHFEVFNFVPCSICGNNQLCKSICKTIPSNKPKKKPTTKPTNKPPTKTTNKR
DPKTLAKTPKKETTINPTKKPTPKTTERDTSTPQSTVLDTTTSKHTERDTSTSQSIAL
DTTTSKHTTQQQSLYSTTPENTPNSTQTPTASEPSTSNST"
ORIGIN
1 atccacacaa actcagccac aatatcaccc aatacaaaat cagaaacaca ccatacaaca
61 gcacaaacca aaggcagaac ctccactcca acacagaaca acaagccaag cacaaaacca
121 cgtccaaaaa atccaccaaa aaaagatgat taccattttg aagtgttcaa cttcgttccc
181 tgtagtatat gtggcaacaa tcaactctgc aaatccattt gcaaaacaat accaagcaat
241 aaaccaaaga aaaaaccaac tacaaaaccc acaaacaaac cacctaccaa aaccacaaac
301 aaaagagacc ccaaaacact agccaaaaca ccgaaaaaag aaaccaccat taacccaaca
361 aaaaaaccaa cccccaagac cacagaaaga gacaccagca ccccacaatc cactgtgctc
421 gacacaacca catcaaaaca cacagaaaga gacaccagca cctcacaatc cattgcgctt
481 gacactacca catcaaaaca cacaacccaa cagcaatctc tctactcaac cacccccgaa
541 aacacaccca actccacaca aacacccaca gcatccgagc cctccacatc aaattccacc
601 taa
//
LOCUS KY328127 603 bp cRNA linear VRL 13-DEC-2016
DEFINITION Human respiratory syncytial virus B isolate TH-CU/CB129/2013
attachment glycoprotein gene, partial cds.
ACCESSION KY328127
VERSION KY328127
KEYWORDS .
SOURCE Human respiratory syncytial virus B
ORGANISM Human respiratory syncytial virus B
Viruses; ssRNA viruses; ssRNA negative-strand viruses;
Mononegavirales; Pneumoviridae; Orthopneumovirus.
REFERENCE 1 (bases 1 to 603)
AUTHORS Thongpan,I., Mauleekoonphairoj,J., Vichiwattana,P., Korkong,S.,
Vongpunsawad,S. and Poovorawan,Y.
TITLE Molecular Characterization of Respiratory Syncytial Virus in
Thailand, 2012-2015
JOURNAL Unpublished
REFERENCE 2 (bases 1 to 603)
AUTHORS Thongpan,I., Mauleekoonphairoj,J., Vichiwattana,P., Korkong,S.,
Vongpunsawad,S. and Poovorawan,Y.
TITLE Direct Submission
JOURNAL Submitted (13-DEC-2016) Department of Pediatrics, Center of
Excellence in Clinical Virology, Faculty of Medicine, Chulalongkorn
University, Bangkok 10330, Thailand
COMMENT ##Assembly-Data-START##
Assembly Method :: DNASTAR-Lasergene v. 6
Sequencing Technology :: Sanger dideoxy sequencing
##Assembly-Data-END##
FEATURES Location/Qualifiers
source 1..603
/organism="Human respiratory syncytial virus B"
/mol_type="viral cRNA"
/isolate="TH-CU/CB129/2013"

/host="Homo sapiens"
/db_xref="taxon:208895"
/country="Thailand"
/collection_date="01-Nov-2013"
/note="group: B"
CDS <1..603
/note="G protein"
/codon_start=1
/product="attachment glycoprotein"
/protein_id="APY20468"
/translation="IHTNSATISPNTKSETHHTTAQTKGRTSTPTQNNKPSTKPRPKN
PSKKDDYHFEVFNFVPCSICGNNQLCKSICKTIPSNKPKKKPTTKPTNKPPTKTTDKR
DPKTLAKTPKKETTINPTKKPTPKTTERDTSTPQSTVLDTTTSKHTERDTSTSQSIAL
DTTTSKHTTQQQSLYSTTPENTPNSTQTPTASEPSTSNST"
ORIGIN
1 atccacacaa actcagccac aatatcaccc aatacaaaat cagaaacaca ccatacaaca
61 gcacaaacca aaggcagaac ctccactcca acacagaaca acaagccaag cacaaaacca
121 cgtccaaaaa atccatcaaa aaaagatgat taccattttg aagtgttcaa cttcgttccc
181 tgtagtatat gtggcaacaa tcaactctgc aaatccattt gcaaaacaat accaagcaat
241 aaaccaaaga aaaaaccaac tacaaaaccc acaaacaaac cacctaccaa aaccacagac
301 aaaagagacc ccaaaacact agccaaaaca ccgaaaaaag aaaccaccat taacccaaca
361 aaaaaaccaa cccccaagac cacagaaaga gacaccagca ccccacaatc cactgtgctc
421 gacacaacca catcaaaaca cacagaaaga gacaccagca cctcacaatc cattgcgctt
481 gacactacca catcaaaaca cacaacccaa cagcaatctc tctactcaac cacccccgaa
541 aacacaccca actccacaca aacacccaca gcatccgagc cctccacatc aaattccacc
601 taa
//
LOCUS KY328128 603 bp cRNA linear VRL 13-DEC-2016
DEFINITION Human respiratory syncytial virus B isolate TH-CU/CB18/2013
attachment glycoprotein gene, partial cds.
ACCESSION KY328128
VERSION KY328128
KEYWORDS .
SOURCE Human respiratory syncytial virus B
ORGANISM Human respiratory syncytial virus B
Viruses; ssRNA viruses; ssRNA negative-strand viruses;
Mononegavirales; Pneumoviridae; Orthopneumovirus.
REFERENCE 1 (bases 1 to 603)
AUTHORS Thongpan,I., Mauleekoonphairoj,J., Vichiwattana,P., Korkong,S.,
Vongpunsawad,S. and Poovorawan,Y.
TITLE Molecular Characterization of Respiratory Syncytial Virus in
Thailand, 2012-2015
JOURNAL Unpublished
REFERENCE 2 (bases 1 to 603)
AUTHORS Thongpan,I., Mauleekoonphairoj,J., Vichiwattana,P., Korkong,S.,
Vongpunsawad,S. and Poovorawan,Y.
TITLE Direct Submission
JOURNAL Submitted (13-DEC-2016) Department of Pediatrics, Center of
Excellence in Clinical Virology, Faculty of Medicine, Chulalongkorn
University, Bangkok 10330, Thailand
COMMENT ##Assembly-Data-START##
Assembly Method :: DNASTAR-Lasergene v. 6
Sequencing Technology :: Sanger dideoxy sequencing
##Assembly-Data-END##

```
FEATURES             Location/Qualifiers
     source          1..603
                     /organism="Human respiratory syncytial virus B"
                     /mol_type="viral cRNA"
                     /isolate="TH-CU/CB18/2013"
                     /host="Homo sapiens"
                     /db_xref="taxon:208895"
                     /country="Thailand"
                     /collection_date="01-Jul-2013"
                     /note="group: B"
     CDS             <1..603
                     /note="G protein"
                     /codon_start=1
                     /product="attachment glycoprotein"
                     /protein_id="APY20469"
                     /translation="IHTNSATISPNTKSETHHTTAQTKGRTSTPTQNNKPSTKPRPKN
                     PPKKDDYHFEVFNFVPCSICGNNQLCKSICKTIPSNKPKKKPTTKPTNKPPTKTTNKR
                     DPKTLAKTPKKETTINPTKKPTPKTTERDTSTPQSTVLDTTTSKHTERDTSTSQSIAL
                     DTTTSKHTTQQQSLYSTTPENTPNSTQTPTASEPSTSNST"
ORIGIN
        1 atccacacaa actcagccac aatatcaccc aatacaaaat cagaaacaca ccatacaaca
       61 gcacaaacca aaggcagaac ctccactcca acacagaaca acaagccaag cacaaaacca
      121 cgtccaaaaa atccaccaaa aaaagatgat taccattttg aagtgttcaa cttcgttccc
      181 tgtagtatat gtggcaacaa tcaactctgc aaatccattt gcaaaacaat accaagcaat
      241 aaaccaaaga aaaaaccaac tacaaaaccc acaaacaaac cacctaccaa aaccacaaac
      301 aaaagagacc ccaaaacact agccaaaaca ccgaaaaaag aaaccaccat taacccaaca
      361 aaaaaaccaa cccccaagac cacagaaaga gacacaagca ccccacaatc cactgtgctc
      421 gacacaacca catcaaaaca cacagaaaga gacaccagca cctcacaatc cattgcgctt
      481 gacactacca catcaaaaca cacaacccaa cagcaatctc tctactcaac cacccccgaa
      541 aacacaccca actccacaca aacacccaca gcatccgagc cctccacatc aaattccacc
      601 taa
//
LOCUS       KY328129                 603 bp    cRNA    linear   VRL 13-DEC-2016
DEFINITION  Human respiratory syncytial virus B isolate TH-CU/CB137/2013
            attachment glycoprotein gene, partial cds.
ACCESSION   KY328129
VERSION     KY328129
KEYWORDS    .
SOURCE      Human respiratory syncytial virus B
  ORGANISM  Human respiratory syncytial virus B
            Viruses; ssRNA viruses; ssRNA negative-strand viruses;
            Mononegavirales; Pneumoviridae; Orthopneumovirus.
REFERENCE   1  (bases 1 to 603)
  AUTHORS   Thongpan,I., Mauleekoonphairoj,J., Vichiwattana,P., Korkong,S.,
            Vongpunsawad,S. and Poovorawan,Y.
  TITLE     Molecular Characterization of Respiratory Syncytial Virus in
            Thailand, 2012-2015
  JOURNAL   Unpublished
REFERENCE   2  (bases 1 to 603)
  AUTHORS   Thongpan,I., Mauleekoonphairoj,J., Vichiwattana,P., Korkong,S.,
            Vongpunsawad,S. and Poovorawan,Y.
  TITLE     Direct Submission
  JOURNAL   Submitted (13-DEC-2016) Department of Pediatrics, Center of
            Excellence in Clinical Virology, Faculty of Medicine, Chulalongkorn
```

University, Bangkok 10330, Thailand
COMMENT ##Assembly-Data-START##
Assembly Method :: DNASTAR-Lasergene v. 6
Sequencing Technology :: Sanger dideoxy sequencing
##Assembly-Data-END##
FEATURES Location/Qualifiers
source 1..603
/organism="Human respiratory syncytial virus B"
/mol_type="viral cRNA"
/isolate="TH-CU/CB137/2013"
/host="Homo sapiens"
/db_xref="taxon:208895"
/country="Thailand"
/collection_date="01-Nov-2013"
/note="group: B"
CDS <1..603
/note="G protein"
/codon_start=1
/product="attachment glycoprotein"
/protein_id="APY20470"
/translation="IHTNSATISPNTKSETHHTTAQTKGRTSTPTQNNKPSTKPRPKN
PPKKDDYHFEVFNFVPCSICGNNQLCKSICKTIPSNKPKKKPTTKPTNKPPTKTTNKR
DPKTLAKTPKKETTTNPTKKPTPKTTERDTSTPQSTVLDTTTSKHTERDTSTSQSIAL
DTTTSKHTTQQQSLYSTTPENTPNSTQTPTASEPSTSNST"
ORIGIN
1 atccacacaa actcagccac aatatcaccc aatacaaaat cagaaacaca ccatacaaca
61 gcacaaacca aaggcagaac ctccactcca acacagaaca acaagccaag cacaaaacca
121 cgtccaaaaa atccaccaaa aaaagatgat taccattttg aagtgttcaa cttcgttccc
181 tgtagtatat gtggcaacaa tcaactctgc aaatccattt gcaaaacaat accaagcaat
241 aaaccaaaga aaaaaccaac tacaaaaccc acaaacaaac cacctaccaa aaccacaaac
301 aaaagagacc ccaaaacact agccaaaaca ccgaaaaaag aaaccaccac caacccaaca
361 aaaaaaccaa cccccaagac cacagaaaga gacaccagca ccccacaatc cactgtgctc
421 gacacaacca catcaaaaca cacagaaaga gacaccagca cctcacaatc cattgcgctt
481 gacacaacca catcaaaaca cacaacccaa cagcagtctc tctactcaac cacccccgaa
541 aacacaccca actccacaca aacacccaca gcatccgagc cctccacatc aaattccacc
601 taa
//
LOCUS KY328130 624 bp cRNA linear VRL 13-DEC-2016
DEFINITION Human respiratory syncytial virus B isolate TH-CU/C3115/2012
attachment glycoprotein gene, partial cds.
ACCESSION KY328130
VERSION KY328130
KEYWORDS .
SOURCE Human respiratory syncytial virus B
ORGANISM Human respiratory syncytial virus B
Viruses; ssRNA viruses; ssRNA negative-strand viruses;
Mononegavirales; Pneumoviridae; Orthopneumovirus.
REFERENCE 1 (bases 1 to 624)
AUTHORS Thongpan,I., Mauleekoonphairoj,J., Vichiwattana,P., Korkong,S.,
Vongpunsawad,S. and Poovorawan,Y.
TITLE Molecular Characterization of Respiratory Syncytial Virus in
Thailand, 2012-2015
JOURNAL Unpublished
REFERENCE 2 (bases 1 to 624)

AUTHORS   Thongpan,I., Mauleekoonphairoj,J., Vichiwattana,P., Korkong,S.,
Vongpunsawad,S. and Poovorawan,Y.
TITLE   Direct Submission
JOURNAL   Submitted (13-DEC-2016) Department of Pediatrics, Center of
Excellence in Clinical Virology, Faculty of Medicine, Chulalongkorn
University, Bangkok 10330, Thailand
COMMENT   ##Assembly-Data-START##
Assembly Method   :: DNASTAR-Lasergene v. 6
Sequencing Technology :: Sanger dideoxy sequencing
##Assembly-Data-END##
FEATURES   Location/Qualifiers
source   1..624
/organism="Human respiratory syncytial virus B"
/mol_type="viral cRNA"
/isolate="TH-CU/C3115/2012"
/host="Homo sapiens"
/db_xref="taxon:208895"
/country="Thailand"
/collection_date="01-Aug-2012"
/note="group: B"
CDS   <1..624
/note="G protein"
/codon_start=1
/product="attachment glycoprotein"
/protein_id="APY20471"
/translation="IHTNSATISPNTKSETHHTTAQTKGRTSTPTQNNKPSTKPRPKN
PPKKDDYHFEVFNFVPCSICGNNQLCKSICKTIPSNKPKKKPTTKPTNKPPTKTTNKR
DPKPLAKTPKKETTINPTKKPTPKTTERDTSTPQSTVLDTTTSKHTERDTSTSQSIAL
DTTTSKHTTQQQSLYSTTPENTPNSTQTPTASEPSTSNSTQKLQSYA"
ORIGIN
1 atccacacaa actcagccac aatatcaccc aatacaaaat cagaaacaca ccatacaaca
61 gcacaaacca aaggcagaac ctccactcca acacagaaca acaagccaag cacaaaacca
121 cgtccaaaaa atccaccaaa aaaagatgat taccattttg aagtgttcaa cttcgttccc
181 tgtagtatat gtggcaacaa tcaactctgc aaatccattt gcaaaacaat accaagcaat
241 aaaccaaaga aaaaaccaac tacaaaaccc acaaacaaac cacccaccaa aaccacaaac
301 aaaagagacc ccaaaccact agccaaaaca ccgaaaaaag aaaccaccat caacccaaca
361 aaaaaaccaa cccccaagac cacagaaaga gacaccagca ccccacaatc cactgtgctc
421 gacacaacca catcaaaaca cacagaaaga gacaccagca cctcacaatc cattgcgctt
481 gacacaacca catcaaaaca cacaacccaa cagcaatctc tctactcaac cacccccgaa
541 aacacaccca actccacaca aacacccaca gcatccgagc cctccacatc aaattccacc
601 caaaaactcc agtcatatgc ttag
//
LOCUS   KY328131   624 bp   cRNA   linear   VRL 13-DEC-2016
DEFINITION Human respiratory syncytial virus B isolate TH-CU/B10985/2014
attachment glycoprotein gene, partial cds.
ACCESSION   KY328131
VERSION   KY328131
KEYWORDS   .
SOURCE   Human respiratory syncytial virus B
ORGANISM Human respiratory syncytial virus B
Viruses; ssRNA viruses; ssRNA negative-strand viruses;
Mononegavirales; Pneumoviridae; Orthopneumovirus.
REFERENCE   1 (bases 1 to 624)
AUTHORS   Thongpan,I., Mauleekoonphairoj,J., Vichiwattana,P., Korkong,S.,

Vongpunsawad,S. and Poovorawan,Y.
TITLE Molecular Characterization of Respiratory Syncytial Virus in
Thailand, 2012-2015
JOURNAL Unpublished
REFERENCE 2 (bases 1 to 624)
AUTHORS Thongpan,I., Mauleekoonphairoj,J., Vichiwattana,P., Korkong,S.,
Vongpunsawad,S. and Poovorawan,Y.
TITLE Direct Submission
JOURNAL Submitted (13-DEC-2016) Department of Pediatrics, Center of
Excellence in Clinical Virology, Faculty of Medicine, Chulalongkorn
University, Bangkok 10330, Thailand
COMMENT ##Assembly-Data-START##
Assembly Method :: DNASTAR-Lasergene v. 6
Sequencing Technology :: Sanger dideoxy sequencing
##Assembly-Data-END##
FEATURES Location/Qualifiers
source 1..624
/organism="Human respiratory syncytial virus B"
/mol_type="viral cRNA"
/isolate="TH-CU/B10985/2014"
/host="Homo sapiens"
/db_xref="taxon:208895"
/country="Thailand"
/collection_date="12-Sep-2014"
/note="group: B"
CDS <1..624
/note="G protein"
/codon_start=1
/product="attachment glycoprotein"
/protein_id="APY20472"
/translation="IHTNSATISPNTKSETHHTTAQTKGRTSTPTQNNKPSTKPRPKN
PPKKDDYHFEVFNFVPCSICGNNQLCKSICKTIPSNKPKKKPTTKPTNKPPTKTTNKR
DPKTLAKTPKKETTINPTKKPTPKTTERDTSTPQSTVLDTTISKHTERDTSTSQSIAL
DTTTSKHTTQQQSLYSTTPENTPNSAQTPTASEPSTSNSTQKLQSYA"
ORIGIN
1 atccacacaa actcagccac aatatcacct aatacaaaat cagaaacaca ccatacaaca
61 gcacaaacca aaggcagaac ctctactcca acacagaaca acaagccaag cacaaaacca
121 cgtccaaaaa atccaccaaa aaaagatgat taccattttg aagtgttcaa cttcgttccc
181 tgtagtatat gtggcaacaa tcaactctgc aaatccattt gcaaaacaat accaagcaat
241 aaaccaaaga aaaaaccaac cacaaaaccc acaaacaaac cacccaccaa aaccacaaac
301 aaaagagacc ccaaaacact agccaaaaca ccgaaaaaag aaaccaccat taacccaaca
361 aaaaaaccaa cccccaagac cacagaaaga gacaccagca ccccacaatc cactgtgctc
421 gacacaacca tatcaaaaca cacagaaaga gacaccagca cctcacaatc cattgcactt
481 gacacaacca catcaaaaca cacaacccaa cagcaatctc tctactcaac cacccccgaa
541 aacacaccca actccgcaca aacacccaca gcatccgagc cctccacatc aaattccacc
601 caaaaactcc agtcatatgc ttag
//
LOCUS KY328132 603 bp cRNA linear VRL 13-DEC-2016
DEFINITION Human respiratory syncytial virus B isolate TH-CU/C4144/2013
attachment glycoprotein gene, partial cds.
ACCESSION KY328132
VERSION KY328132
KEYWORDS .
SOURCE Human respiratory syncytial virus B

```
  ORGANISM  Human respiratory syncytial virus B
            Viruses; ssRNA viruses; ssRNA negative-strand viruses;
            Mononegavirales; Pneumoviridae; Orthopneumovirus.
REFERENCE   1  (bases 1 to 603)
  AUTHORS   Thongpan,I., Mauleekoonphairoj,J., Vichiwattana,P., Korkong,S.,
            Vongpunsawad,S. and Poovorawan,Y.
  TITLE     Molecular Characterization of Respiratory Syncytial Virus in
            Thailand, 2012-2015
  JOURNAL   Unpublished
REFERENCE   2  (bases 1 to 603)
  AUTHORS   Thongpan,I., Mauleekoonphairoj,J., Vichiwattana,P., Korkong,S.,
            Vongpunsawad,S. and Poovorawan,Y.
  TITLE     Direct Submission
  JOURNAL   Submitted (13-DEC-2016) Department of Pediatrics, Center of
            Excellence in Clinical Virology, Faculty of Medicine, Chulalongkorn
            University, Bangkok 10330, Thailand
COMMENT     ##Assembly-Data-START##
            Assembly Method       :: DNASTAR-Lasergene v. 6
            Sequencing Technology :: Sanger dideoxy sequencing
            ##Assembly-Data-END##
FEATURES             Location/Qualifiers
     source          1..603
                     /organism="Human respiratory syncytial virus B"
                     /mol_type="viral cRNA"
                     /isolate="TH-CU/C4144/2013"
                     /host="Homo sapiens"
                     /db_xref="taxon:208895"
                     /country="Thailand"
                     /collection_date="01-Sep-2013"
                     /note="group: B"
     CDS             <1..603
                     /note="G protein"
                     /codon_start=1
                     /product="attachment glycoprotein"
                     /protein_id="APY20473"
                     /translation="IHTNSATISPNTKSETHHTTAQTKGRTSTPTQNNKPSTKPRPKN
                     PPKKDDYHFEVFNFVPCSICGNNQLCKSICKTIPSNKPKKKPTTKPTNKPPTKTTNKR
                     DPKTLAKTPKKETTINPTKKPTPKTTERDTSTSQSTVLDTTTSKHTERDTSTPQSTAL
                     DTTTSKHTTQQQSLHSTTPENTPNSTQTPTASEPSTSNST"
ORIGIN
        1 atccacacaa actcagccac aatatcaccc aatacaaaat cagaaacaca ccacacaaca
       61 gcacaaacca aaggcagaac ctctactcca acacagaaca acaagccaag cacaaaacca
      121 cgtccaaaaa atccaccaaa aaaagatgat taccattttg aagtgttcaa cttcgttccc
      181 tgtagtatat gtggcaacaa tcaactctgc aaatccattt gcaaaacaat accaagcaat
      241 aaaccaaaga aaaaaccaac tacaaaaccc acaaacaaac cacctaccaa aaccacaaac
      301 aaaagagacc ccaaaacact agccaaaaca ccgaaaaaag aaaccaccat taacccaaca
      361 aaaaaaccaa cccccaaaac cacagaaaga gacaccagca cctcacagtc cactgtgctc
      421 gacacaacca catcaaaaca cacagaaaga gacaccagca ccccacaatc cactgcgctt
      481 gacacaacca catcaaaaca cacaacccaa cagcaatctc tccactcaac cacccccgaa
      541 aacacaccca actccacaca aacacccaca gcatccgagc cctccacatc aaattccacc
      601 taa
//
LOCUS       KY328133                 603 bp    cRNA    linear   VRL 13-DEC-2016
DEFINITION  Human respiratory syncytial virus B isolate TH-CU/C5090/2014
```

attachment glycoprotein gene, partial cds.
ACCESSION KY328133
VERSION KY328133
KEYWORDS .
SOURCE Human respiratory syncytial virus B
ORGANISM Human respiratory syncytial virus B
Viruses; ssRNA viruses; ssRNA negative-strand viruses;
Mononegavirales; Pneumoviridae; Orthopneumovirus.
REFERENCE 1 (bases 1 to 603)
AUTHORS Thongpan,I., Mauleekoonphairoj,J., Vichiwattana,P., Korkong,S.,
Vongpunsawad,S. and Poovorawan,Y.
TITLE Molecular Characterization of Respiratory Syncytial Virus in
Thailand, 2012-2015
JOURNAL Unpublished
REFERENCE 2 (bases 1 to 603)
AUTHORS Thongpan,I., Mauleekoonphairoj,J., Vichiwattana,P., Korkong,S.,
Vongpunsawad,S. and Poovorawan,Y.
TITLE Direct Submission
JOURNAL Submitted (13-DEC-2016) Department of Pediatrics, Center of
Excellence in Clinical Virology, Faculty of Medicine, Chulalongkorn
University, Bangkok 10330, Thailand
COMMENT ##Assembly-Data-START##
Assembly Method :: DNASTAR-Lasergene v. 6
Sequencing Technology :: Sanger dideoxy sequencing
##Assembly-Data-END##
FEATURES Location/Qualifiers
source 1..603
/organism="Human respiratory syncytial virus B"
/mol_type="viral cRNA"
/isolate="TH-CU/C5090/2014"
/host="Homo sapiens"
/db_xref="taxon:208895"
/country="Thailand"
/collection_date="01-Sep-2014"
/note="group: B"
CDS <1..603
/note="G protein"
/codon_start=1
/product="attachment glycoprotein"
/protein_id="APY20474"
/translation="IHTNSATISPNTKSETHHTTAQTKGRTSTPTQNNKPSTKPRPKN
PPKKDDYHFEVFNFVPCSICGNNQLCKSICKTIPSNKPKKKPTTKPTNKPPTKTTNKR
DPKTLAKTPKKETTINPTKKPTPKTTERDTSTPQSTVLDTTTSKHTERDTSTSQSIAL
DTTTSKQTTQQLSLYSTTPENTPNSTQTPTASEPSTSNST"
ORIGIN
1 atccacacaa actcagccac aatatcaccc aatacaaaat cagaaacaca ccatacaaca
61 gcacaaacca aaggcagaac ctccactcca acacagaaca acaagccaag cacaaaacca
121 cgtccaaaaa atccaccaaa aaaagatgat taccattttg aagtgttcaa cttcgttccc
181 tgtagtatat gtggcaacaa tcaactctgc aaatccattt gcaaaacaat accaagcaat
241 aaaccaaaga aaaaaccaac tacaaaaccc acaaacaaac cacctaccaa aaccacaaac
301 aaaagagacc ccaaaacact agccaaaaca ccgaaaaaag aaaccaccat taacccaaca
361 aaaaaaccaa cccccaagac cacagaaaga gacaccagca ccccacaatc cactgtgctc
421 gacacaacca catcaaaaca cacagaaaga gacaccagca cctcacaatc cattgcgctt
481 gacacaacca catcaaaaca aacaacccaa cagctatctc tctactcaac cacccccgaa

```
     541 aacacaccca actccacaca aacacccaca gcatccgagc cctccacatc aaattccacc
     601 taa
//
LOCUS       KY328134                 591 bp    cRNA    linear   VRL 13-DEC-2016
DEFINITION  Human respiratory syncytial virus B isolate TH-CU/C5155/2014
            attachment glycoprotein gene, partial cds.
ACCESSION   KY328134
VERSION     KY328134
KEYWORDS    .
SOURCE      Human respiratory syncytial virus B
  ORGANISM  Human respiratory syncytial virus B
            Viruses; ssRNA viruses; ssRNA negative-strand viruses;
            Mononegavirales; Pneumoviridae; Orthopneumovirus.
REFERENCE   1  (bases 1 to 591)
  AUTHORS   Thongpan,I., Mauleekoonphairoj,J., Vichiwattana,P., Korkong,S.,
            Vongpunsawad,S. and Poovorawan,Y.
  TITLE     Molecular Characterization of Respiratory Syncytial Virus in
            Thailand, 2012-2015
  JOURNAL   Unpublished
REFERENCE   2  (bases 1 to 591)
  AUTHORS   Thongpan,I., Mauleekoonphairoj,J., Vichiwattana,P., Korkong,S.,
            Vongpunsawad,S. and Poovorawan,Y.
  TITLE     Direct Submission
  JOURNAL   Submitted (13-DEC-2016) Department of Pediatrics, Center of
            Excellence in Clinical Virology, Faculty of Medicine, Chulalongkorn
            University, Bangkok 10330, Thailand
COMMENT     ##Assembly-Data-START##
            Assembly Method       :: DNASTAR-Lasergene v. 6
            Sequencing Technology :: Sanger dideoxy sequencing
            ##Assembly-Data-END##
FEATURES             Location/Qualifiers
     source          1..591
                     /organism="Human respiratory syncytial virus B"
                     /mol_type="viral cRNA"
                     /isolate="TH-CU/C5155/2014"
                     /host="Homo sapiens"
                     /db_xref="taxon:208895"
                     /country="Thailand"
                     /collection_date="01-Oct-2014"
                     /note="group: B"
     CDS             <1..591
                     /note="G protein"
                     /codon_start=1
                     /product="attachment glycoprotein"
                     /protein_id="APY20475"
                     /translation="SATISPNTKSETHHTTAQTKGRTSTPTQNNKPSTKPRPKNPPKK
                     DDYHFEVFNFVPCSICGNNQLCKSICKTIPSNKPKKKPTTKPTNKPPTKTTNKRDPKT
                     LAKTPKKETTINPTKKPTPKTTERDTSTPQSTVLDTTTSKHTERDTSTSQSIALDTTT
                     SKQTTQQLSLYSTTPENTPNSTQTPTASEPSTSNST"
ORIGIN
        1 tcagccacaa tatcacccaa tacaaaatca gaaacacacc atacaacagc acaaaccaaa
       61 ggcagaacct ccactccaac acagaacaac aagccaagca caaaaccacg tccaaaaaat
      121 ccaccaaaaa aagatgatta ccattttgaa gtgttcaact tcgttccctg tagtatatgt
      181 ggcaacaatc aactctgcaa atccatttgc aaaacaatac caagcaataa accaaagaaa
```

```
     241 aaaccaacta caaaacccac aaacaaacca cctaccaaaa ccacaaacaa aagagacccc
     301 aaaacactag ccaaaacacc gaaaaaagaa accaccatta acccaacaaa aaaaccaacc
     361 cccaagacca cagaaagaga caccagcacc ccacaatcca ctgtgctcga cacaaccaca
     421 tcaaaacaca cagaaagaga caccagcacc tcacaatcca ttgcgcttga cacaaccaca
     481 tcaaaacaaa caacccaaca gctatctctc tactcaacca cccccgaaaa cacacccaac
     541 tccacacaaa cacccacagc atccgagccc tccacatcaa attccaccta a
//
LOCUS       KY328135                 624 bp    cRNA    linear   VRL 13-DEC-2016
DEFINITION  Human respiratory syncytial virus B isolate TH-CU497/2012
            attachment glycoprotein gene, partial cds.
ACCESSION   KY328135
VERSION     KY328135
KEYWORDS    .
SOURCE      Human respiratory syncytial virus B
  ORGANISM  Human respiratory syncytial virus B
            Viruses; ssRNA viruses; ssRNA negative-strand viruses;
            Mononegavirales; Pneumoviridae; Orthopneumovirus.
REFERENCE   1  (bases 1 to 624)
  AUTHORS   Thongpan,I., Mauleekoonphairoj,J., Vichiwattana,P., Korkong,S.,
            Vongpunsawad,S. and Poovorawan,Y.
  TITLE     Molecular Characterization of Respiratory Syncytial Virus in
            Thailand, 2012-2015
  JOURNAL   Unpublished
REFERENCE   2  (bases 1 to 624)
  AUTHORS   Thongpan,I., Mauleekoonphairoj,J., Vichiwattana,P., Korkong,S.,
            Vongpunsawad,S. and Poovorawan,Y.
  TITLE     Direct Submission
  JOURNAL   Submitted (13-DEC-2016) Department of Pediatrics, Center of
            Excellence in Clinical Virology, Faculty of Medicine, Chulalongkorn
            University, Bangkok 10330, Thailand
COMMENT     ##Assembly-Data-START##
            Assembly Method       :: DNASTAR-Lasergene v. 6
            Sequencing Technology :: Sanger dideoxy sequencing
            ##Assembly-Data-END##
FEATURES             Location/Qualifiers
     source          1..624
                     /organism="Human respiratory syncytial virus B"
                     /mol_type="viral cRNA"
                     /isolate="TH-CU497/2012"
                     /host="Homo sapiens"
                     /db_xref="taxon:208895"
                     /country="Thailand"
                     /collection_date="01-Nov-2012"
                     /note="group: B"
     CDS             <1..624
                     /note="G protein"
                     /codon_start=1
                     /product="attachment glycoprotein"
                     /protein_id="APY20476"
                     /translation="IHTNSATISPNTKSETHHTTAQTKGRTSTPTQNNKPSTKPRPKN
                     PPKKDDYHFEVFNFVPCSICGNNQLCKSICKTIPSNKPKKKPTTKPTNKPPTKTTNKR
                     DPKTLAKTPKKETTINPTKKPTPKTTERDTSTPQSTVLDTTTSKHTERDTSTPQSIAL
                     DTTTSKHTTQQQSPYSTTPENTHNSTQTPTASEPSTSNSTQKLQSYA"
ORIGIN
```

```
        1 atccacacaa actcagccac aatatcaccc aacacaaaat cagaaacaca ccatacaaca
       61 gcacaaacca aaggcagaac ctctactcca acacagaaca acaagccaag cacaaaacca
      121 cgtccaaaaa atccaccaaa aaaagatgat taccattttg aagtgttcaa ctttgttccc
      181 tgtagtatat gtggcaacaa tcaactctgc aaatccattt gcaaaacaat accaagcaat
      241 aaaccaaaga aaaaaccaac tacaaaaccc acaaacaaac cacctaccaa aaccacaaac
      301 aaaagagacc ccaaaacact agccaaaaca ccgaaaaaag aaaccaccat taacccaaca
      361 aaaaaaccaa cccccaagac cacagaaaga gacaccagca ccccacaatc cactgtgctc
      421 gacacaacca catcaaaaca cacagaaaga gacaccagca ccccacaatc cattgcgctt
      481 gacacaacca catcaaaaca cacaacccaa cagcaatctc cctactcaac cacccccgaa
      541 aacacacaca actccacaca aacacccaca gcatccgagc cctccacatc aaattctacc
      601 caaaaactcc agtcatatgc ttag
//
LOCUS       KY328136                 603 bp    cRNA    linear   VRL 13-DEC-2016
DEFINITION  Human respiratory syncytial virus B isolate B/TH-CU448/2012
            attachment glycoprotein gene, partial cds.
ACCESSION   KY328136
VERSION     KY328136
KEYWORDS    .
SOURCE      Human respiratory syncytial virus B
  ORGANISM  Human respiratory syncytial virus B
            Viruses; ssRNA viruses; ssRNA negative-strand viruses;
            Mononegavirales; Pneumoviridae; Orthopneumovirus.
REFERENCE   1  (bases 1 to 603)
  AUTHORS   Thongpan,I., Mauleekoonphairoj,J., Vichiwattana,P., Korkong,S.,
            Vongpunsawad,S. and Poovorawan,Y.
  TITLE     Molecular Characterization of Respiratory Syncytial Virus in
            Thailand, 2012-2015
  JOURNAL   Unpublished
REFERENCE   2  (bases 1 to 603)
  AUTHORS   Thongpan,I., Mauleekoonphairoj,J., Vichiwattana,P., Korkong,S.,
            Vongpunsawad,S. and Poovorawan,Y.
  TITLE     Direct Submission
  JOURNAL   Submitted (13-DEC-2016) Department of Pediatrics, Center of
            Excellence in Clinical Virology, Faculty of Medicine, Chulalongkorn
            University, Bangkok 10330, Thailand
COMMENT     ##Assembly-Data-START##
            Assembly Method       :: DNASTAR-Lasergene v. 6
            Sequencing Technology :: Sanger dideoxy sequencing
            ##Assembly-Data-END##
FEATURES             Location/Qualifiers
     source          1..603
                     /organism="Human respiratory syncytial virus B"
                     /mol_type="viral cRNA"
                     /isolate="B/TH-CU448/2012"
                     /host="Homo sapiens"
                     /db_xref="taxon:208895"
                     /country="Thailand"
                     /collection_date="01-Sep-2012"
                     /note="group: B"
     CDS             <1..603
                     /note="G protein"
                     /codon_start=1
                     /product="attachment glycoprotein"
                     /protein_id="APY20477"
```

/translation="IHTNSATISPNTKSETHHTTAQTKGRTSTPTQNNKPSTKPRPKN
PPKKDDYHFEVFNFVPCSICGNNQLCKSICKTIPSNKPKKKPTTKPTNKPPTKTTNKR
DPKTLAKTPKKETTINPTKKPTPKTTERDTSTPQSTVLDTTTSKNTERDTSTSQSTAL
DTTTSKHTTQQQSLYSTTPENTPNSTQTPTASELSTSNST"
ORIGIN
1 atccacacaa actcagccac aatatcacct aatacaaaat cagaaacaca ccatacaaca
61 gcacaaacca aaggcagaac ctctactcca acacagaaca acaagccaag cacaaaacca
121 cgtccaaaaa atccaccaaa aaaagatgat taccattttg aagtgttcaa cttcgttccc
181 tgtagtatat gtggcaacaa tcaactctgc aaatccattt gcaaaacaat accaagcaat
241 aaaccaaaga aaaaaccaac tacaaaaccc acaaacaaac cacccaccaa aaccacaaac
301 aaaagagacc ccaaaacact agccaaaaca ccgaaaaaag aaaccaccat caacccaaca
361 aaaaaaccaa cccccaagac cacagaaaga gacaccagca ccccacaatc cactgtgctc
421 gacacaacca catcaaaaaa cacagaaaga gacaccagca cttcacaatc caccgcgctt
481 gacacaacca catcaaaaca cacaacccaa cagcaatctc tctactcaac cacccccgaa
541 aacacaccca actccacaca aacacccaca gcatccgagc tctccacatc aaattccacc
601 taa
//
LOCUS KY328137 624 bp cRNA linear VRL 13-DEC-2016
DEFINITION Human respiratory syncytial virus B isolate TH-CU/B10997/2014
attachment glycoprotein gene, partial cds.
ACCESSION KY328137
VERSION KY328137
KEYWORDS .
SOURCE Human respiratory syncytial virus B
ORGANISM Human respiratory syncytial virus B
Viruses; ssRNA viruses; ssRNA negative-strand viruses;
Mononegavirales; Pneumoviridae; Orthopneumovirus.
REFERENCE 1 (bases 1 to 624)
AUTHORS Thongpan,I., Mauleekoonphairoj,J., Vichiwattana,P., Korkong,S.,
Vongpunsawad,S. and Poovorawan,Y.
TITLE Molecular Characterization of Respiratory Syncytial Virus in
Thailand, 2012-2015
JOURNAL Unpublished
REFERENCE 2 (bases 1 to 624)
AUTHORS Thongpan,I., Mauleekoonphairoj,J., Vichiwattana,P., Korkong,S.,
Vongpunsawad,S. and Poovorawan,Y.
TITLE Direct Submission
JOURNAL Submitted (13-DEC-2016) Department of Pediatrics, Center of
Excellence in Clinical Virology, Faculty of Medicine, Chulalongkorn
University, Bangkok 10330, Thailand
COMMENT ##Assembly-Data-START##
Assembly Method :: DNASTAR-Lasergene v. 6
Sequencing Technology :: Sanger dideoxy sequencing
##Assembly-Data-END##
FEATURES Location/Qualifiers
source 1..624
/organism="Human respiratory syncytial virus B"
/mol_type="viral cRNA"
/isolate="TH-CU/B10997/2014"
/host="Homo sapiens"
/db_xref="taxon:208895"
/country="Thailand"
/collection_date="16-Sep-2014"
/note="group: B"

```
     CDS             <1..624
                     /note="G protein"
                     /codon_start=1
                     /product="attachment glycoprotein"
                     /protein_id="APY20478"
                     /translation="IHTNSATISPNTKSETHHTTAQTKGRTSTPTQNNKPSTKPRPKN
                     PPKKDDYHFEVFNFVPCSICGNNQLCKSICKTIPSNKPKKKPTTKPTNKPPTKTTNKR
                     DPKTLAKTPKKETTINPTKKPTPKTTERDTSTPQSTVLDTTTSKHTERDTRTPQSTAL
                     DKTTSKHITQQQSLHSTTPENTPNSTQTPTASEPSTSNSTQKLQSYA"
ORIGIN
        1 atccacacaa actcagccac aatatcacct aatacaaaat cagaaacaca ccatacaaca
       61 gcacaaacca aaggcagaac ctctactcca acacagaaca acaagccaag cacaaaacca
      121 cgtccaaaaa atccaccaaa aaaagatgat taccattttg aagtgttcaa cttcgttccc
      181 tgtagtatat gtggcaacaa tcaactctgc aaatccattt gcaaaacaat accaagcaat
      241 aaaccaaaga aaaaaccaac cactaaaccc acaaacaaac cacctaccaa aaccacaaac
      301 aaaagagacc ccaaaacact agccaaaaca ccgaaaaaag aaaccaccat taacccaaca
      361 aaaaaaccaa cccccaagac cacagaaaga gacaccagca ccccacaatc cactgtgctt
      421 gacacaacca catcaaaaca cacagaaaga gacaccagaa ccccacaatc cactgcgctt
      481 gacaaaacca catcaaaaca cataacccaa cagcaatctc tccactcaac cacccccgaa
      541 aacacaccca actccacaca aacacccaca gcatccgagc cctccacatc aaattccacc
      601 caaaaactcc agtcatatgc ttag
//
LOCUS       KY328138                 624 bp    cRNA    linear   VRL 13-DEC-2016
DEFINITION  Human respiratory syncytial virus B isolate TH-CU/C5217/2014
            attachment glycoprotein gene, partial cds.
ACCESSION   KY328138
VERSION     KY328138
KEYWORDS    .
SOURCE      Human respiratory syncytial virus B
  ORGANISM  Human respiratory syncytial virus B
            Viruses; ssRNA viruses; ssRNA negative-strand viruses;
            Mononegavirales; Pneumoviridae; Orthopneumovirus.
REFERENCE   1  (bases 1 to 624)
  AUTHORS   Thongpan,I., Mauleekoonphairoj,J., Vichiwattana,P., Korkong,S.,
            Vongpunsawad,S. and Poovorawan,Y.
  TITLE     Molecular Characterization of Respiratory Syncytial Virus in
            Thailand, 2012-2015
  JOURNAL   Unpublished
REFERENCE   2  (bases 1 to 624)
  AUTHORS   Thongpan,I., Mauleekoonphairoj,J., Vichiwattana,P., Korkong,S.,
            Vongpunsawad,S. and Poovorawan,Y.
  TITLE     Direct Submission
  JOURNAL   Submitted (13-DEC-2016) Department of Pediatrics, Center of
            Excellence in Clinical Virology, Faculty of Medicine, Chulalongkorn
            University, Bangkok 10330, Thailand
COMMENT     ##Assembly-Data-START##
            Assembly Method       :: DNASTAR-Lasergene v. 6
            Sequencing Technology :: Sanger dideoxy sequencing
            ##Assembly-Data-END##
FEATURES             Location/Qualifiers
     source          1..624
                     /organism="Human respiratory syncytial virus B"
                     /mol_type="viral cRNA"
                     /isolate="TH-CU/C5217/2014"
```

```
                     /host="Homo sapiens"
                     /db_xref="taxon:208895"
                     /country="Thailand"
                     /collection_date="01-Nov-2014"
                     /note="group: B"
     CDS             <1..624
                     /note="G protein"
                     /codon_start=1
                     /product="attachment glycoprotein"
                     /protein_id="APY20479"
                     /translation="IHTNSATISPNTKSETHHTTAQTKGRTSTPTQNNKPSTKPRPKN
                     PPKKDDYHFEVFNFVPCSICGNNQLCKSICKTIPSNKPKKKPTTKPTNKPPTKTTNKR
                     DPKTLAKTPKKETTINPTKKPTPKTTERDTSTPQSTVLDTTTSKHTERDTRTPQSTAL
                     DKTTSKHITQQQSLHSTTPENTPNSTQTPTASEPSTSNSTQKLQSYA"
ORIGIN
        1 atccacacaa actcagccac aatatcacct aatacaaaat cagaaacaca ccatacaaca
       61 gcacaaacca aaggcagaac ctctactcca acacagaaca acaagccaag cacaaaacca
      121 cgtccaaaaa atccaccaaa aaaagatgat taccattttg aagtgttcaa cttcgttccc
      181 tgtagtatat gtggcaacaa tcaactctgc aaatccattt gcaaaacaat accaagcaat
      241 aaaccaaaga aaaaaccaac cactaaaccc acaaacaaac cacctaccaa aaccacaaac
      301 aaaagagacc ccaaaacact agccaaaaca ccgaaaaaag aaaccaccat taacccaaca
      361 aaaaaaccaa cccccaagac cacagaaaga gacaccagca ccccacaatc cactgtgctt
      421 gacacaacca catcaaaaca cacagaaaga gacaccagaa ccccacaatc cactgcgctt
      481 gacaaaacca catcaaaaca cataacccaa cagcaatctc tccactcaac cacccccgaa
      541 aacacaccca actccacaca aacacccaca gcatccgagc cctccacatc aaattccacc
      601 caaaaactcc agtcatatgc ttag
//
LOCUS       KY328139                 624 bp    cRNA    linear   VRL 13-DEC-2016
DEFINITION  Human respiratory syncytial virus B isolate TH-CU431/2012
            attachment glycoprotein gene, partial cds.
ACCESSION   KY328139
VERSION     KY328139
KEYWORDS    .
SOURCE      Human respiratory syncytial virus B
  ORGANISM  Human respiratory syncytial virus B
            Viruses; ssRNA viruses; ssRNA negative-strand viruses;
            Mononegavirales; Pneumoviridae; Orthopneumovirus.
REFERENCE   1  (bases 1 to 624)
  AUTHORS   Thongpan,I., Mauleekoonphairoj,J., Vichiwattana,P., Korkong,S.,
            Vongpunsawad,S. and Poovorawan,Y.
  TITLE     Molecular Characterization of Respiratory Syncytial Virus in
            Thailand, 2012-2015
  JOURNAL   Unpublished
REFERENCE   2  (bases 1 to 624)
  AUTHORS   Thongpan,I., Mauleekoonphairoj,J., Vichiwattana,P., Korkong,S.,
            Vongpunsawad,S. and Poovorawan,Y.
  TITLE     Direct Submission
  JOURNAL   Submitted (13-DEC-2016) Department of Pediatrics, Center of
            Excellence in Clinical Virology, Faculty of Medicine, Chulalongkorn
            University, Bangkok 10330, Thailand
COMMENT     ##Assembly-Data-START##
            Assembly Method       :: DNASTAR-Lasergene v. 6
            Sequencing Technology :: Sanger dideoxy sequencing
            ##Assembly-Data-END##
```

```
FEATURES             Location/Qualifiers
     source          1..624
                     /organism="Human respiratory syncytial virus B"
                     /mol_type="viral cRNA"
                     /isolate="TH-CU431/2012"
                     /host="Homo sapiens"
                     /db_xref="taxon:208895"
                     /country="Thailand"
                     /collection_date="01-Aug-2012"
                     /note="group: B"
     CDS             <1..624
                     /note="G protein"
                     /codon_start=1
                     /product="attachment glycoprotein"
                     /protein_id="APY20480"
                     /translation="IHTNSATISPNTKSETHHTTAQTKGRTSTPTQNNKPSTKPRPKN
                     PPKKDDYHFEVFNFVPCSICGNNQLCKSICKTIPSNKPKKKPTIKPTNKPPTKTTNKR
                     DPKTLAKTPKKETTINPTKKPTPKTTERDTSTPQSTVLDTTTPKHTERDTSTPQSIAL
                     DTTTSKHTIQQQSLYSTTLENTPNSTQTPTASEPSTSNSTQKLQSYA"
ORIGIN
        1 atccacacaa actcagccac aatatcacct aatacaaaat cagaaacaca ccatacaaca
       61 gcacaaacca aaggcagaac ctctactcca acacagaaca acaagccaag cacaaaacca
      121 cgtccaaaaa atccaccaaa aaaagatgat taccattttg aagtgttcaa cttcgttccc
      181 tgtagtatat gtggcaacaa tcaactctgc aaatccattt gcaaaacaat accaagcaat
      241 aaaccaaaga aaaagccaac tataaaaccc acaaacaaac cacccaccaa aaccacaaac
      301 aaaagagacc ctaaaacact agccaaaaca ccgaaaaaag aaaccaccat taacccaaca
      361 aaaaaaccaa cccccaagac cacagaaaga gacaccagca ccccacaatc cactgtgctc
      421 gacacaacca caccaaaaca cacagaaaga gacaccagca ccccacaatc cattgcgctt
      481 gacacaacca catcaaaaca cacaatccaa cagcaatccc tctactcaac caccctcgaa
      541 aacacaccaa actccacaca aacacccaca gcatccgagc cctccacatc aaattccacc
      601 caaaaactcc agtcatatgc ttag
//
LOCUS       KY328140                 603 bp    cRNA    linear   VRL 13-DEC-2016
DEFINITION  Human respiratory syncytial virus B isolate CU_C5157/2014
            attachment glycoprotein gene, partial cds.
ACCESSION   KY328140
VERSION     KY328140
KEYWORDS    .
SOURCE      Human respiratory syncytial virus B
  ORGANISM  Human respiratory syncytial virus B
            Viruses; ssRNA viruses; ssRNA negative-strand viruses;
            Mononegavirales; Pneumoviridae; Orthopneumovirus.
REFERENCE   1  (bases 1 to 603)
  AUTHORS   Thongpan,I., Mauleekoonphairoj,J., Vichiwattana,P., Korkong,S.,
            Vongpunsawad,S. and Poovorawan,Y.
  TITLE     Molecular Characterization of Respiratory Syncytial Virus in
            Thailand, 2012-2015
  JOURNAL   Unpublished
REFERENCE   2  (bases 1 to 603)
  AUTHORS   Thongpan,I., Mauleekoonphairoj,J., Vichiwattana,P., Korkong,S.,
            Vongpunsawad,S. and Poovorawan,Y.
  TITLE     Direct Submission
  JOURNAL   Submitted (13-DEC-2016) Department of Pediatrics, Center of
            Excellence in Clinical Virology, Faculty of Medicine, Chulalongkorn
```

University, Bangkok 10330, Thailand
COMMENT ##Assembly-Data-START##
Assembly Method :: DNASTAR-Lasergene v. 6
Sequencing Technology :: Sanger dideoxy sequencing
##Assembly-Data-END##
FEATURES Location/Qualifiers
source 1..603
/organism="Human respiratory syncytial virus B"
/mol_type="viral cRNA"
/isolate="CU_C5157/2014"
/host="Homo sapiens"
/db_xref="taxon:208895"
/country="Thailand"
/collection_date="01-Oct-2014"
/note="group: B"
CDS <1..603
/note="G protein"
/codon_start=1
/product="attachment glycoprotein"
/protein_id="APY20481"
/translation="IHTNSATISPNTKSETHHTTAQTKGRTSTPTQNNKPSTKPRPKN
PPKKDDYHFEVFNFVPCSICGNNQLCKSICKTIPSNKPKKKPTIKPTNKPPTKTTNKR
DPKTLAKTPKKETTINPTKKPTPKTTERDTSTPQSTVLNTTTSKHTERDTSTSQSIAL
DTTTSKHTIQHQSLYSTTLENTLNSTQTPIASEPSTSNST"
ORIGIN
1 atccacacaa actcagccac aatatcaccc aatacaaaat cagaaacaca ccatacaaca
61 gcacaaacca aaggcagaac ctccactcca acacagaaca acaagccaag cacaaaacca
121 cgtccaaaaa atccaccaaa aaaagatgat taccattttg aagtgttcaa cttcgttccc
181 tgtagtatat gtggcaacaa tcaactctgc aaatccattt gcaaaacaat accaagcaat
241 aaaccaaaga aaaaaccaac tataaaaccc acaaacaaac cacccaccaa aaccacaaac
301 aaaagagacc ctaaaacact agccaaaaca ccgaaaaaag aaaccaccat taacccaaca
361 aaaaaaccaa ctcccaagac cacagaaaga gacaccagca ccccacaatc cactgtgctc
421 aacacaacca catcaaaaca cacagaaaga gacaccagca cctcacaatc cattgcgctt
481 gacacaacca catcaaaaca cacaatccaa catcaatccc tctactcaac caccctcgaa
541 aacacactaa actccacaca aacacccata gcatccgagc cctccacatc aaattccacc
601 taa
//
LOCUS KY328141 624 bp cRNA linear VRL 13-DEC-2016
DEFINITION Human respiratory syncytial virus B isolate TH-CU/C6141/2015
attachment glycoprotein gene, partial cds.
ACCESSION KY328141
VERSION KY328141
KEYWORDS .
SOURCE Human respiratory syncytial virus B
ORGANISM Human respiratory syncytial virus B
Viruses; ssRNA viruses; ssRNA negative-strand viruses;
Mononegavirales; Pneumoviridae; Orthopneumovirus.
REFERENCE 1 (bases 1 to 624)
AUTHORS Thongpan,I., Mauleekoonphairoj,J., Vichiwattana,P., Korkong,S.,
Vongpunsawad,S. and Poovorawan,Y.
TITLE Molecular Characterization of Respiratory Syncytial Virus in
Thailand, 2012-2015
JOURNAL Unpublished
REFERENCE 2 (bases 1 to 624)

```
  AUTHORS   Thongpan,I., Mauleekoonphairoj,J., Vichiwattana,P., Korkong,S.,
            Vongpunsawad,S. and Poovorawan,Y.
  TITLE     Direct Submission
  JOURNAL   Submitted (13-DEC-2016) Department of Pediatrics, Center of
            Excellence in Clinical Virology, Faculty of Medicine, Chulalongkorn
            University, Bangkok 10330, Thailand
COMMENT     ##Assembly-Data-START##
            Assembly Method       :: DNASTAR-Lasergene v. 6
            Sequencing Technology :: Sanger dideoxy sequencing
            ##Assembly-Data-END##
FEATURES             Location/Qualifiers
     source          1..624
                     /organism="Human respiratory syncytial virus B"
                     /mol_type="viral cRNA"
                     /isolate="TH-CU/C6141/2015"
                     /host="Homo sapiens"
                     /db_xref="taxon:208895"
                     /country="Thailand"
                     /collection_date="08-Oct-2015"
                     /note="group: B"
     CDS             <1..624
                     /note="G protein"
                     /codon_start=1
                     /product="attachment glycoprotein"
                     /protein_id="APY20482"
                     /translation="IHTNSATISPNTKSETHHTTAQTKGTTSTPTQNNKPSTKPRPKN
                     PPKKDDYHFEVFNFVPCSICGNNQLCKSICKTIPSNKPKKKPTTKPTNKPPTKTTNKR
                     DPKTLAKTPKKENTINPTKKPTPKTTERDTSTPQSTVLDITTSKHTERDTSTSQSIAL
                     DTTTSKHTTQQQSLYSTTPENTPNSTQTPTASEPSTSNSTQRLQSYA"
ORIGIN
        1 atccacacaa actcagccac aatatcaccc aatacaaaat cagaaacaca ccatacaaca
       61 gcacaaacca aaggcacaac ctctactcca acacagaaca acaagccaag cacaaaacca
      121 cgtccaaaaa atccaccaaa aaaagatgat taccattttg aagtgttcaa ctttgttccc
      181 tgtagtatat gtggcaacaa tcaactctgc aaatccattt gcaaaacaat accaagcaat
      241 aaaccaaaga aaaaaccaac tacaaaaccc acaaacaaac cacctaccaa aaccacaaac
      301 aaaagagacc ccaaaacact agccaaaaca ccgaaaaaag aaaacaccat taacccaaca
      361 aaaaaaccaa cccccaagac tacagaaaga gacacaagca ccccacaatc cactgttctc
      421 gacataacca catcaaaaca cacagaaaga gacaccagca cctcacaatc cattgcactt
      481 gacacaacca catcaaaaca cacaacccaa cagcaatctc tctactcaac cacccccgaa
      541 aacacaccca actccacaca aacacccaca gcatccgagc cctccacatc aaactctacc
      601 caaagactcc agtcatatgc ctag
//
LOCUS       KY328142                 603 bp    cRNA    linear   VRL 13-DEC-2016
DEFINITION  Human respiratory syncytial virus B isolate TH-CU/C5097/2015
            attachment glycoprotein gene, partial cds.
ACCESSION   KY328142
VERSION     KY328142
KEYWORDS    .
SOURCE      Human respiratory syncytial virus B
  ORGANISM  Human respiratory syncytial virus B
            Viruses; ssRNA viruses; ssRNA negative-strand viruses;
            Mononegavirales; Pneumoviridae; Orthopneumovirus.
REFERENCE   1  (bases 1 to 603)
  AUTHORS   Thongpan,I., Mauleekoonphairoj,J., Vichiwattana,P., Korkong,S.,
```

```
            Vongpunsawad,S. and Poovorawan,Y.
  TITLE     Molecular Characterization of Respiratory Syncytial Virus in
            Thailand, 2012-2015
  JOURNAL   Unpublished
REFERENCE   2  (bases 1 to 603)
  AUTHORS   Thongpan,I., Mauleekoonphairoj,J., Vichiwattana,P., Korkong,S.,
            Vongpunsawad,S. and Poovorawan,Y.
  TITLE     Direct Submission
  JOURNAL   Submitted (13-DEC-2016) Department of Pediatrics, Center of
            Excellence in Clinical Virology, Faculty of Medicine, Chulalongkorn
            University, Bangkok 10330, Thailand
COMMENT     ##Assembly-Data-START##
            Assembly Method       :: DNASTAR-Lasergene v. 6
            Sequencing Technology :: Sanger dideoxy sequencing
            ##Assembly-Data-END##
FEATURES             Location/Qualifiers
     source          1..603
                     /organism="Human respiratory syncytial virus B"
                     /mol_type="viral cRNA"
                     /isolate="TH-CU/C5097/2015"
                     /host="Homo sapiens"
                     /db_xref="taxon:208895"
                     /country="Thailand"
                     /collection_date="01-Sep-2015"
                     /note="group: B"
     CDS             <1..603
                     /note="G protein"
                     /codon_start=1
                     /product="attachment glycoprotein"
                     /protein_id="APY20483"
                     /translation="IHTNSATISPNTKSETHHTTAQTKGRTSTPTQNNKPSTKPRPKN
                     PPKKDDYHFEVFNFVPCSICGNNQLCKSICKTIPSNKPKKKPTIKPTNKTPTKTTNKR
                     DPKTPAKTPKKDTTTNPTKKPTPKTTERDTSTPQSTVLDTTTSKHTERDTSTSQSIVL
                     DTTTSKHTIQQQSLHSTTPGNTPNSTQTPTASEPSTSNST"
ORIGIN
        1 atccacacaa attcagccac aatatcacct aatacaaaat cagaaacaca ccatacaaca
       61 gcacaaacca aaggcagaac ctctactcca acacagaaca acaaaccaag cacaaaacca
      121 cgtccaaaaa atccaccaaa aaaagatgat taccattttg aagtgttcaa cttcgttccc
      181 tgcagtatat gtggcaacaa tcaactctgc aaatccattt gcaaaacaat accaagcaat
      241 aaaccaaaga aaaaaccaac cataaaacca acaaacaaaa cacccaccaa aaccacaaac
      301 aaaagagacc caaaaacacc agctaaaaca ccgaaaaaag acaccaccac caacccaaca
      361 aaaaaaccaa cccccaaaac cacagaaaga gacaccagca ccccacaatc cactgtgctc
      421 gacacaacca catcaaaaca cacagaaaga gacaccagca cctcacaatc tattgtgctc
      481 gacacaacca catcaaaaca cacaatccaa cagcaatccc tccactcaac cacccccgga
      541 aacacaccca actccacaca gacacccaca gcatccgagc cctccacatc aaattccacc
      601 taa
//
LOCUS       KY328143                 603 bp    cRNA    linear   VRL 13-DEC-2016
DEFINITION  Human respiratory syncytial virus B isolate TH-CU/C5159/2015
            attachment glycoprotein gene, partial cds.
ACCESSION   KY328143
VERSION     KY328143
KEYWORDS    .
SOURCE      Human respiratory syncytial virus B
```

```
  ORGANISM  Human respiratory syncytial virus B
            Viruses; ssRNA viruses; ssRNA negative-strand viruses;
            Mononegavirales; Pneumoviridae; Orthopneumovirus.
REFERENCE   1  (bases 1 to 603)
  AUTHORS   Thongpan,I., Mauleekoonphairoj,J., Vichiwattana,P., Korkong,S.,
            Vongpunsawad,S. and Poovorawan,Y.
  TITLE     Molecular Characterization of Respiratory Syncytial Virus in
            Thailand, 2012-2015
  JOURNAL   Unpublished
REFERENCE   2  (bases 1 to 603)
  AUTHORS   Thongpan,I., Mauleekoonphairoj,J., Vichiwattana,P., Korkong,S.,
            Vongpunsawad,S. and Poovorawan,Y.
  TITLE     Direct Submission
  JOURNAL   Submitted (13-DEC-2016) Department of Pediatrics, Center of
            Excellence in Clinical Virology, Faculty of Medicine, Chulalongkorn
            University, Bangkok 10330, Thailand
COMMENT     ##Assembly-Data-START##
            Assembly Method       :: DNASTAR-Lasergene v. 6
            Sequencing Technology :: Sanger dideoxy sequencing
            ##Assembly-Data-END##
FEATURES             Location/Qualifiers
     source          1..603
                     /organism="Human respiratory syncytial virus B"
                     /mol_type="viral cRNA"
                     /isolate="TH-CU/C5159/2015"
                     /host="Homo sapiens"
                     /db_xref="taxon:208895"
                     /country="Thailand"
                     /collection_date="08-Oct-2015"
                     /note="group: B"
     CDS             <1..603
                     /note="G protein"
                     /codon_start=1
                     /product="attachment glycoprotein"
                     /protein_id="APY20484"
                     /translation="IHTNSATISPNTKSETHHTTAQTKGRTSTPTQNNKPSTKPRPKN
                     PPKKDDYHFEVFNFVPCSICGNNQLCKSICKTIPSNKPKKKPTIKPTNKTPTKTTNKR
                     DPKTPAKTPKKDTTTNPTKKPTPKTTERDTSTPQSTVLDTTTSKHTERDTSTSQSIVL
                     DTTTSKHTIQQQSLHSTTPGNTPNSTQTPTASEPSTSNST"
ORIGIN      
        1 atccacacaa attcagccac aatatcacct aatacaaaat cagaaacaca ccatacaaca
       61 gcacaaacca aaggcagaac ctctactcca acacagaaca acaaaccaag cacaaaacca
      121 cgtccaaaaa atccaccaaa aaaagatgat taccattttg aagtgttcaa cttcgttccc
      181 tgcagtatat gtggcaacaa tcaactctgc aaatccattt gcaaaacaat accaagcaat
      241 aaaccaaaga aaaaaccaac cataaaacca acaaacaaaa cacccaccaa aaccacaaac
      301 aaaagagacc caaaaacacc agctaaaaca ccgaaaaaag acaccaccac caacccaaca
      361 aaaaaaccaa cccccaaaac cacagaaaga gacaccagca ccccacaatc cactgtgctc
      421 gacacaacca catcaaaaca cacagaaaga gacaccagca cctcacaatc tattgtgctc
      481 gacacaacca catcaaaaca cacaatccaa cagcaatccc tccactcaac cacccccgga
      541 aacacaccca actccacaca gacacccaca gcatccgagc cctccacatc aaattccacc
      601 taa
//
LOCUS       KY328144                 603 bp    cRNA    linear   VRL 13-DEC-2016
DEFINITION  Human respiratory syncytial virus B isolate TH-CU/C5063/2015
```

attachment glycoprotein gene, partial cds.
ACCESSION   KY328144
VERSION   KY328144
KEYWORDS   .
SOURCE   Human respiratory syncytial virus B
  ORGANISM Human respiratory syncytial virus B
            Viruses; ssRNA viruses; ssRNA negative-strand viruses;
            Mononegavirales; Pneumoviridae; Orthopneumovirus.
REFERENCE   1 (bases 1 to 603)
  AUTHORS   Thongpan,I., Mauleekoonphairoj,J., Vichiwattana,P., Korkong,S.,
            Vongpunsawad,S. and Poovorawan,Y.
  TITLE   Molecular Characterization of Respiratory Syncytial Virus in
            Thailand, 2012-2015
  JOURNAL   Unpublished
REFERENCE   2 (bases 1 to 603)
  AUTHORS   Thongpan,I., Mauleekoonphairoj,J., Vichiwattana,P., Korkong,S.,
            Vongpunsawad,S. and Poovorawan,Y.
  TITLE   Direct Submission
  JOURNAL   Submitted (13-DEC-2016) Department of Pediatrics, Center of
            Excellence in Clinical Virology, Faculty of Medicine, Chulalongkorn
            University, Bangkok 10330, Thailand
COMMENT   ##Assembly-Data-START##
            Assembly Method   :: DNASTAR-Lasergene v. 6
            Sequencing Technology :: Sanger dideoxy sequencing
            ##Assembly-Data-END##
FEATURES   Location/Qualifiers
     source   1..603
                     /organism="Human respiratory syncytial virus B"
                     /mol_type="viral cRNA"
                     /isolate="TH-CU/C5063/2015"
                     /host="Homo sapiens"
                     /db_xref="taxon:208895"
                     /country="Thailand"
                     /collection_date="10-Sep-2015"
                     /note="group: B"
     CDS   <1..603
                     /note="G protein"
                     /codon_start=1
                     /product="attachment glycoprotein"
                     /protein_id="APY20485"
                     /translation="IHTNSATISPNTKSETHHTTAQTKGRTSTPTQNNKPSTKPRPKN
                     PPKKDDYHFEVFNFVPCSICGNNQLCKSICKTIPSNKPKKKPTIKPTNKTPTKTTNKR
                     DPKTPAKTPKKDTTTNPTKKPTPKTTERDTSTPQSTVLDTTTSKHTERDTSTSQSIVL
                     DTTTSKHTIQQQSLHSTTPGNTPNSTQTPTASEPSTSNST"
ORIGIN
        1 atccacacaa attcagccac aatatcacct aatacaaaat cagaaacaca ccatacaaca
       61 gcacaaacca aaggcagaac ctctactcca acacagaaca acaaaccaag cacaaaacca
      121 cgtccaaaaa atccaccaaa aaaagatgat taccattttg aagtgttcaa cttcgttccc
      181 tgcagtatat gtggcaacaa tcaactctgc aaatccattt gcaaaacaat accaagcaat
      241 aaaccaaaga aaaaaccaac cataaaacca acaaacaaaa cacccaccaa aaccacaaac
      301 aaaagagacc caaaaacacc agctaaaaca ccgaaaaaag acaccaccac caacccaaca
      361 aaaaaaccaa cccccaaaac cacagaaaga gacaccagca ccccacaatc cactgtgctc
      421 gacacaacca catcaaaaca cacagaaaga gacaccagca cctcacaatc tattgtgctc
      481 gacacaacca catcaaaaca cacaatccaa cagcaatccc tccactcaac cacccccgga

```
     541 aacacaccca actccacaca gacacccaca gcatccgagc cctccacatc aaattccacc
     601 taa
//
LOCUS       KY328145                 624 bp    cRNA    linear   VRL 13-DEC-2016
DEFINITION  Human respiratory syncytial virus B isolate TH-CU402/2012
            attachment glycoprotein gene, partial cds.
ACCESSION   KY328145
VERSION     KY328145
KEYWORDS    .
SOURCE      Human respiratory syncytial virus B
  ORGANISM  Human respiratory syncytial virus B
            Viruses; ssRNA viruses; ssRNA negative-strand viruses;
            Mononegavirales; Pneumoviridae; Orthopneumovirus.
REFERENCE   1  (bases 1 to 624)
  AUTHORS   Thongpan,I., Mauleekoonphairoj,J., Vichiwattana,P., Korkong,S.,
            Vongpunsawad,S. and Poovorawan,Y.
  TITLE     Molecular Characterization of Respiratory Syncytial Virus in
            Thailand, 2012-2015
  JOURNAL   Unpublished
REFERENCE   2  (bases 1 to 624)
  AUTHORS   Thongpan,I., Mauleekoonphairoj,J., Vichiwattana,P., Korkong,S.,
            Vongpunsawad,S. and Poovorawan,Y.
  TITLE     Direct Submission
  JOURNAL   Submitted (13-DEC-2016) Department of Pediatrics, Center of
            Excellence in Clinical Virology, Faculty of Medicine, Chulalongkorn
            University, Bangkok 10330, Thailand
COMMENT     ##Assembly-Data-START##
            Assembly Method       :: DNASTAR-Lasergene v. 6
            Sequencing Technology :: Sanger dideoxy sequencing
            ##Assembly-Data-END##
FEATURES             Location/Qualifiers
     source          1..624
                     /organism="Human respiratory syncytial virus B"
                     /mol_type="viral cRNA"
                     /isolate="TH-CU402/2012"
                     /host="Homo sapiens"
                     /db_xref="taxon:208895"
                     /country="Thailand"
                     /collection_date="16-Aug-2012"
                     /note="group: B"
     CDS             <1..624
                     /note="G protein"
                     /codon_start=1
                     /product="attachment glycoprotein"
                     /protein_id="APY20486"
                     /translation="IHTNSATISPSTKSETHHTTAQTKGRTSTPTQNNKPSTKPRPKN
                     PPKKDDYHFEVFNFVPCSICGNNQLCKSICKTIPSNKPKKKPTIKPTNKTPTKTTNKR
                     DPKTPAKTPKKDTTTNPTKKPTPKTTERDTSTPQSTVLDTTTSKHTERDTSTSQSIVL
                     DTTTSKHTIQQQSLHSTTPGNTPNSTQTPTASEPSTSNSTQKLQSYA"
ORIGIN
        1 atccacacaa actcagccac aatatcaccc agtacaaaat cagaaacaca ccatacaaca
       61 gcacaaacca aaggcagaac ctccactcca acacagaaca acaaaccaag cacaaaacca
      121 cgtccaaaaa atccaccaaa aaaagatgat taccattttg aagtgttcaa cttcgttccc
      181 tgtagtatat gtggcaacaa tcaactctgc aaatccatct gcaaaacaat accaagcaat
```

```
     241 aaaccaaaga aaaaaccaac cataaaacca acaaacaaaa cacccaccaa aaccacaaac
     301 aaaagagacc caaaaacacc agccaaaaca ccgaaaaaag acaccaccac caacccaaca
     361 aaaaaaccaa cccccaagac cacagaaaga gacaccagca ccccacaatc cactgtgctc
     421 gacacaacca catcaaaaca cacagaaaga gacaccagca cctcacaatc tattgtgctc
     481 gacacaacca catcaaaaca cacaatccaa cagcaatccc tccactcaac cacccccgga
     541 aacacaccca actccacaca gacacccaca gcatccgagc cctccacatc aaattccacc
     601 caaaaactcc agtcatatgc ttag
//
LOCUS       KY328146                 609 bp    cRNA    linear   VRL 13-DEC-2016
DEFINITION  Human respiratory syncytial virus B isolate TH-CU/B12640/2015
            attachment glycoprotein gene, partial cds.
ACCESSION   KY328146
VERSION     KY328146
KEYWORDS    .
SOURCE      Human respiratory syncytial virus B
  ORGANISM  Human respiratory syncytial virus B
            Viruses; ssRNA viruses; ssRNA negative-strand viruses;
            Mononegavirales; Pneumoviridae; Orthopneumovirus.
REFERENCE   1  (bases 1 to 609)
  AUTHORS   Thongpan,I., Mauleekoonphairoj,J., Vichiwattana,P., Korkong,S.,
            Vongpunsawad,S. and Poovorawan,Y.
  TITLE     Molecular Characterization of Respiratory Syncytial Virus in
            Thailand, 2012-2015
  JOURNAL   Unpublished
REFERENCE   2  (bases 1 to 609)
  AUTHORS   Thongpan,I., Mauleekoonphairoj,J., Vichiwattana,P., Korkong,S.,
            Vongpunsawad,S. and Poovorawan,Y.
  TITLE     Direct Submission
  JOURNAL   Submitted (13-DEC-2016) Department of Pediatrics, Center of
            Excellence in Clinical Virology, Faculty of Medicine, Chulalongkorn
            University, Bangkok 10330, Thailand
COMMENT     ##Assembly-Data-START##
            Assembly Method       :: DNASTAR-Lasergene v. 6
            Sequencing Technology :: Sanger dideoxy sequencing
            ##Assembly-Data-END##
FEATURES             Location/Qualifiers
     source          1..609
                     /organism="Human respiratory syncytial virus B"
                     /mol_type="viral cRNA"
                     /isolate="TH-CU/B12640/2015"
                     /host="Homo sapiens"
                     /db_xref="taxon:208895"
                     /country="Thailand"
                     /collection_date="15-Aug-2015"
                     /note="group: B"
     CDS             <1..609
                     /note="G protein"
                     /codon_start=1
                     /product="attachment glycoprotein"
                     /protein_id="APY20487"
                     /translation="IYTSSATISPNTKSETHHTTAQTKSRITTPTQNNKPSTKPRPKN
                     PPKKPKDDYHFEVFNFVPCSICGNNQLCKSICKTIPSNKPKKKPTTKPTTKPPTKTTN
                     KRDPKTPVKPLKKENTINPTKKPTPRITERDTSTPQSTLLDTTASKHTEIDTSTPQSI
                     VLDTTTSKHTIRQQSLHSTTPENTPNPTQTPTASEPSTSNSY"
```

```
ORIGIN
        1 atctacacaa gctcagccac aatatcacct aatacaaaat cagaaacaca ccatacaaca
       61 gcacaaacca aaagcagaat caccactcca acacagaaca acaagccaag cacaaaacca
      121 cgtccaaaaa atccaccaaa aaaaccaaaa gatgattacc attttgaagt gttcaacttc
      181 gttccctgta gtatatgtgg caacaatcaa ctctgcaaat ccatttgcaa aacaatacca
      241 agcaataaac caaagaaaaa accaaccaca aaacccacaa ccaaaccacc caccaaaacc
      301 acaaacaaaa gagacccaaa aacaccagtc aaaccactga aaaaagaaaa caccatcaac
      361 ccaacaaaaa aaccaacccc caggatcaca gaaagagaca ccagcacccc acaatccact
      421 ctgctcgaca caaccgcatc aaaacacaca gaaatagaca ccagcacccc acaatccatt
      481 gtgctcgaca caaccacatc aaaacacaca atccgacagc aatccctcca ctcaaccacc
      541 cccgaaaaca cacccaaccc cacacaaaca cccacagcat ccgagccctc cacatcaaac
      601 tcctactaa
//
LOCUS       KY328147                 609 bp    cRNA    linear   VRL 13-DEC-2016
DEFINITION  Human respiratory syncytial virus B isolate TH-CU/C6143/2015
            attachment glycoprotein gene, partial cds.
ACCESSION   KY328147
VERSION     KY328147
KEYWORDS    .
SOURCE      Human respiratory syncytial virus B
  ORGANISM  Human respiratory syncytial virus B
            Viruses; ssRNA viruses; ssRNA negative-strand viruses;
            Mononegavirales; Pneumoviridae; Orthopneumovirus.
REFERENCE   1  (bases 1 to 609)
  AUTHORS   Thongpan,I., Mauleekoonphairoj,J., Vichiwattana,P., Korkong,S.,
            Vongpunsawad,S. and Poovorawan,Y.
  TITLE     Molecular Characterization of Respiratory Syncytial Virus in
            Thailand, 2012-2015
  JOURNAL   Unpublished
REFERENCE   2  (bases 1 to 609)
  AUTHORS   Thongpan,I., Mauleekoonphairoj,J., Vichiwattana,P., Korkong,S.,
            Vongpunsawad,S. and Poovorawan,Y.
  TITLE     Direct Submission
  JOURNAL   Submitted (13-DEC-2016) Department of Pediatrics, Center of
            Excellence in Clinical Virology, Faculty of Medicine, Chulalongkorn
            University, Bangkok 10330, Thailand
COMMENT     ##Assembly-Data-START##
            Assembly Method       :: DNASTAR-Lasergene v. 6
            Sequencing Technology :: Sanger dideoxy sequencing
            ##Assembly-Data-END##
FEATURES             Location/Qualifiers
     source          1..609
                     /organism="Human respiratory syncytial virus B"
                     /mol_type="viral cRNA"
                     /isolate="TH-CU/C6143/2015"
                     /host="Homo sapiens"
                     /db_xref="taxon:208895"
                     /country="Thailand"
                     /collection_date="08-Oct-2015"
                     /note="group: B"
     CDS             <1..609
                     /note="G protein"
                     /codon_start=1
                     /product="attachment glycoprotein"
```

```
                     /protein_id="APY20488"
                     /translation="IYTSSATISPNTKSETHHTTAQTKSRITTPTQNNKPSTKPRPKN
                     PPKKPKDDYHFEVFNFVPCSICGNNQLCKSICKTIPSNKPKKKPTTKPTTKPPTKTTN
                     KRDPKTPVKPLKKENTINPTKKPTPRITERDTSTPQSTLLDTTASKHTEIDTSTPQSI
                     VLDTTTSKHTIRQQSLHSTTPENTPNPTQTPTASEPSTSNSY"
ORIGIN
        1 atctacacaa gctcagccac aatatcacct aatacaaaat cagaaacaca ccatacaaca
       61 gcacaaacca aaagcagaat caccactcca acacagaaca acaagccaag cacaaaacca
      121 cgtccaaaaa atccaccaaa aaaaccaaaa gatgattacc attttgaagt gttcaacttc
      181 gttccctgta gtatatgtgg caacaatcaa ctctgcaaat ccatttgcaa aacaatacca
      241 agcaataaac caaagaaaaa accaaccaca aaacccacaa ccaaaccacc caccaaaacc
      301 acaaacaaaa gagacccaaa aacaccagtc aaaccactga aaaaagaaaa caccatcaac
      361 ccaacaaaaa aaccaacccc caggatcaca gaaagagaca ccagcacccc acaatccact
      421 ctgctcgaca caaccgcatc aaaacacaca gaaatagaca ccagcacccc acaatccatt
      481 gtgctcgaca caaccacatc aaaacacaca atccgacagc aatccctcca ctcaaccacc
      541 cccgaaaaca cacccaaccc cacacaaaca cccacagcat ccgagccctc cacatcaaac
      601 tcctactaa
//
LOCUS       KY328148                 609 bp    cRNA    linear   VRL 13-DEC-2016
DEFINITION  Human respiratory syncytial virus B isolate TH-CU413/2012
            attachment glycoprotein gene, partial cds.
ACCESSION   KY328148
VERSION     KY328148
KEYWORDS    .
SOURCE      Human respiratory syncytial virus B
  ORGANISM  Human respiratory syncytial virus B
            Viruses; ssRNA viruses; ssRNA negative-strand viruses;
            Mononegavirales; Pneumoviridae; Orthopneumovirus.
REFERENCE   1  (bases 1 to 609)
  AUTHORS   Thongpan,I., Mauleekoonphairoj,J., Vichiwattana,P., Korkong,S.,
            Vongpunsawad,S. and Poovorawan,Y.
  TITLE     Molecular Characterization of Respiratory Syncytial Virus in
            Thailand, 2012-2015
  JOURNAL   Unpublished
REFERENCE   2  (bases 1 to 609)
  AUTHORS   Thongpan,I., Mauleekoonphairoj,J., Vichiwattana,P., Korkong,S.,
            Vongpunsawad,S. and Poovorawan,Y.
  TITLE     Direct Submission
  JOURNAL   Submitted (13-DEC-2016) Department of Pediatrics, Center of
            Excellence in Clinical Virology, Faculty of Medicine, Chulalongkorn
            University, Bangkok 10330, Thailand
COMMENT     ##Assembly-Data-START##
            Assembly Method       :: DNASTAR-Lasergene v. 6
            Sequencing Technology :: Sanger dideoxy sequencing
            ##Assembly-Data-END##
FEATURES             Location/Qualifiers
     source          1..609
                     /organism="Human respiratory syncytial virus B"
                     /mol_type="viral cRNA"
                     /isolate="TH-CU413/2012"
                     /host="Homo sapiens"
                     /db_xref="taxon:208895"
                     /country="Thailand"
                     /collection_date="16-Aug-2012"
```

```
                     /note="group: B"
     CDS             <1..609
                     /note="G protein"
                     /codon_start=1
                     /product="attachment glycoprotein"
                     /protein_id="APY20489"
                     /translation="IHTNSATISPNKKSETHHTTVQTKSRTTTPTQNNKPSTKPRPKN
                     PPKKPKDDYHFEVFNFVPCSICGNNQLCKSICKTIPSNKPKKKPTTKPTNKPPTKTTN
                     KRDPKTPAKPLKKENTINPTKKPTPRITERDTSTPQSTLLDTTASKHTERDTSTLQST
                     VLDTTTSKHTIRQQSLYSTTPENTPNSTQTPTASEPSTSNSN"
ORIGIN
        1 atccacacaa actcagccac aatatcacct aataaaaaat cagaaacaca ccatacaaca
       61 gtacaaacca aaagcagaac caccactcca acacagaaca acaagccaag cacaaaacca
      121 cgtccaaaaa atccaccaaa aaaaccaaaa gatgattacc attttgaagt gttcaacttc
      181 gttccctgta gtatatgtgg caacaatcaa ctctgcaaat ccatttgcaa aacaatacca
      241 agcaataaac caaagaaaaa accaaccaca aaacccacaa acaaaccacc taccaaaacc
      301 acaaacaaaa gagacccaaa aacaccagcc aaaccactga aaaaagaaaa taccatcaac
      361 ccaacaaaaa aaccaacccc caggatcaca gaaagagaca ccagcacccc acaatccact
      421 ttgctcgaca caaccgcatc aaaacacaca gaaagagaca ccagcaccct acaatccact
      481 gtgctcgaca caaccacatc aaaacacaca atccgacagc aatccctcta ctcaaccacc
      541 cccgaaaaca cacccaactc cacacaaaca cccacagcat ccgagccctc cacatcaaac
      601 tccaactaa
//
```

file:///D|/Final%20peerJ/GenBank%20KY327937-KY328148_.txt[3/10/2560 14:59:50]
