# Supplementary material for: Respiratory syncytial virus genotypes NA1, ON1, and BA9 are prevalent in Thailand, 2012–2015
Source: PeerJ. 2017 Oct 27;5:e3970. doi: 10.7717/peerj.3970 (PMC5661434; doi:10.7717/peerj.3970)
Supplement: Table S1 [file peerj-05-3970-s002.docx]

**Supplemental Table S1. Intra-genotype and inter-genotype genetic diversity (measured by *p*-distance) among RSV-A and RSV-B strains in this study.**

|  |  | **RSV-A** | | | **RSV-B** | | |
| --- | --- | --- | --- | --- | --- | --- | --- |
|  | **Genotypes** | **NA1** | **ON1** | **NA3** | **BA9** | **BA10** | **BA-C** |
| **RSV-A** | **NA1** | 0.004-0.060 | 0.050 | 0.062 | - | - | - |
|  | **ON1** | - | 0.003-0.060 | 0.083 | - | - | - |
|  | **NA3** | - | - | N/A | - | - | - |
| **RSV-B** | **BA9** | - | - | - | 0-0.670 | 0.062 | 0.090 |
|  | **BA10** | - | - | - | - | 0.010 | 0.080 |
|  | **BA-C** | - | - | - | - | - | 0.030 |
